# A systematic dissection of determinants and consequences of snoRNA-guided pseudouridylation of human mRNA

**DOI:** 10.1093/nar/gkac347

**Published:** 2022-05-10

**Authors:** Ronit Nir, Thomas Philipp Hoernes, Hiromi Muramatsu, Klaus Faserl, Katalin Karikó, Matthias David Erlacher, Aldema Sas-Chen, Schraga Schwartz

**Affiliations:** Department of Molecular Genetics, Weizmann Institute of Science, Rehovot 7610001, Israel; Institute of Genomics and RNomics, Medical University of Innsbruck, 6020 Innsbruck, Austria; Department of Neurosurgery, University of Pennsylvania, Philadelphia, PA, USA; Department of Microbiology, University of Pennsylvania, Philadelphia, PA, USA; Institute of Clinical Biochemistry, Biocenter, Medical University of Innsbruck, 6020 Innsbruck, Austria; Department of Neurosurgery, University of Pennsylvania, Philadelphia, PA, USA; BioNTech RNA Pharmaceuticals, Mainz, Germany; Institute of Genomics and RNomics, Medical University of Innsbruck, 6020 Innsbruck, Austria; Department of Molecular Genetics, Weizmann Institute of Science, Rehovot 7610001, Israel; The Shmunis School of Biomedicine and Cancer Research, The George S. Wise Faculty of Life Sciences, Tel Aviv University, Tel Aviv, Israel; Department of Molecular Genetics, Weizmann Institute of Science, Rehovot 7610001, Israel

## Abstract

RNA can be extensively modified post-transcriptionally with >170 covalent modifications, expanding its functional and structural repertoire. Pseudouridine (Ψ), the most abundant modified nucleoside in rRNA and tRNA, has recently been found within mRNA molecules. It remains unclear whether pseudouridylation of mRNA can be snoRNA-guided, bearing important implications for understanding the physiological target spectrum of snoRNAs and for their potential therapeutic exploitation in genetic diseases. Here, using a massively parallel reporter based strategy we simultaneously interrogate Ψ levels across hundreds of synthetic constructs with predesigned complementarity against endogenous snoRNAs. Our results demonstrate that snoRNA-mediated pseudouridylation can occur on mRNA targets. However, this is typically achieved at relatively low efficiencies, and is constrained by mRNA localization, snoRNA expression levels and the length of the snoRNA:mRNA complementarity stretches. We exploited these insights for the design of snoRNAs targeting pseudouridylation at premature termination codons, which was previously shown to suppress translational termination. However, in this and follow-up experiments in human cells we observe no evidence for significant levels of readthrough of pseudouridylated stop codons. Our study enhances our understanding of the scope, ‘design rules’, constraints and consequences of snoRNA-mediated pseudouridylation.

## INTRODUCTION

Following their synthesis, nucleosides in RNA can be covalently modified with >170 modifications, providing a rich reservoir for modulating RNA structure and function. Nucleoside modifications in RNA were traditionally studied within the context of the highly expressed and hence biochemically tractable tRNAs and rRNAs, where they are also highly abundant. Advances in genomic approaches in recent years have revealed that some modifications are also present in other classes of RNA, including mRNA (1–5). Modifications of mRNA are of particular interest, as they harbor the potential of modulating different aspects of the mRNA function (e.g. stability, localization, translation) and thereby regulating translation of the encoded protein.

The most abundant modified nucleoside in human total RNA is pseudouridine (Ψ) (6,7), present at >100 sites on the rRNA, and at numerous sites on tRNA and snRNA. The human genome encodes 13 different Ψ synthases (PUSs), catalyzing this modification via two different mechanisms. Nearly all of them likely act in a site-specific manner, by both recognizing and modifying their targets. However, one PUS, DKC1, is guided towards its target sites via small H/ACA box nucleolar RNAs (snoRNAs) (8). H/ACA box snoRNAs are short (120–140-nt) sequences, each harboring up to two hairpin ‘arms’, containing a large internal loop called a ’pseudouridylation pocket’ (8) with sequence complementarity of various lengths (between 2 and 11nt on each side) to target sites, typically on the rRNA. The snoRNAs are bound by DKC1 (and additional proteins) as part of a snoRNP complex and direct it towards precise targets (9–11).

In recent years it has been recognized that Ψ is also highly abundant in mRNA (12,13). Four studies have developed conceptually similar genomic approaches for transcriptome-wide mapping of pseudouridylated targets within mRNA (12,14–16). These approaches all relied on pre-treatment of purified mRNA with *N*-cyclohexyl-*N*′-(β-[*N*-methylmorpholino]ethyl)carbodiimide *p*-toluenesulfonate salt (CMC), a bulky residue that selectively binds to pseudouridylated sites but cannot be transversed by reverse transcriptase, leading to truncated cDNAs that end precisely one base downstream of the pseudouridylated target. These truncated products were captured via high-throughput sequencing, and served as a basis for inference of hundreds to thousands of Ψ-harboring sites in human and yeast mRNAs.

Interestingly, the PUSs responsible for catalysis of Ψ in mRNA targets have only been identified for a minority of highly modified sites. These sites were found to be modified primarily via three site-specific Ψ synthases (TRUB1, PUS7 and PUS1) (12,17,18). The majority of putatively identified pseudouridine sites have not been assigned with a pseudouridine synthase, suggesting that additional PUSs might have activity on mRNA, including DKC1 which has been implicated in potential pseudouridylation of a small number of sites (14). A main challenge in establishing such enzyme:substrate relationships is the limitations of our ability to robustly and systematically monitor presence of Ψ. Sensitive and site-specific detection of Ψ requires immense sequencing depth which is unrealistic for the majority of sites in mRNA, in particular ones modified at lower stoichiometries. As a consequence, pseudouridylation mapping at a transcriptome-wide scale is associated with considerable levels of both false positives and false negatives, which has led to poor overlap in Ψ detection between studies (17–20), and renders it challenging to compare pseudouridylation maps of mRNAs in WT versus PUS-depleted samples.

A dissection of the targeting scope of H/ACA box snoRNA is of considerable interest for both basic science and applied reasons. First, there are 76 ‘orphan’ snoRNAs encoded in the human genome, roughly half of which form the H/ACA class (21). Orphan snoRNAs lack identifiable complementarity stretches towards the rRNA and some of them are implicated in human disease (22–24). Thus it is tempting to speculate that they may serve to guide modifications of other classes of RNA, such as mRNA. Indeed, there is considerable evidence for interactions forming between snoRNAs and mRNAs (25–29) in addition to limited evidence also for snoRNA-directed modifications on mRNA (14,30). Second, if snoRNA-guided pseudouridylation of mRNA can occur, then understanding the rules guiding such activity can be exploited therapeutically. It was previously shown that replacement of uridine (U) by Ψ in the context of a termination codon (e.g. UAA→ΨAA) leads to robust read-through into downstream regions (31–33). An ability to introduce read-through could serve as a powerful therapeutic strategy to overcome diverse genetic disease caused by premature termination codons (PTCs), such as Duchenne muscular dystrophy, cystic fibrosis and spinal muscular atrophy (34). Thus, administration of exogenous tailor-designed snoRNAs targeting these PTCs could serve as an attractive therapeutic strategy allowing nonsense suppression and translation of full-length proteins.

Here, we investigate the *potential* for snoRNAs-guided pseudouridylation of mRNAs, the *rules* underlying this activity, the *constraints* limiting snoRNA-guided pseudouridylation, and the *functional consequences* of mRNA pseudouridylation. Specifically, we designed and expressed within cells hundreds of reporter-constructs, each of which with a predefined complementarity towards one of dozens of endogenously expressed snoRNAs. Our results demonstrate that snoRNA-mediated pseudouridylation can occur on mRNA targets. Nonetheless, we find that pseudouridylation is typically achieved at relatively low levels. We identify three factors constraining snoRNA-guided pseudouridylation of mRNA: subcellular localization of the targeted mRNA, expression levels of the snoRNA and the length of the snoRNA:mRNA complementarity stretches. We exploit these insights to design snoRNAs targeting pseudouridylation at two PTCs genetically introduced in the context of Duchenne muscular dystrophy and cystic fibrosis. We were successfully able to introduce Ψ at an intended target site, but find no evidence for significant levels of readthrough of pseudouridylated stop codons, even when we synthetically introduce Ψ at stoichiometric levels. Our results thus expand our technical abilities to interrogate snoRNA-mediated pseudouridylation, enhance our understanding of snoRNA targets and determinants of specificity, and suggest that there are contexts in which pseudouridine-mediated readthrough does not occur at appreciable levels.

## MATERIALS AND METHODS

### Plasmids and cell lines

pZDonor FC plasmid was a gift from Ilya Vainberg Slutskin and Prof. Eran Segal (Weizmann Institute of Science). phPol1Ex plasmid was a gift from Prof. Tetsuro Hirose (Hokkaido University). pmTurquoise2-H2A was a gift from Dorus Gadella (Addgene plasmid #36207). EGFP-DMD-UGA-dTomato and EGFP-CFTR-UAA-dTomato were a gift from Prof. Warren Tate (University of Otago). HEK-293T, A549 and MCF7 cells were grown in DMEM supplemented with 10% FBS and 1% penicillin–streptomycin solution. K562 cells were grown in Iscove's media supplemented with 10% FBS and 1% penicillin–streptomycin solution. All cell lines were obtained from ATCC.

### Design of massively parallel reporter assay (MPRA) library

The MPRA design included two libraries: First, a synthetic library in which targets for 68 snoRNAs were designed by varying the degree of complementarity of the target to the binding loop of the snoRNA, and keeping the non-paired base downstream to the modified uridine as a guanosine (based on snoRNA:target pairing from https://www-snorna.biotoul.fr//). The complementarity levels were ‘3nt’–3nt upstream of the uridine and following guanosine and two downstream (3,2), ‘5nt’–5nt upstream and four downstream (5,4), ‘8nt’–(8,8) or ‘10nt’–(10,10). In addition, point mutations (from guanosine to cytidine, adenosine or uridine) or a deletion of the first position following the pseudouridylation site were designed for a subset of the snoRNAs only in costructs with 10nt complementarity. As a negative control for each 10nt complementarity construct the modified uridine was changed into a guanosine. Second, a native rRNA and snRNA library in which each construct of the library represents a 75 nt long fragment from the native RNA sequences in which each known Ψ site, as annotated in the MODOMICS database (35), was positioned in position 42. For each construct a negative control was designed by mutating the modified uridine into a guanosine. Sequences whose design generated a restriction site of the enzyme used for cloning the libraries into the pZDonor FC plasmid were omitted. Overall, 606 and 216 constructs were designed for the synthetic and native RNA libraries, respectively (Supplementary Table S1).

### Cloning of the MPRA library

#### Cloning to a Pol-II promoter plasmid

The pool of sequences was synthesized by Twist Bioscience and cloned to pZDonor FC (36) as a 3’UTR downstream of a reporter gene. The cloning was performed essentially as described in (37). Specifically, the library was amplified in 5 different PCR reactions, each using 30pg as a template and 14 cycles. The reactions were combined, cleaned by QIAquick PCR purification kit (Qiagen), and a total of 540 ng was cut by AscI and SpeI restriction enzymes (FastDigest, Thermo Scientific). Following electro-elution from a gel using Midi GeBAflex tubes (GeBA, Kfar Hanagid, Israel), the library was ligated (in 1:1 ratio) to 150ng pZDonor FC plasmid digested by AscI and SpeI, using CloneDirect Rapid Ligation kit (Lucigen Corporation) and transformed into E. coli 10G electrocompetent cells (Lucigen) in a single cuvette. The bacteria were grown on five 14cm plates, reaching in average 500 colonies per each sequence variant.

#### Cloning to a Pol-I promoter plasmid

The library was similarly cloned to phPol1Ex plasmid (38) using HindIII and BamHI restriction enzymes. The bacteria were grown on five 14cm plates, and reached in average 45 colonies per each sequence variant.

### Targeted measurement of pseudouridylation within the construct

A 10-cm plate of HEK-293T cells was transiently transfected with 5 μg plasmids (either the library plasmid alone or divided between the library plasmid and other plasmids) using PolyJet reagent (Signagen Laboratories). RNA was purified using Nucleozol reagent (Macherey Nagel). Sequence specific Ψ-seq was performed on total RNA essentially as described in Safra *et al.* (17), reverse-transcription was carried out from a constant sequence stemming from either of the plasmids (Pol-I and Pol-II). Adapter ligation to the cDNA was carried out as described, followed by PCR enrichment with an inner library subset specific primer, carrying indexed Illumina adapters (primers used for the amplification were: 2P universal and Indexed pool specific (for example G10) (Supplementary Table S8). Libraries were sequenced on Illumina NextSeq 500 or NovaSeq 6000 platforms generating short paired-end reads, ranging from 37 to 51 bp from each end. Overall four experiments were carried out for quantification of Ψ levels in constructs from the MPRA libraries as follows: Experiment #1: two samples of Pol-I- and two samples of Pol-II-driven libraries transfected into HEK-293T cells, each treated with CMC or input. Experiment #2: three samples of Pol-II-driven libraries co-transfected with ACA21 and ACA61 overexpressing plasmids, or with a control (empty) overexpression plasmid or with no additional vectors. Each sample was treated with CMC. Experiment #3: two samples of Pol-II-driven libraries co-transfected with either control or DKC1-targeting siRNAs, each treated with CMC or input. Experiment #4: a single sample from either HEK-293T, A549, MCF7 or K562 was transfected with a Pol-II-driven library and treated with CMC or input. This setup is further presented in Supplementary Figure S1A and in Supplementary Table S2.

### Read mapping and Ψ quantification

Detection of Ψ was performed essentially as described in (12,14–16). A custom reference transcriptome was generated, comprising endogenous rRNA sequences as well as each of the variable 822 sequences comprising the two MPRA libraries embedded within a 328-bp target environment in the plasmid, into which it was cloned (sequences are found in Supplementary Table S1). Paired-end reads were aligned to the custom reference transcriptome, using the STAR aligner (version 2.5.3a) (39), without allowing introns, using the parameters ‘ –alignIntronMax 1’. Resulting bam files were filtered via samtools to retain only read pairs with mapping quality = 255, which fall within the expected boundaries of the PCR insert.

Filtered bam files were analyzed via the bam2ReadEnds.R code published by Garcia-Campos et al. (40) (DOI: 10.5281/zenodo.3581426) to quantify the number of reads starting and overlapping each position. A custom script (available at https://github.com/aldemasas/pseudouridine) was subsequently used to calculate Ψ-scores, corresponding to the number of reads beginning at the position divided by the overall number of reads covering it. Importantly, Ψ level of a specific position in the transcriptome is evaluated based on the Ψ-score calculated for the position downstream to it. Downstream analysis included only sites with coverage ≥100 reads, although all sites are present in Supplementary Table S2.

### Reporters for assessment of the effect of nearby nucleotides on the ability to detect psi using CMC assay

#### Design

A 157nt reporter, composed of a spacer, a T7 promoter, a 40nt sequence containing only A/G/C, a single T followed by 6 V mixed nucleotides (A/G/C), and 64 additional A/G/C only sequence, followed by a sequence complementary to the rTd RT primer, was assembled from the two DNA oligos (IDT) Psi-reporter-FW and Psi-reporter-RV, sharing a 17-nt complementation sequence (Supplementary Table S8). The assembly was carried out using KOD hot start DNA polymerase (Merck Millipore). The correct size fragment was cut from a gel, purified by Qiaquick gel extraction kit (Qiagen) and cleaned from salts using Kapa pure beads (Roche). 2 pmol DNA was used for *in vitro* transcription, carried out using T7 Megascript transcription kit (Thermo Fisher Scientific), following the manufacturer's protocol, with either UTP or same amount of Pseudouridine-5'-Triphosphate (TriLink). Following an 11 h incubation in 37°C, the RNA was treated with DNase and cleaned on RNA clean and concentrator columns (Zymo research). Ψ containing RNA was mixed with U containing RNA in ratios of 20%, 40%, 60%, 80% or 100%, and the pools were treated with CMC in BEU buffer, essentially as in (14), except cleanups following the CMC treatment and sodium bicarbonate incubation were performed on Dynabeads MyOne Silane beads (Thermo Fisher scientific). Library preparation, including cDNA synthesis, RNA hydrolysis, adapter ligation to the cDNA and PCR enrichment, was carried out as in (14) using SuperScript III reverse transcriptase (Thermo Fisher scientific).

#### Analysis

**Figure 1.**
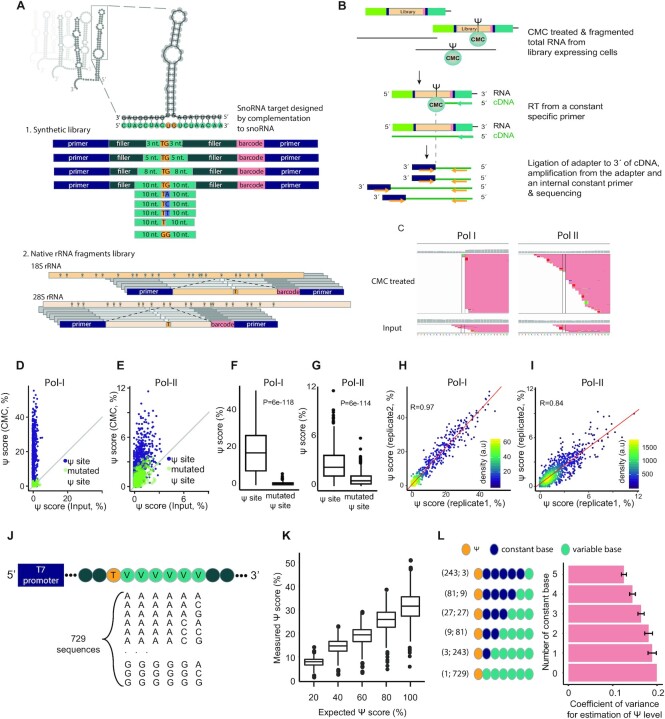
Massively parallel reporter assay-based method for quantification of Ψ at broad-range stoichiometries. (**A**) Design of the snoRNAs’ targets library. 1. Synthetic library—targets for 68 snoRNAs were designed by varying the degree of complementarity of the target to the binding loop of the snoRNA, from upto 3 to 10 nt from each side of the Ψ site (Materials and Methods). In addition, point mutations or a deletion of the first position following the pseudouridylation site were designed for part of the snoRNAs (see Materials and Methods). For each snoRNA, an additional variant with 10 nt-long complementarity was designed in which the modified uridine was mutated into a guanosine. 2. Native rRNA fragments library—each library variant was designed in a manner positioning a known Ψ site in ribosomal RNA sequences or snRNAs in a constant position. (**B**) The libraries were transfected into HEK-293T cells, and total RNA was extracted, treated with CMC and used for NGS library preparation. A library specific Ψ-Seq protocol (17) was used to identify reverse transcription stops, an estimate for Ψ levels. (**C**) Representative data of one of the snoRNA targets (E2 snoRNA). A significant pileup of reverse transcription stops is present in the expected site (highlighted by parallel lines) when the library is treated with CMC compared to control (Input) samples. The transcription stops are more prevalent in Pol-I-driven constructs. (**D**, **E**) Library specific Ψ-Seq was conducted on HEK-293T samples transfected with Pol-I- or Pol-II-promoter libraries and treated with CMC or input (*n* = 2). Shown are native rRNA and snRNA sites and synthetic sites with 8 or 10 nt-long complementarity to a known snoRNA (purple) and their counterparts, in which the modified uridine was mutated (green). Grey lines represent a positive correlation of one between conditions. (**F**, **G**) Ψ-score of native rRNA and snRNA sites and synthetic sites with 10 nt-long complementarity to a known snoRNA and counterpart sites mutated in the modified uridine, in samples treated with CMC. P values represent results of two-tailed paired *t*-tests. (**H**, **I**) Pearson's correlation between the Ψ-score of two biologically independent samples. Regression line in red. Kernel density estimate of data distribution in arbitrary units (a.u). (**J**) Design of the in vitro transcription library containing 729 sequences, each encoding a single thymidine (T; yellow) followed by six varying bases (V; light green). The seven bases are embedded in a backbone of 111 nts and flanked by a spacer, a T7 promoter and a sequence used for priming of reverse transcription. Varying bases represent all possible combinations of adenine, cytidine and guanine. (**K**) Sequences from (J) were transcribed in the presence of uridine or pseudouridine, and mixed to generate the indicated ratios (x-axis), followed by treatment with CMC and generation of libraries to assess individual Ψ scores. (**L**) Coefficient of variance, allowing to assess the effect of downstream bases on measured Ψ scores, was calculated on subsets of sequences from **K**, which were transcribed using 100% Ψ. The bottom bar represents the maximal coefficient of variance calculated for a single group with all 729 sequences (1;729 in brackets), the top bar represents an averaged coefficient of variance calculated based on binning all sequences into 243 groups each with 3 sequences (243;3 in brackets), where each group had a fixed pattern of 5 bases downstream of the Ψ site, followed by a variable base (A, C or G) at the 6th position. Error bars represent standard error of the mean; For all box plots in the figure, the center line indicates the median, the box boundaries mark the 25th and 75th percentiles, the whiskers indicate ±1.5 × the interquartile range (IQR) and outliers are shown as individual dots.

Ψ-scores were calculated as described above to assess the Ψ level of individual reporter sequences in distinct % of Ψ. For the analysis in Figure 1L only reporters generated by incorporation of 100% Ψ were used. In short, coefficient of variance was calculated for subsets of the reporter sequences in order to assess the effect of nearby bases of quantified Ψ signals. First, to assess the maximal variance in Ψ estimation, coefficient of variance was calculated for all 729 sequences grouped into a single group. To assess the contribution of the 1st base downstream to the Ψ site, sequences were grouped into 3 groups each harboring a fixed nucleotide at position +1 to the Ψ site (A, C or G) while the rest of the 5 bases represented all possible combinations of A,C and G (i.e. 3 groups of 243 sequences each). For each group a coefficient of variance was calculated. The three calculated values were then averaged. To assess the contribution of the 1st two bases downstream to the Ψ site, sequences were grouped into 9 groups each harboring fixed nucleotides at position +1, +2 to the Ψ site (i.e. AA, AC, AG, CA, CC, CG, GA, GC or GG) while the rest of the 4 bases represented all possible combinations of A, C and G (i.e. nine groups of 81 sequences each). Again, nine coefficient of variance values were generated and averaged. The analysis was repeated for fixing the 3, 4 and 5 bases immediately downstream to the Ψ site. Overall, the analysis resulted in a maximal coefficient of variance, and five additional averaged coefficient of variance values calculated for groups in which an increasing number of bases were fixed downstream to the Ψ site.

### snoRNA overexpression

A cassette containing the intron in which each snoRNA resides, and its two flanking exons (For ACA21, RPL23 exons 2–3, for ACA61, SNHG12 exons 4–5) was amplified from genomic DNA using Phusion HF (NEB). The fragment was cloned to pmTurquoise2-H2A plasmid (41) between the BglII and HindIII sites. A mixture of the two snoRNA expression plasmids was transfected in equal amounts to the pool of variants. As a control, an empty pmTurquoise2-H2A plasmid was co-transfected with the pool of variants. ACA21 and ACA61 were chosen as they complied with the following experimental demands: first, they are expressed in WT HEK-293T cells, and are among the 10 H/ACA snoRNAs with the highest rank of expression in ENCODE cell lines (21). Second, their rRNA targets have high Ψ levels as previously measured by Ψ-seq (data not shown). Third, they are the sole targeting snoRNAs of their rRNA targets, ensuring potential Ψ signals are not a result of redundancy in targeting by additional snoRNAs. Fourth, they are not part of a cluster of highly similar snoRNAs, thus do not resemble any other snoRNA by >80% (21). Last, the length of the region encompassing the snoRNAs, the intron they are embedded in and the two flanking exons was <500 nt-long, facilitating the amplification and cloning of the fragment.

### DKC1 knockdown experiment

Human HEK-293T cells were plated in 10cm plates at low confluence (500 000 cells per plate). siRNAs targeting DKC1 (Thermo Fisher, catalog number S4111) were transfected using Lipofectamine RNAiMAX (Life Technologies) following the manufacturer's protocols, with two siRNA boosts at 48 and 96 h following transfection; As negative controls, we used Ambion® In Vivo Negative Control #1 siRNA (catalog number: 4457287). 120 h post the first siRNA transfection, the cells were transfected with the MPRA library (using PolyJet reagent (Signagen Laboratories). The cells were harvested in Nucleozol (Macherey Nagel) 24 h post the library transfection.

### Measurement of pseudouridylation within endogenous rRNA

For quantification of Ψ levels of known sites in endogenous rRNA, total RNA was extracted from three HEK-293T samples and treated with CMC in BEU buffer or only resuspended in BEU buffer (as input control), essentially as in (14), except cleanups following the CMC treatment and sodium bicarbonate incubation were performed on Dynabeads MyOne Silane beads (Thermo Fisher scientific). Library preparation was carried out as in (14) using SuperScript III reverse transcriptase (Thermo Fisher scientific). Corresponding data is presented in Supplementary Table S6, and was used to generate Supplementary Figure S1G.

### Measurement of pseudouridylation within endogenous mRNA

Transcripts from GENCODE V19 were scanned to identify ones with full or partial complementarity to H/ACA box snoRNA arms (using the ‘matchPattern’ command from the Biostrings package in R). Ψ-scores at potential target uridines with a 14, 16, 17 or 18 bp complementarity to the snoRNAs were calculated as described above, utilizing data from Carlile *et al.* (15). In parallel, Ψ-scores from 1000 random uridines were calculated as background. Ψ-scores were calculated only for sites with coverage >90 reads in both CMC-treated and input samples.

### Quantification of snoRNA levels

RNA from the snoRNA overexpression experiment and from the various cell lines was size selected using RNA clean and concentrator-5 kit (Zymo research), following the manufacturer's protocol for purification of RNA under 200nt long. RNA-seq libraries were prepared as in (14).

### Cloning novel snoRNAs to target DMD and CFTR PTC reporter

Replacement of the target binding sequences in ACA61 (8nt 5’ part of the pseudouridylation pocket and 3nt of the 3’ part) with the targeted DMD reporter sequence was carried out using the TPCR method (42) with Phusion enzyme and the following primers: DMD hyb only FW and H2A RV. Replacement of the target binding sequences in ACA61 with the targeted CFTR sequence was carried out using Gibson assembly (Neb). Insert primers were ACA61 CFTR hyb only FW and RV, vector primers were ACA61 gibson vector FW and RV (Supplementary Table S8).

### Quantification of psedouridylation of the PTC in the DMD reporter

500,000 HEK-293T cells were plated in each well of a six wells plate a day before transfection. Cells were transfected using polyJet reagent (Signagen Laboratories) with a 0.5 μg of the reporter and 0.5 μg of the snoRNA (either ACA61 modified to target DMD or WT ACA61). RNA was purified using Nucleozol (Macherey Nagel) one day post-transfection, and duplicates of each sample were fragmented, dephosphorylated and ligated to an RNA adapter, pooled and treated with ribo-zero Gold rRNA Removal Kit (Human/Mouse/Rat) (Illumina). CMC treatment was carried out in a pool.

### Fluorescent PTC readthrough assay in HEK-293T cells

5,000 Cells were plated in each well of a 96-well black flat clear bottom Polystyrene plates (Greiner), a day before transfection. For transfection of the 8- or 9-well replicates of each sample, a pool containing 0.5 μg of the reporter and 0.5 μg of the snoRNA was prepared with polyJet reagent (Signagen Laboratories), according to the manufacturer's manual. As a calibration curve, mixes of the DMD reporter that does not contain a PTC (DMD no stop reporter) with the DMD PTC reporter were prepared, such that the no stop reporter was 0%, 0.5%, 1%, 2%, 4% or 8%. The fluorescence was measured 24 h post-transfection, in PBS, using an Infinite M200 Pro plate reader (Tecan). In order to assess the levels of readthrough based on the calibration curve, the ratio of the two fluorophores measurements of the samples were overlaid on the plot by calculating the intersection point of the average of the 8 measurements of each sample with the calibration line.

### Synthesis of 100% pseudouridylated constructs and Western Blot analysis

The Flag-eGFP reporter mRNAs encoding the DMD and CFTR target sequences were generated as previously described (43). In short, the protocol is based on the ligation of a capped 5’ transcript, encoding a part of the eGFP sequence, to chemically synthesized oligonucleotides that were enzymatically poly(A) tailed. The eGFP sequence served as a reporter construct and did not harbor any fluorescent properties on the protein level. The template for the *in vitro* transcribed 5’ fragment was generated by PCR amplification of a fragment of the eGFP cassette of the lentiviral pHR-DEST-SFFV-eGFP plasmid with an N-terminal Flag-tag introduced through the GFP reporter Flag FW primer (Supplementary Table S8), T7 promoter italicized, start codon in bold, and Flag-tag underlined). In order to generate a defined 3’ end of the RNA fragment we employed a methylated reverse primer, GFP reporter Flag RV (Supplementary Table S8). After transcription, the 5’ transcript was ligated to the chemically synthesized and enzymatically poly(A) tailed oligonucleotides: DMD readthrough control, DMD Ψ-PTC, CFTR readthrough control and CFTR Ψ-PTC (Supplementary Table S8, modified codon in bold and underlined, gene-specific sequence in italic, ligation site indicated with *, the 3’ end of the T7 transcript in bold, (A)n represents the poly(A)-tail). The ligated mRNAs were purified employing an mRNA isolation kit (NEB) and transfected into HEK-293T cells using metafectene (Biontex). Twenty four hours post transfection total protein was isolated, separated on Novex 16% Tris–tricine gels (Thermo Scientific) and blotted on 0.45 μm PVDF membranes (Amersham). The blots were probed with anti-Flag M2 (Sigma) and anti-α-tubulin antibodies (Abcam).

### Mass spectrometry analysis of translation products

The Ψ-PTC mRNAs were compared to uridine containing (non-Ψ) PTC mRNAs (Supplementary Table S8). Flag-eGFP peptides translated in HEK-293T cells were purified with anti-Flag M2 magnetic beads (Sigma). Pulled down proteins were extensively washed with 50 mM ammonium acetate and were directly digested on the beads adding Trypsin and alkylated with iodoacetamide (55 mM). Peptides were analyzed using a Dionex, UltiMate 3000 nano-HPLC system (Germering, Germany) coupled via nanospray ionization source to a Thermo Scientific Q Exactive HF mass spectrometer (Vienna, Austria) using instrument settings as described previously (44). The database search was performed using ProteomeDiscover (Version 2.1, Thermo Scientific). Sensitivity of the assay was calculated by the median abundance of the 10 least abundant peptides in the least sensitive sample, out of the total abundance of all of the C-terminal peptides in any of the samples.

### Luciferase stop codon readthrough assay

Plasmids were generated by inserting the coding sequence (CDS) of Renilla luciferase downstream from the CDS of Firefly luciferase for which the stop codon was removed, thus creating a template for expression of a fusion protein with dual luciferase activity. Using site-directed mutagenesis, three stop codons were inserted individually into the constructs, between the two CDSs. Thus, four plasmid constructs were generated in which Firefly luciferase/Renilla luciferase encoded either a fusion product or bi-cistronic products interrupted by three different stop codons.

The mRNAs were transcribed as previously described (45), using the 4 different linearized plasmid templates encoding Firefly/Renilla luciferases with or without inserted stop codons, adding T7 RNA polymerase (Megascript, Ambion) and either UTP or ΨTP, to generate 8 mRNAs containing only U or Ψ in all of the encoded positions. The transcribed mRNAs contained 101 nt-long poly(A) tails. All RNAs were capped using the m7G capping kit with 2’-*O*-methyltransferase (ScriptCap, CellScript) to obtain cap1. mRNAs were HPLC purified as described (45).

HEK-293T cells were seeded (50,000 cells/well) into 96-well plate a day prior to transfection with 0.3 μg/well of the bi-cistronic mRNA constructs formulated with TransIT mRNA reagent (Mirus Bio) as described (45). Following 24 h incubation, the medium was removed and the cells were lysed in 1× dual-luciferase lysis buffer (Promega). Aliquots were assayed for Firefly and Renilla luciferase enzyme activities using the dual-luciferase reporter assay system (Promega) in LUMAT LB 950 luminometer (Berthold/EG&G; Wallac).

For the experiments described in the supplementary material, several changes were made in the experimental approach: in one experiment, non-purified mRNA was transfected to HEK-293T cells. In another experiment, HEK-293T cells were transfected with mRNA in-vitro transcribed with either 100% 1-methylpseudouridine or uridine, which was HPLC-purified. In addition, constructs in which the order of the luciferases is reversed were cloned similarly. Non-purified in-vitro transcribed mRNAs from these constructs were either transfected to HEK-293T cells or in-vitro translated in rabbit reticulocyte lysate (RRL).

### Expression analysis of DKC1 and snoRNAs

To estimate expression of DKC1 and snoRNAs in siDKC1 and control HEK-293T samples, reads were aligned against the human genome using RSEM (version 1.2.31) in paired-end and strand-specific mode with default parameters (46). To estimate expression of snoRNAs in the experiment comparing their abundance across cell lines (HEK-293T, A549, K562 and MCF7) and in cells overexpressing ACA21 and ACA61 compared to control plasmids VERSE was used in stranded HTSeq-Intersection_strict mode (https://github.com/qinzhu/VERSE). For robust comparison between different samples, we used trimmed mean of M values (TMM) normalization (47) of the RSEM and VERSE read counts as implemented by the NOISeq package (48) in R. For differential analysis of snoRNA expression between cell lines, a pseudocount of 1 was added to TMM-normalized expression values to stabilize the ratio and avoid division by 0. Binning snoRNAs into three distinct bins according to expression was done using the ‘cut2’ function from the ‘base’ package in R (R Core Team (2013), R: A Language and Environment for Statistical Computing), with ‘number of quantile groups’ set to 3 (Figure 4B, C, Supplementary Figure S2C, D).

### Statistical tests

**Figure 2.**
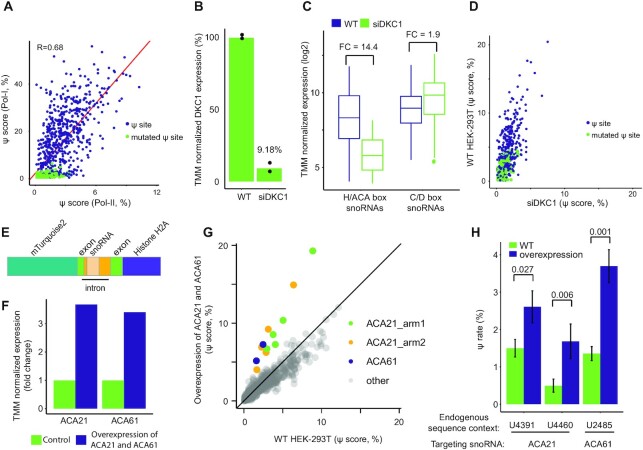
Ψ signals in the MPRA system are DKC1 and snoRNA dependent. (**A**) Pearson's correlation between Ψ-score of constructs from CMC-treated HEK-293T samples transfected with Pol-I- or Pol-II-promoter libraries. Colors as in Figure 1D, E. *N* = 2 for each promoter library. (B–D) HEK-293T cells were transfected with Pol-II-promoter libraries and with siRNA targeting DKC1 or control. *N* = 2. (**B**) Normalized RNA level of DKC1. (**C**) Normalized RNA level of H/ACA box and C/D box snoRNAs. Boxplot parameters as in Figure 1G. FC: fold change. (**D**) Correlation between the Ψ-score of constructs from CMC-treated HEK-293T, colored as in Figure 1E. (**E**) Scheme of the snoRNA overexpression construct. (**F**) Normalized RNA level of ACA21 and ACA61 in WT HEK-293T cells (green) and in cells overexpressing the two snoRNAs (purple). *N* = 1. (**G**) Correlation between Ψ-score of synthetic snoRNA target constructs from CMC-treated HEK-293T samples transfected with Pol-II-promoter libraries and with plasmids overexpressing ACA21 and ACA61 or a mock plasmid (*n* = 1 and 2, respectively). Colors indicate whether a construct is targeted by ACA21 (green and yellow), by ACA61 (purple) or by another snoRNA (grey). (**H**) Ψ-scores of constructs representing native sites from 28S, which are targeted by ACA21 and ACA61, as quantified from the samples in (G). Error bars represent standard error of the binomial distribution of the mean Ψ-score. *P* values of generalized linear mixed models are presented.

All statistical tests were conducted using built in packages in R as described (R Core Team (2013), R: A Language and Environment for Statistical Computing), except for Generalized Linear Mixed Model (GLMM), which utilized the lme4 package (49). For calculation of the standard error of the binomial distribution of the mean Ψ-score (Figure 2H and 5B) the following equation was used:

– number of reads stopping at the inquired position
– coverage of the inquired position
- standard error = ((S/C*(1 – S/C))/C)^0.5^

## RESULTS

### Massively Parallel Reporter Assay based method for quantification of Ψ in snoRNA-target sequences

To systematically explore the potential for snoRNA-mediated pseudouridylation of mRNA targets, we developed a highly sensitive set of reporters allowing direct and systematic interrogation of such activity. This approach relies on synthesis, cloning and expression within cells of hundreds of sequence variants, each of which is designed with a predefined level of sequence complementarity to targeting arms of human H/ACA box snoRNAs. Specifically, we designed two series of synthetic snoRNA target sequences as part of a Massively Parallel Reporter Assay (MPRA) (Figure 1A). The first series was designed to systematically explore the length of complementarity stretches required for formation of Ψ in mRNA. For each of the 68 snoRNAs in this series (with a total of 99 targeting arms), we designed four sequence variants, each including a target uridine followed by a guanosine, flanked on both sides by up to 3, 5, 8 or 10 nt-long sequences complementary to the snoRNA (see Materials and Methods). The second series, implemented on a subset of 30 H/ACA snoRNAs, was designed to explore the impact of the nucleotide downstream from the pseudouridylated target site. This position is typically not involved in base-pairing with the snoRNA, and hence in principle is under no sequence or structural constraints. In this series, the snoRNA complementarity stretch was kept constant and maximal (10 nt on each side), but the position immediately following the target uridine site was systematically perturbed to include each of the four nucleosides or none at all (Figure 1A, nucleosides in blue in upper part). As a control, for each snoRNA, an additional variant with 10 nt-long complementarity was generated in which the modified uridine was mutated into a guanosine. This set of 606 synthetic snoRNA target sequences enabled us to assess whether mRNA-like transcripts can undergo snoRNA-dependent pseudouridylation and to explore the parameters affecting the extent of the modification. In addition, a set of 108 variants, representing native Ψ sites in the human rRNA 18S, 28S and 5.8S and in snRNAs, was designed by positioning each known Ψ target-site in position 42 within a 75 nt-long stretch of the native rRNA and snRNA transcripts (Figure 1A, bottom). For each native site a point mutant variant was generated by replacing the modified uridine by a guanosine.

Each of the 822 sequences in these series further comprised a unique 8 nt-long barcode and common adapters on both ends (Supplementary Table S1). These pooled sequences were cloned into two distinct plasmids (Materials & Methods) allowing expression of the constructs under RNA Pol-I- and RNA Pol-II-promoters, which naturally direct transcription of rRNA and mRNA, respectively. The former promoter, which drives rRNA transcription and results in nucleolar localization (50,51), serves as a positive control, whereas the Pol-II promoter allows assessing the extent to which pseudouridylation can occur on mRNAs.

To facilitate adequate sequence coverage for each of the sequence variants, we utilized targeted Ψ-seq, which relies on construct-specific priming of reverse-transcription ((17), Materials and Methods) and provided readouts of Ψ levels exclusively within the constructs of CMC-treated samples (Figure 1B). Using this strategy, a coverage of >200 reads for 97% of the constructs was obtained, even at a relatively shallow sequencing depth (∼0.7–2.6M reads per sample). Utilizing a dedicated analytical pipeline, a Ψ-score was calculated for each position, capturing the extent to which reverse transcriptase terminates at a Ψ harboring site (Figure 1C and Supplementary Table S2).

Transfection of pooled reporter libraries into HEK-293T cells revealed the expected signal at the synthetic and native target sites. Relatively high signal was observed for constructs transcribed from Pol-I promoters, known to result in nucleolar localization of the RNA (50,51), whereas a substantially lower—and yet highly significant—signal was observed for those transcribed from constructs driven by Pol-II promoters (median 17.4% and 2.4%, respectively). In both cases the signal was highly significant in comparison to non-CMC treated (‘Input’) samples (paired *t*-test *P* = 2.04e–149 and 9.36e–162 for in Pol-I and Pol-II, respectively) (Figure 1D, E) and in comparison to control constructs in which the target Ψ site was mutated (paired *t*-test *P* = 6e–118 and 6e–114 for Pol-I and Pol-II, respectively; Figure 1F, G). We further found that our measurements were highly reproducible across biological replicates (Pearson's *R* = 0.97 and 0.84 for Pol-I and Pol-II-driven libraries, respectively; Figure 1H, I and Supplementary Figure S1A).

On the basis of these libraries, in the analyses below we aimed to perform two sets of comparisons: (i) of pseudouridine levels within the same constructs under different conditions/perturbations, (ii) between constructs aimed at examining varying complementation lengths between the snoRNA and its targets, possessing related, yet distinct sequences in which the nucleotides immediately adjacent to the pseudouridylated site were kept constant but more remote ones were varied. Given that Ψ-seq is not quantitative in an absolute sense (14), and quantifications can be impacted by sequence-dependent differences in the formation of Ψ-CMC adducts and/or sequence-dependent variability of RT-termination rates at such adducts, we sought to systematically assess the extent to which Ψ-seq could allow inferences along both dimensions. With these two goals in mind, we synthesized 729 DNA reporter sequences such that each harbored a single thymidine followed by a stretch of six varying bases, representing all possible combinations of adenine, cytidine and guanine (Figure 1J). The reporters were used as a template for in vitro transcription, which was conducted with either pseudouridine or uridine, and were then mixed in ratios ranging from 100% uridine to 100% pseudouridine (see Materials and Methods). We then acquired pseudouridine measurements across the distinct sequences and pseudouridine stoichiometries. An examination of the increase in signal as a function of increased stoichiometry of pseudouridylation revealed that the overwhelming majority of pseudouridylation targets displayed a clear linear increase, with R^2 of fitted slopes ranging between 0.91 and 0.99 (median of Pearson's correlation = 0.99; Figure 1K, Supplementary Figure S1B and Supplementary Table S3), demonstrating the utility of this approach for relative comparisons of the same sites. Moreover, even an examination of the distribution of Ψ-scores (stemming from distinct sequences) as measured across distinct stoichiometries revealed clear distinction between sets of sites modified at varying levels, supporting the ability to compare signals also across different sequences (Figure 1K). Nonetheless, this analysis also revealed variability in the extent of signal obtained between individual sequences modified at identical ratios. For example, the Ψ-scores of sites with 100% pseudouridine ranged from 23–39% (10th to 90th percentile) and reached extremes as low as 6% (minimal value) to as high as 52% (maximal value). We suspected that this variability could, in part, be attributed to sequence-context of the different sites. To explore this, we binned sites into groups based on the number of shared identical nucleotides immediately adjacent to the pseudouridylated target. This analysis revealed that the extent of variability in pseudouridylation quantifications consistently decreased as the extent of shared sequence increased. While the overall coefficient of variation across all sequences was ∼20%, the average coefficient of variation among sites sharing 3 bases was reduced to 16.4% and ones sharing 5 bases dropped to 12.5% (Figure 1L). Collectively, these analyses reinforce the validity of comparing relative levels of pseudouridylation at the same site, and furthermore support the validity of comparisons between different sequences, in particular when (i) they share sequence identity immediately flanking the pseudouridylation target, and when (ii) such analyses are conducted between groups of sequences, in which case summarizing metrics of the distribution (e.g. median) are robust to extreme outliers of individual measurements. These insights guided the analyses below.

### Ψ signals in the MPRA system are DKC1- and snoRNA-dependent

Although mRNAs from Pol-I-driven constructs exhibited higher Ψ-scores compared to Pol-II-driven constructs, overall we observed a good agreement between Ψ-scores at corresponding sites transcribed under the two different promoters (Figure 2A; Pearson's *R* = 0.68). As nucleolar pseudouridylation of rRNA transcripts is catalyzed by the H/ACA snoRNP complex (9–11), we sought to verify that the Ψ signals of our mRNA-like transcripts are dependent on the same machinery, by manipulating the levels of DKC1, the catalytic component of the complex (52), and of selected snoRNAs, that guide DKC1 to its targets. Thus, we first knocked-down DKC1 in HEK-293T by transfecting cells with siRNAs against DKC1 or a control sequence. The knock-down, which resulted in a marked decrease in DKC1 mRNA abundance (Figure 2B and Supplementary Table S4; 9.18% of the abundance in control transfected cells) was accompanied by a significant global decrease of >14-fold in the mean abundance of H/ACA box snoRNAs (Figure 2C and Supplementary Table S4; paired *t*-test *P* = 5.25e–10), as was reported earlier (52), whereas only a subtle impact was observed on the abundance of C/D box snoRNAs (Figure 2C and Supplementary Table S4; <2-fold difference between mean abundance, paired *t*-test *P* = 1.14e–13). Critically, DKC1 knock-down resulted in a significant decrease in Ψ-scores of native rRNA sites and high-complementarity synthetic targets (8–10 nt complementarity; purple dots in Figure 2D) expressed in HEK-293T cells under the Pol-II promoter, indicating that DKC1 is required for their modification (Figure 2D, median Ψ-scores of 3.4% and 1.3% in WT and siDKC1, respectively; paired *t*-test *P* = 2.21e–59).

Next, we utilized a plasmid based overexpression system to co-overexpress two snoRNAs, ACA21 and ACA61 (see Materials and Methods for considerations underlying their selection), in HEK-293T cells (Figure 2E). Both snoRNAs were individually cloned into the pmTurquoise2-H2A plasmid and co-transfected into HEK-293T cells, resulting in a >3-fold increase in their RNA abundance (Figure 2E, F and Supplementary Table S5). Overexpression of ACA21 and ACA61 snoRNAs, resulted in a specific elevation of Ψ-scores in synthetic targets with high complementarity to each of the snoRNAs (Figure 2G), while no change was observed in Ψ-score of target sites lacking complementarity to ACA21 or ACA61 (Figure 2G). Concordantly, Ψ-score of native rRNA constructs of ACA21 and ACA61, were also elevated upon the dual overexpression (Figure 2H, generalized linear mixed models *P* = 0.001, 0.027 and 0.006 for sites 28S:2485, 28S:4391 and 28S:4460, respectively). Overall, these results indicate that the moderate levels of pseudouridylation seen in Pol-II-driven mRNA-like transcripts is dependent on the H/ACA snoRNP complex. Moreover, these results suggest that the limited expression levels of snoRNAs are among the bottlenecks constraining snoRNA-mediated pseudouridylation of mRNA.

### Features modulating Ψ level in mRNA-like snoRNA targets

**Figure 3.**
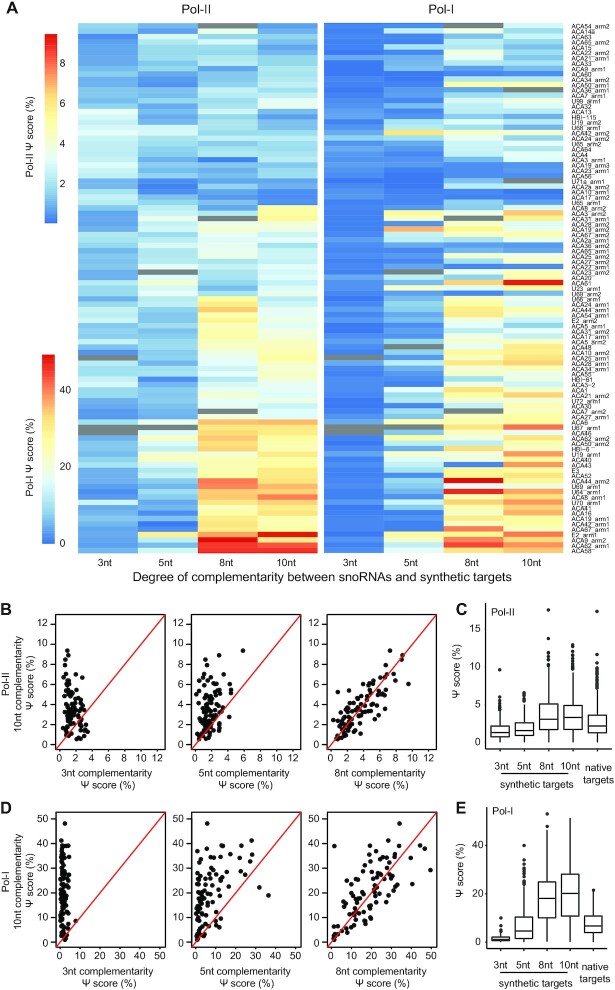
Ψ level in snoRNA targets is modulated by the degree of complementarity between the snoRNA and its target. (**A**) Heatmap of Ψ-score of synthetic snoRNA targets with increasing complementarity to snoRNAs as measured in HEK-293T cells transfected with Pol-II- or Pol-I-promoter libraries (left and right, respectively). Each row represents constructs targeted by a specific snoRNA (listed on the right). Columns represent the degree of complementarity of the synthetic target to its specific snoRNA. Hierarchical clustering was conducted according to the signals in Pol-II reporters, and reporters in Pol-I were ordered accordingly. *N* = 2. Missing values are shown in grey. (**B**, **C**) Ψ-score values of synthetic snoRNA constructs with increasing complementarity to snoRNAs (B, C) and WT rRNA and snRNA target constructs (C, right group in boxplot) as measured in HEK-293T cells transfected with Pol-II-promoter libraries (*n* = 6). (**D, E**) Ψ-score values of synthetic snoRNA constructs with increasing complementarity to snoRNAs (D, E) and WT rRNA and snRNA target constructs (E, right group in boxplot) as measured in HEK-293T cells transfected with Pol-I-promoter libraries (*n* = 2).

**Figure 4.**
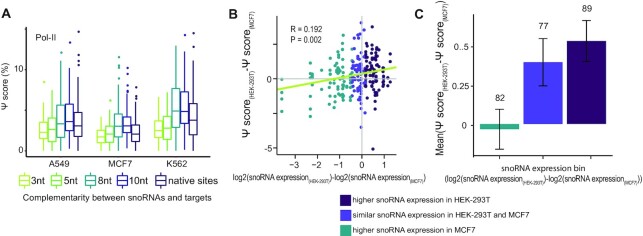
Ψ level in snoRNA targets is correlated to the snoRNA abundance. (**A**) Ψ-score values of synthetic snoRNA constructs with increasing complementarity to snoRNAs and WT rRNA and snRNA target constructs as measured in A549, MCF7 and K562 cells transfected with Pol-II-promoter libraries (*n* = 1). (**B**) Spearman's correlation between the difference in abundance of snoRNAs in HEK-293T and MCF7 cells and the difference in Ψ-score of targets of these snoRNAs in the two cell lines. Analysis includes constructs of native 18S and 28S target sites and synthetic constructs with high complementarity (10nt and 8nt) to the targeting snoRNA. Colors indicate division into three similarly sized bins according to differential expression in the two cell lines. Regression line in green. *N* = 1. R, P: Spearman correlation and p value, respectively. (**C**) Data from (B) is represented as a barplot displaying the differential Ψ-score between HEK-293T and MCF7 cells as a function of snoRNA differential expression bin (mean ± standard error; *n* = 1). Number at the top of each bar indicates the number of constructs in that bin.

We next utilized our MPRA libraries to systematically explore two key features within the target sequences impacting efficiencies of mRNA pseudouridylation. We first examined the impact of the length of complementarity between a given snoRNA and its synthetic target. As shown in the heatmap in Figure 3A (left panel), Ψ-scores in RNAs transcribed from the Pol-II-driven constructs progressively increase as the complementarity between the target mRNA and snoRNA increases from 3 to 10 nts on each side of the target site (Figure 3B, C; median of Ψ-score in 3–5 nt and 8–10 nt complementarity constructs = 1.4% and 3.1%, respectively). The same trend was seen for sequences transcribed from the Pol-I-driven constructs, but with an increased effect size and dynamic range (Figure 3A (right panel), and Figure 3D,E; median of Ψ-score in 3–5 nt and 8–10 nt complementarity constructs = 2% and 18.9%, respectively). Interestingly, the Ψ-score of constructs containing native rRNA and snRNA target sites in both promoter contexts displays an overall lower signal than in synthetic constructs of 8–10 nt complementarity (median of rRNA sites = 2.2% and 6.7% in Pol-II- and Pol-I-driven libraries, respectively), consistent with the decreased length of complementarity in natural targets. This suggests that increasing the length of complementarity between a snoRNA and its mRNA target beyond those naturally occurring at rRNA targets facilitates higher modification levels of mRNA (Figure 3C, E). Notably, transfecting the Pol-II-driven library into three additional human cell lines (A549, K562 and MCF7) recapitulated the finding that the measured Ψ-score increases as the length of complementarity between the snoRNA and its targets increases, suggesting that this phenomenon is not cell-type specific (Figure 4A).

As indicated above, the Ψ-score cannot be interpreted as an absolute metric of pseudouridine levels. Nonetheless, examination of metrics summarizing the overall distribution of values can provide a qualitative sense of the relative efficiency of pseudouridylation. Specifically, median Ψ-scores within the endogenous rRNA targets (measured by applying Ψ-seq to total RNA), were ∼16% (Supplementary Table S6). In a parallel-CMC-treated targeted sequencing library, median levels at the same sequence contexts, but when installed into Pol-I driven libraries which lack the full context and structure of the ribosome was only 6.7%, suggesting that within these more limited contexts pseudouridylation occurs at an efficiency of roughly 40% of maximal levels; However, these levels could be increased to 19%, i.e. similar to maximal levels, using extended sequence complementarity of 8–10 bases. When driven from a Pol-II promoter, even with the 8–10 nt long target constructs, we could achieve median levels of 3.1%. These levels correspond to roughly a sixth of the pseudouridylation level of their potentially stoichiometrically modified Pol-I counterparts, and hence are suggestive of median pseudouridylation levels roughly at the order of 16%. Our results thus demonstrate that pseudouridylation can be directed at mRNA-like targets, but that even the most optimally designed targets are typically modified at substantially lower efficiency (stoichiometries) than their Pol-I counterparts or endogenous rRNA targets.

A second feature we examined was the identity of the non-paired nucleotide immediately downstream to the modified uridine. Quantifying the Ψ-score of constructs with distinct nucleotides downstream to the modified site revealed that removing the non-paired nucleotide causes the most dramatic reduction in Ψ-score (Supplementary Figure S1C–F), presumably because it introduces an offset in the complementarity and abolishes base-pairing interactions between all positions downstream of the target site and their cognate positions on the snoRNA. We also found that presence of uridine immediately downstream of the modified site typically resulted in higher Ψ-scores (Supplementary Figure S1C–F). However, this observation likely reflects a technical sequencing-dependent bias in CMC-based profiling of Ψ, rather than a biological effect, given that a similar effect was apparent when we analyzed Ψ-scores at endogenous targets on the human rRNA, the majority of which are modified at stoichiometric levels(53) (Supplementary Figure S1G, Supplementary Table S6).

Although most constructs responded similarly in the different cell lines (Figure 3C, Figure 4A, Supplementary Figure S1D,F and Supplementary Figure S2A), some targets had significantly higher Ψ-scores in one cell line compared to others. This is in line with recent evidence for cell specific modification levels of a subset of Ψ-sites in human rRNA (54). We hypothesized that differences in snoRNA levels between the different cell lines may partially underlie the differences in Ψ-scores. To directly explore this, we acquired snoRNA expression levels and Ψ-scores for each of our target sites in four different cell lines, and assessed their relationship (Supplementary Table S7). Although snoRNA levels generally correlated well with each other across cell lines (Supplementary Figure S2B), plotting the *difference* in Ψ-score as a function of the *difference* in snoRNA abundance revealed a moderate but consistent positive correlation (Figure 4B, C, Supplementary Figure S2C, D), suggesting that higher levels of a snoRNA lead, to some extent, to higher Ψ levels of its target. Thus, differences in snoRNA levels between cell lines can result in differential pseudouridylation of mRNA targets.

Our results thus suggest that mRNA pseudouridylation can, in principle, occur on mRNA targets. We further find that such pseudouridylation is constrained at three levels: First, the promoter driving mRNA transcription is suboptimal, presumably as Pol-II transcripts are not directed to the nucleolus in which the snoRNA machinery is particularly abundant; Second, physiologically occurring levels of snoRNAs are suboptimal for achieving efficient mRNA pseudouridylation; Third, the length of the complementarity stretch required for achieving efficient mRNA targeting is extended compared to that of the rRNA.

### No evidence for significant ribosomal readthrough caused by Ψ in stop-codons in human cells

**Figure 5.**
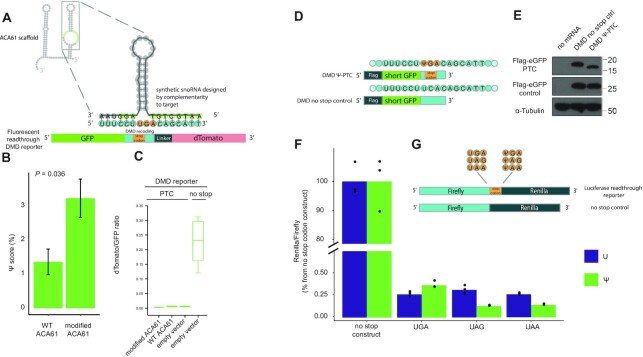
Ψ in stop codons does not result in significant stop codon readthrough in HEK-293T cells. (**A**) Design of a synthetic snoRNA to target a premature termination codon (PTC) causing Duchenne muscular dystrophy (DMD). The target for pseudouridylation was a bicistronic fluorescent readthrough reporter, in which the disease-related PTC, its surrounding sequence and a linker separate the two fluorophores. The sequence of ACA61 was used as a scaffold for the novel snoRNA, which differed in the nucleotides complementary to the target (complementary to the surroundings of the uridine at the PTC, instead of to the endogenous target). (**B**) Ψ-score of the uridine in the stop codon of the DMD reporter was quantified in HEK-293T cells co-expressing the reporter with either the modified ACA61 or WT ACA61 not targeting the reporter as a control. Generalized linear mixed models *P* = 0.036. (**C**) The ratio between the fluorophore downstream to the stop codon (dTomato) to the one preceding it (GFP) was quantified using a fluorescent plate reader as a measurement of the readthrough of the stop codons in the reporter. (**D**) Constructs for quantification of readthrough of a 100% pseudouridylated stop codon. The same sequence context of the DMD PTC was synthesized with a Ψ residue (top) or a no stop control (bottom), and ligated downstream of a sequence encoding a short GFP ORF tagged with a C-terminal FLAG tag. (**E**) Proteins were purified from HEK-293T cells transfected with the synthetic DMD RNAs and a longer control RNA, also tagged with FLAG, and subjected to Western blot analysis. Top panel: Flag antibody, product of the synthetic reporters. Middle panel: Flag antibody, transfection control. Bottom panel: Tubulin antibody, loading control. (**F**) Dual luciferase assay measuring in HEK-293T cells the readthrough of the various stop codons present in the transfected HPLC-purified mRNAs generated to contain either uridine or Ψ. (**G**) Constructs used in the dual luciferase assay. RNA encoding Firefly luciferase, followed by either of the stop codons or none, and Renilla luciferase was in vitro transcribed to contain either uridine or Ψ.

Given the ability of snoRNAs to act on mRNA, we next wondered whether this activity could be harnessed for therapeutic purposes. Specifically, it has been previously reported that the presence of Ψ in stop codons can result in read-through (31,55) with 70% read-through observed in an in-vitro translation assay (31). Considering that a wide array of genetic diseases are caused by mutations giving rise to premature stop codons, we sought to explore the potential of using snoRNA mediated pseudouridylation of predefined termination codons to achieve readthrough into downstream regions. We concentrated on the commonly caused premature termination codon (PTC) in dystrophin (DMD) mRNA, causing Duchenne muscular dystrophy. We employed a bicistronic fluorescent readthrough reporter, in which the two fluorophores are separated by the sequence element preceding and immediately following the S319X mutation in the dystrophin gene, giving rise to a premature UGA codon (56). We then designed a synthetic snoRNA based on the scaffold of the ACA61 snoRNA, replacing the sequence complementary to the native target with a sequence complementary to the surroundings of the uridine at the DMD PTC, to target it for pseudouridylation (Figure 5A). As a synthetic snoRNA, in which a 10nt stretch complementary to each side of the target PTC was changed, did not express well (presumably due to a damage to the sequences needed for the maturation of the snoRNA, data not shown), we only altered the nucleotides that are predicted to be involved in target binding. We co-expressed this synthetic snoRNA and the corresponding reporter in HEK-293T cells, purified RNA and quantified the Ψ level of the site (Figure 5B). We observed a low but statistically significant Ψ-score of 3%, compared to background levels of ∼1% when a WT ACA61 was expressed alongside the reporter as a control, confirming the pseudouridylation of the target site. We then examined potential read through of the PTC, by quantification of the fluorescence of the fluorophore following the PTC (dTomato) relative to that of the preceding one (GFP) using a plate reader. We failed to observe evidence for selective readthrough of the pseudouridylated target in the DMD reporter (Figure 5C, Supplementary Figure S3D, E). To assess the sensitivity of the plate reader measurement we expressed different mixtures of the reporter lacking a stop codon (‘no stop’ reporter) with the PTC reporter. The readthrough levels observed with the DMD targeting ACA61 snoRNA were in the range of the control lacking the no stop reporter altogether (Supplementary Figure S3A).

To rule out the possibility that low-level pseudouridylation of the target site precluded us from observing an effect, we turned to measurement of readthrough in a construct that was 100% pseudouridylated. To this end we chemically synthesized the target sequence surrounding the DMD PTC with fully pseudouridylated stop codons, downstream of a sequence encoding a FLAG tag and a part of the gfp gene. As a positive control, we also synthesized the same RNA construct with the wild-type codon, which should lead to 100% full size product (Figure 5D). We transfected these mRNAs to HEK-293T cells, purified proteins, separated them through high-resolution tricine–SDS-PAGE and conducted a western-blot analysis (Figure 5E). We observed no evidence for full-length protein translation from the pseudouridylated PTC construct. To allow detection of potential rare readthrough products, we conducted LC–MS/MS analysis of the Ψ-PTC construct compared to the corresponding U-PTC and searched for C-terminal peptides that should be present if translation is continued beyond the stop codon. We were unable to detect any peptide indicative of readthrough (sensitivity of the assay ranged between 0.08% and 0.9%, data not shown). Of note, in this system the PTC-containing reporter RNAs are directly transfected into cells, and hence are not subject to nonsense-mediated decay (NMD). A similar synthetic snoRNA was designed for a reporter containing another disease-related PTC-creating mutation, the Y122X mutation in the cystic fibrosis transmembrane conductance regulator (CFTR) gene, giving rise to a premature UAA codon (Supplementary Figure S3B). When the CFTR reporter and targeting snoRNA were used, a similar effect was observed in the fluorescent bicistronic reporter assay performed in a plate reader (Supplementary Figure S3C, F, G). The CFTR PTC was also employed in the 100% pseudouridylated assay (Supplementary Figure S3H), leading to a similar difficulty to observe readthrough products in the WB assay (Supplementary Figure S3I), and in the LC-MS/MS assay a very low percentage (0.2–0.3%) of readthrough peptides were observed, in which the stop codon was replaced with a sequence of the following amino acids: QLGIGK (Supplementary Figure S3J). Interestingly, we also observed that 2–3% of the peptides contained an addition of a single asparagine following the stop codon, suggesting that the Ψ was skipped by the translation system, resulting in a frameshift creating an AAC codon followed by a stop codon.

To rule out that the inability to observe readthrough of PTC in HEK-293T cells was limited to the set of PTC used in our assay, we designed a set of four dual-luciferase reporter constructs, each of which encoding for two in-frame luciferases (Firefly and Renilla) either as a direct fusion protein (no stop positive control) or separated by each of the three stop codons (UAA, UAG and UGA). Each of these reporters was in vitro transcribed using either 100% uridines or Ψs, following which the resultant RNA was stringently purified. The mRNAs were transfected to HEK-293T cells, and 24 h post-transfection cells were lysed for the luciferase assay. Despite the introduction of 100% Ψs in the stop codon, only negligible levels of readthrough (<0.5%) were observed across all three constructs, in addition to negligible differences in readthrough between the uridine- and Ψ-harboring stop codons (Figure 5F,G). Similar results were obtained when unpurified mRNAs were transfected (Supplementary Figure S3K), when the mRNAs were made to contain 100% 1-methylpseudouridine (Supplementary Figure S3L), when the order of the coding sequences were changed to Renilla/Firefly luciferase ((Supplementary Figure S3M), or when the mRNAs were translated in rabbit reticulocyte lysates (Supplementary Figure S3N)). These lines of evidence thus consistently fail to demonstrate significant levels of Ψ-induced readthrough of stop codons of mRNA in HEK-293T cells.

## DISCUSSION

Here we systematically explore the potential for snoRNA-mediated pseudouridylation of mRNA, the ‘design rules’ of snoRNA targeting, and the consequences thereof. We find that endogenously present H/ACA box snoRNAs can guide pseudouridylation of mRNA. This typically results in relatively low levels of pseudouridylation, due to three constraints that we identify, involving the promoter from which the RNA targets are transcribed, the expression levels of the snoRNA and the length of the complementarity between the snoRNA and the target mRNA. These three observations collectively suggest that a key constraint on mRNA pseudouridylation is the local effective concentration of snoRNAs in the vicinity of the targeted RNA. Whereas Pol-I transcription results in localization of the RNA to the nucleolus (50,51) where local concentrations of snoRNAs are high, Pol-II transcription does not result in such localization, which can be compensated by overexpressing the snoRNA or by increasing the affinity between the snoRNA and the mRNA.

Our work has implications on the targeting scope of snoRNAs, suggesting that mRNAs can serve as snoRNA targets, in particular mRNA-targets with an extended complementarity to the snoRNA and under conditions in which the snoRNA is expressed at high levels. Thus, the *potential* for snoRNA-mediated targeting of mRNA exists. In practice, however, there is a very limited repertoire of mRNA sites that have such extended complementarity stretches as in our reporters. Specifically, scanning the entire transcriptome we were unable to find a single target with either 8 or 10 bp of full complementarity (from both sides of the targeted uridine) to any of the 99 snoRNA arms that we analyzed (Supplementary Figure S4A). However, we found 163 813 potential sites which have complementarity stretches of 14–18 bases within the 20 bases surrounding the potential Ψ site (Supplementary Figure S4B). Quantification of the Ψ level at a subset of these sites which had sufficient coverage (>90 reads) in published Pseudo-seq data from HeLa cells (15) indicates that putative targets with the longest complementarity stretch (18 bp) had the highest distribution of CMC-induced RT termination (Supplementary Figure S4C), with a mean Ψ score of 3.46 (versus 1.81 in the remaining bins). However, this trend was not significant, which may in part also be due to the low number of sites that could be quantified (Supplementary Figure S4B). Overall these findings suggest that a subset of mRNA targets may undergo pseudouridylation via snoRNAs, but for the most part probably at low efficiencies.

Using diverse cell lines with relatively subtle differences in expression levels of snoRNAs, we were able to observe a correlation between differences in abundances in snoRNA level and mRNA pseudouridylation (Figure 4). Our findings have bearings on diverse contexts in which snoRNA levels were found to be dramatically modulated, including cancer, genetic neurodegenerative diseases such as Prader-Willi syndrome (PWS) and viral infection (57–59), and which may result in condition-specific snoRNA guided pseudouridylation of mRNA. Unraveling such targets imposes a major challenge for the future. Despite the availability of transcriptome-wide approaches for identifying such targets (14–16), the relatively low extent of signal that we anticipate to be present at such sites based on this study renders their unbiased identification extremely challenging.

In the three contexts explored in this manuscript, we do not find evidence for appreciable levels of Ψ-mediated readthrough (Figure 5). In a previous study, readthrough levels of 70% were observed in a rabbit reticulocyte lysate in-vitro translation system (31), and similarly high levels of readthrough were observed in bacterial cell lysates (32). Considerably lower levels of 5%-10% readthrough were reported in yeast (31,60), but these levels were thought to reflect the low pseudouridylation efficiencies (measured, in one case, to reach roughly 5% (31)) that could be achieved in these cells using synthetic snoRNAs. These results thus hinted that in yeast, too, pseudouridine-induced readthrough efficiencies are high. In our experiments using mRNA, in which the stop codon was modified either partially via synthetic snoRNA-mediated pseudouridylation or fully via *in vitro* transcription or chemical synthesis, we were unable to find any evidence for readthrough in levels higher than a fraction of a percent, using an array of sensitive detection methods including measurement of luciferase activity of fusion reporter constructs, western blots, or mass-spectrometry. One possibility for reconciling these diverging results is that readthrough is dependent on local sequence context, although a recent report presented evidence for readthrough being generally independent of local sequence context (33). A second possibility is that read-through is organism or context specific, and that it does not occur in significant levels in human cells. Indeed, to date, readthrough was reported to occur in-vivo in significant levels only in yeast cells. The only evidence for significant stop-codon readthrough in mammalian systems is based on *in-vitro* experiments in rabbit-reticulocyte lysates (RRL) (31). However, translational profiles in RRLs can be highly sensitive to experimental parameters, as shown in recent experiments in which Ψ-dependent translational arrest was either observed in RRLs or not, dependent on whether or not rough endoplasmic reticulum (ER)-derived microsomal vesicles were added to the system (61). Hence, results in RRLs need to be confirmed in-vivo. It is further noteworthy that we failed to observe evidence for significant levels of readthrough in our dual luciferase assays conducted in RRLs (Supplementary Figure S3N). Our failure to observe significant levels of readthrough at pseudouridylated stop codons is consistent with (i) a recent study that found no evidence for readthrough in a fully pseudouridine-substituted luciferase reporter subjected to an E. coli based in vitro translation system (62), (ii) with findings that recognition of a pseudouridine-modified stop codon by release factors does not differ from that of a canonical stop codon (62,63) and (iii) with a failure to incorporate Serine on ΨAA in the absence of release factors in an in-vitro assay (62) - such incorporation had been originally proposed as a mechanism for readthrough (31).

Nonetheless, negative results must be interpreted with caution, and it is possible that pseudouridine-induced readthrough requires a specific biological regime. It should further be highlighted that two of our three sets of experiments rely on unnatural, intron-lacking reporters, which are synthesized outside of the cell and transfected and expressed directly within the cytoplasm. One of the reporters (Figure 5D, E, Supplementary Figure S3H–J) is also unnaturally short. It is possible that pseudouridylation-mediated readthrough requires a typical mRNA structure and/or that readthrough is linked to the natural life-cycle of an mRNA, and hence cannot be observed in such reporters. Yet, it should be noted that such reporters have previously been used in cell lysates, where readthrough has been observed (31,32).

Other studies recently addressed the base pairing requirements for snoRNA-dependent pseudouridylation (64,65). In an *in-vivo* study in yeast (64) a requirement for at least eight nucleotide-long base pairing between the pseudouridylation pocket of the snoRNA and the target RNA was recently demonstrated. Indeed, we observe a similar requirement also in human cells, as five nucleotide-long target sequences (corresponding to the ‘3nt complementation’–3nt upstream of the uridine and following guanosine and two downstream (3,2)) do not lead to significant levels of modification (Figure 3), however a nine nucleotide-long complementation, corresponding to ‘5nt complementation’ (5,4) leads to a higher Ψ score, readily observed under the Pol-I promoter (Figure 3E). In addition it was suggested that the pseudouridylation pocket exhibits flexibility that allows it to tolerate at least four unpaired nucleotides (64). However, in a recent in vitro study it was shown that addition of a single nucleotide between the two unpaired nucleotides reduces the rate of Ψ formation about 10-fold (65). Here we report that deletion of the unpaired nucleotide following the modified uridine significantly impairs the levels of pseudouridylation (Supplementary Figure S1). These findings suggest that the pseudouridylation pocket is probably not a flexible structure, but rather that bulges of certain length can be formed in the target RNA to fit the pocket.

In the future, it will be interesting to exploit the massively parallel reporter based strategy that we develop here, to interrogate whether C/D box snoRNAs, guiding ribose methylations, are able to guide mRNA modifications. Although there have been a number of reports of physical interactions between C/D box snoRNAs and mRNA (25–29), it currently remains unclear whether such interactions give rise to 2'-*O*-ribose methylation of mRNA. We are aware of a single report to date in which a C/D box snoRNA was reported to guide methylation of an mRNA target (30), but this has not been systematically explored to date, and as such the prevalence of this phenomenon and the rules dictating such interactions are unknown.

This study expands our knowledge pertaining to the targeting scope of snoRNA, the underlying constraints and the consequences thereof. We anticipate that the systematic methodological toolkit developed in the present study will help to functionally and mechanistically dissect the consequences of mRNA pseudouridylation in the future.

## DATA AVAILABILITY

Ψ-seq datasets generated in this manuscript were deposited to GEO (GSE159749). These datasets were used to generate Supplementary Table S2 (used for Figures 1-4 and Supplementary Figures. S1-S2), Supplementary Table S3 (Figure 1K, L and Supplementary Figure S1B), Supplementary Table S4 (Figure 2B, C), Supplementary Table S5 (Figure 2F), Supplementary Table S6 (Supplementary Figure S1G), Supplementary Table S7 (Figure 4 and Supplementary Figures S1F and S2) and Figure 5B.

Code for the analyses described in this paper is available at https://github.com/aldemasas/pseudouridine.

## Supplementary Material

gkac347_Supplemental_Files
