# Supplementary material for: A systematic dissection of determinants and consequences of snoRNA-guided pseudouridylation of human mRNA
Source: Nucleic Acids Res. 2022 May 10;50(9):4900–16. doi: 10.1093/nar/gkac347 (PMC9122591; doi:10.1093/nar/gkac347)
Supplement: gkac347_Supplemental_Files [file gkac347_supplemental_files.zip › Nir_etal_TableS1.docx]

> rRNASSU18S

TACCTGGTTGATCCTGCCAGTAGCATATGCTTGTCTCAAAGATTAAGCCATGCATGTCTAAGTACGCACGGCCGGTACAGTGAAACTGCGAATGGCTCATTAAATCAGTTATGGTTCCTTTGGTCGCTCGCTCCTCTCCTACTTGGATAACTGTGGTAATTCTAGAGCTAATACATGCCGACGGGCGCTGACCCCCTTCGCGGGGGGGATGCGTGCATTTATCAGATCAAAACCAACCCGGTCAGCCCCTCTCCGGCCCCGGCCGGGGGGCGGGCGCCGGCGGCTTTGGTGACTCTAGATAACCTCGGGCCGATCGCACGCCCCCCGTGGCGGCGACGACCCATTCGAACGTCTGCCCTATCAACTTTCGATGGTAGTCGCCGTGCCTACCATGGTGACCACGGGTGACGGGGAATCAGGGTTCGATTCCGGAGAGGGAGCCTGAGAAACGGCTACCACATCCAAGGAAGGCAGCAGGCGCGCAAATTACCCACTCCCGACCCGGGGAGGTAGTGACGAAAAATAACAATACAGGACTCTTTCGAGGCCCTGTAATTGGAATGAGTCCACTTTAAATCCTTTAACGAGGATCCATTGGAGGGCAAGTCTGGTGCCAGCAGCCGCGGTAATTCCAGCTCCAATAGCGTATATTAAAGTTGCTGCAGTTAAAAAGCTCGTAGTTGGATCTTGGGAGCGGGCGGGCGGTCCGCCGCGAGGCGAGCCACCGCCCGTCCCCGCCCCTTGCCTCTCGGCGCCCCCTCGATGCTCTTAGCTGAGTGTCCCGCGGGGCCCGAAGCGTTTACTTTGAAAAAATTAGAGTGTTCAAAGCAGGCCCGAGCCGCCTGGATACCGCAGCTAGGAATAATGGAATAGGACCGCGGTTCTATTTTGTTGGTTTTCGGAACTGAGGCCATGATTAAGAGGGACGGCCGGGGGCATTCGTATTGCGCCGCTAGAGGTGAAATTCCTTGGACCGGCGCAAGACGGACCAGAGCGAAAGCATTTGCCAAGAATGTTTTCATTAATCAAGAACGAAAGTCGGAGGTTCGAAGACGATCAGATACCGTCGTAGTTCCGACCATAAACGATGCCGACCGGCGATGCGGCGGCGTTATTCCCATGACCCGCCGGGCAGCTTCCGGGAAACCAAAGTCTTTGGGTTCCGGGGGGAGTATGGTTGCAAAGCTGAAACTTAAAGGAATTGACGGAAGGGCACCACCAGGAGTGGAGCCTGCGGCTTAATTTGACTCAACACGGGAAACCTCACCCGGCCCGGACACGGACAGGATTGACAGATTGATAGCTCTTTCTCGATTCCGTGGGTGGTGGTGCATGGCCGTTCTTAGTTGGTGGAGCGATTTGTCTGGTTAATTCCGATAACGAACGAGACTCTGGCATGCTAACTAGTTACGCGACCCCCGAGCGGTCGGCGTCCCCCAACTTCTTAGAGGGACAAGTGGCGTTCAGCCACCCGAGATTGAGCAATAACAGGTCTGTGATGCCCTTAGATGTCCGGGGCTGCACGCGCGCTACACTGACTGGCTCAGCGTGTGCCTACCCTACGCCGGCAGGCGCGGGTAACCCGTTGAACCCCATTCGTGATGGGGATCGGGGATTGCAATTATTCCCCATGAACGAGGAATTCCCAGTAAGTGCGGGTCATAAGCTTGCGTTGATTAAGTCCCTGCCCTTTGTACACACCGCCCGTCGCTACTACCGATTGGATGGTTTAGTGAGGCCCTCGGATCGGCCCCGCCGGGGTCGGCCCACGGCCCTGGCGGAGCGCTGAGAAGACGGTCGAACTTGACTATCTAGAGGAAGTAAAAGTCGTAACAAGGTTTCCGTAGGTGAACCTGCGGAAGGATCATTA

> rRNALSUS58S

CGACTCTTAGCGGTGGATCACTCGGCTCGTGCGTCGATGAAGAACGCAGCGCTAGCTGCGAGAATTAATGTGAATTGCAGGACACATTGATCATCGACACTTCGAACGCACTTGCGGCCCCGGGTTCCTCCCGGGGCTACGCCTGTCTGAGCGTCGCTT

> rRNALSUL28S

CGCGACCTCAGATCAGACGTGGCGACCCGCTGAATTTAAGCATATTAGTCAGCGGAGGAAAAGAAACTAACCAGGATTCCCTCAGTAACGGCGAGTGAACAGGGAAGAGCCCAGCGCCGAATCCCCGCCCCGCGGGGCGCGGGACATGTGGCGTACGGAAGACCCGCTCCCCGGCGCCGCTCGTGGGGGGCCCAAGTCCTTCTGATCGAGGCCCAGCCCGTGGACGGTGTGAGGCCGGTAGCGGCCGGCGCGCGCCCGGGTCTTCCCGGAGTCGGGTTGCTTGGGAATGCAGCCCAAAGCGGGTGGTAAACTCCATCTAAGGCTAAATACCGGCACGAGACCGATAGTCAACAAGTACCGTAAGGGAAAGTTGAAAAGAACTTTGAAGAGAGAGTTCAAGAGGGCGTGAAACCGTTAAGAGGTAAACGGGTGGGGTCCGCGCAGTCCGCCCGGAGGATTCAACCCGGCGGCGGGTCCGGCCGTGTCGGCGGCCCGGCGGATCTTTCCCGCCCCCCGTTCCTCCCGACCCCTCCACCCGCCCTCCCTTCCCCCGCCGCCCCTCCTCCTCCTCCCCGGAGGGGGCGGGCTCCGGCGGGTGCGGGGGTGGGCGGGCGGGGCCGGGGGTGGGGTCGGCGGGGGACCGTCCCCCGGACCGGCGACCGGCCGCCGCCGGGCGCATTTCCAGGCGGTGCGCCGCGACCGGCTCCGGGACGGCTGGGAAGGCCCGGCGGGGAAGGTGGCTCGGGGGGCCCCGTCCGTCCGTCCGTCCTCCTCCTCCCCCGTCTCCGCCCCCCGGCCCCGCGTCCTCCCTCGGGAGGGCGCGCGGGTCGGGGCGGCGGCGGCGGCGGCGGTGGCGGCGGCGGCGGGGGCGGCGGGACCGAAACCCCCCCCGAGTGTTACAGCCCCCCCGGCAGCAGCACTCGCCGAATCCCGGGGCCGAGGGAGCGAGACCCGTCGCCGCGCTCTCCCCCCTCCCGGCGCCCACCCCCGCGGGAATCCCCGCGAGGGGGGTCTCCCCCGGCGCGGCGCCGGCGTCTCCTCGTGGGGGGGCCGGGCCACCCCTCCCACGGCGCGACCGCTCTCCCACCCCTCCTCCCCGCGCCCCCGCCCCGGCGACGGGGGGGGTGCCGCGCGCGGGTCGGGGGGCGGGGCGGACTGTCCCCAGTGCGCCCCGGGCGGGTCGCGCCGTCGGGCCCGGGGGAGGTTCTCTCGGGGCCACGCGCGCGTCCCCCGAAGAGGGGGACGGCGGAGCGAGCGCACGGGGTCGGCGGCGACGTCGGCTACCCACCCGACCCGTCTTGAAACACGGACCAAGGAGTCTAACACGTGCGCGAGTCGGGGGCTCGCACGAAAGCCGCCGTGGCGCAATGAAGGTGAAGGCCGGCGCGCTCGCCGGCCGAGGTGGGATCCCGAGGCCTCTCCAGTCCGCCGAGGGGCACCACCGGCCCGTCTCGCCCGCCGCGCCGGGGAGGTGGAGCACGAGCGCACGTGTTAGGACCCGAAAGATGGTGAACTATGCCTGGGCAGGGCGAAGCCAGAGGAAACTCTGGTGGAGGTCCGTAGCGGTCCTGACGTGCAAATCGGTCGTCCGACCTGGGTATAGGGGCGAAAGACTAATCGAACCATCTAGTAGCTGGTTCCCTCCGAAGTTTCCCTCAGGATAGCTGGCGCTCTCGCAGACCCGACGCACCCCCGCCACGCAGTTTTATCCGGTAAAGCGAATGATTAGAGGTCTTGGGGCCGAAACGATCTCAACCTATTCTCAAACTTTAAATGGGTAAGAAGCCCGGCTCGCTGGCGTGGAGCCGGGGTGGAATGCGAGTGCCTAGTGGGCCACTTTTGGTAAGCAGAACTGGCGCTGCGGGATGAACCGAACGCCGGGTTAAGGCGCCCGATGCCGACGCTCATCAGACCCCAGAAAAGGTGTTGGTTGATATAGACAGCAGGACGGTGGCCATGGAAGTCGGAATCCGCTAAGGAGTGTGTAACAACTCACCTGCCGAATCAACTAGCCCTGAAAATGGATGGCGCTGGAGCGTCGGGCCCATACCCGGCCGTCGCCGGCAGTCGAGAGTGGACGGGAGCGGCGGGGGCGGCGGCGCGCGCGCGCGTGTGGTGTGCGTCGGAGGGCGGCGGCGGCGGCGGCGGCGGGGGTGTGGGGTCCTTCCCCCGCCCCCCCCCCCACGCCTCCTCCCCTCCTCCCGCCCACGCCCCGCTCCCCGCCCCCGGAGCCCCGCGGAGCTACGCCGCGACGAGTAGGAGGGCCGCTGCGGTGAGCCTTGAAGCCTAGGGCGCGGGCCCGGGTGGAGGCCGCCGCAGGTGCAGATCTTGGTGGTAGTAGCAAATATTCAAACGAGAACTTTGAAGGCCGAAGTGGAGAAGGGTTCCATGTGAACAGCAGTTGAACATGGGTCAGTCGGTCCTGAGAGATGGGCGAGCGCCGTTCCGAAGGGACGGGCGATGGCCTCCGTTGCCCTCGGCCGATCGAAAGGGAGTCGGGTTCAGATCCCCGAATCCGGAGTGGCGGAGATGGGCGCCGCGAGGCGTCCAGTGCGGTAACGCGACCGATCCCGGAGAAGCCGGCGGGAGCCCCGGGGAGAGTTCTCTTTTCTTTGTGAAGGGCAGGGCGCCCTGGAATGGGTTCGCCCCGAGAGAGGGGCCCGTGCCTTGGAAAGCGTCGCGGTTCCGGCGGCGTCCGGTGAGCTCTCGCTGGCCCTTGAAAATCCGGGGGAGAGGGTGTAAATCTCGCGCCGGGCCGTACCCATATCCGCAGCAGGTCTCCAAGGTGAACAGCCTCTGGCATGTTGGAACAATGTAGGTAAGGGAAGTCGGCAAGCCGGATCCGTAACTTCGGGATAAGGATTGGCTCTAAGGGCTGGGTCGGTCGGGCTGGGGCGCGAAGCGGGGCTGGGCGCGCGCCGCGGCTGGACGAGGCGCGCGCCCCCCCCACGCCCGGGGCACCCCCCTCGCGGCCCTCCCCCGCCCCACCCGCGCGCGCCGCTCGCTCCCTCCCCACCCCGCGCCCTCTCTCTCTCTCTCTCCCCCGCTCCCCGTCCTCCCCCCTCCCCGGGGGAGCGCCGCGTGGGGGCGCGGCGGGGGGAGAAGGGTCGGGGCGGCAGGGGCCGCGCGGCGGCCGCCGGGGCGGCCGGCGGGGGCAGGTCCCCGCGAGGGGGGCCCCGGGGACCCGGGGGGCCGGCGGCGGCGCGGACTCTGGACGCGAGCCGGGCCCTTCCCGTGGATCGCCCCAGCTGCGGCGGGCGTCGCGGCCGCCCCCGGGGAGCCCGGCGGCGGCGCGGCGCGCCCCCCACCCCCACCCCACGTCTCGGTCGCGCGCGCGTCCGCTGGGGGCGGGAGCGGTCGGGCGGCGGCGGTCGGCGGGCGGCGGGGCGGGGCGGTTCGTCCCCCCGCCCTACCCCCCCGGCCCCGTCCGCCCCCCGTTCCCCCCTCCTCCTCGGCGCGCGGCGGCGGCGGCGGCAGGCGGCGGAGGGGCCGCGGGCCGGTCCCCCCCGCCGGGTCCGCCCCCGGGGCCGCGGTTCCGCGCGCGCCTCGCCTCGGCCGGCGCCTAGCAGCCGACTTAGAACTGGTGCGGACCAGGGGAATCCGACTGTTTAATTAAAACAAAGCATCGCGAAGGCCCGCGGCGGGTGTTGACGCGATGTGATTTCTGCCCAGTGCTCTGAATGTCAAAGTGAAGAAATTCAATGAAGCGCGGGTAAACGGCGGGAGTAACTATGACTCTCTTAAGGTAGCCAAATGCCTCGTCATCTAATTAGTGACGCGCATGAATGGATGAACGAGATTCCCACTGTCCCTACCTACTATCCAGCGAAACCACAGCCAAGGGAACGGGCTTGGCGGAATCAGCGGGGAAAGAAGACCCTGTTGAGCTTGACTCTAGTCTGGCACGGTGAAGAGACATGAGAGGTGTAGAATAAGTGGGAGGCCCCCGGCGCCCCCCCGGTGTCCCCGCGAGGGGCCCGGGGCGGGGTCCGCGGCCCTGCGGGCCGCCGGTGAAATACCACTACTCTGATCGTTTTTTCACTGACCCGGTGAGGCGGGGGGGCGAGCCCGAGGGGCTCTCGCTTCTGGCGCCAAGCGCCCGCCCGGCCGGGCGCGACCCGCTCCGGGGACAGTGCCAGGTGGGGAGTTTGACTGGGGCGGTACACCTGTCAAACGGTAACGCAGGTGTCCTAAGGCGAGCTCAGGGAGGACAGAAACCTCCCGTGGAGCAGAAGGGCAAAAGCTCGCTTGATCTTGATTTTCAGTACGAATACAGACCGTGAAAGCGGGGCCTCACGATCCTTCTGACCTTTTGGGTTTTAAGCAGGAGGTGTCAGAAAAGTTACCACAGGGATAACTGGCTTGTGGCGGCCAAGCGTTCATAGCGACGTCGCTTTTTGATCCTTCGATGTCGGCTCTTCCTATCATTGTGAAGCAGAATTCGCCAAGCGTTGGATTGTTCACCCACTAATAGGGAACGTGAGCTGGGTTTAGACCGTCGTGAGACAGGTTAGTTTTACCCTACTGATGATGTGTTGTTGCCATGGTAATCCTGCTCAGTACGAGAGGAACCGCAGGTTCAGACATTTGGTGTATGTGCTTGGCTGAGGAGCCAATGGGGCGAAGCTACCATCTGTGGGATTATGACTGAACGCCTCTAAGTCAGAATCCCGCCCAGGCGAACGATACGGCAGCGCCGCGGAGCCTCGGTTGGCCTCGGATAGCCGGTCCCCCGCCTGTCCCCGCCGGCGGGCCGCCCCCCCCTCCACGCGCCCCGCCGCGGGAGGGCGCGTGCCCCGCCGCGCGCCGGGACCGGGGTCCGGTGCGGAGTGCCCTTCGTCCTGGGAAACGGGGCGCGGCCGGAAAGGCGGCCGCCCCCTCGCCCGTCACGCACCGCACGTTCGTGGGGAACCTGGCGCTAAACCATTCGTAGACGACCTGCTTCTGGGTCGGGGTTTCGTACGTAGCAGAGCAGCTCCCTCGCTGCGATCTATTGAAAGTCAGCCCTCGACACAAGGGTTTGTC

>ACA1_SynSno_pointMutU

acctttgccaagccaatggcggctaactatctgaagaaccagccgatgtacgtgttccgt

aagacggagctcaagcactccaagaccgagctcaacttcaaggagtggcaaaaggccttt

accgatgtgatgggcatggacgagctgtacaagtaaactagtagcagacagatgcgccgt

ggataaattgttataggtctcattgggaagcataaccgaagttgcctgattatttctggc

agccgaattacgcagtcagccacccgatccaatgcagttggcgcgccattcgatatcgga

ccctttccctttagtgagggttaatgct

>ACA10_s1_SynSno_pointMutU

acctttgccaagccaatggcggctaactatctgaagaaccagccgatgtacgtgttccgt

aagacggagctcaagcactccaagaccgagctcaacttcaaggagtggcaaaaggccttt

accgatgtgatgggcatggacgagctgtacaagtaaactagtagcagacagatgcgccgt

ggataaattgttatacctgaaaacaggagctgagagagaagttgcctgattatttctggc

agccgaattctgtggaaagccacccgatccaatgcagttggcgcgccattcgatatcgga

ccctttccctttagtgagggttaatgct

>ACA10_s2_SynSno_pointMutU

acctttgccaagccaatggcggctaactatctgaagaaccagccgatgtacgtgttccgt

aagacggagctcaagcactccaagaccgagctcaacttcaaggagtggcaaaaggccttt

accgatgtgatgggcatggacgagctgtacaagtaaactagtagcagacagatgcgccgt

ggataaattgttataactcctaggaggcgtgcattaggaagttgcctgattatttctggc

agccgaattccccctgtagccacccgatccaatgcagttggcgcgccattcgatatcgga

ccctttccctttagtgagggttaatgct

>ACA13_SynSno_pointMutU

acctttgccaagccaatggcggctaactatctgaagaaccagccgatgtacgtgttccgt

aagacggagctcaagcactccaagaccgagctcaacttcaaggagtggcaaaaggccttt

accgatgtgatgggcatggacgagctgtacaagtaaactagtagcagacagatgcgccgt

ggataaattgttatacttttttgacggaacacaaagggaagttgcctgattatttctggc

agccgaattggtccgtgagccacccgatccaatgcagttggcgcgccattcgatatcgga

ccctttccctttagtgagggttaatgct

>ACA14a_SynSno_pointMutU

acctttgccaagccaatggcggctaactatctgaagaaccagccgatgtacgtgttccgt

aagacggagctcaagcactccaagaccgagctcaacttcaaggagtggcaaaaggccttt

accgatgtgatgggcatggacgagctgtacaagtaaactagtagcagacagatgcgccgt

ggataaattgttataggtatgaaatggttggactttcgaagttgcctgattatttctggc

agccgaattatgtcctcagccacccgatccaatgcagttggcgcgccattcgatatcgga

ccctttccctttagtgagggttaatgct

>ACA15_SynSno_pointMutU

acctttgccaagccaatggcggctaactatctgaagaaccagccgatgtacgtgttccgt

aagacggagctcaagcactccaagaccgagctcaacttcaaggagtggcaaaaggccttt

accgatgtgatgggcatggacgagctgtacaagtaaactagtagcagacagatgcgccgt

ggataaattgttatatggtgtctggggtattcggccagaagttgcctgattatttctggc

agccgaattgagaggtcagccacccgatccaatgcagttggcgcgccattcgatatcgga

ccctttccctttagtgagggttaatgct

>ACA16_SynSno_pointMutU

acctttgccaagccaatggcggctaactatctgaagaaccagccgatgtacgtgttccgt

aagacggagctcaagcactccaagaccgagctcaacttcaaggagtggcaaaaggccttt

accgatgtgatgggcatggacgagctgtacaagtaaactagtagcagacagatgcgccgt

ggataaattgttatatcctttttgaggcttcgataaggaagttgcctgattatttctggc

agccgaattgaggaaaaagccacccgatccaatgcagttggcgcgccattcgatatcgga

ccctttccctttagtgagggttaatgct

>ACA17_s1_SynSno_pointMutU

acctttgccaagccaatggcggctaactatctgaagaaccagccgatgtacgtgttccgt

aagacggagctcaagcactccaagaccgagctcaacttcaaggagtggcaaaaggccttt

accgatgtgatgggcatggacgagctgtacaagtaaactagtagcagacagatgcgccgt

ggataaattgttataacctaatgacggaacgcctctagaagttgcctgattatttctggc

agccgaattttacctgtagccacccgatccaatgcagttggcgcgccattcgatatcgga

ccctttccctttagtgagggttaatgct

>ACA17_s2_SynSno_pointMutU

acctttgccaagccaatggcggctaactatctgaagaaccagccgatgtacgtgttccgt

aagacggagctcaagcactccaagaccgagctcaacttcaaggagtggcaaaaggccttt

accgatgtgatgggcatggacgagctgtacaagtaaactagtagcagacagatgcgccgt

ggataaattgttatatatcaaacatggagcgtagacagaagttgcctgattatttctggc

agccgaatttttactaaagccacccgatccaatgcagttggcgcgccattcgatatcgga

ccctttccctttagtgagggttaatgct

>ACA19_s1_SynSno_pointMutU

acctttgccaagccaatggcggctaactatctgaagaaccagccgatgtacgtgttccgt

aagacggagctcaagcactccaagaccgagctcaacttcaaggagtggcaaaaggccttt

accgatgtgatgggcatggacgagctgtacaagtaaactagtagcagacagatgcgccgt

ggataaattgttatacataagaaatggaatgaaatgtgaagttgcctgattatttctggc

agccgaattccccctttagccacccgatccaatgcagttggcgcgccattcgatatcgga

ccctttccctttagtgagggttaatgct

>ACA19_s2_SynSno_pointMutU

acctttgccaagccaatggcggctaactatctgaagaaccagccgatgtacgtgttccgt

aagacggagctcaagcactccaagaccgagctcaacttcaaggagtggcaaaaggccttt

accgatgtgatgggcatggacgagctgtacaagtaaactagtagcagacagatgcgccgt

ggataaattgttataaaggagactgggtaattagcctgaagttgcctgattatttctggc

agccgaattctgttttcagccacccgatccaatgcagttggcgcgccattcgatatcgga

ccctttccctttagtgagggttaatgct

>ACA19_s3_SynSno_pointMutU

acctttgccaagccaatggcggctaactatctgaagaaccagccgatgtacgtgttccgt

aagacggagctcaagcactccaagaccgagctcaacttcaaggagtggcaaaaggccttt

accgatgtgatgggcatggacgagctgtacaagtaaactagtagcagacagatgcgccgt

ggataaattgttatacacataagaaggatgaaatgtggaagttgcctgattatttctggc

agccgaattcatgaagcagccacccgatccaatgcagttggcgcgccattcgatatcgga

ccctttccctttagtgagggttaatgct

>ACA20_SynSno_pointMutU

acctttgccaagccaatggcggctaactatctgaagaaccagccgatgtacgtgttccgt

aagacggagctcaagcactccaagaccgagctcaacttcaaggagtggcaaaaggccttt

accgatgtgatgggcatggacgagctgtacaagtaaactagtagcagacagatgcgccgt

ggataaattgttatacatgcgtataggaaataaatgggaagttgcctgattatttctggc

agccgaattaatccggaagccacccgatccaatgcagttggcgcgccattcgatatcgga

ccctttccctttagtgagggttaatgct

>ACA21_s1_SynSno_pointMutU

acctttgccaagccaatggcggctaactatctgaagaaccagccgatgtacgtgttccgt

aagacggagctcaagcactccaagaccgagctcaacttcaaggagtggcaaaaggccttt

accgatgtgatgggcatggacgagctgtacaagtaaactagtagcagacagatgcgccgt

ggataaattgttatatttcttgacggggcttttaaaagaagttgcctgattatttctggc

agccgaattattggttcagccacccgatccaatgcagttggcgcgccattcgatatcgga

ccctttccctttagtgagggttaatgct

>ACA21_s2_SynSno_pointMutU

acctttgccaagccaatggcggctaactatctgaagaaccagccgatgtacgtgttccgt

aagacggagctcaagcactccaagaccgagctcaacttcaaggagtggcaaaaggccttt

accgatgtgatgggcatggacgagctgtacaagtaaactagtagcagacagatgcgccgt

ggataaattgttataccaccgattgggcacccaaaaggaagttgcctgattatttctggc

agccgaattctcgtacgagccacccgatccaatgcagttggcgcgccattcgatatcgga

ccctttccctttagtgagggttaatgct

>ACA22_s1_SynSno_pointMutU

acctttgccaagccaatggcggctaactatctgaagaaccagccgatgtacgtgttccgt

aagacggagctcaagcactccaagaccgagctcaacttcaaggagtggcaaaaggccttt

accgatgtgatgggcatggacgagctgtacaagtaaactagtagcagacagatgcgccgt

ggataaattgttataagggtcaaagggtcactgtgcagaagttgcctgattatttctggc

agccgaatttcccattaagccacccgatccaatgcagttggcgcgccattcgatatcgga

ccctttccctttagtgagggttaatgct

>ACA22_s2_SynSno_pointMutU

acctttgccaagccaatggcggctaactatctgaagaaccagccgatgtacgtgttccgt

aagacggagctcaagcactccaagaccgagctcaacttcaaggagtggcaaaaggccttt

accgatgtgatgggcatggacgagctgtacaagtaaactagtagcagacagatgcgccgt

ggataaattgttatatctgaatacagggcagaggagagaagttgcctgattatttctggc

agccgaattcgtaggttagccacccgatccaatgcagttggcgcgccattcgatatcgga

ccctttccctttagtgagggttaatgct

>ACA23_s1_SynSno_pointMutU

acctttgccaagccaatggcggctaactatctgaagaaccagccgatgtacgtgttccgt

aagacggagctcaagcactccaagaccgagctcaacttcaaggagtggcaaaaggccttt

accgatgtgatgggcatggacgagctgtacaagtaaactagtagcagacagatgcgccgt

ggataaattgttatacatgatgtttggagcagccatggaagttgcctgattatttctggc

agccgaattaacgtgggagccacccgatccaatgcagttggcgcgccattcgatatcgga

ccctttccctttagtgagggttaatgct

>ACA23_s2_SynSno_pointMutU

acctttgccaagccaatggcggctaactatctgaagaaccagccgatgtacgtgttccgt

aagacggagctcaagcactccaagaccgagctcaacttcaaggagtggcaaaaggccttt

accgatgtgatgggcatggacgagctgtacaagtaaactagtagcagacagatgcgccgt

ggataaattgttatagaatttggagggactatgatccgaagttgcctgattatttctggc

agccgaattctagttacagccacccgatccaatgcagttggcgcgccattcgatatcgga

ccctttccctttagtgagggttaatgct

>ACA24_s1_SynSno_pointMutU

acctttgccaagccaatggcggctaactatctgaagaaccagccgatgtacgtgttccgt

aagacggagctcaagcactccaagaccgagctcaacttcaaggagtggcaaaaggccttt

accgatgtgatgggcatggacgagctgtacaagtaaactagtagcagacagatgcgccgt

ggataaattgttatatggctaggaaggaaagatacatgaagttgcctgattatttctggc

agccgaattttcccaccagccacccgatccaatgcagttggcgcgccattcgatatcgga

ccctttccctttagtgagggttaatgct

>ACA24_s2_SynSno_pointMutU

acctttgccaagccaatggcggctaactatctgaagaaccagccgatgtacgtgttccgt

aagacggagctcaagcactccaagaccgagctcaacttcaaggagtggcaaaaggccttt

accgatgtgatgggcatggacgagctgtacaagtaaactagtagcagacagatgcgccgt

ggataaattgttatatgtgcaagtcggttgccaataagaagttgcctgattatttctggc

agccgaattctgcgttcagccacccgatccaatgcagttggcgcgccattcgatatcgga

ccctttccctttagtgagggttaatgct

>ACA25_s1_SynSno_pointMutU

acctttgccaagccaatggcggctaactatctgaagaaccagccgatgtacgtgttccgt

aagacggagctcaagcactccaagaccgagctcaacttcaaggagtggcaaaaggccttt

accgatgtgatgggcatggacgagctgtacaagtaaactagtagcagacagatgcgccgt

ggataaattgttatatcacagcgttggctttgaaatggaagttgcctgattatttctggc

agccgaatttcgagagtagccacccgatccaatgcagttggcgcgccattcgatatcgga

ccctttccctttagtgagggttaatgct

>ACA25_s2_SynSno_pointMutU

acctttgccaagccaatggcggctaactatctgaagaaccagccgatgtacgtgttccgt

aagacggagctcaagcactccaagaccgagctcaacttcaaggagtggcaaaaggccttt

accgatgtgatgggcatggacgagctgtacaagtaaactagtagcagacagatgcgccgt

ggataaattgttatactcaggaaaaggggagtgctatgaagttgcctgattatttctggc

agccgaattgccctccgagccacccgatccaatgcagttggcgcgccattcgatatcgga

ccctttccctttagtgagggttaatgct

>ACA27_s1_SynSno_pointMutU

acctttgccaagccaatggcggctaactatctgaagaaccagccgatgtacgtgttccgt

aagacggagctcaagcactccaagaccgagctcaacttcaaggagtggcaaaaggccttt

accgatgtgatgggcatggacgagctgtacaagtaaactagtagcagacagatgcgccgt

ggataaattgttatacacttgaatgggaaagtgaaaagaagttgcctgattatttctggc

agccgaattgggtaaaaagccacccgatccaatgcagttggcgcgccattcgatatcgga

ccctttccctttagtgagggttaatgct

>ACA27_s2_SynSno_pointMutU

acctttgccaagccaatggcggctaactatctgaagaaccagccgatgtacgtgttccgt

aagacggagctcaagcactccaagaccgagctcaacttcaaggagtggcaaaaggccttt

accgatgtgatgggcatggacgagctgtacaagtaaactagtagcagacagatgcgccgt

ggataaattgttatatctgacaggagggttttaaaaggaagttgcctgattatttctggc

agccgaattctccatgtagccacccgatccaatgcagttggcgcgccattcgatatcgga

ccctttccctttagtgagggttaatgct

>ACA28_s1_SynSno_pointMutU

acctttgccaagccaatggcggctaactatctgaagaaccagccgatgtacgtgttccgt

aagacggagctcaagcactccaagaccgagctcaacttcaaggagtggcaaaaggccttt

accgatgtgatgggcatggacgagctgtacaagtaaactagtagcagacagatgcgccgt

ggataaattgttataaagctcaaatgggagtgttgctgaagttgcctgattatttctggc

agccgaattattcccttagccacccgatccaatgcagttggcgcgccattcgatatcgga

ccctttccctttagtgagggttaatgct

>ACA28_s2_SynSno_pointMutU

acctttgccaagccaatggcggctaactatctgaagaaccagccgatgtacgtgttccgt

aagacggagctcaagcactccaagaccgagctcaacttcaaggagtggcaaaaggccttt

accgatgtgatgggcatggacgagctgtacaagtaaactagtagcagacagatgcgccgt

ggataaattgttatagtctatataagggaataggttagaagttgcctgattatttctggc

agccgaattatgacggaagccacccgatccaatgcagttggcgcgccattcgatatcgga

ccctttccctttagtgagggttaatgct

>ACA2a_s1_SynSno_pointMutU

acctttgccaagccaatggcggctaactatctgaagaaccagccgatgtacgtgttccgt

aagacggagctcaagcactccaagaccgagctcaacttcaaggagtggcaaaaggccttt

accgatgtgatgggcatggacgagctgtacaagtaaactagtagcagacagatgcgccgt

ggataaattgttatatcctgtttgaggttgattcagggaagttgcctgattatttctggc

agccgaattcgcgggctagccacccgatccaatgcagttggcgcgccattcgatatcgga

ccctttccctttagtgagggttaatgct

>ACA2a_s2_SynSno_pointMutU

acctttgccaagccaatggcggctaactatctgaagaaccagccgatgtacgtgttccgt

aagacggagctcaagcactccaagaccgagctcaacttcaaggagtggcaaaaggccttt

accgatgtgatgggcatggacgagctgtacaagtaaactagtagcagacagatgcgccgt

ggataaattgttatatccaactgaaggcagaccacaagaagttgcctgattatttctggc

agccgaattcggtccgtagccacccgatccaatgcagttggcgcgccattcgatatcgga

ccctttccctttagtgagggttaatgct

>ACA3_s1_SynSno_pointMutU

acctttgccaagccaatggcggctaactatctgaagaaccagccgatgtacgtgttccgt

aagacggagctcaagcactccaagaccgagctcaacttcaaggagtggcaaaaggccttt

accgatgtgatgggcatggacgagctgtacaagtaaactagtagcagacagatgcgccgt

ggataaattgttataggactctagcgggactctagccgaagttgcctgattatttctggc

agccgaattgcgtcttgagccacccgatccaatgcagttggcgcgccattcgatatcgga

ccctttccctttagtgagggttaatgct

>ACA3_s2_SynSno_pointMutU

acctttgccaagccaatggcggctaactatctgaagaaccagccgatgtacgtgttccgt

aagacggagctcaagcactccaagaccgagctcaacttcaaggagtggcaaaaggccttt

accgatgtgatgggcatggacgagctgtacaagtaaactagtagcagacagatgcgccgt

ggataaattgttatataacctggtggggaataaggtcgaagttgcctgattatttctggc

agccgaattgtgaaggcagccacccgatccaatgcagttggcgcgccattcgatatcgga

ccctttccctttagtgagggttaatgct

>ACA3-2_SynSno_pointMutU

acctttgccaagccaatggcggctaactatctgaagaaccagccgatgtacgtgttccgt

aagacggagctcaagcactccaagaccgagctcaacttcaaggagtggcaaaaggccttt

accgatgtgatgggcatggacgagctgtacaagtaaactagtagcagacagatgcgccgt

ggataaattgttatagtactctagcgggactctagtcgaagttgcctgattatttctggc

agccgaattcgctaactagccacccgatccaatgcagttggcgcgccattcgatatcgga

ccctttccctttagtgagggttaatgct

>ACA30_SynSno_pointMutU

acctttgccaagccaatggcggctaactatctgaagaaccagccgatgtacgtgttccgt

aagacggagctcaagcactccaagaccgagctcaacttcaaggagtggcaaaaggccttt

accgatgtgatgggcatggacgagctgtacaagtaaactagtagcagacagatgcgccgt

ggataaattgttataccagctaccaggtgtgaaagtggaagttgcctgattatttctggc

agccgaattggttaggcagccacccgatccaatgcagttggcgcgccattcgatatcgga

ccctttccctttagtgagggttaatgct

>ACA31_s1_SynSno_pointMutU

acctttgccaagccaatggcggctaactatctgaagaaccagccgatgtacgtgttccgt

aagacggagctcaagcactccaagaccgagctcaacttcaaggagtggcaaaaggccttt

accgatgtgatgggcatggacgagctgtacaagtaaactagtagcagacagatgcgccgt

ggataaattgttatatcctagtgcaggtatcagtggagaagttgcctgattatttctggc

agccgaattcgagttaaagccacccgatccaatgcagttggcgcgccattcgatatcgga

ccctttccctttagtgagggttaatgct

>ACA31_s2_SynSno_pointMutU

acctttgccaagccaatggcggctaactatctgaagaaccagccgatgtacgtgttccgt

aagacggagctcaagcactccaagaccgagctcaacttcaaggagtggcaaaaggccttt

accgatgtgatgggcatggacgagctgtacaagtaaactagtagcagacagatgcgccgt

ggataaattgttatacccaattcaaggaagcgcaggggaagttgcctgattatttctggc

agccgaattcgggaacaagccacccgatccaatgcagttggcgcgccattcgatatcgga

ccctttccctttagtgagggttaatgct

>ACA32_SynSno_pointMutU

acctttgccaagccaatggcggctaactatctgaagaaccagccgatgtacgtgttccgt

aagacggagctcaagcactccaagaccgagctcaacttcaaggagtggcaaaaggccttt

accgatgtgatgggcatggacgagctgtacaagtaaactagtagcagacagatgcgccgt

ggataaattgttatatcatgtccacggttggtaatgagaagttgcctgattatttctggc

agccgaattcatctgctagccacccgatccaatgcagttggcgcgccattcgatatcgga

ccctttccctttagtgagggttaatgct

>ACA33_SynSno_pointMutU

acctttgccaagccaatggcggctaactatctgaagaaccagccgatgtacgtgttccgt

aagacggagctcaagcactccaagaccgagctcaacttcaaggagtggcaaaaggccttt

accgatgtgatgggcatggacgagctgtacaagtaaactagtagcagacagatgcgccgt

ggataaattgttataattctcagggggtcgtaacatggaagttgcctgattatttctggc

agccgaattttatatatagccacccgatccaatgcagttggcgcgccattcgatatcgga

ccctttccctttagtgagggttaatgct

>ACA34_s1_SynSno_pointMutU

acctttgccaagccaatggcggctaactatctgaagaaccagccgatgtacgtgttccgt

aagacggagctcaagcactccaagaccgagctcaacttcaaggagtggcaaaaggccttt

accgatgtgatgggcatggacgagctgtacaagtaaactagtagcagacagatgcgccgt

ggataaattgttatactctgcttgaggttcagtcagggaagttgcctgattatttctggc

agccgaattctgcttttagccacccgatccaatgcagttggcgcgccattcgatatcgga

ccctttccctttagtgagggttaatgct

>ACA34_s2_SynSno_pointMutU

acctttgccaagccaatggcggctaactatctgaagaaccagccgatgtacgtgttccgt

aagacggagctcaagcactccaagaccgagctcaacttcaaggagtggcaaaaggccttt

accgatgtgatgggcatggacgagctgtacaagtaaactagtagcagacagatgcgccgt

ggataaattgttatatccaaatgaaggcagaccacaggaagttgcctgattatttctggc

agccgaattggtgttggagccacccgatccaatgcagttggcgcgccattcgatatcgga

ccctttccctttagtgagggttaatgct

>ACA36_s1_SynSno_pointMutU

acctttgccaagccaatggcggctaactatctgaagaaccagccgatgtacgtgttccgt

aagacggagctcaagcactccaagaccgagctcaacttcaaggagtggcaaaaggccttt

accgatgtgatgggcatggacgagctgtacaagtaaactagtagcagacagatgcgccgt

ggataaattgttataaagtttaattggactcaacactgaagttgcctgattatttctggc

agccgaattttcaatctagccacccgatccaatgcagttggcgcgccattcgatatcgga

ccctttccctttagtgagggttaatgct

>ACA36_s2_SynSno_pointMutU

acctttgccaagccaatggcggctaactatctgaagaaccagccgatgtacgtgttccgt

aagacggagctcaagcactccaagaccgagctcaacttcaaggagtggcaaaaggccttt

accgatgtgatgggcatggacgagctgtacaagtaaactagtagcagacagatgcgccgt

ggataaattgttatagggaggcaaaggaatcatctccgaagttgcctgattatttctggc

agccgaattaccctggaagccacccgatccaatgcagttggcgcgccattcgatatcgga

ccctttccctttagtgagggttaatgct

>ACA4_SynSno_pointMutU

acctttgccaagccaatggcggctaactatctgaagaaccagccgatgtacgtgttccgt

aagacggagctcaagcactccaagaccgagctcaacttcaaggagtggcaaaaggccttt

accgatgtgatgggcatggacgagctgtacaagtaaactagtagcagacagatgcgccgt

ggataaattgttataacaaaatagtgggtggagtgtggaagttgcctgattatttctggc

agccgaattctattcatagccacccgatccaatgcagttggcgcgccattcgatatcgga

ccctttccctttagtgagggttaatgct

>ACA40_SynSno_pointMutU

acctttgccaagccaatggcggctaactatctgaagaaccagccgatgtacgtgttccgt

aagacggagctcaagcactccaagaccgagctcaacttcaaggagtggcaaaaggccttt

accgatgtgatgggcatggacgagctgtacaagtaaactagtagcagacagatgcgccgt

ggataaattgttatacttatatgtagggttgcaaaaggaagttgcctgattatttctggc

agccgaattgaccgtgaagccacccgatccaatgcagttggcgcgccattcgatatcgga

ccctttccctttagtgagggttaatgct

>ACA41_SynSno_pointMutU

acctttgccaagccaatggcggctaactatctgaagaaccagccgatgtacgtgttccgt

aagacggagctcaagcactccaagaccgagctcaacttcaaggagtggcaaaaggccttt

accgatgtgatgggcatggacgagctgtacaagtaaactagtagcagacagatgcgccgt

ggataaattgttataacagaggaatggccagtagctggaagttgcctgattatttctggc

agccgaatttagaacttagccacccgatccaatgcagttggcgcgccattcgatatcgga

ccctttccctttagtgagggttaatgct

>ACA42_s1_SynSno_pointMutU

acctttgccaagccaatggcggctaactatctgaagaaccagccgatgtacgtgttccgt

aagacggagctcaagcactccaagaccgagctcaacttcaaggagtggcaaaaggccttt

accgatgtgatgggcatggacgagctgtacaagtaaactagtagcagacagatgcgccgt

ggataaattgttatatgggtacactggaaatccattagaagttgcctgattatttctggc

agccgaattttacagaaagccacccgatccaatgcagttggcgcgccattcgatatcgga

ccctttccctttagtgagggttaatgct

>ACA42_s2_SynSno_pointMutU

acctttgccaagccaatggcggctaactatctgaagaaccagccgatgtacgtgttccgt

aagacggagctcaagcactccaagaccgagctcaacttcaaggagtggcaaaaggccttt

accgatgtgatgggcatggacgagctgtacaagtaaactagtagcagacagatgcgccgt

ggataaattgttatacccccttcagggatggttcgaggaagttgcctgattatttctggc

agccgaatttgacatcgagccacccgatccaatgcagttggcgcgccattcgatatcgga

ccctttccctttagtgagggttaatgct

>ACA43_SynSno_pointMutU

acctttgccaagccaatggcggctaactatctgaagaaccagccgatgtacgtgttccgt

aagacggagctcaagcactccaagaccgagctcaacttcaaggagtggcaaaaggccttt

accgatgtgatgggcatggacgagctgtacaagtaaactagtagcagacagatgcgccgt

ggataaattgttatacataaaccatgggtagaaatcggaagttgcctgattatttctggc

agccgaattggtgggggagccacccgatccaatgcagttggcgcgccattcgatatcgga

ccctttccctttagtgagggttaatgct

>ACA44_s1_SynSno_pointMutU

acctttgccaagccaatggcggctaactatctgaagaaccagccgatgtacgtgttccgt

aagacggagctcaagcactccaagaccgagctcaacttcaaggagtggcaaaaggccttt

accgatgtgatgggcatggacgagctgtacaagtaaactagtagcagacagatgcgccgt

ggataaattgttatatgcagttggaggttggaaacatgaagttgcctgattatttctggc

agccgaattctcacggaagccacccgatccaatgcagttggcgcgccattcgatatcgga

ccctttccctttagtgagggttaatgct

>ACA44_s2_SynSno_pointMutU

acctttgccaagccaatggcggctaactatctgaagaaccagccgatgtacgtgttccgt

aagacggagctcaagcactccaagaccgagctcaacttcaaggagtggcaaaaggccttt

accgatgtgatgggcatggacgagctgtacaagtaaactagtagcagacagatgcgccgt

ggataaattgttataaagctgagtgggcaaagtctttgaagttgcctgattatttctggc

agccgaatttgacgaagagccacccgatccaatgcagttggcgcgccattcgatatcgga

ccctttccctttagtgagggttaatgct

>ACA46_SynSno_pointMutU

acctttgccaagccaatggcggctaactatctgaagaaccagccgatgtacgtgttccgt

aagacggagctcaagcactccaagaccgagctcaacttcaaggagtggcaaaaggccttt

accgatgtgatgggcatggacgagctgtacaagtaaactagtagcagacagatgcgccgt

ggataaattgttatactacagcgtaggttaaatataggaagttgcctgattatttctggc

agccgaatttaaaagggagccacccgatccaatgcagttggcgcgccattcgatatcgga

ccctttccctttagtgagggttaatgct

>ACA48_SynSno_pointMutU

acctttgccaagccaatggcggctaactatctgaagaaccagccgatgtacgtgttccgt

aagacggagctcaagcactccaagaccgagctcaacttcaaggagtggcaaaaggccttt

accgatgtgatgggcatggacgagctgtacaagtaaactagtagcagacagatgcgccgt

ggataaattgttataaaggcatgaagggatcaaaagcgaagttgcctgattatttctggc

agccgaattgctcgcacagccacccgatccaatgcagttggcgcgccattcgatatcgga

ccctttccctttagtgagggttaatgct

>U19_up_SynSno_pointMutU

acctttgccaagccaatggcggctaactatctgaagaaccagccgatgtacgtgttccgt

aagacggagctcaagcactccaagaccgagctcaacttcaaggagtggcaaaaggccttt

accgatgtgatgggcatggacgagctgtacaagtaaactagtagcagacagatgcgccgt

ggataaattgttataacagtgtaacggtgacaaccgcgaagttgcctgattatttctggc

agccgaattgagttaccagccacccgatccaatgcagttggcgcgccattcgatatcgga

ccctttccctttagtgagggttaatgct

>U19_dn_SynSno_pointMutU

acctttgccaagccaatggcggctaactatctgaagaaccagccgatgtacgtgttccgt

aagacggagctcaagcactccaagaccgagctcaacttcaaggagtggcaaaaggccttt

accgatgtgatgggcatggacgagctgtacaagtaaactagtagcagacagatgcgccgt

ggataaattgttataacccagactaggctctcagagggaagttgcctgattatttctggc

agccgaattgtgagcggagccacccgatccaatgcagttggcgcgccattcgatatcgga

ccctttccctttagtgagggttaatgct

>U23_up_SynSno_pointMutU

acctttgccaagccaatggcggctaactatctgaagaaccagccgatgtacgtgttccgt

aagacggagctcaagcactccaagaccgagctcaacttcaaggagtggcaaaaggccttt

accgatgtgatgggcatggacgagctgtacaagtaaactagtagcagacagatgcgccgt

ggataaattgttatactcgtgcgaagggctcaatgaggaagttgcctgattatttctggc

agccgaattaactatatagccacccgatccaatgcagttggcgcgccattcgatatcgga

ccctttccctttagtgagggttaatgct

>U64_up_SynSno_pointMutU

acctttgccaagccaatggcggctaactatctgaagaaccagccgatgtacgtgttccgt

aagacggagctcaagcactccaagaccgagctcaacttcaaggagtggcaaaaggccttt

accgatgtgatgggcatggacgagctgtacaagtaaactagtagcagacagatgcgccgt

ggataaattgttataccccgttacggggcagagccgagaagttgcctgattatttctggc

agccgaattaacaggttagccacccgatccaatgcagttggcgcgccattcgatatcgga

ccctttccctttagtgagggttaatgct

>U65_up_SynSno_pointMutU

acctttgccaagccaatggcggctaactatctgaagaaccagccgatgtacgtgttccgt

aagacggagctcaagcactccaagaccgagctcaacttcaaggagtggcaaaaggccttt

accgatgtgatgggcatggacgagctgtacaagtaaactagtagcagacagatgcgccgt

ggataaattgttatacaccactggagggtggcgggtggaagttgcctgattatttctggc

agccgaatttcgtagttagccacccgatccaatgcagttggcgcgccattcgatatcgga

ccctttccctttagtgagggttaatgct

>U65_dn_SynSno_pointMutU

acctttgccaagccaatggcggctaactatctgaagaaccagccgatgtacgtgttccgt

aagacggagctcaagcactccaagaccgagctcaacttcaaggagtggcaaaaggccttt

accgatgtgatgggcatggacgagctgtacaagtaaactagtagcagacagatgcgccgt

ggataaattgttatatgctttcggcggttcctaagctgaagttgcctgattatttctggc

agccgaatttatgcttgagccacccgatccaatgcagttggcgcgccattcgatatcgga

ccctttccctttagtgagggttaatgct

>U66_up_SynSno_pointMutU

acctttgccaagccaatggcggctaactatctgaagaaccagccgatgtacgtgttccgt

aagacggagctcaagcactccaagaccgagctcaacttcaaggagtggcaaaaggccttt

accgatgtgatgggcatggacgagctgtacaagtaaactagtagcagacagatgcgccgt

ggataaattgttataacctggttccggtgatcgagttgaagttgcctgattatttctggc

agccgaattgtataactagccacccgatccaatgcagttggcgcgccattcgatatcgga

ccctttccctttagtgagggttaatgct

>U67_up_SynSno_pointMutU

acctttgccaagccaatggcggctaactatctgaagaaccagccgatgtacgtgttccgt

aagacggagctcaagcactccaagaccgagctcaacttcaaggagtggcaaaaggccttt

accgatgtgatgggcatggacgagctgtacaagtaaactagtagcagacagatgcgccgt

ggataaattgttataagctgctactgggagggaatcagaagttgcctgattatttctggc

agccgaattggtagctaagccacccgatccaatgcagttggcgcgccattcgatatcgga

ccctttccctttagtgagggttaatgct

>U68_up_SynSno_pointMutU

acctttgccaagccaatggcggctaactatctgaagaaccagccgatgtacgtgttccgt

aagacggagctcaagcactccaagaccgagctcaacttcaaggagtggcaaaaggccttt

accgatgtgatgggcatggacgagctgtacaagtaaactagtagcagacagatgcgccgt

ggataaattgttatatccaaattcagggcgacaagatgaagttgcctgattatttctggc

agccgaattgtattgggagccacccgatccaatgcagttggcgcgccattcgatatcgga

ccctttccctttagtgagggttaatgct

>U69_up_SynSno_pointMutU

acctttgccaagccaatggcggctaactatctgaagaaccagccgatgtacgtgttccgt

aagacggagctcaagcactccaagaccgagctcaacttcaaggagtggcaaaaggccttt

accgatgtgatgggcatggacgagctgtacaagtaaactagtagcagacagatgcgccgt

ggataaattgttatacaggataatgggaattgcaaccgaagttgcctgattatttctggc

agccgaattacactacaagccacccgatccaatgcagttggcgcgccattcgatatcgga

ccctttccctttagtgagggttaatgct

>U69_dn_SynSno_pointMutU

acctttgccaagccaatggcggctaactatctgaagaaccagccgatgtacgtgttccgt

aagacggagctcaagcactccaagaccgagctcaacttcaaggagtggcaaaaggccttt

accgatgtgatgggcatggacgagctgtacaagtaaactagtagcagacagatgcgccgt

ggataaattgttatatcaatctgtcggaaaaatgattgaagttgcctgattatttctggc

agccgaatttgtatcaaagccacccgatccaatgcagttggcgcgccattcgatatcgga

ccctttccctttagtgagggttaatgct

>U70_up_SynSno_pointMutU

acctttgccaagccaatggcggctaactatctgaagaaccagccgatgtacgtgttccgt

aagacggagctcaagcactccaagaccgagctcaacttcaaggagtggcaaaaggccttt

accgatgtgatgggcatggacgagctgtacaagtaaactagtagcagacagatgcgccgt

ggataaattgttataaaggtcccttggtacactaccagaagttgcctgattatttctggc

agccgaattgtaattatagccacccgatccaatgcagttggcgcgccattcgatatcgga

ccctttccctttagtgagggttaatgct

>U71a_up_SynSno_pointMutU

acctttgccaagccaatggcggctaactatctgaagaaccagccgatgtacgtgttccgt

aagacggagctcaagcactccaagaccgagctcaacttcaaggagtggcaaaaggccttt

accgatgtgatgggcatggacgagctgtacaagtaaactagtagcagacagatgcgccgt

ggataaattgttataaagcttcaggggacggggacaagaagttgcctgattatttctggc

agccgaattctagcgtaagccacccgatccaatgcagttggcgcgccattcgatatcgga

ccctttccctttagtgagggttaatgct

>U72_up_SynSno_pointMutU

acctttgccaagccaatggcggctaactatctgaagaaccagccgatgtacgtgttccgt

aagacggagctcaagcactccaagaccgagctcaacttcaaggagtggcaaaaggccttt

accgatgtgatgggcatggacgagctgtacaagtaaactagtagcagacagatgcgccgt

ggataaattgttataaatgtttagcggcgagaatattgaagttgcctgattatttctggc

agccgaatttggggccaagccacccgatccaatgcagttggcgcgccattcgatatcgga

ccctttccctttagtgagggttaatgct

>U99_up_SynSno_pointMutU

acctttgccaagccaatggcggctaactatctgaagaaccagccgatgtacgtgttccgt

aagacggagctcaagcactccaagaccgagctcaacttcaaggagtggcaaaaggccttt

accgatgtgatgggcatggacgagctgtacaagtaaactagtagcagacagatgcgccgt

ggataaattgttatatccgtacattggccaagaaaaggaagttgcctgattatttctggc

agccgaattccgaagacagccacccgatccaatgcagttggcgcgccattcgatatcgga

ccctttccctttagtgagggttaatgct

>ACA5_s1_SynSno_pointMutU

acctttgccaagccaatggcggctaactatctgaagaaccagccgatgtacgtgttccgt

aagacggagctcaagcactccaagaccgagctcaacttcaaggagtggcaaaaggccttt

accgatgtgatgggcatggacgagctgtacaagtaaactagtagcagacagatgcgccgt

ggataaattgttataccttctgggcggaatttgacacgaagttgcctgattatttctggc

agccgaattcccattccagccacccgatccaatgcagttggcgcgccattcgatatcgga

ccctttccctttagtgagggttaatgct

>ACA5_s2_SynSno_pointMutU

acctttgccaagccaatggcggctaactatctgaagaaccagccgatgtacgtgttccgt

aagacggagctcaagcactccaagaccgagctcaacttcaaggagtggcaaaaggccttt

accgatgtgatgggcatggacgagctgtacaagtaaactagtagcagacagatgcgccgt

ggataaattgttataccaaatttatggcccatgagtcgaagttgcctgattatttctggc

agccgaattggacatcgagccacccgatccaatgcagttggcgcgccattcgatatcgga

ccctttccctttagtgagggttaatgct

>ACA50_s1_SynSno_pointMutU

acctttgccaagccaatggcggctaactatctgaagaaccagccgatgtacgtgttccgt

aagacggagctcaagcactccaagaccgagctcaacttcaaggagtggcaaaaggccttt

accgatgtgatgggcatggacgagctgtacaagtaaactagtagcagacagatgcgccgt

ggataaattgttatactgttgcttgggtcaaaggcaggaagttgcctgattatttctggc

agccgaattcccaaaagagccacccgatccaatgcagttggcgcgccattcgatatcgga

ccctttccctttagtgagggttaatgct

>ACA50_s2_SynSno_pointMutU

acctttgccaagccaatggcggctaactatctgaagaaccagccgatgtacgtgttccgt

aagacggagctcaagcactccaagaccgagctcaacttcaaggagtggcaaaaggccttt

accgatgtgatgggcatggacgagctgtacaagtaaactagtagcagacagatgcgccgt

ggataaattgttatatgtttttaaaggagttataacagaagttgcctgattatttctggc

agccgaattgctatagaagccacccgatccaatgcagttggcgcgccattcgatatcgga

ccctttccctttagtgagggttaatgct

>ACA52_SynSno_pointMutU

acctttgccaagccaatggcggctaactatctgaagaaccagccgatgtacgtgttccgt

aagacggagctcaagcactccaagaccgagctcaacttcaaggagtggcaaaaggccttt

accgatgtgatgggcatggacgagctgtacaagtaaactagtagcagacagatgcgccgt

ggataaattgttatacacaagcgaaggattaggatgggaagttgcctgattatttctggc

agccgaattcttgcttgagccacccgatccaatgcagttggcgcgccattcgatatcgga

ccctttccctttagtgagggttaatgct

>ACA54_s1_SynSno_pointMutU

acctttgccaagccaatggcggctaactatctgaagaaccagccgatgtacgtgttccgt

aagacggagctcaagcactccaagaccgagctcaacttcaaggagtggcaaaaggccttt

accgatgtgatgggcatggacgagctgtacaagtaaactagtagcagacagatgcgccgt

ggataaattgttataccggaatggaggtacgaacagtgaagttgcctgattatttctggc

agccgaattgaaggcggagccacccgatccaatgcagttggcgcgccattcgatatcgga

ccctttccctttagtgagggttaatgct

>ACA54_s2_SynSno_pointMutU

acctttgccaagccaatggcggctaactatctgaagaaccagccgatgtacgtgttccgt

aagacggagctcaagcactccaagaccgagctcaacttcaaggagtggcaaaaggccttt

accgatgtgatgggcatggacgagctgtacaagtaaactagtagcagacagatgcgccgt

ggataaattgttatactgaaactgaggatgtgtcgctgaagttgcctgattatttctggc

agccgaattagatctttagccacccgatccaatgcagttggcgcgccattcgatatcgga

ccctttccctttagtgagggttaatgct

>ACA55_SynSno_pointMutU

acctttgccaagccaatggcggctaactatctgaagaaccagccgatgtacgtgttccgt

aagacggagctcaagcactccaagaccgagctcaacttcaaggagtggcaaaaggccttt

accgatgtgatgggcatggacgagctgtacaagtaaactagtagcagacagatgcgccgt

ggataaattgttataaccatctgtcggaaagattcaggaagttgcctgattatttctggc

agccgaatttgtgctccagccacccgatccaatgcagttggcgcgccattcgatatcgga

ccctttccctttagtgagggttaatgct

>ACA56_SynSno_pointMutU

acctttgccaagccaatggcggctaactatctgaagaaccagccgatgtacgtgttccgt

aagacggagctcaagcactccaagaccgagctcaacttcaaggagtggcaaaaggccttt

accgatgtgatgggcatggacgagctgtacaagtaaactagtagcagacagatgcgccgt

ggataaattgttataactgactcccgggggataactggaagttgcctgattatttctggc

agccgaatttggttaagagccacccgatccaatgcagttggcgcgccattcgatatcgga

ccctttccctttagtgagggttaatgct

>ACA58_SynSno_pointMutU

acctttgccaagccaatggcggctaactatctgaagaaccagccgatgtacgtgttccgt

aagacggagctcaagcactccaagaccgagctcaacttcaaggagtggcaaaaggccttt

accgatgtgatgggcatggacgagctgtacaagtaaactagtagcagacagatgcgccgt

ggataaattgttataacactgtcccggtctacgagtagaagttgcctgattatttctggc

agccgaattagcatagtagccacccgatccaatgcagttggcgcgccattcgatatcgga

ccctttccctttagtgagggttaatgct

>ACA6_SynSno_pointMutU

acctttgccaagccaatggcggctaactatctgaagaaccagccgatgtacgtgttccgt

aagacggagctcaagcactccaagaccgagctcaacttcaaggagtggcaaaaggccttt

accgatgtgatgggcatggacgagctgtacaagtaaactagtagcagacagatgcgccgt

ggataaattgttatacacttccgacggtttaatagtggaagttgcctgattatttctggc

agccgaattcagcccatagccacccgatccaatgcagttggcgcgccattcgatatcgga

ccctttccctttagtgagggttaatgct

>ACA60_SynSno_pointMutU

acctttgccaagccaatggcggctaactatctgaagaaccagccgatgtacgtgttccgt

aagacggagctcaagcactccaagaccgagctcaacttcaaggagtggcaaaaggccttt

accgatgtgatgggcatggacgagctgtacaagtaaactagtagcagacagatgcgccgt

ggataaattgttatacgccagcattggccaaacacgggaagttgcctgattatttctggc

agccgaattaaatgccaagccacccgatccaatgcagttggcgcgccattcgatatcgga

ccctttccctttagtgagggttaatgct

>ACA61_SynSno_pointMutU

acctttgccaagccaatggcggctaactatctgaagaaccagccgatgtacgtgttccgt

aagacggagctcaagcactccaagaccgagctcaacttcaaggagtggcaaaaggccttt

accgatgtgatgggcatggacgagctgtacaagtaaactagtagcagacagatgcgccgt

ggataaattgttataccttttacgagggaaagggatcgaagttgcctgattatttctggc

agccgaatttgcccgacagccacccgatccaatgcagttggcgcgccattcgatatcgga

ccctttccctttagtgagggttaatgct

>ACA62_s1_SynSno_pointMutU

acctttgccaagccaatggcggctaactatctgaagaaccagccgatgtacgtgttccgt

aagacggagctcaagcactccaagaccgagctcaacttcaaggagtggcaaaaggccttt

accgatgtgatgggcatggacgagctgtacaagtaaactagtagcagacagatgcgccgt

ggataaattgttatactgcagattgggtcaaagacaggaagttgcctgattatttctggc

agccgaattgctaatacagccacccgatccaatgcagttggcgcgccattcgatatcgga

ccctttccctttagtgagggttaatgct

>ACA62_s2_SynSno_pointMutU

acctttgccaagccaatggcggctaactatctgaagaaccagccgatgtacgtgttccgt

aagacggagctcaagcactccaagaccgagctcaacttcaaggagtggcaaaaggccttt

accgatgtgatgggcatggacgagctgtacaagtaaactagtagcagacagatgcgccgt

ggataaattgttatacgcggttaaaggagttatgcgcgaagttgcctgattatttctggc

agccgaattcaacaacaagccacccgatccaatgcagttggcgcgccattcgatatcgga

ccctttccctttagtgagggttaatgct

>ACA63_SynSno_pointMutU

acctttgccaagccaatggcggctaactatctgaagaaccagccgatgtacgtgttccgt

aagacggagctcaagcactccaagaccgagctcaacttcaaggagtggcaaaaggccttt

accgatgtgatgggcatggacgagctgtacaagtaaactagtagcagacagatgcgccgt

ggataaattgttatagacagaaaaaggatagtgagtcgaagttgcctgattatttctggc

agccgaattccgggcgaagccacccgatccaatgcagttggcgcgccattcgatatcgga

ccctttccctttagtgagggttaatgct

>ACA64_SynSno_pointMutU

acctttgccaagccaatggcggctaactatctgaagaaccagccgatgtacgtgttccgt

aagacggagctcaagcactccaagaccgagctcaacttcaaggagtggcaaaaggccttt

accgatgtgatgggcatggacgagctgtacaagtaaactagtagcagacagatgcgccgt

ggataaattgttatagcctgggtttggagctgaggccgaagttgcctgattatttctggc

agccgaattagggtctgagccacccgatccaatgcagttggcgcgccattcgatatcgga

ccctttccctttagtgagggttaatgct

>ACA65_s1_SynSno_pointMutU

acctttgccaagccaatggcggctaactatctgaagaaccagccgatgtacgtgttccgt

aagacggagctcaagcactccaagaccgagctcaacttcaaggagtggcaaaaggccttt

accgatgtgatgggcatggacgagctgtacaagtaaactagtagcagacagatgcgccgt

ggataaattgttatatgacctaaaaggagaacagtcagaagttgcctgattatttctggc

agccgaattcattcgggagccacccgatccaatgcagttggcgcgccattcgatatcgga

ccctttccctttagtgagggttaatgct

>ACA65_s2_SynSno_pointMutU

acctttgccaagccaatggcggctaactatctgaagaaccagccgatgtacgtgttccgt

aagacggagctcaagcactccaagaccgagctcaacttcaaggagtggcaaaaggccttt

accgatgtgatgggcatggacgagctgtacaagtaaactagtagcagacagatgcgccgt

ggataaattgttatattccgcaaatgggtgaagaaaagaagttgcctgattatttctggc

agccgaattttactcgcagccacccgatccaatgcagttggcgcgccattcgatatcgga

ccctttccctttagtgagggttaatgct

>ACA67_s1_SynSno_pointMutU

acctttgccaagccaatggcggctaactatctgaagaaccagccgatgtacgtgttccgt

aagacggagctcaagcactccaagaccgagctcaacttcaaggagtggcaaaaggccttt

accgatgtgatgggcatggacgagctgtacaagtaaactagtagcagacagatgcgccgt

ggataaattgttataatggggtactggaaatccaaacgaagttgcctgattatttctggc

agccgaatttgatttgtagccacccgatccaatgcagttggcgcgccattcgatatcgga

ccctttccctttagtgagggttaatgct

>ACA67_s2_SynSno_pointMutU

acctttgccaagccaatggcggctaactatctgaagaaccagccgatgtacgtgttccgt

aagacggagctcaagcactccaagaccgagctcaacttcaaggagtggcaaaaggccttt

accgatgtgatgggcatggacgagctgtacaagtaaactagtagcagacagatgcgccgt

ggataaattgttatacccacttcagggatggttcagggaagttgcctgattatttctggc

agccgaattgtaaagctagccacccgatccaatgcagttggcgcgccattcgatatcgga

ccctttccctttagtgagggttaatgct

>ACA7_s1_SynSno_pointMutU

acctttgccaagccaatggcggctaactatctgaagaaccagccgatgtacgtgttccgt

aagacggagctcaagcactccaagaccgagctcaacttcaaggagtggcaaaaggccttt

accgatgtgatgggcatggacgagctgtacaagtaaactagtagcagacagatgcgccgt

ggataaattgttatatccctttccggggcgatcccaggaagttgcctgattatttctggc

agccgaattcatgccgaagccacccgatccaatgcagttggcgcgccattcgatatcgga

ccctttccctttagtgagggttaatgct

>ACA7_s2_SynSno_pointMutU

acctttgccaagccaatggcggctaactatctgaagaaccagccgatgtacgtgttccgt

aagacggagctcaagcactccaagaccgagctcaacttcaaggagtggcaaaaggccttt

accgatgtgatgggcatggacgagctgtacaagtaaactagtagcagacagatgcgccgt

ggataaattgttataagagtgtcttggaatgggtgccgaagttgcctgattatttctggc

agccgaattccagcgacagccacccgatccaatgcagttggcgcgccattcgatatcgga

ccctttccctttagtgagggttaatgct

>ACA8_s1_SynSno_pointMutU

acctttgccaagccaatggcggctaactatctgaagaaccagccgatgtacgtgttccgt

aagacggagctcaagcactccaagaccgagctcaacttcaaggagtggcaaaaggccttt

accgatgtgatgggcatggacgagctgtacaagtaaactagtagcagacagatgcgccgt

ggataaattgttatatgacctttgaggagataccatggaagttgcctgattatttctggc

agccgaattatgttgaaagccacccgatccaatgcagttggcgcgccattcgatatcgga

ccctttccctttagtgagggttaatgct

>ACA8_s2_SynSno_pointMutU

acctttgccaagccaatggcggctaactatctgaagaaccagccgatgtacgtgttccgt

aagacggagctcaagcactccaagaccgagctcaacttcaaggagtggcaaaaggccttt

accgatgtgatgggcatggacgagctgtacaagtaaactagtagcagacagatgcgccgt

ggataaattgttataagaaataccaggcaacgatgccgaagttgcctgattatttctggc

agccgaatttgaggacaagccacccgatccaatgcagttggcgcgccattcgatatcgga

ccctttccctttagtgagggttaatgct

>ACA9_s1_SynSno_pointMutU

acctttgccaagccaatggcggctaactatctgaagaaccagccgatgtacgtgttccgt

aagacggagctcaagcactccaagaccgagctcaacttcaaggagtggcaaaaggccttt

accgatgtgatgggcatggacgagctgtacaagtaaactagtagcagacagatgcgccgt

ggataaattgttatacactgaaggagggctggaggctgaagttgcctgattatttctggc

agccgaattcaagctgaagccacccgatccaatgcagttggcgcgccattcgatatcgga

ccctttccctttagtgagggttaatgct

>ACA9_s2_SynSno_pointMutU

acctttgccaagccaatggcggctaactatctgaagaaccagccgatgtacgtgttccgt

aagacggagctcaagcactccaagaccgagctcaacttcaaggagtggcaaaaggccttt

accgatgtgatgggcatggacgagctgtacaagtaaactagtagcagacagatgcgccgt

ggataaattgttataaaatttctatggtcaaaccgttgaagttgcctgattatttctggc

agccgaattactctagcagccacccgatccaatgcagttggcgcgccattcgatatcgga

ccctttccctttagtgagggttaatgct

>E2_s1_SynSno_pointMutU

acctttgccaagccaatggcggctaactatctgaagaaccagccgatgtacgtgttccgt

aagacggagctcaagcactccaagaccgagctcaacttcaaggagtggcaaaaggccttt

accgatgtgatgggcatggacgagctgtacaagtaaactagtagcagacagatgcgccgt

ggataaattgttatacactacctacggtctaacaatggaagttgcctgattatttctggc

agccgaatttactcctgagccacccgatccaatgcagttggcgcgccattcgatatcgga

ccctttccctttagtgagggttaatgct

>E2_s2_SynSno_pointMutU

acctttgccaagccaatggcggctaactatctgaagaaccagccgatgtacgtgttccgt

aagacggagctcaagcactccaagaccgagctcaacttcaaggagtggcaaaaggccttt

accgatgtgatgggcatggacgagctgtacaagtaaactagtagcagacagatgcgccgt

ggataaattgttataagaattactaggtagcgaaaacgaagttgcctgattatttctggc

agccgaatttacccttcagccacccgatccaatgcagttggcgcgccattcgatatcgga

ccctttccctttagtgagggttaatgct

>E3_SynSno_pointMutU

acctttgccaagccaatggcggctaactatctgaagaaccagccgatgtacgtgttccgt

aagacggagctcaagcactccaagaccgagctcaacttcaaggagtggcaaaaggccttt

accgatgtgatgggcatggacgagctgtacaagtaaactagtagcagacagatgcgccgt

ggataaattgttatagaccaagcgtggatagttaagagaagttgcctgattatttctggc

agccgaattcttgtcttagccacccgatccaatgcagttggcgcgccattcgatatcgga

ccctttccctttagtgagggttaatgct

>HBI-115_SynSno_pointMutU

acctttgccaagccaatggcggctaactatctgaagaaccagccgatgtacgtgttccgt

aagacggagctcaagcactccaagaccgagctcaacttcaaggagtggcaaaaggccttt

accgatgtgatgggcatggacgagctgtacaagtaaactagtagcagacagatgcgccgt

ggataaattgttataggtcttaaccggttctcagtccgaagttgcctgattatttctggc

agccgaattatccgtgtagccacccgatccaatgcagttggcgcgccattcgatatcgga

ccctttccctttagtgagggttaatgct

>HBI-6_SynSno_pointMutU

acctttgccaagccaatggcggctaactatctgaagaaccagccgatgtacgtgttccgt

aagacggagctcaagcactccaagaccgagctcaacttcaaggagtggcaaaaggccttt

accgatgtgatgggcatggacgagctgtacaagtaaactagtagcagacagatgcgccgt

ggataaattgttatacagacaggatgggttttatggagaagttgcctgattatttctggc

agccgaattaagcgtccagccacccgatccaatgcagttggcgcgccattcgatatcgga

ccctttccctttagtgagggttaatgct

>HBI-61_SynSno_pointMutU

acctttgccaagccaatggcggctaactatctgaagaaccagccgatgtacgtgttccgt

aagacggagctcaagcactccaagaccgagctcaacttcaaggagtggcaaaaggccttt

accgatgtgatgggcatggacgagctgtacaagtaaactagtagcagacagatgcgccgt

ggataaattgttatacttgtgtgtaggtgcttatacagaagttgcctgattatttctggc

agccgaattcccacggtagccacccgatccaatgcagttggcgcgccattcgatatcgga

ccctttccctttagtgagggttaatgct

>ACA1_SynSno_hyb10nt

acctttgccaagccaatggcggctaactatctgaagaaccagccgatgtacgtgttccgt

aagacggagctcaagcactccaagaccgagctcaacttcaaggagtggcaaaaggccttt

accgatgtgatgggcatggacgagctgtacaagtaaactagtagcagacagatgcgccgt

ggataaattgttataggtctcattgtgaagcataaccgaagttgcctgattatttctggc

agccgaatttcatacccagccacccgatccaatgcagttggcgcgccattcgatatcgga

ccctttccctttagtgagggttaatgct

>ACA10_s1_SynSno_hyb10nt

acctttgccaagccaatggcggctaactatctgaagaaccagccgatgtacgtgttccgt

aagacggagctcaagcactccaagaccgagctcaacttcaaggagtggcaaaaggccttt

accgatgtgatgggcatggacgagctgtacaagtaaactagtagcagacagatgcgccgt

ggataaattgttatacctgaaaacatgagctgagagagaagttgcctgattatttctggc

agccgaattgcattcgcagccacccgatccaatgcagttggcgcgccattcgatatcgga

ccctttccctttagtgagggttaatgct

>ACA10_s2_SynSno_hyb10nt

acctttgccaagccaatggcggctaactatctgaagaaccagccgatgtacgtgttccgt

aagacggagctcaagcactccaagaccgagctcaacttcaaggagtggcaaaaggccttt

accgatgtgatgggcatggacgagctgtacaagtaaactagtagcagacagatgcgccgt

ggataaattgttataactcctaggatgcgtgcattaggaagttgcctgattatttctggc

agccgaatttccgccggagccacccgatccaatgcagttggcgcgccattcgatatcgga

ccctttccctttagtgagggttaatgct

>ACA13_SynSno_hyb10nt

acctttgccaagccaatggcggctaactatctgaagaaccagccgatgtacgtgttccgt

aagacggagctcaagcactccaagaccgagctcaacttcaaggagtggcaaaaggccttt

accgatgtgatgggcatggacgagctgtacaagtaaactagtagcagacagatgcgccgt

ggataaattgttatacttttttgactgaacacaaagggaagttgcctgattatttctggc

agccgaattagaaatggagccacccgatccaatgcagttggcgcgccattcgatatcgga

ccctttccctttagtgagggttaatgct

>ACA14a_SynSno_hyb10nt

acctttgccaagccaatggcggctaactatctgaagaaccagccgatgtacgtgttccgt

aagacggagctcaagcactccaagaccgagctcaacttcaaggagtggcaaaaggccttt

accgatgtgatgggcatggacgagctgtacaagtaaactagtagcagacagatgcgccgt

ggataaattgttataggtatgaaattgttggactttcgaagttgcctgattatttctggc

agccgaattttaagtccagccacccgatccaatgcagttggcgcgccattcgatatcgga

ccctttccctttagtgagggttaatgct

>ACA15_SynSno_hyb10nt

acctttgccaagccaatggcggctaactatctgaagaaccagccgatgtacgtgttccgt

aagacggagctcaagcactccaagaccgagctcaacttcaaggagtggcaaaaggccttt

accgatgtgatgggcatggacgagctgtacaagtaaactagtagcagacagatgcgccgt

ggataaattgttatatggtgtctggtgtattcggccagaagttgcctgattatttctggc

agccgaattccaattccagccacccgatccaatgcagttggcgcgccattcgatatcgga

ccctttccctttagtgagggttaatgct

>ACA16_SynSno_hyb10nt

acctttgccaagccaatggcggctaactatctgaagaaccagccgatgtacgtgttccgt

aagacggagctcaagcactccaagaccgagctcaacttcaaggagtggcaaaaggccttt

accgatgtgatgggcatggacgagctgtacaagtaaactagtagcagacagatgcgccgt

ggataaattgttatatcctttttgatgcttcgataaggaagttgcctgattatttctggc

agccgaattaatcgtatagccacccgatccaatgcagttggcgcgccattcgatatcgga

ccctttccctttagtgagggttaatgct

>ACA17_s1_SynSno_hyb10nt

acctttgccaagccaatggcggctaactatctgaagaaccagccgatgtacgtgttccgt

aagacggagctcaagcactccaagaccgagctcaacttcaaggagtggcaaaaggccttt

accgatgtgatgggcatggacgagctgtacaagtaaactagtagcagacagatgcgccgt

ggataaattgttataacctaatgactgaacgcctctagaagttgcctgattatttctggc

agccgaattcagttcacagccacccgatccaatgcagttggcgcgccattcgatatcgga

ccctttccctttagtgagggttaatgct

>ACA17_s2_SynSno_hyb10nt

acctttgccaagccaatggcggctaactatctgaagaaccagccgatgtacgtgttccgt

aagacggagctcaagcactccaagaccgagctcaacttcaaggagtggcaaaaggccttt

accgatgtgatgggcatggacgagctgtacaagtaaactagtagcagacagatgcgccgt

ggataaattgttatatatcaaacattgagcgtagacagaagttgcctgattatttctggc

agccgaatttgcccgtcagccacccgatccaatgcagttggcgcgccattcgatatcgga

ccctttccctttagtgagggttaatgct

>ACA19_s1_SynSno_hyb10nt

acctttgccaagccaatggcggctaactatctgaagaaccagccgatgtacgtgttccgt

aagacggagctcaagcactccaagaccgagctcaacttcaaggagtggcaaaaggccttt

accgatgtgatgggcatggacgagctgtacaagtaaactagtagcagacagatgcgccgt

ggataaattgttatacataagaaattgaatgaaatgtgaagttgcctgattatttctggc

agccgaattaatcgctcagccacccgatccaatgcagttggcgcgccattcgatatcgga

ccctttccctttagtgagggttaatgct

>ACA19_s2_SynSno_hyb10nt

acctttgccaagccaatggcggctaactatctgaagaaccagccgatgtacgtgttccgt

aagacggagctcaagcactccaagaccgagctcaacttcaaggagtggcaaaaggccttt

accgatgtgatgggcatggacgagctgtacaagtaaactagtagcagacagatgcgccgt

ggataaattgttataaaggagactgtgtaattagcctgaagttgcctgattatttctggc

agccgaattggttcacaagccacccgatccaatgcagttggcgcgccattcgatatcgga

ccctttccctttagtgagggttaatgct

>ACA19_s3_SynSno_hyb10nt

acctttgccaagccaatggcggctaactatctgaagaaccagccgatgtacgtgttccgt

aagacggagctcaagcactccaagaccgagctcaacttcaaggagtggcaaaaggccttt

accgatgtgatgggcatggacgagctgtacaagtaaactagtagcagacagatgcgccgt

ggataaattgttatacacataagaatgatgaaatgtggaagttgcctgattatttctggc

agccgaattcaatgtgtagccacccgatccaatgcagttggcgcgccattcgatatcgga

ccctttccctttagtgagggttaatgct

>ACA20_SynSno_hyb10nt

acctttgccaagccaatggcggctaactatctgaagaaccagccgatgtacgtgttccgt

aagacggagctcaagcactccaagaccgagctcaacttcaaggagtggcaaaaggccttt

accgatgtgatgggcatggacgagctgtacaagtaaactagtagcagacagatgcgccgt

ggataaattgttatacatgcgtatatgaaataaatgggaagttgcctgattatttctggc

agccgaattacccggcgagccacccgatccaatgcagttggcgcgccattcgatatcgga

ccctttccctttagtgagggttaatgct

>ACA21_s1_SynSno_hyb10nt

acctttgccaagccaatggcggctaactatctgaagaaccagccgatgtacgtgttccgt

aagacggagctcaagcactccaagaccgagctcaacttcaaggagtggcaaaaggccttt

accgatgtgatgggcatggacgagctgtacaagtaaactagtagcagacagatgcgccgt

ggataaattgttatatttcttgacgtggcttttaaaagaagttgcctgattatttctggc

agccgaattccagtgaaagccacccgatccaatgcagttggcgcgccattcgatatcgga

ccctttccctttagtgagggttaatgct

>ACA21_s2_SynSno_hyb10nt

acctttgccaagccaatggcggctaactatctgaagaaccagccgatgtacgtgttccgt

aagacggagctcaagcactccaagaccgagctcaacttcaaggagtggcaaaaggccttt

accgatgtgatgggcatggacgagctgtacaagtaaactagtagcagacagatgcgccgt

ggataaattgttataccaccgattgtgcacccaaaaggaagttgcctgattatttctggc

agccgaattatgttatcagccacccgatccaatgcagttggcgcgccattcgatatcgga

ccctttccctttagtgagggttaatgct

>ACA22_s1_SynSno_hyb10nt

acctttgccaagccaatggcggctaactatctgaagaaccagccgatgtacgtgttccgt

aagacggagctcaagcactccaagaccgagctcaacttcaaggagtggcaaaaggccttt

accgatgtgatgggcatggacgagctgtacaagtaaactagtagcagacagatgcgccgt

ggataaattgttataagggtcaaagtgtcactgtgcagaagttgcctgattatttctggc

agccgaattccaaacgaagccacccgatccaatgcagttggcgcgccattcgatatcgga

ccctttccctttagtgagggttaatgct

>ACA22_s2_SynSno_hyb10nt

acctttgccaagccaatggcggctaactatctgaagaaccagccgatgtacgtgttccgt

aagacggagctcaagcactccaagaccgagctcaacttcaaggagtggcaaaaggccttt

accgatgtgatgggcatggacgagctgtacaagtaaactagtagcagacagatgcgccgt

ggataaattgttatatctgaatacatggcagaggagagaagttgcctgattatttctggc

agccgaattgtagcgatagccacccgatccaatgcagttggcgcgccattcgatatcgga

ccctttccctttagtgagggttaatgct

>ACA23_s1_SynSno_hyb10nt

acctttgccaagccaatggcggctaactatctgaagaaccagccgatgtacgtgttccgt

aagacggagctcaagcactccaagaccgagctcaacttcaaggagtggcaaaaggccttt

accgatgtgatgggcatggacgagctgtacaagtaaactagtagcagacagatgcgccgt

ggataaattgttatacatgatgttttgagcagccatggaagttgcctgattatttctggc

agccgaattgaggactcagccacccgatccaatgcagttggcgcgccattcgatatcgga

ccctttccctttagtgagggttaatgct

>ACA23_s2_SynSno_hyb10nt

acctttgccaagccaatggcggctaactatctgaagaaccagccgatgtacgtgttccgt

aagacggagctcaagcactccaagaccgagctcaacttcaaggagtggcaaaaggccttt

accgatgtgatgggcatggacgagctgtacaagtaaactagtagcagacagatgcgccgt

ggataaattgttatagaatttggagtgactatgatccgaagttgcctgattatttctggc

agccgaattaatagtttagccacccgatccaatgcagttggcgcgccattcgatatcgga

ccctttccctttagtgagggttaatgct

>ACA24_s1_SynSno_hyb10nt

acctttgccaagccaatggcggctaactatctgaagaaccagccgatgtacgtgttccgt

aagacggagctcaagcactccaagaccgagctcaacttcaaggagtggcaaaaggccttt

accgatgtgatgggcatggacgagctgtacaagtaaactagtagcagacagatgcgccgt

ggataaattgttatatggctaggaatgaaagatacatgaagttgcctgattatttctggc

agccgaattaagtcctaagccacccgatccaatgcagttggcgcgccattcgatatcgga

ccctttccctttagtgagggttaatgct

>ACA24_s2_SynSno_hyb10nt

acctttgccaagccaatggcggctaactatctgaagaaccagccgatgtacgtgttccgt

aagacggagctcaagcactccaagaccgagctcaacttcaaggagtggcaaaaggccttt

accgatgtgatgggcatggacgagctgtacaagtaaactagtagcagacagatgcgccgt

ggataaattgttatatgtgcaagtctgttgccaataagaagttgcctgattatttctggc

agccgaattcctagctaagccacccgatccaatgcagttggcgcgccattcgatatcgga

ccctttccctttagtgagggttaatgct

>ACA25_s1_SynSno_hyb10nt

acctttgccaagccaatggcggctaactatctgaagaaccagccgatgtacgtgttccgt

aagacggagctcaagcactccaagaccgagctcaacttcaaggagtggcaaaaggccttt

accgatgtgatgggcatggacgagctgtacaagtaaactagtagcagacagatgcgccgt

ggataaattgttatatcacagcgtttgctttgaaatggaagttgcctgattatttctggc

agccgaattataaacgtagccacccgatccaatgcagttggcgcgccattcgatatcgga

ccctttccctttagtgagggttaatgct

>ACA25_s2_SynSno_hyb10nt

acctttgccaagccaatggcggctaactatctgaagaaccagccgatgtacgtgttccgt

aagacggagctcaagcactccaagaccgagctcaacttcaaggagtggcaaaaggccttt

accgatgtgatgggcatggacgagctgtacaagtaaactagtagcagacagatgcgccgt

ggataaattgttatactcaggaaaatgggagtgctatgaagttgcctgattatttctggc

agccgaatttgtcaatcagccacccgatccaatgcagttggcgcgccattcgatatcgga

ccctttccctttagtgagggttaatgct

>ACA27_s1_SynSno_hyb10nt

acctttgccaagccaatggcggctaactatctgaagaaccagccgatgtacgtgttccgt

aagacggagctcaagcactccaagaccgagctcaacttcaaggagtggcaaaaggccttt

accgatgtgatgggcatggacgagctgtacaagtaaactagtagcagacagatgcgccgt

ggataaattgttatacacttgaatgtgaaagtgaaaagaagttgcctgattatttctggc

agccgaattttattcagagccacccgatccaatgcagttggcgcgccattcgatatcgga

ccctttccctttagtgagggttaatgct

>ACA27_s2_SynSno_hyb10nt

acctttgccaagccaatggcggctaactatctgaagaaccagccgatgtacgtgttccgt

aagacggagctcaagcactccaagaccgagctcaacttcaaggagtggcaaaaggccttt

accgatgtgatgggcatggacgagctgtacaagtaaactagtagcagacagatgcgccgt

ggataaattgttatatctgacaggatggttttaaaaggaagttgcctgattatttctggc

agccgaattcaggagacagccacccgatccaatgcagttggcgcgccattcgatatcgga

ccctttccctttagtgagggttaatgct

>ACA28_s1_SynSno_hyb10nt

acctttgccaagccaatggcggctaactatctgaagaaccagccgatgtacgtgttccgt

aagacggagctcaagcactccaagaccgagctcaacttcaaggagtggcaaaaggccttt

accgatgtgatgggcatggacgagctgtacaagtaaactagtagcagacagatgcgccgt

ggataaattgttataaagctcaaattggagtgttgctgaagttgcctgattatttctggc

agccgaattctgatctgagccacccgatccaatgcagttggcgcgccattcgatatcgga

ccctttccctttagtgagggttaatgct

>ACA28_s2_SynSno_hyb10nt

acctttgccaagccaatggcggctaactatctgaagaaccagccgatgtacgtgttccgt

aagacggagctcaagcactccaagaccgagctcaacttcaaggagtggcaaaaggccttt

accgatgtgatgggcatggacgagctgtacaagtaaactagtagcagacagatgcgccgt

ggataaattgttatagtctatataatggaataggttagaagttgcctgattatttctggc

agccgaattagtgttccagccacccgatccaatgcagttggcgcgccattcgatatcgga

ccctttccctttagtgagggttaatgct

>ACA2a_s1_SynSno_hyb10nt

acctttgccaagccaatggcggctaactatctgaagaaccagccgatgtacgtgttccgt

aagacggagctcaagcactccaagaccgagctcaacttcaaggagtggcaaaaggccttt

accgatgtgatgggcatggacgagctgtacaagtaaactagtagcagacagatgcgccgt

ggataaattgttatatcctgtttgatgttgattcagggaagttgcctgattatttctggc

agccgaattcttctccgagccacccgatccaatgcagttggcgcgccattcgatatcgga

ccctttccctttagtgagggttaatgct

>ACA2a_s2_SynSno_hyb10nt

acctttgccaagccaatggcggctaactatctgaagaaccagccgatgtacgtgttccgt

aagacggagctcaagcactccaagaccgagctcaacttcaaggagtggcaaaaggccttt

accgatgtgatgggcatggacgagctgtacaagtaaactagtagcagacagatgcgccgt

ggataaattgttatatccaactgaatgcagaccacaagaagttgcctgattatttctggc

agccgaattgccagttaagccacccgatccaatgcagttggcgcgccattcgatatcgga

ccctttccctttagtgagggttaatgct

>ACA3_s1_SynSno_hyb10nt

acctttgccaagccaatggcggctaactatctgaagaaccagccgatgtacgtgttccgt

aagacggagctcaagcactccaagaccgagctcaacttcaaggagtggcaaaaggccttt

accgatgtgatgggcatggacgagctgtacaagtaaactagtagcagacagatgcgccgt

ggataaattgttataggactctagctggactctagccgaagttgcctgattatttctggc

agccgaattataactgaagccacccgatccaatgcagttggcgcgccattcgatatcgga

ccctttccctttagtgagggttaatgct

>ACA3_s2_SynSno_hyb10nt

acctttgccaagccaatggcggctaactatctgaagaaccagccgatgtacgtgttccgt

aagacggagctcaagcactccaagaccgagctcaacttcaaggagtggcaaaaggccttt

accgatgtgatgggcatggacgagctgtacaagtaaactagtagcagacagatgcgccgt

ggataaattgttatataacctggtgtggaataaggtcgaagttgcctgattatttctggc

agccgaattgagaggcaagccacccgatccaatgcagttggcgcgccattcgatatcgga

ccctttccctttagtgagggttaatgct

>ACA3-2_SynSno_hyb10nt

acctttgccaagccaatggcggctaactatctgaagaaccagccgatgtacgtgttccgt

aagacggagctcaagcactccaagaccgagctcaacttcaaggagtggcaaaaggccttt

accgatgtgatgggcatggacgagctgtacaagtaaactagtagcagacagatgcgccgt

ggataaattgttatagtactctagctggactctagtcgaagttgcctgattatttctggc

agccgaattacttgaatagccacccgatccaatgcagttggcgcgccattcgatatcgga

ccctttccctttagtgagggttaatgct

>ACA30_SynSno_hyb10nt

acctttgccaagccaatggcggctaactatctgaagaaccagccgatgtacgtgttccgt

aagacggagctcaagcactccaagaccgagctcaacttcaaggagtggcaaaaggccttt

accgatgtgatgggcatggacgagctgtacaagtaaactagtagcagacagatgcgccgt

ggataaattgttataccagctaccatgtgtgaaagtggaagttgcctgattatttctggc

agccgaattcgctcagcagccacccgatccaatgcagttggcgcgccattcgatatcgga

ccctttccctttagtgagggttaatgct

>ACA31_s1_SynSno_hyb10nt

acctttgccaagccaatggcggctaactatctgaagaaccagccgatgtacgtgttccgt

aagacggagctcaagcactccaagaccgagctcaacttcaaggagtggcaaaaggccttt

accgatgtgatgggcatggacgagctgtacaagtaaactagtagcagacagatgcgccgt

ggataaattgttatatcctagtgcatgtatcagtggagaagttgcctgattatttctggc

agccgaatttgcacaggagccacccgatccaatgcagttggcgcgccattcgatatcgga

ccctttccctttagtgagggttaatgct

>ACA31_s2_SynSno_hyb10nt

acctttgccaagccaatggcggctaactatctgaagaaccagccgatgtacgtgttccgt

aagacggagctcaagcactccaagaccgagctcaacttcaaggagtggcaaaaggccttt

accgatgtgatgggcatggacgagctgtacaagtaaactagtagcagacagatgcgccgt

ggataaattgttatacccaattcaatgaagcgcaggggaagttgcctgattatttctggc

agccgaattcataatacagccacccgatccaatgcagttggcgcgccattcgatatcgga

ccctttccctttagtgagggttaatgct

>ACA32_SynSno_hyb10nt

acctttgccaagccaatggcggctaactatctgaagaaccagccgatgtacgtgttccgt

aagacggagctcaagcactccaagaccgagctcaacttcaaggagtggcaaaaggccttt

accgatgtgatgggcatggacgagctgtacaagtaaactagtagcagacagatgcgccgt

ggataaattgttatatcatgtccactgttggtaatgagaagttgcctgattatttctggc

agccgaattgacggactagccacccgatccaatgcagttggcgcgccattcgatatcgga

ccctttccctttagtgagggttaatgct

>ACA33_SynSno_hyb10nt

acctttgccaagccaatggcggctaactatctgaagaaccagccgatgtacgtgttccgt

aagacggagctcaagcactccaagaccgagctcaacttcaaggagtggcaaaaggccttt

accgatgtgatgggcatggacgagctgtacaagtaaactagtagcagacagatgcgccgt

ggataaattgttataattctcagggtgtcgtaacatggaagttgcctgattatttctggc

agccgaattgactgtgaagccacccgatccaatgcagttggcgcgccattcgatatcgga

ccctttccctttagtgagggttaatgct

>ACA34_s1_SynSno_hyb10nt

acctttgccaagccaatggcggctaactatctgaagaaccagccgatgtacgtgttccgt

aagacggagctcaagcactccaagaccgagctcaacttcaaggagtggcaaaaggccttt

accgatgtgatgggcatggacgagctgtacaagtaaactagtagcagacagatgcgccgt

ggataaattgttatactctgcttgatgttcagtcagggaagttgcctgattatttctggc

agccgaattaacggtgtagccacccgatccaatgcagttggcgcgccattcgatatcgga

ccctttccctttagtgagggttaatgct

>ACA34_s2_SynSno_hyb10nt

acctttgccaagccaatggcggctaactatctgaagaaccagccgatgtacgtgttccgt

aagacggagctcaagcactccaagaccgagctcaacttcaaggagtggcaaaaggccttt

accgatgtgatgggcatggacgagctgtacaagtaaactagtagcagacagatgcgccgt

ggataaattgttatatccaaatgaatgcagaccacaggaagttgcctgattatttctggc

agccgaattagaggggaagccacccgatccaatgcagttggcgcgccattcgatatcgga

ccctttccctttagtgagggttaatgct

>ACA36_s1_SynSno_hyb10nt

acctttgccaagccaatggcggctaactatctgaagaaccagccgatgtacgtgttccgt

aagacggagctcaagcactccaagaccgagctcaacttcaaggagtggcaaaaggccttt

accgatgtgatgggcatggacgagctgtacaagtaaactagtagcagacagatgcgccgt

ggataaattgttataaagtttaatttgactcaacactgaagttgcctgattatttctggc

agccgaattttatctccagccacccgatccaatgcagttggcgcgccattcgatatcgga

ccctttccctttagtgagggttaatgct

>ACA36_s2_SynSno_hyb10nt

acctttgccaagccaatggcggctaactatctgaagaaccagccgatgtacgtgttccgt

aagacggagctcaagcactccaagaccgagctcaacttcaaggagtggcaaaaggccttt

accgatgtgatgggcatggacgagctgtacaagtaaactagtagcagacagatgcgccgt

ggataaattgttatagggaggcaaatgaatcatctccgaagttgcctgattatttctggc

agccgaattcagagcaaagccacccgatccaatgcagttggcgcgccattcgatatcgga

ccctttccctttagtgagggttaatgct

>ACA4_SynSno_hyb10nt

acctttgccaagccaatggcggctaactatctgaagaaccagccgatgtacgtgttccgt

aagacggagctcaagcactccaagaccgagctcaacttcaaggagtggcaaaaggccttt

accgatgtgatgggcatggacgagctgtacaagtaaactagtagcagacagatgcgccgt

ggataaattgttataacaaaatagttggtggagtgtggaagttgcctgattatttctggc

agccgaattgtgcggtgagccacccgatccaatgcagttggcgcgccattcgatatcgga

ccctttccctttagtgagggttaatgct

>ACA40_SynSno_hyb10nt

acctttgccaagccaatggcggctaactatctgaagaaccagccgatgtacgtgttccgt

aagacggagctcaagcactccaagaccgagctcaacttcaaggagtggcaaaaggccttt

accgatgtgatgggcatggacgagctgtacaagtaaactagtagcagacagatgcgccgt

ggataaattgttatacttatatgtatggttgcaaaaggaagttgcctgattatttctggc

agccgaatttactcacaagccacccgatccaatgcagttggcgcgccattcgatatcgga

ccctttccctttagtgagggttaatgct

>ACA41_SynSno_hyb10nt

acctttgccaagccaatggcggctaactatctgaagaaccagccgatgtacgtgttccgt

aagacggagctcaagcactccaagaccgagctcaacttcaaggagtggcaaaaggccttt

accgatgtgatgggcatggacgagctgtacaagtaaactagtagcagacagatgcgccgt

ggataaattgttataacagaggaattgccagtagctggaagttgcctgattatttctggc

agccgaattaatccagaagccacccgatccaatgcagttggcgcgccattcgatatcgga

ccctttccctttagtgagggttaatgct

>ACA42_s1_SynSno_hyb10nt

acctttgccaagccaatggcggctaactatctgaagaaccagccgatgtacgtgttccgt

aagacggagctcaagcactccaagaccgagctcaacttcaaggagtggcaaaaggccttt

accgatgtgatgggcatggacgagctgtacaagtaaactagtagcagacagatgcgccgt

ggataaattgttatatgggtacacttgaaatccattagaagttgcctgattatttctggc

agccgaatttcgaggagagccacccgatccaatgcagttggcgcgccattcgatatcgga

ccctttccctttagtgagggttaatgct

>ACA42_s2_SynSno_hyb10nt

acctttgccaagccaatggcggctaactatctgaagaaccagccgatgtacgtgttccgt

aagacggagctcaagcactccaagaccgagctcaacttcaaggagtggcaaaaggccttt

accgatgtgatgggcatggacgagctgtacaagtaaactagtagcagacagatgcgccgt

ggataaattgttatacccccttcagtgatggttcgaggaagttgcctgattatttctggc

agccgaattgactcaaaagccacccgatccaatgcagttggcgcgccattcgatatcgga

ccctttccctttagtgagggttaatgct

>ACA43_SynSno_hyb10nt

acctttgccaagccaatggcggctaactatctgaagaaccagccgatgtacgtgttccgt

aagacggagctcaagcactccaagaccgagctcaacttcaaggagtggcaaaaggccttt

accgatgtgatgggcatggacgagctgtacaagtaaactagtagcagacagatgcgccgt

ggataaattgttatacataaaccattggtagaaatcggaagttgcctgattatttctggc

agccgaatttgctccacagccacccgatccaatgcagttggcgcgccattcgatatcgga

ccctttccctttagtgagggttaatgct

>ACA44_s1_SynSno_hyb10nt

acctttgccaagccaatggcggctaactatctgaagaaccagccgatgtacgtgttccgt

aagacggagctcaagcactccaagaccgagctcaacttcaaggagtggcaaaaggccttt

accgatgtgatgggcatggacgagctgtacaagtaaactagtagcagacagatgcgccgt

ggataaattgttatatgcagttggatgttggaaacatgaagttgcctgattatttctggc

agccgaattgccgcatcagccacccgatccaatgcagttggcgcgccattcgatatcgga

ccctttccctttagtgagggttaatgct

>ACA44_s2_SynSno_hyb10nt

acctttgccaagccaatggcggctaactatctgaagaaccagccgatgtacgtgttccgt

aagacggagctcaagcactccaagaccgagctcaacttcaaggagtggcaaaaggccttt

accgatgtgatgggcatggacgagctgtacaagtaaactagtagcagacagatgcgccgt

ggataaattgttataaagctgagtgtgcaaagtctttgaagttgcctgattatttctggc

agccgaattaactagaaagccacccgatccaatgcagttggcgcgccattcgatatcgga

ccctttccctttagtgagggttaatgct

>ACA46_SynSno_hyb10nt

acctttgccaagccaatggcggctaactatctgaagaaccagccgatgtacgtgttccgt

aagacggagctcaagcactccaagaccgagctcaacttcaaggagtggcaaaaggccttt

accgatgtgatgggcatggacgagctgtacaagtaaactagtagcagacagatgcgccgt

ggataaattgttatactacagcgtatgttaaatataggaagttgcctgattatttctggc

agccgaattgagtcgcaagccacccgatccaatgcagttggcgcgccattcgatatcgga

ccctttccctttagtgagggttaatgct

>ACA48_SynSno_hyb10nt

acctttgccaagccaatggcggctaactatctgaagaaccagccgatgtacgtgttccgt

aagacggagctcaagcactccaagaccgagctcaacttcaaggagtggcaaaaggccttt

accgatgtgatgggcatggacgagctgtacaagtaaactagtagcagacagatgcgccgt

ggataaattgttataaaggcatgaatggatcaaaagcgaagttgcctgattatttctggc

agccgaattggagcgacagccacccgatccaatgcagttggcgcgccattcgatatcgga

ccctttccctttagtgagggttaatgct

>U19_up_SynSno_hyb10nt

acctttgccaagccaatggcggctaactatctgaagaaccagccgatgtacgtgttccgt

aagacggagctcaagcactccaagaccgagctcaacttcaaggagtggcaaaaggccttt

accgatgtgatgggcatggacgagctgtacaagtaaactagtagcagacagatgcgccgt

ggataaattgttataacagtgtaactgtgacaaccgcgaagttgcctgattatttctggc

agccgaattcacgaggtagccacccgatccaatgcagttggcgcgccattcgatatcgga

ccctttccctttagtgagggttaatgct

>U19_dn_SynSno_hyb10nt

acctttgccaagccaatggcggctaactatctgaagaaccagccgatgtacgtgttccgt

aagacggagctcaagcactccaagaccgagctcaacttcaaggagtggcaaaaggccttt

accgatgtgatgggcatggacgagctgtacaagtaaactagtagcagacagatgcgccgt

ggataaattgttataacccagactatgctctcagagggaagttgcctgattatttctggc

agccgaattttttcatcagccacccgatccaatgcagttggcgcgccattcgatatcgga

ccctttccctttagtgagggttaatgct

>U23_up_SynSno_hyb10nt

acctttgccaagccaatggcggctaactatctgaagaaccagccgatgtacgtgttccgt

aagacggagctcaagcactccaagaccgagctcaacttcaaggagtggcaaaaggccttt

accgatgtgatgggcatggacgagctgtacaagtaaactagtagcagacagatgcgccgt

ggataaattgttatactcgtgcgaatggctcaatgaggaagttgcctgattatttctggc

agccgaattttaggtcgagccacccgatccaatgcagttggcgcgccattcgatatcgga

ccctttccctttagtgagggttaatgct

>U64_up_SynSno_hyb10nt

acctttgccaagccaatggcggctaactatctgaagaaccagccgatgtacgtgttccgt

aagacggagctcaagcactccaagaccgagctcaacttcaaggagtggcaaaaggccttt

accgatgtgatgggcatggacgagctgtacaagtaaactagtagcagacagatgcgccgt

ggataaattgttataccccgttacgtggcagagccgagaagttgcctgattatttctggc

agccgaattacgcagatagccacccgatccaatgcagttggcgcgccattcgatatcgga

ccctttccctttagtgagggttaatgct

>U65_up_SynSno_hyb10nt

acctttgccaagccaatggcggctaactatctgaagaaccagccgatgtacgtgttccgt

aagacggagctcaagcactccaagaccgagctcaacttcaaggagtggcaaaaggccttt

accgatgtgatgggcatggacgagctgtacaagtaaactagtagcagacagatgcgccgt

ggataaattgttatacaccactggatggtggcgggtggaagttgcctgattatttctggc

agccgaattatttgtaaagccacccgatccaatgcagttggcgcgccattcgatatcgga

ccctttccctttagtgagggttaatgct

>U65_dn_SynSno_hyb10nt

acctttgccaagccaatggcggctaactatctgaagaaccagccgatgtacgtgttccgt

aagacggagctcaagcactccaagaccgagctcaacttcaaggagtggcaaaaggccttt

accgatgtgatgggcatggacgagctgtacaagtaaactagtagcagacagatgcgccgt

ggataaattgttatatgctttcggctgttcctaagctgaagttgcctgattatttctggc

agccgaattgcgctaacagccacccgatccaatgcagttggcgcgccattcgatatcgga

ccctttccctttagtgagggttaatgct

>U66_up_SynSno_hyb10nt

acctttgccaagccaatggcggctaactatctgaagaaccagccgatgtacgtgttccgt

aagacggagctcaagcactccaagaccgagctcaacttcaaggagtggcaaaaggccttt

accgatgtgatgggcatggacgagctgtacaagtaaactagtagcagacagatgcgccgt

ggataaattgttataacctggttcctgtgatcgagttgaagttgcctgattatttctggc

agccgaattgtctcaagagccacccgatccaatgcagttggcgcgccattcgatatcgga

ccctttccctttagtgagggttaatgct

>U67_up_SynSno_hyb10nt

acctttgccaagccaatggcggctaactatctgaagaaccagccgatgtacgtgttccgt

aagacggagctcaagcactccaagaccgagctcaacttcaaggagtggcaaaaggccttt

accgatgtgatgggcatggacgagctgtacaagtaaactagtagcagacagatgcgccgt

ggataaattgttataagctgctacttggagggaatcagaagttgcctgattatttctggc

agccgaattattcattgagccacccgatccaatgcagttggcgcgccattcgatatcgga

ccctttccctttagtgagggttaatgct

>U68_up_SynSno_hyb10nt

acctttgccaagccaatggcggctaactatctgaagaaccagccgatgtacgtgttccgt

aagacggagctcaagcactccaagaccgagctcaacttcaaggagtggcaaaaggccttt

accgatgtgatgggcatggacgagctgtacaagtaaactagtagcagacagatgcgccgt

ggataaattgttatatccaaattcatggcgacaagatgaagttgcctgattatttctggc

agccgaattcggacagcagccacccgatccaatgcagttggcgcgccattcgatatcgga

ccctttccctttagtgagggttaatgct

>U69_up_SynSno_hyb10nt

acctttgccaagccaatggcggctaactatctgaagaaccagccgatgtacgtgttccgt

aagacggagctcaagcactccaagaccgagctcaacttcaaggagtggcaaaaggccttt

accgatgtgatgggcatggacgagctgtacaagtaaactagtagcagacagatgcgccgt

ggataaattgttatacaggataatgtgaattgcaaccgaagttgcctgattatttctggc

agccgaattcacttcttagccacccgatccaatgcagttggcgcgccattcgatatcgga

ccctttccctttagtgagggttaatgct

>U69_dn_SynSno_hyb10nt

acctttgccaagccaatggcggctaactatctgaagaaccagccgatgtacgtgttccgt

aagacggagctcaagcactccaagaccgagctcaacttcaaggagtggcaaaaggccttt

accgatgtgatgggcatggacgagctgtacaagtaaactagtagcagacagatgcgccgt

ggataaattgttatatcaatctgtctgaaaaatgattgaagttgcctgattatttctggc

agccgaattgtgaccttagccacccgatccaatgcagttggcgcgccattcgatatcgga

ccctttccctttagtgagggttaatgct

>U70_up_SynSno_hyb10nt

acctttgccaagccaatggcggctaactatctgaagaaccagccgatgtacgtgttccgt

aagacggagctcaagcactccaagaccgagctcaacttcaaggagtggcaaaaggccttt

accgatgtgatgggcatggacgagctgtacaagtaaactagtagcagacagatgcgccgt

ggataaattgttataaaggtccctttgtacactaccagaagttgcctgattatttctggc

agccgaattgctcgagcagccacccgatccaatgcagttggcgcgccattcgatatcgga

ccctttccctttagtgagggttaatgct

>U71a_up_SynSno_hyb10nt

acctttgccaagccaatggcggctaactatctgaagaaccagccgatgtacgtgttccgt

aagacggagctcaagcactccaagaccgagctcaacttcaaggagtggcaaaaggccttt

accgatgtgatgggcatggacgagctgtacaagtaaactagtagcagacagatgcgccgt

ggataaattgttataaagcttcaggtgacggggacaagaagttgcctgattatttctggc

agccgaattatcaggggagccacccgatccaatgcagttggcgcgccattcgatatcgga

ccctttccctttagtgagggttaatgct

>U72_up_SynSno_hyb10nt

acctttgccaagccaatggcggctaactatctgaagaaccagccgatgtacgtgttccgt

aagacggagctcaagcactccaagaccgagctcaacttcaaggagtggcaaaaggccttt

accgatgtgatgggcatggacgagctgtacaagtaaactagtagcagacagatgcgccgt

ggataaattgttataaatgtttagctgcgagaatattgaagttgcctgattatttctggc

agccgaattaacaatgcagccacccgatccaatgcagttggcgcgccattcgatatcgga

ccctttccctttagtgagggttaatgct

>U99_up_SynSno_hyb10nt

acctttgccaagccaatggcggctaactatctgaagaaccagccgatgtacgtgttccgt

aagacggagctcaagcactccaagaccgagctcaacttcaaggagtggcaaaaggccttt

accgatgtgatgggcatggacgagctgtacaagtaaactagtagcagacagatgcgccgt

ggataaattgttatatccgtacatttgccaagaaaaggaagttgcctgattatttctggc

agccgaattaaatccgaagccacccgatccaatgcagttggcgcgccattcgatatcgga

ccctttccctttagtgagggttaatgct

>ACA5_s1_SynSno_hyb10nt

acctttgccaagccaatggcggctaactatctgaagaaccagccgatgtacgtgttccgt

aagacggagctcaagcactccaagaccgagctcaacttcaaggagtggcaaaaggccttt

accgatgtgatgggcatggacgagctgtacaagtaaactagtagcagacagatgcgccgt

ggataaattgttataccttctgggctgaatttgacacgaagttgcctgattatttctggc

agccgaattgtagaagcagccacccgatccaatgcagttggcgcgccattcgatatcgga

ccctttccctttagtgagggttaatgct

>ACA5_s2_SynSno_hyb10nt

acctttgccaagccaatggcggctaactatctgaagaaccagccgatgtacgtgttccgt

aagacggagctcaagcactccaagaccgagctcaacttcaaggagtggcaaaaggccttt

accgatgtgatgggcatggacgagctgtacaagtaaactagtagcagacagatgcgccgt

ggataaattgttataccaaatttattgcccatgagtcgaagttgcctgattatttctggc

agccgaatttatctgtcagccacccgatccaatgcagttggcgcgccattcgatatcgga

ccctttccctttagtgagggttaatgct

>ACA50_s1_SynSno_hyb10nt

acctttgccaagccaatggcggctaactatctgaagaaccagccgatgtacgtgttccgt

aagacggagctcaagcactccaagaccgagctcaacttcaaggagtggcaaaaggccttt

accgatgtgatgggcatggacgagctgtacaagtaaactagtagcagacagatgcgccgt

ggataaattgttatactgttgcttgtgtcaaaggcaggaagttgcctgattatttctggc

agccgaattgcaactatagccacccgatccaatgcagttggcgcgccattcgatatcgga

ccctttccctttagtgagggttaatgct

>ACA50_s2_SynSno_hyb10nt

acctttgccaagccaatggcggctaactatctgaagaaccagccgatgtacgtgttccgt

aagacggagctcaagcactccaagaccgagctcaacttcaaggagtggcaaaaggccttt

accgatgtgatgggcatggacgagctgtacaagtaaactagtagcagacagatgcgccgt

ggataaattgttatatgtttttaaatgagttataacagaagttgcctgattatttctggc

agccgaattaatcacggagccacccgatccaatgcagttggcgcgccattcgatatcgga

ccctttccctttagtgagggttaatgct

>ACA52_SynSno_hyb10nt

acctttgccaagccaatggcggctaactatctgaagaaccagccgatgtacgtgttccgt

aagacggagctcaagcactccaagaccgagctcaacttcaaggagtggcaaaaggccttt

accgatgtgatgggcatggacgagctgtacaagtaaactagtagcagacagatgcgccgt

ggataaattgttatacacaagcgaatgattaggatgggaagttgcctgattatttctggc

agccgaattctaacagtagccacccgatccaatgcagttggcgcgccattcgatatcgga

ccctttccctttagtgagggttaatgct

>ACA54_s1_SynSno_hyb10nt

acctttgccaagccaatggcggctaactatctgaagaaccagccgatgtacgtgttccgt

aagacggagctcaagcactccaagaccgagctcaacttcaaggagtggcaaaaggccttt

accgatgtgatgggcatggacgagctgtacaagtaaactagtagcagacagatgcgccgt

ggataaattgttataccggaatggatgtacgaacagtgaagttgcctgattatttctggc

agccgaattgggcccagagccacccgatccaatgcagttggcgcgccattcgatatcgga

ccctttccctttagtgagggttaatgct

>ACA54_s2_SynSno_hyb10nt

acctttgccaagccaatggcggctaactatctgaagaaccagccgatgtacgtgttccgt

aagacggagctcaagcactccaagaccgagctcaacttcaaggagtggcaaaaggccttt

accgatgtgatgggcatggacgagctgtacaagtaaactagtagcagacagatgcgccgt

ggataaattgttatactgaaactgatgatgtgtcgctgaagttgcctgattatttctggc

agccgaattaccggaatagccacccgatccaatgcagttggcgcgccattcgatatcgga

ccctttccctttagtgagggttaatgct

>ACA55_SynSno_hyb10nt

acctttgccaagccaatggcggctaactatctgaagaaccagccgatgtacgtgttccgt

aagacggagctcaagcactccaagaccgagctcaacttcaaggagtggcaaaaggccttt

accgatgtgatgggcatggacgagctgtacaagtaaactagtagcagacagatgcgccgt

ggataaattgttataaccatctgtctgaaagattcaggaagttgcctgattatttctggc

agccgaattacaattccagccacccgatccaatgcagttggcgcgccattcgatatcgga

ccctttccctttagtgagggttaatgct

>ACA56_SynSno_hyb10nt

acctttgccaagccaatggcggctaactatctgaagaaccagccgatgtacgtgttccgt

aagacggagctcaagcactccaagaccgagctcaacttcaaggagtggcaaaaggccttt

accgatgtgatgggcatggacgagctgtacaagtaaactagtagcagacagatgcgccgt

ggataaattgttataactgactccctggggataactggaagttgcctgattatttctggc

agccgaatttaagatcaagccacccgatccaatgcagttggcgcgccattcgatatcgga

ccctttccctttagtgagggttaatgct

>ACA58_SynSno_hyb10nt

acctttgccaagccaatggcggctaactatctgaagaaccagccgatgtacgtgttccgt

aagacggagctcaagcactccaagaccgagctcaacttcaaggagtggcaaaaggccttt

accgatgtgatgggcatggacgagctgtacaagtaaactagtagcagacagatgcgccgt

ggataaattgttataacactgtccctgtctacgagtagaagttgcctgattatttctggc

agccgaattgataattcagccacccgatccaatgcagttggcgcgccattcgatatcgga

ccctttccctttagtgagggttaatgct

>ACA6_SynSno_hyb10nt

acctttgccaagccaatggcggctaactatctgaagaaccagccgatgtacgtgttccgt

aagacggagctcaagcactccaagaccgagctcaacttcaaggagtggcaaaaggccttt

accgatgtgatgggcatggacgagctgtacaagtaaactagtagcagacagatgcgccgt

ggataaattgttatacacttccgactgtttaatagtggaagttgcctgattatttctggc

agccgaatttaagtttcagccacccgatccaatgcagttggcgcgccattcgatatcgga

ccctttccctttagtgagggttaatgct

>ACA60_SynSno_hyb10nt

acctttgccaagccaatggcggctaactatctgaagaaccagccgatgtacgtgttccgt

aagacggagctcaagcactccaagaccgagctcaacttcaaggagtggcaaaaggccttt

accgatgtgatgggcatggacgagctgtacaagtaaactagtagcagacagatgcgccgt

ggataaattgttatacgccagcatttgccaaacacgggaagttgcctgattatttctggc

agccgaattaaaggcttagccacccgatccaatgcagttggcgcgccattcgatatcgga

ccctttccctttagtgagggttaatgct

>ACA61_SynSno_hyb10nt

acctttgccaagccaatggcggctaactatctgaagaaccagccgatgtacgtgttccgt

aagacggagctcaagcactccaagaccgagctcaacttcaaggagtggcaaaaggccttt

accgatgtgatgggcatggacgagctgtacaagtaaactagtagcagacagatgcgccgt

ggataaattgttataccttttacgatggaaagggatcgaagttgcctgattatttctggc

agccgaattctcgatcaagccacccgatccaatgcagttggcgcgccattcgatatcgga

ccctttccctttagtgagggttaatgct

>ACA62_s1_SynSno_hyb10nt

acctttgccaagccaatggcggctaactatctgaagaaccagccgatgtacgtgttccgt

aagacggagctcaagcactccaagaccgagctcaacttcaaggagtggcaaaaggccttt

accgatgtgatgggcatggacgagctgtacaagtaaactagtagcagacagatgcgccgt

ggataaattgttatactgcagattgtgtcaaagacaggaagttgcctgattatttctggc

agccgaattcatttggcagccacccgatccaatgcagttggcgcgccattcgatatcgga

ccctttccctttagtgagggttaatgct

>ACA62_s2_SynSno_hyb10nt

acctttgccaagccaatggcggctaactatctgaagaaccagccgatgtacgtgttccgt

aagacggagctcaagcactccaagaccgagctcaacttcaaggagtggcaaaaggccttt

accgatgtgatgggcatggacgagctgtacaagtaaactagtagcagacagatgcgccgt

ggataaattgttatacgcggttaaatgagttatgcgcgaagttgcctgattatttctggc

agccgaattctcatggaagccacccgatccaatgcagttggcgcgccattcgatatcgga

ccctttccctttagtgagggttaatgct

>ACA63_SynSno_hyb10nt

acctttgccaagccaatggcggctaactatctgaagaaccagccgatgtacgtgttccgt

aagacggagctcaagcactccaagaccgagctcaacttcaaggagtggcaaaaggccttt

accgatgtgatgggcatggacgagctgtacaagtaaactagtagcagacagatgcgccgt

ggataaattgttatagacagaaaaatgatagtgagtcgaagttgcctgattatttctggc

agccgaatttaaaacgaagccacccgatccaatgcagttggcgcgccattcgatatcgga

ccctttccctttagtgagggttaatgct

>ACA64_SynSno_hyb10nt

acctttgccaagccaatggcggctaactatctgaagaaccagccgatgtacgtgttccgt

aagacggagctcaagcactccaagaccgagctcaacttcaaggagtggcaaaaggccttt

accgatgtgatgggcatggacgagctgtacaagtaaactagtagcagacagatgcgccgt

ggataaattgttatagcctgggttttgagctgaggccgaagttgcctgattatttctggc

agccgaattaaccagcgagccacccgatccaatgcagttggcgcgccattcgatatcgga

ccctttccctttagtgagggttaatgct

>ACA65_s1_SynSno_hyb10nt

acctttgccaagccaatggcggctaactatctgaagaaccagccgatgtacgtgttccgt

aagacggagctcaagcactccaagaccgagctcaacttcaaggagtggcaaaaggccttt

accgatgtgatgggcatggacgagctgtacaagtaaactagtagcagacagatgcgccgt

ggataaattgttatatgacctaaaatgagaacagtcagaagttgcctgattatttctggc

agccgaattactttcccagccacccgatccaatgcagttggcgcgccattcgatatcgga

ccctttccctttagtgagggttaatgct

>ACA65_s2_SynSno_hyb10nt

acctttgccaagccaatggcggctaactatctgaagaaccagccgatgtacgtgttccgt

aagacggagctcaagcactccaagaccgagctcaacttcaaggagtggcaaaaggccttt

accgatgtgatgggcatggacgagctgtacaagtaaactagtagcagacagatgcgccgt

ggataaattgttatattccgcaaattggtgaagaaaagaagttgcctgattatttctggc

agccgaattcccacctgagccacccgatccaatgcagttggcgcgccattcgatatcgga

ccctttccctttagtgagggttaatgct

>ACA67_s1_SynSno_hyb10nt

acctttgccaagccaatggcggctaactatctgaagaaccagccgatgtacgtgttccgt

aagacggagctcaagcactccaagaccgagctcaacttcaaggagtggcaaaaggccttt

accgatgtgatgggcatggacgagctgtacaagtaaactagtagcagacagatgcgccgt

ggataaattgttataatggggtacttgaaatccaaacgaagttgcctgattatttctggc

agccgaattagggtaacagccacccgatccaatgcagttggcgcgccattcgatatcgga

ccctttccctttagtgagggttaatgct

>ACA67_s2_SynSno_hyb10nt

acctttgccaagccaatggcggctaactatctgaagaaccagccgatgtacgtgttccgt

aagacggagctcaagcactccaagaccgagctcaacttcaaggagtggcaaaaggccttt

accgatgtgatgggcatggacgagctgtacaagtaaactagtagcagacagatgcgccgt

ggataaattgttatacccacttcagtgatggttcagggaagttgcctgattatttctggc

agccgaattggagaagtagccacccgatccaatgcagttggcgcgccattcgatatcgga

ccctttccctttagtgagggttaatgct

>ACA7_s1_SynSno_hyb10nt

acctttgccaagccaatggcggctaactatctgaagaaccagccgatgtacgtgttccgt

aagacggagctcaagcactccaagaccgagctcaacttcaaggagtggcaaaaggccttt

accgatgtgatgggcatggacgagctgtacaagtaaactagtagcagacagatgcgccgt

ggataaattgttatatccctttccgtggcgatcccaggaagttgcctgattatttctggc

agccgaattcgttccaaagccacccgatccaatgcagttggcgcgccattcgatatcgga

ccctttccctttagtgagggttaatgct

>ACA7_s2_SynSno_hyb10nt

acctttgccaagccaatggcggctaactatctgaagaaccagccgatgtacgtgttccgt

aagacggagctcaagcactccaagaccgagctcaacttcaaggagtggcaaaaggccttt

accgatgtgatgggcatggacgagctgtacaagtaaactagtagcagacagatgcgccgt

ggataaattgttataagagtgtctttgaatgggtgccgaagttgcctgattatttctggc

agccgaattaacactccagccacccgatccaatgcagttggcgcgccattcgatatcgga

ccctttccctttagtgagggttaatgct

>ACA8_s1_SynSno_hyb10nt

acctttgccaagccaatggcggctaactatctgaagaaccagccgatgtacgtgttccgt

aagacggagctcaagcactccaagaccgagctcaacttcaaggagtggcaaaaggccttt

accgatgtgatgggcatggacgagctgtacaagtaaactagtagcagacagatgcgccgt

ggataaattgttatatgacctttgatgagataccatggaagttgcctgattatttctggc

agccgaattcagcccggagccacccgatccaatgcagttggcgcgccattcgatatcgga

ccctttccctttagtgagggttaatgct

>ACA8_s2_SynSno_hyb10nt

acctttgccaagccaatggcggctaactatctgaagaaccagccgatgtacgtgttccgt

aagacggagctcaagcactccaagaccgagctcaacttcaaggagtggcaaaaggccttt

accgatgtgatgggcatggacgagctgtacaagtaaactagtagcagacagatgcgccgt

ggataaattgttataagaaataccatgcaacgatgccgaagttgcctgattatttctggc

agccgaatttagtacccagccacccgatccaatgcagttggcgcgccattcgatatcgga

ccctttccctttagtgagggttaatgct

>ACA9_s1_SynSno_hyb10nt

acctttgccaagccaatggcggctaactatctgaagaaccagccgatgtacgtgttccgt

aagacggagctcaagcactccaagaccgagctcaacttcaaggagtggcaaaaggccttt

accgatgtgatgggcatggacgagctgtacaagtaaactagtagcagacagatgcgccgt

ggataaattgttatacactgaaggatggctggaggctgaagttgcctgattatttctggc

agccgaattaccgtgtcagccacccgatccaatgcagttggcgcgccattcgatatcgga

ccctttccctttagtgagggttaatgct

>ACA9_s2_SynSno_hyb10nt

acctttgccaagccaatggcggctaactatctgaagaaccagccgatgtacgtgttccgt

aagacggagctcaagcactccaagaccgagctcaacttcaaggagtggcaaaaggccttt

accgatgtgatgggcatggacgagctgtacaagtaaactagtagcagacagatgcgccgt

ggataaattgttataaaatttctattgtcaaaccgttgaagttgcctgattatttctggc

agccgaattcaccgtttagccacccgatccaatgcagttggcgcgccattcgatatcgga

ccctttccctttagtgagggttaatgct

>E2_s1_SynSno_hyb10nt

acctttgccaagccaatggcggctaactatctgaagaaccagccgatgtacgtgttccgt

aagacggagctcaagcactccaagaccgagctcaacttcaaggagtggcaaaaggccttt

accgatgtgatgggcatggacgagctgtacaagtaaactagtagcagacagatgcgccgt

ggataaattgttatacactacctactgtctaacaatggaagttgcctgattatttctggc

agccgaattactgtggtagccacccgatccaatgcagttggcgcgccattcgatatcgga

ccctttccctttagtgagggttaatgct

>E2_s2_SynSno_hyb10nt

acctttgccaagccaatggcggctaactatctgaagaaccagccgatgtacgtgttccgt

aagacggagctcaagcactccaagaccgagctcaacttcaaggagtggcaaaaggccttt

accgatgtgatgggcatggacgagctgtacaagtaaactagtagcagacagatgcgccgt

ggataaattgttataagaattactatgtagcgaaaacgaagttgcctgattatttctggc

agccgaatttgcccttaagccacccgatccaatgcagttggcgcgccattcgatatcgga

ccctttccctttagtgagggttaatgct

>E3_SynSno_hyb10nt

acctttgccaagccaatggcggctaactatctgaagaaccagccgatgtacgtgttccgt

aagacggagctcaagcactccaagaccgagctcaacttcaaggagtggcaaaaggccttt

accgatgtgatgggcatggacgagctgtacaagtaaactagtagcagacagatgcgccgt

ggataaattgttatagaccaagcgttgatagttaagagaagttgcctgattatttctggc

agccgaattacatcgttagccacccgatccaatgcagttggcgcgccattcgatatcgga

ccctttccctttagtgagggttaatgct

>HBI-115_SynSno_hyb10nt

acctttgccaagccaatggcggctaactatctgaagaaccagccgatgtacgtgttccgt

aagacggagctcaagcactccaagaccgagctcaacttcaaggagtggcaaaaggccttt

accgatgtgatgggcatggacgagctgtacaagtaaactagtagcagacagatgcgccgt

ggataaattgttataggtcttaacctgttctcagtccgaagttgcctgattatttctggc

agccgaatttgtctgacagccacccgatccaatgcagttggcgcgccattcgatatcgga

ccctttccctttagtgagggttaatgct

>HBI-6_SynSno_hyb10nt

acctttgccaagccaatggcggctaactatctgaagaaccagccgatgtacgtgttccgt

aagacggagctcaagcactccaagaccgagctcaacttcaaggagtggcaaaaggccttt

accgatgtgatgggcatggacgagctgtacaagtaaactagtagcagacagatgcgccgt

ggataaattgttatacagacaggattggttttatggagaagttgcctgattatttctggc

agccgaatttcacggtaagccacccgatccaatgcagttggcgcgccattcgatatcgga

ccctttccctttagtgagggttaatgct

>HBI-61_SynSno_hyb10nt

acctttgccaagccaatggcggctaactatctgaagaaccagccgatgtacgtgttccgt

aagacggagctcaagcactccaagaccgagctcaacttcaaggagtggcaaaaggccttt

accgatgtgatgggcatggacgagctgtacaagtaaactagtagcagacagatgcgccgt

ggataaattgttatacttgtgtgtatgtgcttatacagaagttgcctgattatttctggc

agccgaattggtgatcaagccacccgatccaatgcagttggcgcgccattcgatatcgga

ccctttccctttagtgagggttaatgct

>ACA1_SynSno_8nt

acctttgccaagccaatggcggctaactatctgaagaaccagccgatgtacgtgttccgt

aagacggagctcaagcactccaagaccgagctcaacttcaaggagtggcaaaaggccttt

accgatgtgatgggcatggacgagctgtacaagtaaactagtagcagacagatgcgccgt

ggataaattgttatatttctcattgtgaagcataaaagaagttgcctgattatttctggc

agccgaattctcatataagccacccgatccaatgcagttggcgcgccattcgatatcgga

ccctttccctttagtgagggttaatgct

>ACA10_s1_SynSno_8nt

acctttgccaagccaatggcggctaactatctgaagaaccagccgatgtacgtgttccgt

aagacggagctcaagcactccaagaccgagctcaacttcaaggagtggcaaaaggccttt

accgatgtgatgggcatggacgagctgtacaagtaaactagtagcagacagatgcgccgt

ggataaattgttataaatgaaaacatgagctgagatcgaagttgcctgattatttctggc

agccgaattcttgacgaagccacccgatccaatgcagttggcgcgccattcgatatcgga

ccctttccctttagtgagggttaatgct

>ACA10_s2_SynSno_8nt

acctttgccaagccaatggcggctaactatctgaagaaccagccgatgtacgtgttccgt

aagacggagctcaagcactccaagaccgagctcaacttcaaggagtggcaaaaggccttt

accgatgtgatgggcatggacgagctgtacaagtaaactagtagcagacagatgcgccgt

ggataaattgttatacatcctaggatgcgtgcattctgaagttgcctgattatttctggc

agccgaattcattaaagagccacccgatccaatgcagttggcgcgccattcgatatcgga

ccctttccctttagtgagggttaatgct

>ACA13_SynSno_8nt

acctttgccaagccaatggcggctaactatctgaagaaccagccgatgtacgtgttccgt

aagacggagctcaagcactccaagaccgagctcaacttcaaggagtggcaaaaggccttt

accgatgtgatgggcatggacgagctgtacaagtaaactagtagcagacagatgcgccgt

ggataaattgttataagtttttgactgaacacaaattgaagttgcctgattatttctggc

agccgaattggagctgaagccacccgatccaatgcagttggcgcgccattcgatatcgga

ccctttccctttagtgagggttaatgct

>ACA14a_SynSno_8nt

acctttgccaagccaatggcggctaactatctgaagaaccagccgatgtacgtgttccgt

aagacggagctcaagcactccaagaccgagctcaacttcaaggagtggcaaaaggccttt

accgatgtgatgggcatggacgagctgtacaagtaaactagtagcagacagatgcgccgt

ggataaattgttatatttatgaaattgttggacttgagaagttgcctgattatttctggc

agccgaatttgcggtcgagccacccgatccaatgcagttggcgcgccattcgatatcgga

ccctttccctttagtgagggttaatgct

>ACA15_SynSno_8nt

acctttgccaagccaatggcggctaactatctgaagaaccagccgatgtacgtgttccgt

aagacggagctcaagcactccaagaccgagctcaacttcaaggagtggcaaaaggccttt

accgatgtgatgggcatggacgagctgtacaagtaaactagtagcagacagatgcgccgt

ggataaattgttatagtgtgtctggtgtattcggcacgaagttgcctgattatttctggc

agccgaattgttagtaaagccacccgatccaatgcagttggcgcgccattcgatatcgga

ccctttccctttagtgagggttaatgct

>ACA16_SynSno_8nt

acctttgccaagccaatggcggctaactatctgaagaaccagccgatgtacgtgttccgt

aagacggagctcaagcactccaagaccgagctcaacttcaaggagtggcaaaaggccttt

accgatgtgatgggcatggacgagctgtacaagtaaactagtagcagacagatgcgccgt

ggataaattgttatagactttttgatgcttcgatactgaagttgcctgattatttctggc

agccgaatttttagccaagccacccgatccaatgcagttggcgcgccattcgatatcgga

ccctttccctttagtgagggttaatgct

>ACA17_s1_SynSno_8nt

acctttgccaagccaatggcggctaactatctgaagaaccagccgatgtacgtgttccgt

aagacggagctcaagcactccaagaccgagctcaacttcaaggagtggcaaaaggccttt

accgatgtgatgggcatggacgagctgtacaagtaaactagtagcagacagatgcgccgt

ggataaattgttatacactaatgactgaacgcctcgcgaagttgcctgattatttctggc

agccgaattagtattcgagccacccgatccaatgcagttggcgcgccattcgatatcgga

ccctttccctttagtgagggttaatgct

>ACA17_s2_SynSno_8nt

acctttgccaagccaatggcggctaactatctgaagaaccagccgatgtacgtgttccgt

aagacggagctcaagcactccaagaccgagctcaacttcaaggagtggcaaaaggccttt

accgatgtgatgggcatggacgagctgtacaagtaaactagtagcagacagatgcgccgt

ggataaattgttatagctcaaacattgagcgtagaacgaagttgcctgattatttctggc

agccgaattcttgcgctagccacccgatccaatgcagttggcgcgccattcgatatcgga

ccctttccctttagtgagggttaatgct

>ACA19_s1_SynSno_8nt

acctttgccaagccaatggcggctaactatctgaagaaccagccgatgtacgtgttccgt

aagacggagctcaagcactccaagaccgagctcaacttcaaggagtggcaaaaggccttt

accgatgtgatgggcatggacgagctgtacaagtaaactagtagcagacagatgcgccgt

ggataaattgttataactaagaaattgaatgaaattggaagttgcctgattatttctggc

agccgaattagggtctcagccacccgatccaatgcagttggcgcgccattcgatatcgga

ccctttccctttagtgagggttaatgct

>ACA19_s2_SynSno_8nt

acctttgccaagccaatggcggctaactatctgaagaaccagccgatgtacgtgttccgt

aagacggagctcaagcactccaagaccgagctcaacttcaaggagtggcaaaaggccttt

accgatgtgatgggcatggacgagctgtacaagtaaactagtagcagacagatgcgccgt

ggataaattgttataccggagactgtgtaattagcaggaagttgcctgattatttctggc

agccgaattttgttgtaagccacccgatccaatgcagttggcgcgccattcgatatcgga

ccctttccctttagtgagggttaatgct

>ACA19_s3_SynSno_8nt

acctttgccaagccaatggcggctaactatctgaagaaccagccgatgtacgtgttccgt

aagacggagctcaagcactccaagaccgagctcaacttcaaggagtggcaaaaggccttt

accgatgtgatgggcatggacgagctgtacaagtaaactagtagcagacagatgcgccgt

ggataaattgttataaccataagaatgatgaaatggtgaagttgcctgattatttctggc

agccgaattgtcacagaagccacccgatccaatgcagttggcgcgccattcgatatcgga

ccctttccctttagtgagggttaatgct

>ACA20_SynSno_8nt

acctttgccaagccaatggcggctaactatctgaagaaccagccgatgtacgtgttccgt

aagacggagctcaagcactccaagaccgagctcaacttcaaggagtggcaaaaggccttt

accgatgtgatgggcatggacgagctgtacaagtaaactagtagcagacagatgcgccgt

ggataaattgttataactgcgtatatgaaataaatttgaagttgcctgattatttctggc

agccgaatttccaagatagccacccgatccaatgcagttggcgcgccattcgatatcgga

ccctttccctttagtgagggttaatgct

>ACA21_s1_SynSno_8nt

acctttgccaagccaatggcggctaactatctgaagaaccagccgatgtacgtgttccgt

aagacggagctcaagcactccaagaccgagctcaacttcaaggagtggcaaaaggccttt

accgatgtgatgggcatggacgagctgtacaagtaaactagtagcagacagatgcgccgt

ggataaattgttataggtcttgacgtggcttttaaccgaagttgcctgattatttctggc

agccgaatttcgcggacagccacccgatccaatgcagttggcgcgccattcgatatcgga

ccctttccctttagtgagggttaatgct

>ACA21_s2_SynSno_8nt

acctttgccaagccaatggcggctaactatctgaagaaccagccgatgtacgtgttccgt

aagacggagctcaagcactccaagaccgagctcaacttcaaggagtggcaaaaggccttt

accgatgtgatgggcatggacgagctgtacaagtaaactagtagcagacagatgcgccgt

ggataaattgttataaaaccgattgtgcacccaaactgaagttgcctgattatttctggc

agccgaattatgtaatgagccacccgatccaatgcagttggcgcgccattcgatatcgga

ccctttccctttagtgagggttaatgct

>ACA22_s1_SynSno_8nt

acctttgccaagccaatggcggctaactatctgaagaaccagccgatgtacgtgttccgt

aagacggagctcaagcactccaagaccgagctcaacttcaaggagtggcaaaaggccttt

accgatgtgatgggcatggacgagctgtacaagtaaactagtagcagacagatgcgccgt

ggataaattgttatactggtcaaagtgtcactgtgacgaagttgcctgattatttctggc

agccgaattaaccgaatagccacccgatccaatgcagttggcgcgccattcgatatcgga

ccctttccctttagtgagggttaatgct

>ACA22_s2_SynSno_8nt

acctttgccaagccaatggcggctaactatctgaagaaccagccgatgtacgtgttccgt

aagacggagctcaagcactccaagaccgagctcaacttcaaggagtggcaaaaggccttt

accgatgtgatgggcatggacgagctgtacaagtaaactagtagcagacagatgcgccgt

ggataaattgttatagatgaatacatggcagaggatcgaagttgcctgattatttctggc

agccgaattgaactagaagccacccgatccaatgcagttggcgcgccattcgatatcgga

ccctttccctttagtgagggttaatgct

>ACA23_s1_SynSno_8nt

acctttgccaagccaatggcggctaactatctgaagaaccagccgatgtacgtgttccgt

aagacggagctcaagcactccaagaccgagctcaacttcaaggagtggcaaaaggccttt

accgatgtgatgggcatggacgagctgtacaagtaaactagtagcagacagatgcgccgt

ggataaattgttataactgatgttttgagcagccagtgaagttgcctgattatttctggc

agccgaatttattctatagccacccgatccaatgcagttggcgcgccattcgatatcgga

ccctttccctttagtgagggttaatgct

>ACA23_s2_SynSno_8nt

acctttgccaagccaatggcggctaactatctgaagaaccagccgatgtacgtgttccgt

aagacggagctcaagcactccaagaccgagctcaacttcaaggagtggcaaaaggccttt

accgatgtgatgggcatggacgagctgtacaagtaaactagtagcagacagatgcgccgt

ggataaattgttatatcatttggagtgactatgataagaagttgcctgattatttctggc

agccgaattataactgcagccacccgatccaatgcagttggcgcgccattcgatatcgga

ccctttccctttagtgagggttaatgct

>ACA24_s1_SynSno_8nt

acctttgccaagccaatggcggctaactatctgaagaaccagccgatgtacgtgttccgt

aagacggagctcaagcactccaagaccgagctcaacttcaaggagtggcaaaaggccttt

accgatgtgatgggcatggacgagctgtacaagtaaactagtagcagacagatgcgccgt

ggataaattgttatagtgctaggaatgaaagataccggaagttgcctgattatttctggc

agccgaattgtgaggaaagccacccgatccaatgcagttggcgcgccattcgatatcgga

ccctttccctttagtgagggttaatgct

>ACA24_s2_SynSno_8nt

acctttgccaagccaatggcggctaactatctgaagaaccagccgatgtacgtgttccgt

aagacggagctcaagcactccaagaccgagctcaacttcaaggagtggcaaaaggccttt

accgatgtgatgggcatggacgagctgtacaagtaaactagtagcagacagatgcgccgt

ggataaattgttatagttgcaagtctgttgccaatccgaagttgcctgattatttctggc

agccgaatttagtggcgagccacccgatccaatgcagttggcgcgccattcgatatcgga

ccctttccctttagtgagggttaatgct

>ACA25_s1_SynSno_8nt

acctttgccaagccaatggcggctaactatctgaagaaccagccgatgtacgtgttccgt

aagacggagctcaagcactccaagaccgagctcaacttcaaggagtggcaaaaggccttt

accgatgtgatgggcatggacgagctgtacaagtaaactagtagcagacagatgcgccgt

ggataaattgttatagaacagcgtttgctttgaaagtgaagttgcctgattatttctggc

agccgaattgactcgaaagccacccgatccaatgcagttggcgcgccattcgatatcgga

ccctttccctttagtgagggttaatgct

>ACA25_s2_SynSno_8nt

acctttgccaagccaatggcggctaactatctgaagaaccagccgatgtacgtgttccgt

aagacggagctcaagcactccaagaccgagctcaacttcaaggagtggcaaaaggccttt

accgatgtgatgggcatggacgagctgtacaagtaaactagtagcagacagatgcgccgt

ggataaattgttataagcaggaaaatgggagtgctcggaagttgcctgattatttctggc

agccgaattagctaaacagccacccgatccaatgcagttggcgcgccattcgatatcgga

ccctttccctttagtgagggttaatgct

>ACA27_s1_SynSno_8nt

acctttgccaagccaatggcggctaactatctgaagaaccagccgatgtacgtgttccgt

aagacggagctcaagcactccaagaccgagctcaacttcaaggagtggcaaaaggccttt

accgatgtgatgggcatggacgagctgtacaagtaaactagtagcagacagatgcgccgt

ggataaattgttataaccttgaatgtgaaagtgaaccgaagttgcctgattatttctggc

agccgaatttgtaggcaagccacccgatccaatgcagttggcgcgccattcgatatcgga

ccctttccctttagtgagggttaatgct

>ACA27_s2_SynSno_8nt

acctttgccaagccaatggcggctaactatctgaagaaccagccgatgtacgtgttccgt

aagacggagctcaagcactccaagaccgagctcaacttcaaggagtggcaaaaggccttt

accgatgtgatgggcatggacgagctgtacaagtaaactagtagcagacagatgcgccgt

ggataaattgttatagatgacaggatggttttaaactgaagttgcctgattatttctggc

agccgaattgcttgctaagccacccgatccaatgcagttggcgcgccattcgatatcgga

ccctttccctttagtgagggttaatgct

>ACA28_s1_SynSno_8nt

acctttgccaagccaatggcggctaactatctgaagaaccagccgatgtacgtgttccgt

aagacggagctcaagcactccaagaccgagctcaacttcaaggagtggcaaaaggccttt

accgatgtgatgggcatggacgagctgtacaagtaaactagtagcagacagatgcgccgt

ggataaattgttataccgctcaaattggagtgttgaggaagttgcctgattatttctggc

agccgaattccaaagggagccacccgatccaatgcagttggcgcgccattcgatatcgga

ccctttccctttagtgagggttaatgct

>ACA28_s2_SynSno_8nt

acctttgccaagccaatggcggctaactatctgaagaaccagccgatgtacgtgttccgt

aagacggagctcaagcactccaagaccgagctcaacttcaaggagtggcaaaaggccttt

accgatgtgatgggcatggacgagctgtacaagtaaactagtagcagacagatgcgccgt

ggataaattgttatatgctatataatggaataggtgcgaagttgcctgattatttctggc

agccgaatttcccagggagccacccgatccaatgcagttggcgcgccattcgatatcgga

ccctttccctttagtgagggttaatgct

>ACA2a_s1_SynSno_8nt

acctttgccaagccaatggcggctaactatctgaagaaccagccgatgtacgtgttccgt

aagacggagctcaagcactccaagaccgagctcaacttcaaggagtggcaaaaggccttt

accgatgtgatgggcatggacgagctgtacaagtaaactagtagcagacagatgcgccgt

ggataaattgttatagactgtttgatgttgattcattgaagttgcctgattatttctggc

agccgaatttaccagagagccacccgatccaatgcagttggcgcgccattcgatatcgga

ccctttccctttagtgagggttaatgct

>ACA2a_s2_SynSno_8nt

acctttgccaagccaatggcggctaactatctgaagaaccagccgatgtacgtgttccgt

aagacggagctcaagcactccaagaccgagctcaacttcaaggagtggcaaaaggccttt

accgatgtgatgggcatggacgagctgtacaagtaaactagtagcagacagatgcgccgt

ggataaattgttatagacaactgaatgcagaccacccgaagttgcctgattatttctggc

agccgaattgcgcctggagccacccgatccaatgcagttggcgcgccattcgatatcgga

ccctttccctttagtgagggttaatgct

>ACA3_s1_SynSno_8nt

acctttgccaagccaatggcggctaactatctgaagaaccagccgatgtacgtgttccgt

aagacggagctcaagcactccaagaccgagctcaacttcaaggagtggcaaaaggccttt

accgatgtgatgggcatggacgagctgtacaagtaaactagtagcagacagatgcgccgt

ggataaattgttatattactctagctggactctagaagaagttgcctgattatttctggc

agccgaattctagagccagccacccgatccaatgcagttggcgcgccattcgatatcgga

ccctttccctttagtgagggttaatgct

>ACA3_s2_SynSno_8nt

acctttgccaagccaatggcggctaactatctgaagaaccagccgatgtacgtgttccgt

aagacggagctcaagcactccaagaccgagctcaacttcaaggagtggcaaaaggccttt

accgatgtgatgggcatggacgagctgtacaagtaaactagtagcagacagatgcgccgt

ggataaattgttatagcacctggtgtggaataagggagaagttgcctgattatttctggc

agccgaattccggatacagccacccgatccaatgcagttggcgcgccattcgatatcgga

ccctttccctttagtgagggttaatgct

>ACA3-2_SynSno_8nt

acctttgccaagccaatggcggctaactatctgaagaaccagccgatgtacgtgttccgt

aagacggagctcaagcactccaagaccgagctcaacttcaaggagtggcaaaaggccttt

accgatgtgatgggcatggacgagctgtacaagtaaactagtagcagacagatgcgccgt

ggataaattgttatatgactctagctggactctaggagaagttgcctgattatttctggc

agccgaattttacaacaagccacccgatccaatgcagttggcgcgccattcgatatcgga

ccctttccctttagtgagggttaatgct

>ACA30_SynSno_8nt

acctttgccaagccaatggcggctaactatctgaagaaccagccgatgtacgtgttccgt

aagacggagctcaagcactccaagaccgagctcaacttcaaggagtggcaaaaggccttt

accgatgtgatgggcatggacgagctgtacaagtaaactagtagcagacagatgcgccgt

ggataaattgttataaaagctaccatgtgtgaaaggtgaagttgcctgattatttctggc

agccgaattcttttttaagccacccgatccaatgcagttggcgcgccattcgatatcgga

ccctttccctttagtgagggttaatgct

>ACA31_s2_SynSno_8nt

acctttgccaagccaatggcggctaactatctgaagaaccagccgatgtacgtgttccgt

aagacggagctcaagcactccaagaccgagctcaacttcaaggagtggcaaaaggccttt

accgatgtgatgggcatggacgagctgtacaagtaaactagtagcagacagatgcgccgt

ggataaattgttataaacaattcaatgaagcgcagttgaagttgcctgattatttctggc

agccgaattgttggcaaagccacccgatccaatgcagttggcgcgccattcgatatcgga

ccctttccctttagtgagggttaatgct

>ACA32_SynSno_8nt

acctttgccaagccaatggcggctaactatctgaagaaccagccgatgtacgtgttccgt

aagacggagctcaagcactccaagaccgagctcaacttcaaggagtggcaaaaggccttt

accgatgtgatgggcatggacgagctgtacaagtaaactagtagcagacagatgcgccgt

ggataaattgttatagaatgtccactgttggtaattcgaagttgcctgattatttctggc

agccgaatttggcgccgagccacccgatccaatgcagttggcgcgccattcgatatcgga

ccctttccctttagtgagggttaatgct

>ACA33_SynSno_8nt

acctttgccaagccaatggcggctaactatctgaagaaccagccgatgtacgtgttccgt

aagacggagctcaagcactccaagaccgagctcaacttcaaggagtggcaaaaggccttt

accgatgtgatgggcatggacgagctgtacaagtaaactagtagcagacagatgcgccgt

ggataaattgttatacgtctcagggtgtcgtaacagtgaagttgcctgattatttctggc

agccgaattttgtaggcagccacccgatccaatgcagttggcgcgccattcgatatcgga

ccctttccctttagtgagggttaatgct

>ACA34_s1_SynSno_8nt

acctttgccaagccaatggcggctaactatctgaagaaccagccgatgtacgtgttccgt

aagacggagctcaagcactccaagaccgagctcaacttcaaggagtggcaaaaggccttt

accgatgtgatgggcatggacgagctgtacaagtaaactagtagcagacagatgcgccgt

ggataaattgttataagctgcttgatgttcagtcattgaagttgcctgattatttctggc

agccgaattgcgtctccagccacccgatccaatgcagttggcgcgccattcgatatcgga

ccctttccctttagtgagggttaatgct

>ACA34_s2_SynSno_8nt

acctttgccaagccaatggcggctaactatctgaagaaccagccgatgtacgtgttccgt

aagacggagctcaagcactccaagaccgagctcaacttcaaggagtggcaaaaggccttt

accgatgtgatgggcatggacgagctgtacaagtaaactagtagcagacagatgcgccgt

ggataaattgttatagacaaatgaatgcagaccacctgaagttgcctgattatttctggc

agccgaattacgttctcagccacccgatccaatgcagttggcgcgccattcgatatcgga

ccctttccctttagtgagggttaatgct

>ACA36_s1_SynSno_8nt

acctttgccaagccaatggcggctaactatctgaagaaccagccgatgtacgtgttccgt

aagacggagctcaagcactccaagaccgagctcaacttcaaggagtggcaaaaggccttt

accgatgtgatgggcatggacgagctgtacaagtaaactagtagcagacagatgcgccgt

ggataaattgttataccgtttaatttgactcaacaaggaagttgcctgattatttctggc

agccgaattctactgacagccacccgatccaatgcagttggcgcgccattcgatatcgga

ccctttccctttagtgagggttaatgct

>ACA36_s2_SynSno_8nt

acctttgccaagccaatggcggctaactatctgaagaaccagccgatgtacgtgttccgt

aagacggagctcaagcactccaagaccgagctcaacttcaaggagtggcaaaaggccttt

accgatgtgatgggcatggacgagctgtacaagtaaactagtagcagacagatgcgccgt

ggataaattgttatattgaggcaaatgaatcatctaagaagttgcctgattatttctggc

agccgaattaacttaccagccacccgatccaatgcagttggcgcgccattcgatatcgga

ccctttccctttagtgagggttaatgct

>ACA4_SynSno_8nt

acctttgccaagccaatggcggctaactatctgaagaaccagccgatgtacgtgttccgt

aagacggagctcaagcactccaagaccgagctcaacttcaaggagtggcaaaaggccttt

accgatgtgatgggcatggacgagctgtacaagtaaactagtagcagacagatgcgccgt

ggataaattgttatacaaaaatagttggtggagtggtgaagttgcctgattatttctggc

agccgaattattagtagagccacccgatccaatgcagttggcgcgccattcgatatcgga

ccctttccctttagtgagggttaatgct

>ACA40_SynSno_8nt

acctttgccaagccaatggcggctaactatctgaagaaccagccgatgtacgtgttccgt

aagacggagctcaagcactccaagaccgagctcaacttcaaggagtggcaaaaggccttt

accgatgtgatgggcatggacgagctgtacaagtaaactagtagcagacagatgcgccgt

ggataaattgttataagtatatgtatggttgcaaactgaagttgcctgattatttctggc

agccgaattctgttcaaagccacccgatccaatgcagttggcgcgccattcgatatcgga

ccctttccctttagtgagggttaatgct

>ACA41_SynSno_8nt

acctttgccaagccaatggcggctaactatctgaagaaccagccgatgtacgtgttccgt

aagacggagctcaagcactccaagaccgagctcaacttcaaggagtggcaaaaggccttt

accgatgtgatgggcatggacgagctgtacaagtaaactagtagcagacagatgcgccgt

ggataaattgttatacaagaggaattgccagtagcgtgaagttgcctgattatttctggc

agccgaatttagcaggtagccacccgatccaatgcagttggcgcgccattcgatatcgga

ccctttccctttagtgagggttaatgct

>ACA42_s1_SynSno_8nt

acctttgccaagccaatggcggctaactatctgaagaaccagccgatgtacgtgttccgt

aagacggagctcaagcactccaagaccgagctcaacttcaaggagtggcaaaaggccttt

accgatgtgatgggcatggacgagctgtacaagtaaactagtagcagacagatgcgccgt

ggataaattgttatagtggtacacttgaaatccatgcgaagttgcctgattatttctggc

agccgaatttgaaacatagccacccgatccaatgcagttggcgcgccattcgatatcgga

ccctttccctttagtgagggttaatgct

>ACA42_s2_SynSno_8nt

acctttgccaagccaatggcggctaactatctgaagaaccagccgatgtacgtgttccgt

aagacggagctcaagcactccaagaccgagctcaacttcaaggagtggcaaaaggccttt

accgatgtgatgggcatggacgagctgtacaagtaaactagtagcagacagatgcgccgt

ggataaattgttataaacccttcagtgatggttcgctgaagttgcctgattatttctggc

agccgaatttaaggcagagccacccgatccaatgcagttggcgcgccattcgatatcgga

ccctttccctttagtgagggttaatgct

>ACA43_SynSno_8nt

acctttgccaagccaatggcggctaactatctgaagaaccagccgatgtacgtgttccgt

aagacggagctcaagcactccaagaccgagctcaacttcaaggagtggcaaaaggccttt

accgatgtgatgggcatggacgagctgtacaagtaaactagtagcagacagatgcgccgt

ggataaattgttataactaaaccattggtagaaatatgaagttgcctgattatttctggc

agccgaattgaaactgcagccacccgatccaatgcagttggcgcgccattcgatatcgga

ccctttccctttagtgagggttaatgct

>ACA44_s1_SynSno_8nt

acctttgccaagccaatggcggctaactatctgaagaaccagccgatgtacgtgttccgt

aagacggagctcaagcactccaagaccgagctcaacttcaaggagtggcaaaaggccttt

accgatgtgatgggcatggacgagctgtacaagtaaactagtagcagacagatgcgccgt

ggataaattgttatagtcagttggatgttggaaaccggaagttgcctgattatttctggc

agccgaattttgtctcgagccacccgatccaatgcagttggcgcgccattcgatatcgga

ccctttccctttagtgagggttaatgct

>ACA44_s2_SynSno_8nt

acctttgccaagccaatggcggctaactatctgaagaaccagccgatgtacgtgttccgt

aagacggagctcaagcactccaagaccgagctcaacttcaaggagtggcaaaaggccttt

accgatgtgatgggcatggacgagctgtacaagtaaactagtagcagacagatgcgccgt

ggataaattgttataccgctgagtgtgcaaagtctgggaagttgcctgattatttctggc

agccgaattctacccggagccacccgatccaatgcagttggcgcgccattcgatatcgga

ccctttccctttagtgagggttaatgct

>ACA46_SynSno_8nt

acctttgccaagccaatggcggctaactatctgaagaaccagccgatgtacgtgttccgt

aagacggagctcaagcactccaagaccgagctcaacttcaaggagtggcaaaaggccttt

accgatgtgatgggcatggacgagctgtacaagtaaactagtagcagacagatgcgccgt

ggataaattgttataagacagcgtatgttaaatatctgaagttgcctgattatttctggc

agccgaattaataaggcagccacccgatccaatgcagttggcgcgccattcgatatcgga

ccctttccctttagtgagggttaatgct

>ACA48_SynSno_8nt

acctttgccaagccaatggcggctaactatctgaagaaccagccgatgtacgtgttccgt

aagacggagctcaagcactccaagaccgagctcaacttcaaggagtggcaaaaggccttt

accgatgtgatgggcatggacgagctgtacaagtaaactagtagcagacagatgcgccgt

ggataaattgttataccggcatgaatggatcaaaatagaagttgcctgattatttctggc

agccgaattgtacgcctagccacccgatccaatgcagttggcgcgccattcgatatcgga

ccctttccctttagtgagggttaatgct

>U19_up_SynSno_8nt

acctttgccaagccaatggcggctaactatctgaagaaccagccgatgtacgtgttccgt

aagacggagctcaagcactccaagaccgagctcaacttcaaggagtggcaaaaggccttt

accgatgtgatgggcatggacgagctgtacaagtaaactagtagcagacagatgcgccgt

ggataaattgttatacaagtgtaactgtgacaacctagaagttgcctgattatttctggc

agccgaattaccttgacagccacccgatccaatgcagttggcgcgccattcgatatcgga

ccctttccctttagtgagggttaatgct

>U19_dn_SynSno_8nt

acctttgccaagccaatggcggctaactatctgaagaaccagccgatgtacgtgttccgt

aagacggagctcaagcactccaagaccgagctcaacttcaaggagtggcaaaaggccttt

accgatgtgatgggcatggacgagctgtacaagtaaactagtagcagacagatgcgccgt

ggataaattgttatacaccagactatgctctcagattgaagttgcctgattatttctggc

agccgaatttcgttccaagccacccgatccaatgcagttggcgcgccattcgatatcgga

ccctttccctttagtgagggttaatgct

>U23_up_SynSno_8nt

acctttgccaagccaatggcggctaactatctgaagaaccagccgatgtacgtgttccgt

aagacggagctcaagcactccaagaccgagctcaacttcaaggagtggcaaaaggccttt

accgatgtgatgggcatggacgagctgtacaagtaaactagtagcagacagatgcgccgt

ggataaattgttataagcgtgcgaatggctcaatgctgaagttgcctgattatttctggc

agccgaattcgcatcgaagccacccgatccaatgcagttggcgcgccattcgatatcgga

ccctttccctttagtgagggttaatgct

>U64_up_SynSno_8nt

acctttgccaagccaatggcggctaactatctgaagaaccagccgatgtacgtgttccgt

aagacggagctcaagcactccaagaccgagctcaacttcaaggagtggcaaaaggccttt

accgatgtgatgggcatggacgagctgtacaagtaaactagtagcagacagatgcgccgt

ggataaattgttataaaccgttacgtggcagagcctcgaagttgcctgattatttctggc

agccgaattctacagtcagccacccgatccaatgcagttggcgcgccattcgatatcgga

ccctttccctttagtgagggttaatgct

>U65_up_SynSno_8nt

acctttgccaagccaatggcggctaactatctgaagaaccagccgatgtacgtgttccgt

aagacggagctcaagcactccaagaccgagctcaacttcaaggagtggcaaaaggccttt

accgatgtgatgggcatggacgagctgtacaagtaaactagtagcagacagatgcgccgt

ggataaattgttataacccactggatggtggcggggtgaagttgcctgattatttctggc

agccgaattgtcgttccagccacccgatccaatgcagttggcgcgccattcgatatcgga

ccctttccctttagtgagggttaatgct

>U65_dn_SynSno_8nt

acctttgccaagccaatggcggctaactatctgaagaaccagccgatgtacgtgttccgt

aagacggagctcaagcactccaagaccgagctcaacttcaaggagtggcaaaaggccttt

accgatgtgatgggcatggacgagctgtacaagtaaactagtagcagacagatgcgccgt

ggataaattgttatagtctttcggctgttcctaagaggaagttgcctgattatttctggc

agccgaattttgccatcagccacccgatccaatgcagttggcgcgccattcgatatcgga

ccctttccctttagtgagggttaatgct

>U66_up_SynSno_8nt

acctttgccaagccaatggcggctaactatctgaagaaccagccgatgtacgtgttccgt

aagacggagctcaagcactccaagaccgagctcaacttcaaggagtggcaaaaggccttt

accgatgtgatgggcatggacgagctgtacaagtaaactagtagcagacagatgcgccgt

ggataaattgttatacactggttcctgtgatcgaggggaagttgcctgattatttctggc

agccgaattattgaagaagccacccgatccaatgcagttggcgcgccattcgatatcgga

ccctttccctttagtgagggttaatgct

>U67_up_SynSno_8nt

acctttgccaagccaatggcggctaactatctgaagaaccagccgatgtacgtgttccgt

aagacggagctcaagcactccaagaccgagctcaacttcaaggagtggcaaaaggccttt

accgatgtgatgggcatggacgagctgtacaagtaaactagtagcagacagatgcgccgt

ggataaattgttatactctgctacttggagggaatacgaagttgcctgattatttctggc

agccgaatttaaatcgcagccacccgatccaatgcagttggcgcgccattcgatatcgga

ccctttccctttagtgagggttaatgct

>U68_up_SynSno_8nt

acctttgccaagccaatggcggctaactatctgaagaaccagccgatgtacgtgttccgt

aagacggagctcaagcactccaagaccgagctcaacttcaaggagtggcaaaaggccttt

accgatgtgatgggcatggacgagctgtacaagtaaactagtagcagacagatgcgccgt

ggataaattgttatagacaaattcatggcgacaagcggaagttgcctgattatttctggc

agccgaatttctagataagccacccgatccaatgcagttggcgcgccattcgatatcgga

ccctttccctttagtgagggttaatgct

>U69_up_SynSno_8nt

acctttgccaagccaatggcggctaactatctgaagaaccagccgatgtacgtgttccgt

aagacggagctcaagcactccaagaccgagctcaacttcaaggagtggcaaaaggccttt

accgatgtgatgggcatggacgagctgtacaagtaaactagtagcagacagatgcgccgt

ggataaattgttataacggataatgtgaattgcaaaagaagttgcctgattatttctggc

agccgaattacacccctagccacccgatccaatgcagttggcgcgccattcgatatcgga

ccctttccctttagtgagggttaatgct

>U69_dn_SynSno_8nt

acctttgccaagccaatggcggctaactatctgaagaaccagccgatgtacgtgttccgt

aagacggagctcaagcactccaagaccgagctcaacttcaaggagtggcaaaaggccttt

accgatgtgatgggcatggacgagctgtacaagtaaactagtagcagacagatgcgccgt

ggataaattgttatagaaatctgtctgaaaaatgagggaagttgcctgattatttctggc

agccgaatttcgattgtagccacccgatccaatgcagttggcgcgccattcgatatcgga

ccctttccctttagtgagggttaatgct

>U70_up_SynSno_8nt

acctttgccaagccaatggcggctaactatctgaagaaccagccgatgtacgtgttccgt

aagacggagctcaagcactccaagaccgagctcaacttcaaggagtggcaaaaggccttt

accgatgtgatgggcatggacgagctgtacaagtaaactagtagcagacagatgcgccgt

ggataaattgttataccggtccctttgtacactacacgaagttgcctgattatttctggc

agccgaattgtttgttaagccacccgatccaatgcagttggcgcgccattcgatatcgga

ccctttccctttagtgagggttaatgct

>U71a_up_SynSno_8nt

acctttgccaagccaatggcggctaactatctgaagaaccagccgatgtacgtgttccgt

aagacggagctcaagcactccaagaccgagctcaacttcaaggagtggcaaaaggccttt

accgatgtgatgggcatggacgagctgtacaagtaaactagtagcagacagatgcgccgt

ggataaattgttataccgcttcaggtgacggggacccgaagttgcctgattatttctggc

agccgaattcccaacggagccacccgatccaatgcagttggcgcgccattcgatatcgga

ccctttccctttagtgagggttaatgct

>U72_up_SynSno_8nt

acctttgccaagccaatggcggctaactatctgaagaaccagccgatgtacgtgttccgt

aagacggagctcaagcactccaagaccgagctcaacttcaaggagtggcaaaaggccttt

accgatgtgatgggcatggacgagctgtacaagtaaactagtagcagacagatgcgccgt

ggataaattgttatacctgtttagctgcgagaatagggaagttgcctgattatttctggc

agccgaattgcaagctcagccacccgatccaatgcagttggcgcgccattcgatatcgga

ccctttccctttagtgagggttaatgct

>U99_up_SynSno_8nt

acctttgccaagccaatggcggctaactatctgaagaaccagccgatgtacgtgttccgt

aagacggagctcaagcactccaagaccgagctcaacttcaaggagtggcaaaaggccttt

accgatgtgatgggcatggacgagctgtacaagtaaactagtagcagacagatgcgccgt

ggataaattgttatagacgtacatttgccaagaaactgaagttgcctgattatttctggc

agccgaatttgccgcttagccacccgatccaatgcagttggcgcgccattcgatatcgga

ccctttccctttagtgagggttaatgct

>ACA5_s1_SynSno_8nt

acctttgccaagccaatggcggctaactatctgaagaaccagccgatgtacgtgttccgt

aagacggagctcaagcactccaagaccgagctcaacttcaaggagtggcaaaaggccttt

accgatgtgatgggcatggacgagctgtacaagtaaactagtagcagacagatgcgccgt

ggataaattgttataaattctgggctgaatttgaccagaagttgcctgattatttctggc

agccgaatttcttccaaagccacccgatccaatgcagttggcgcgccattcgatatcgga

ccctttccctttagtgagggttaatgct

>ACA5_s2_SynSno_8nt

acctttgccaagccaatggcggctaactatctgaagaaccagccgatgtacgtgttccgt

aagacggagctcaagcactccaagaccgagctcaacttcaaggagtggcaaaaggccttt

accgatgtgatgggcatggacgagctgtacaagtaaactagtagcagacagatgcgccgt

ggataaattgttataaaaaatttattgcccatgaggagaagttgcctgattatttctggc

agccgaattggccaggcagccacccgatccaatgcagttggcgcgccattcgatatcgga

ccctttccctttagtgagggttaatgct

>ACA50_s1_SynSno_8nt

acctttgccaagccaatggcggctaactatctgaagaaccagccgatgtacgtgttccgt

aagacggagctcaagcactccaagaccgagctcaacttcaaggagtggcaaaaggccttt

accgatgtgatgggcatggacgagctgtacaagtaaactagtagcagacagatgcgccgt

ggataaattgttataaggttgcttgtgtcaaaggcctgaagttgcctgattatttctggc

agccgaattgagtcaaaagccacccgatccaatgcagttggcgcgccattcgatatcgga

ccctttccctttagtgagggttaatgct

>ACA50_s2_SynSno_8nt

acctttgccaagccaatggcggctaactatctgaagaaccagccgatgtacgtgttccgt

aagacggagctcaagcactccaagaccgagctcaacttcaaggagtggcaaaaggccttt

accgatgtgatgggcatggacgagctgtacaagtaaactagtagcagacagatgcgccgt

ggataaattgttatagttttttaaatgagttataaacgaagttgcctgattatttctggc

agccgaattacaatttcagccacccgatccaatgcagttggcgcgccattcgatatcgga

ccctttccctttagtgagggttaatgct

>ACA52_SynSno_8nt

acctttgccaagccaatggcggctaactatctgaagaaccagccgatgtacgtgttccgt

aagacggagctcaagcactccaagaccgagctcaacttcaaggagtggcaaaaggccttt

accgatgtgatgggcatggacgagctgtacaagtaaactagtagcagacagatgcgccgt

ggataaattgttataaccaagcgaatgattaggatttgaagttgcctgattatttctggc

agccgaattcttgttacagccacccgatccaatgcagttggcgcgccattcgatatcgga

ccctttccctttagtgagggttaatgct

>ACA54_s1_SynSno_8nt

acctttgccaagccaatggcggctaactatctgaagaaccagccgatgtacgtgttccgt

aagacggagctcaagcactccaagaccgagctcaacttcaaggagtggcaaaaggccttt

accgatgtgatgggcatggacgagctgtacaagtaaactagtagcagacagatgcgccgt

ggataaattgttataaaggaatggatgtacgaacatggaagttgcctgattatttctggc

agccgaattgatcggtgagccacccgatccaatgcagttggcgcgccattcgatatcgga

ccctttccctttagtgagggttaatgct

>ACA55_SynSno_8nt

acctttgccaagccaatggcggctaactatctgaagaaccagccgatgtacgtgttccgt

aagacggagctcaagcactccaagaccgagctcaacttcaaggagtggcaaaaggccttt

accgatgtgatgggcatggacgagctgtacaagtaaactagtagcagacagatgcgccgt

ggataaattgttatacacatctgtctgaaagattcctgaagttgcctgattatttctggc

agccgaatttccacagaagccacccgatccaatgcagttggcgcgccattcgatatcgga

ccctttccctttagtgagggttaatgct

>ACA56_SynSno_8nt

acctttgccaagccaatggcggctaactatctgaagaaccagccgatgtacgtgttccgt

aagacggagctcaagcactccaagaccgagctcaacttcaaggagtggcaaaaggccttt

accgatgtgatgggcatggacgagctgtacaagtaaactagtagcagacagatgcgccgt

ggataaattgttatacatgactccctggggataacgtgaagttgcctgattatttctggc

agccgaattgcgtcgttagccacccgatccaatgcagttggcgcgccattcgatatcgga

ccctttccctttagtgagggttaatgct

>ACA58_SynSno_8nt

acctttgccaagccaatggcggctaactatctgaagaaccagccgatgtacgtgttccgt

aagacggagctcaagcactccaagaccgagctcaacttcaaggagtggcaaaaggccttt

accgatgtgatgggcatggacgagctgtacaagtaaactagtagcagacagatgcgccgt

ggataaattgttatacaactgtccctgtctacgaggcgaagttgcctgattatttctggc

agccgaattcaaagattagccacccgatccaatgcagttggcgcgccattcgatatcgga

ccctttccctttagtgagggttaatgct

>ACA6_SynSno_8nt

acctttgccaagccaatggcggctaactatctgaagaaccagccgatgtacgtgttccgt

aagacggagctcaagcactccaagaccgagctcaacttcaaggagtggcaaaaggccttt

accgatgtgatgggcatggacgagctgtacaagtaaactagtagcagacagatgcgccgt

ggataaattgttataaccttccgactgtttaataggtgaagttgcctgattatttctggc

agccgaatttacacaccagccacccgatccaatgcagttggcgcgccattcgatatcgga

ccctttccctttagtgagggttaatgct

>ACA60_SynSno_8nt

acctttgccaagccaatggcggctaactatctgaagaaccagccgatgtacgtgttccgt

aagacggagctcaagcactccaagaccgagctcaacttcaaggagtggcaaaaggccttt

accgatgtgatgggcatggacgagctgtacaagtaaactagtagcagacagatgcgccgt

ggataaattgttataatccagcatttgccaaacacttgaagttgcctgattatttctggc

agccgaattcggtgttaagccacccgatccaatgcagttggcgcgccattcgatatcgga

ccctttccctttagtgagggttaatgct

>ACA61_SynSno_8nt

acctttgccaagccaatggcggctaactatctgaagaaccagccgatgtacgtgttccgt

aagacggagctcaagcactccaagaccgagctcaacttcaaggagtggcaaaaggccttt

accgatgtgatgggcatggacgagctgtacaagtaaactagtagcagacagatgcgccgt

ggataaattgttataaattttacgatggaaagggagagaagttgcctgattatttctggc

agccgaattaacctgcaagccacccgatccaatgcagttggcgcgccattcgatatcgga

ccctttccctttagtgagggttaatgct

>ACA62_s1_SynSno_8nt

acctttgccaagccaatggcggctaactatctgaagaaccagccgatgtacgtgttccgt

aagacggagctcaagcactccaagaccgagctcaacttcaaggagtggcaaaaggccttt

accgatgtgatgggcatggacgagctgtacaagtaaactagtagcagacagatgcgccgt

ggataaattgttataaggcagattgtgtcaaagacctgaagttgcctgattatttctggc

agccgaattcctggttcagccacccgatccaatgcagttggcgcgccattcgatatcgga

ccctttccctttagtgagggttaatgct

>ACA62_s2_SynSno_8nt

acctttgccaagccaatggcggctaactatctgaagaaccagccgatgtacgtgttccgt

aagacggagctcaagcactccaagaccgagctcaacttcaaggagtggcaaaaggccttt

accgatgtgatgggcatggacgagctgtacaagtaaactagtagcagacagatgcgccgt

ggataaattgttataatcggttaaatgagttatgctagaagttgcctgattatttctggc

agccgaattcaacatcgagccacccgatccaatgcagttggcgcgccattcgatatcgga

ccctttccctttagtgagggttaatgct

>ACA63_SynSno_8nt

acctttgccaagccaatggcggctaactatctgaagaaccagccgatgtacgtgttccgt

aagacggagctcaagcactccaagaccgagctcaacttcaaggagtggcaaaaggccttt

accgatgtgatgggcatggacgagctgtacaagtaaactagtagcagacagatgcgccgt

ggataaattgttatatccagaaaaatgatagtgaggagaagttgcctgattatttctggc

agccgaattaacctggaagccacccgatccaatgcagttggcgcgccattcgatatcgga

ccctttccctttagtgagggttaatgct

>ACA64_SynSno_8nt

acctttgccaagccaatggcggctaactatctgaagaaccagccgatgtacgtgttccgt

aagacggagctcaagcactccaagaccgagctcaacttcaaggagtggcaaaaggccttt

accgatgtgatgggcatggacgagctgtacaagtaaactagtagcagacagatgcgccgt

ggataaattgttatatactgggttttgagctgaggaagaagttgcctgattatttctggc

agccgaattagcgatcaagccacccgatccaatgcagttggcgcgccattcgatatcgga

ccctttccctttagtgagggttaatgct

>ACA65_s1_SynSno_8nt

acctttgccaagccaatggcggctaactatctgaagaaccagccgatgtacgtgttccgt

aagacggagctcaagcactccaagaccgagctcaacttcaaggagtggcaaaaggccttt

accgatgtgatgggcatggacgagctgtacaagtaaactagtagcagacagatgcgccgt

ggataaattgttatagtacctaaaatgagaacagtacgaagttgcctgattatttctggc

agccgaatttgcagctaagccacccgatccaatgcagttggcgcgccattcgatatcgga

ccctttccctttagtgagggttaatgct

>ACA65_s2_SynSno_8nt

acctttgccaagccaatggcggctaactatctgaagaaccagccgatgtacgtgttccgt

aagacggagctcaagcactccaagaccgagctcaacttcaaggagtggcaaaaggccttt

accgatgtgatgggcatggacgagctgtacaagtaaactagtagcagacagatgcgccgt

ggataaattgttataggccgcaaattggtgaagaaccgaagttgcctgattatttctggc

agccgaattcgttcctaagccacccgatccaatgcagttggcgcgccattcgatatcgga

ccctttccctttagtgagggttaatgct

>ACA67_s1_SynSno_8nt

acctttgccaagccaatggcggctaactatctgaagaaccagccgatgtacgtgttccgt

aagacggagctcaagcactccaagaccgagctcaacttcaaggagtggcaaaaggccttt

accgatgtgatgggcatggacgagctgtacaagtaaactagtagcagacagatgcgccgt

ggataaattgttatacgggggtacttgaaatccaacagaagttgcctgattatttctggc

agccgaattcccgcgtaagccacccgatccaatgcagttggcgcgccattcgatatcgga

ccctttccctttagtgagggttaatgct

>ACA67_s2_SynSno_8nt

acctttgccaagccaatggcggctaactatctgaagaaccagccgatgtacgtgttccgt

aagacggagctcaagcactccaagaccgagctcaacttcaaggagtggcaaaaggccttt

accgatgtgatgggcatggacgagctgtacaagtaaactagtagcagacagatgcgccgt

ggataaattgttataaacacttcagtgatggttcattgaagttgcctgattatttctggc

agccgaattttggcactagccacccgatccaatgcagttggcgcgccattcgatatcgga

ccctttccctttagtgagggttaatgct

>ACA7_s1_SynSno_8nt

acctttgccaagccaatggcggctaactatctgaagaaccagccgatgtacgtgttccgt

aagacggagctcaagcactccaagaccgagctcaacttcaaggagtggcaaaaggccttt

accgatgtgatgggcatggacgagctgtacaagtaaactagtagcagacagatgcgccgt

ggataaattgttatagacctttccgtggcgatcccctgaagttgcctgattatttctggc

agccgaatttccgaactagccacccgatccaatgcagttggcgcgccattcgatatcgga

ccctttccctttagtgagggttaatgct

>ACA8_s1_SynSno_8nt

acctttgccaagccaatggcggctaactatctgaagaaccagccgatgtacgtgttccgt

aagacggagctcaagcactccaagaccgagctcaacttcaaggagtggcaaaaggccttt

accgatgtgatgggcatggacgagctgtacaagtaaactagtagcagacagatgcgccgt

ggataaattgttatagtacctttgatgagataccagtgaagttgcctgattatttctggc

agccgaattatttataaagccacccgatccaatgcagttggcgcgccattcgatatcgga

ccctttccctttagtgagggttaatgct

>ACA8_s2_SynSno_8nt

acctttgccaagccaatggcggctaactatctgaagaaccagccgatgtacgtgttccgt

aagacggagctcaagcactccaagaccgagctcaacttcaaggagtggcaaaaggccttt

accgatgtgatgggcatggacgagctgtacaagtaaactagtagcagacagatgcgccgt

ggataaattgttatactaaataccatgcaacgatgaagaagttgcctgattatttctggc

agccgaattcttatcgtagccacccgatccaatgcagttggcgcgccattcgatatcgga

ccctttccctttagtgagggttaatgct

>ACA9_s1_SynSno_8nt

acctttgccaagccaatggcggctaactatctgaagaaccagccgatgtacgtgttccgt

aagacggagctcaagcactccaagaccgagctcaacttcaaggagtggcaaaaggccttt

accgatgtgatgggcatggacgagctgtacaagtaaactagtagcagacagatgcgccgt

ggataaattgttataacctgaaggatggctggaggaggaagttgcctgattatttctggc

agccgaattgatggtccagccacccgatccaatgcagttggcgcgccattcgatatcgga

ccctttccctttagtgagggttaatgct

>ACA9_s2_SynSno_8nt

acctttgccaagccaatggcggctaactatctgaagaaccagccgatgtacgtgttccgt

aagacggagctcaagcactccaagaccgagctcaacttcaaggagtggcaaaaggccttt

accgatgtgatgggcatggacgagctgtacaagtaaactagtagcagacagatgcgccgt

ggataaattgttataccatttctattgtcaaaccggggaagttgcctgattatttctggc

agccgaattatctaccaagccacccgatccaatgcagttggcgcgccattcgatatcgga

ccctttccctttagtgagggttaatgct

>E2_s1_SynSno_8nt

acctttgccaagccaatggcggctaactatctgaagaaccagccgatgtacgtgttccgt

aagacggagctcaagcactccaagaccgagctcaacttcaaggagtggcaaaaggccttt

accgatgtgatgggcatggacgagctgtacaagtaaactagtagcagacagatgcgccgt

ggataaattgttataacctacctactgtctaacaagtgaagttgcctgattatttctggc

agccgaattttcacccaagccacccgatccaatgcagttggcgcgccattcgatatcgga

ccctttccctttagtgagggttaatgct

>E2_s2_SynSno_8nt

acctttgccaagccaatggcggctaactatctgaagaaccagccgatgtacgtgttccgt

aagacggagctcaagcactccaagaccgagctcaacttcaaggagtggcaaaaggccttt

accgatgtgatgggcatggacgagctgtacaagtaaactagtagcagacagatgcgccgt

ggataaattgttatactaattactatgtagcgaaacagaagttgcctgattatttctggc

agccgaatttagtccagagccacccgatccaatgcagttggcgcgccattcgatatcgga

ccctttccctttagtgagggttaatgct

>E3_SynSno_8nt

acctttgccaagccaatggcggctaactatctgaagaaccagccgatgtacgtgttccgt

aagacggagctcaagcactccaagaccgagctcaacttcaaggagtggcaaaaggccttt

accgatgtgatgggcatggacgagctgtacaagtaaactagtagcagacagatgcgccgt

ggataaattgttatatcccaagcgttgatagttaatcgaagttgcctgattatttctggc

agccgaattcgaaatacagccacccgatccaatgcagttggcgcgccattcgatatcgga

ccctttccctttagtgagggttaatgct

>HBI-115_SynSno_8nt

acctttgccaagccaatggcggctaactatctgaagaaccagccgatgtacgtgttccgt

aagacggagctcaagcactccaagaccgagctcaacttcaaggagtggcaaaaggccttt

accgatgtgatgggcatggacgagctgtacaagtaaactagtagcagacagatgcgccgt

ggataaattgttatatttcttaacctgttctcagtaagaagttgcctgattatttctggc

agccgaattgcctgtcgagccacccgatccaatgcagttggcgcgccattcgatatcgga

ccctttccctttagtgagggttaatgct

>HBI-6_SynSno_8nt

acctttgccaagccaatggcggctaactatctgaagaaccagccgatgtacgtgttccgt

aagacggagctcaagcactccaagaccgagctcaacttcaaggagtggcaaaaggccttt

accgatgtgatgggcatggacgagctgtacaagtaaactagtagcagacagatgcgccgt

ggataaattgttataacgacaggattggttttatgtcgaagttgcctgattatttctggc

agccgaattcctcgaagagccacccgatccaatgcagttggcgcgccattcgatatcgga

ccctttccctttagtgagggttaatgct

>HBI-61_SynSno_8nt

acctttgccaagccaatggcggctaactatctgaagaaccagccgatgtacgtgttccgt

aagacggagctcaagcactccaagaccgagctcaacttcaaggagtggcaaaaggccttt

accgatgtgatgggcatggacgagctgtacaagtaaactagtagcagacagatgcgccgt

ggataaattgttataagtgtgtgtatgtgcttataacgaagttgcctgattatttctggc

agccgaatttaattcctagccacccgatccaatgcagttggcgcgccattcgatatcgga

ccctttccctttagtgagggttaatgct

>ACA1_SynSno_5nt

acctttgccaagccaatggcggctaactatctgaagaaccagccgatgtacgtgttccgt

aagacggagctcaagcactccaagaccgagctcaacttcaaggagtggcaaaaggccttt

accgatgtgatgggcatggacgagctgtacaagtaaactagtagcagacagatgcgccgt

ggataaattgttatattgagcattgtgaagccgccaagaagttgcctgattatttctggc

agccgaattaattcaacagccacccgatccaatgcagttggcgcgccattcgatatcgga

ccctttccctttagtgagggttaatgct

>ACA10_s1_SynSno_5nt

acctttgccaagccaatggcggctaactatctgaagaaccagccgatgtacgtgttccgt

aagacggagctcaagcactccaagaccgagctcaacttcaaggagtggcaaaaggccttt

accgatgtgatgggcatggacgagctgtacaagtaaactagtagcagacagatgcgccgt

ggataaattgttataaagtcaaacatgagcttctctcgaagttgcctgattatttctggc

agccgaattcgttgctaagccacccgatccaatgcagttggcgcgccattcgatatcgga

ccctttccctttagtgagggttaatgct

>ACA10_s2_SynSno_5nt

acctttgccaagccaatggcggctaactatctgaagaaccagccgatgtacgtgttccgt

aagacggagctcaagcactccaagaccgagctcaacttcaaggagtggcaaaaggccttt

accgatgtgatgggcatggacgagctgtacaagtaaactagtagcagacagatgcgccgt

ggataaattgttatacagaataggatgcgtgacggctgaagttgcctgattatttctggc

agccgaatttcgaagccagccacccgatccaatgcagttggcgcgccattcgatatcgga

ccctttccctttagtgagggttaatgct

>ACA13_SynSno_5nt

acctttgccaagccaatggcggctaactatctgaagaaccagccgatgtacgtgttccgt

aagacggagctcaagcactccaagaccgagctcaacttcaaggagtggcaaaaggccttt

accgatgtgatgggcatggacgagctgtacaagtaaactagtagcagacagatgcgccgt

ggataaattgttataaggggttgactgaacaacccttgaagttgcctgattatttctggc

agccgaattctgtatggagccacccgatccaatgcagttggcgcgccattcgatatcgga

ccctttccctttagtgagggttaatgct

>ACA14a_SynSno_5nt

acctttgccaagccaatggcggctaactatctgaagaaccagccgatgtacgtgttccgt

aagacggagctcaagcactccaagaccgagctcaacttcaaggagtggcaaaaggccttt

accgatgtgatgggcatggacgagctgtacaagtaaactagtagcagacagatgcgccgt

ggataaattgttatattgcggaaattgttggcagggagaagttgcctgattatttctggc

agccgaattcgcctactagccacccgatccaatgcagttggcgcgccattcgatatcgga

ccctttccctttagtgagggttaatgct

>ACA15_SynSno_5nt

acctttgccaagccaatggcggctaactatctgaagaaccagccgatgtacgtgttccgt

aagacggagctcaagcactccaagaccgagctcaacttcaaggagtggcaaaaggccttt

accgatgtgatgggcatggacgagctgtacaagtaaactagtagcagacagatgcgccgt

ggataaattgttatagttgttctggtgtattattaacgaagttgcctgattatttctggc

agccgaattgtatggacagccacccgatccaatgcagttggcgcgccattcgatatcgga

ccctttccctttagtgagggttaatgct

>ACA16_SynSno_5nt

acctttgccaagccaatggcggctaactatctgaagaaccagccgatgtacgtgttccgt

aagacggagctcaagcactccaagaccgagctcaacttcaaggagtggcaaaaggccttt

accgatgtgatgggcatggacgagctgtacaagtaaactagtagcagacagatgcgccgt

ggataaattgttatagaaggtttgatgcttctcgcctgaagttgcctgattatttctggc

agccgaatttatcgagaagccacccgatccaatgcagttggcgcgccattcgatatcgga

ccctttccctttagtgagggttaatgct

>ACA17_s1_SynSno_5nt

acctttgccaagccaatggcggctaactatctgaagaaccagccgatgtacgtgttccgt

aagacggagctcaagcactccaagaccgagctcaacttcaaggagtggcaaaaggccttt

accgatgtgatgggcatggacgagctgtacaagtaaactagtagcagacagatgcgccgt

ggataaattgttatacaagcatgactgaacgaagagcgaagttgcctgattatttctggc

agccgaattcctcgaccagccacccgatccaatgcagttggcgcgccattcgatatcgga

ccctttccctttagtgagggttaatgct

>ACA17_s2_SynSno_5nt

acctttgccaagccaatggcggctaactatctgaagaaccagccgatgtacgtgttccgt

aagacggagctcaagcactccaagaccgagctcaacttcaaggagtggcaaaaggccttt

accgatgtgatgggcatggacgagctgtacaagtaaactagtagcagacagatgcgccgt

ggataaattgttatagcgacaacattgagcggctcacgaagttgcctgattatttctggc

agccgaatttaccgacaagccacccgatccaatgcagttggcgcgccattcgatatcgga

ccctttccctttagtgagggttaatgct

>ACA19_s1_SynSno_5nt

acctttgccaagccaatggcggctaactatctgaagaaccagccgatgtacgtgttccgt

aagacggagctcaagcactccaagaccgagctcaacttcaaggagtggcaaaaggccttt

accgatgtgatgggcatggacgagctgtacaagtaaactagtagcagacagatgcgccgt

ggataaattgttataacgccgaaattgaatgcccgtggaagttgcctgattatttctggc

agccgaattacggtggtagccacccgatccaatgcagttggcgcgccattcgatatcgga

ccctttccctttagtgagggttaatgct

>ACA19_s2_SynSno_5nt

acctttgccaagccaatggcggctaactatctgaagaaccagccgatgtacgtgttccgt

aagacggagctcaagcactccaagaccgagctcaacttcaaggagtggcaaaaggccttt

accgatgtgatgggcatggacgagctgtacaagtaaactagtagcagacagatgcgccgt

ggataaattgttataccttcgactgtgtaatgctaaggaagttgcctgattatttctggc

agccgaattttctatccagccacccgatccaatgcagttggcgcgccattcgatatcgga

ccctttccctttagtgagggttaatgct

>ACA19_s3_SynSno_5nt

acctttgccaagccaatggcggctaactatctgaagaaccagccgatgtacgtgttccgt

aagacggagctcaagcactccaagaccgagctcaacttcaaggagtggcaaaaggccttt

accgatgtgatgggcatggacgagctgtacaagtaaactagtagcagacagatgcgccgt

ggataaattgttataacacgaagaatgatgaccgtgtgaagttgcctgattatttctggc

agccgaattctgagcggagccacccgatccaatgcagttggcgcgccattcgatatcgga

ccctttccctttagtgagggttaatgct

>ACA20_SynSno_5nt

acctttgccaagccaatggcggctaactatctgaagaaccagccgatgtacgtgttccgt

aagacggagctcaagcactccaagaccgagctcaacttcaaggagtggcaaaaggccttt

accgatgtgatgggcatggacgagctgtacaagtaaactagtagcagacagatgcgccgt

ggataaattgttataacgtagtatatgaaatcccgttgaagttgcctgattatttctggc

agccgaattgaaattatagccacccgatccaatgcagttggcgcgccattcgatatcgga

ccctttccctttagtgagggttaatgct

>ACA21_s1_SynSno_5nt

acctttgccaagccaatggcggctaactatctgaagaaccagccgatgtacgtgttccgt

aagacggagctcaagcactccaagaccgagctcaacttcaaggagtggcaaaaggccttt

accgatgtgatgggcatggacgagctgtacaagtaaactagtagcagacagatgcgccgt

ggataaattgttatagggagtgacgtggcttggccccgaagttgcctgattatttctggc

agccgaatttaaggactagccacccgatccaatgcagttggcgcgccattcgatatcgga

ccctttccctttagtgagggttaatgct

>ACA21_s2_SynSno_5nt

acctttgccaagccaatggcggctaactatctgaagaaccagccgatgtacgtgttccgt

aagacggagctcaagcactccaagaccgagctcaacttcaaggagtggcaaaaggccttt

accgatgtgatgggcatggacgagctgtacaagtaaactagtagcagacagatgcgccgt

ggataaattgttataaacaagattgtgcaccacccctgaagttgcctgattatttctggc

agccgaattaactattgagccacccgatccaatgcagttggcgcgccattcgatatcgga

ccctttccctttagtgagggttaatgct

>ACA22_s1_SynSno_5nt

acctttgccaagccaatggcggctaactatctgaagaaccagccgatgtacgtgttccgt

aagacggagctcaagcactccaagaccgagctcaacttcaaggagtggcaaaaggccttt

accgatgtgatgggcatggacgagctgtacaagtaaactagtagcagacagatgcgccgt

ggataaattgttatactttgcaaagtgtcacgtgtacgaagttgcctgattatttctggc

agccgaattcgccttgaagccacccgatccaatgcagttggcgcgccattcgatatcgga

ccctttccctttagtgagggttaatgct

>ACA22_s2_SynSno_5nt

acctttgccaagccaatggcggctaactatctgaagaaccagccgatgtacgtgttccgt

aagacggagctcaagcactccaagaccgagctcaacttcaaggagtggcaaaaggccttt

accgatgtgatgggcatggacgagctgtacaagtaaactagtagcagacagatgcgccgt

ggataaattgttatagagtcatacatggcagcttctcgaagttgcctgattatttctggc

agccgaattcaccatcaagccacccgatccaatgcagttggcgcgccattcgatatcgga

ccctttccctttagtgagggttaatgct

>ACA23_s1_SynSno_5nt

acctttgccaagccaatggcggctaactatctgaagaaccagccgatgtacgtgttccgt

aagacggagctcaagcactccaagaccgagctcaacttcaaggagtggcaaaaggccttt

accgatgtgatgggcatggacgagctgtacaagtaaactagtagcagacagatgcgccgt

ggataaattgttataacgtctgttttgagcataacgtgaagttgcctgattatttctggc

agccgaattattccgttagccacccgatccaatgcagttggcgcgccattcgatatcgga

ccctttccctttagtgagggttaatgct

>ACA24_s1_SynSno_5nt

acctttgccaagccaatggcggctaactatctgaagaaccagccgatgtacgtgttccgt

aagacggagctcaagcactccaagaccgagctcaacttcaaggagtggcaaaaggccttt

accgatgtgatgggcatggacgagctgtacaagtaaactagtagcagacagatgcgccgt

ggataaattgttatagttagaggaatgaaagcgcacggaagttgcctgattatttctggc

agccgaattcgtagctcagccacccgatccaatgcagttggcgcgccattcgatatcgga

ccctttccctttagtgagggttaatgct

>ACA24_s2_SynSno_5nt

acctttgccaagccaatggcggctaactatctgaagaaccagccgatgtacgtgttccgt

aagacggagctcaagcactccaagaccgagctcaacttcaaggagtggcaaaaggccttt

accgatgtgatgggcatggacgagctgtacaagtaaactagtagcagacagatgcgccgt

ggataaattgttatagtgtaaagtctgttgcaccgccgaagttgcctgattatttctggc

agccgaattgatctgcgagccacccgatccaatgcagttggcgcgccattcgatatcgga

ccctttccctttagtgagggttaatgct

>ACA25_s1_SynSno_5nt

acctttgccaagccaatggcggctaactatctgaagaaccagccgatgtacgtgttccgt

aagacggagctcaagcactccaagaccgagctcaacttcaaggagtggcaaaaggccttt

accgatgtgatgggcatggacgagctgtacaagtaaactagtagcagacagatgcgccgt

ggataaattgttatagacacgcgtttgcttttcccgtgaagttgcctgattatttctggc

agccgaattttccaagcagccacccgatccaatgcagttggcgcgccattcgatatcgga

ccctttccctttagtgagggttaatgct

>ACA25_s2_SynSno_5nt

acctttgccaagccaatggcggctaactatctgaagaaccagccgatgtacgtgttccgt

aagacggagctcaagcactccaagaccgagctcaacttcaaggagtggcaaaaggccttt

accgatgtgatgggcatggacgagctgtacaagtaaactagtagcagacagatgcgccgt

ggataaattgttataagactgaaaatgggaggtagcggaagttgcctgattatttctggc

agccgaattgcgcactcagccacccgatccaatgcagttggcgcgccattcgatatcgga

ccctttccctttagtgagggttaatgct

>ACA27_s1_SynSno_5nt

acctttgccaagccaatggcggctaactatctgaagaaccagccgatgtacgtgttccgt

aagacggagctcaagcactccaagaccgagctcaacttcaaggagtggcaaaaggccttt

accgatgtgatgggcatggacgagctgtacaagtaaactagtagcagacagatgcgccgt

ggataaattgttataacagggaatgtgaaaggtccccgaagttgcctgattatttctggc

agccgaattagccacttagccacccgatccaatgcagttggcgcgccattcgatatcgga

ccctttccctttagtgagggttaatgct

>ACA27_s2_SynSno_5nt

acctttgccaagccaatggcggctaactatctgaagaaccagccgatgtacgtgttccgt

aagacggagctcaagcactccaagaccgagctcaacttcaaggagtggcaaaaggccttt

accgatgtgatgggcatggacgagctgtacaagtaaactagtagcagacagatgcgccgt

ggataaattgttatagagtccaggatggtttgcccctgaagttgcctgattatttctggc

agccgaattcgaggaacagccacccgatccaatgcagttggcgcgccattcgatatcgga

ccctttccctttagtgagggttaatgct

>ACA28_s1_SynSno_5nt

acctttgccaagccaatggcggctaactatctgaagaaccagccgatgtacgtgttccgt

aagacggagctcaagcactccaagaccgagctcaacttcaaggagtggcaaaaggccttt

accgatgtgatgggcatggacgagctgtacaagtaaactagtagcagacagatgcgccgt

ggataaattgttatacctagcaaattggagttggtaggaagttgcctgattatttctggc

agccgaattctagggtaagccacccgatccaatgcagttggcgcgccattcgatatcgga

ccctttccctttagtgagggttaatgct

>ACA28_s2_SynSno_5nt

acctttgccaagccaatggcggctaactatctgaagaaccagccgatgtacgtgttccgt

aagacggagctcaagcactccaagaccgagctcaacttcaaggagtggcaaaaggccttt

accgatgtgatgggcatggacgagctgtacaagtaaactagtagcagacagatgcgccgt

ggataaattgttatatgagctataatggaatcttggcgaagttgcctgattatttctggc

agccgaattacggaactagccacccgatccaatgcagttggcgcgccattcgatatcgga

ccctttccctttagtgagggttaatgct

>ACA2a_s1_SynSno_5nt

acctttgccaagccaatggcggctaactatctgaagaaccagccgatgtacgtgttccgt

aagacggagctcaagcactccaagaccgagctcaacttcaaggagtggcaaaaggccttt

accgatgtgatgggcatggacgagctgtacaagtaaactagtagcagacagatgcgccgt

ggataaattgttatagaagttttgatgttgaggacttgaagttgcctgattatttctggc

agccgaattcacgagcaagccacccgatccaatgcagttggcgcgccattcgatatcgga

ccctttccctttagtgagggttaatgct

>ACA2a_s2_SynSno_5nt

acctttgccaagccaatggcggctaactatctgaagaaccagccgatgtacgtgttccgt

aagacggagctcaagcactccaagaccgagctcaacttcaaggagtggcaaaaggccttt

accgatgtgatgggcatggacgagctgtacaagtaaactagtagcagacagatgcgccgt

ggataaattgttatagaaccctgaatgcagaaacaccgaagttgcctgattatttctggc

agccgaatttatccgagagccacccgatccaatgcagttggcgcgccattcgatatcgga

ccctttccctttagtgagggttaatgct

>ACA3_s1_SynSno_5nt

acctttgccaagccaatggcggctaactatctgaagaaccagccgatgtacgtgttccgt

aagacggagctcaagcactccaagaccgagctcaacttcaaggagtggcaaaaggccttt

accgatgtgatgggcatggacgagctgtacaagtaaactagtagcagacagatgcgccgt

ggataaattgttatattcagctagctggactagctaagaagttgcctgattatttctggc

agccgaattgttgtctgagccacccgatccaatgcagttggcgcgccattcgatatcgga

ccctttccctttagtgagggttaatgct

>ACA3_s2_SynSno_5nt

acctttgccaagccaatggcggctaactatctgaagaaccagccgatgtacgtgttccgt

aagacggagctcaagcactccaagaccgagctcaacttcaaggagtggcaaaaggccttt

accgatgtgatgggcatggacgagctgtacaagtaaactagtagcagacagatgcgccgt

ggataaattgttatagccaatggtgtggaatccttgagaagttgcctgattatttctggc

agccgaattaccacttgagccacccgatccaatgcagttggcgcgccattcgatatcgga

ccctttccctttagtgagggttaatgct

>ACA3-2_SynSno_5nt

acctttgccaagccaatggcggctaactatctgaagaaccagccgatgtacgtgttccgt

aagacggagctcaagcactccaagaccgagctcaacttcaaggagtggcaaaaggccttt

accgatgtgatgggcatggacgagctgtacaagtaaactagtagcagacagatgcgccgt

ggataaattgttatatgcagctagctggactagctgagaagttgcctgattatttctggc

agccgaattgtgatacgagccacccgatccaatgcagttggcgcgccattcgatatcgga

ccctttccctttagtgagggttaatgct

>ACA30_SynSno_5nt

acctttgccaagccaatggcggctaactatctgaagaaccagccgatgtacgtgttccgt

aagacggagctcaagcactccaagaccgagctcaacttcaaggagtggcaaaaggccttt

accgatgtgatgggcatggacgagctgtacaagtaaactagtagcagacagatgcgccgt

ggataaattgttataaactataccatgtgtgccctgtgaagttgcctgattatttctggc

agccgaatttaggagttagccacccgatccaatgcagttggcgcgccattcgatatcgga

ccctttccctttagtgagggttaatgct

>ACA31_s1_SynSno_5nt

acctttgccaagccaatggcggctaactatctgaagaaccagccgatgtacgtgttccgt

aagacggagctcaagcactccaagaccgagctcaacttcaaggagtggcaaaaggccttt

accgatgtgatgggcatggacgagctgtacaagtaaactagtagcagacagatgcgccgt

ggataaattgttatagaagcgtgcatgtatcctgttcgaagttgcctgattatttctggc

agccgaattagggtccgagccacccgatccaatgcagttggcgcgccattcgatatcgga

ccctttccctttagtgagggttaatgct

>ACA31_s2_SynSno_5nt

acctttgccaagccaatggcggctaactatctgaagaaccagccgatgtacgtgttccgt

aagacggagctcaagcactccaagaccgagctcaacttcaaggagtggcaaaaggccttt

accgatgtgatgggcatggacgagctgtacaagtaaactagtagcagacagatgcgccgt

ggataaattgttataaaaccttcaatgaagctactttgaagttgcctgattatttctggc

agccgaattcatgcagaagccacccgatccaatgcagttggcgcgccattcgatatcgga

ccctttccctttagtgagggttaatgct

>ACA32_SynSno_5nt

acctttgccaagccaatggcggctaactatctgaagaaccagccgatgtacgtgttccgt

aagacggagctcaagcactccaagaccgagctcaacttcaaggagtggcaaaaggccttt

accgatgtgatgggcatggacgagctgtacaagtaaactagtagcagacagatgcgccgt

ggataaattgttatagacgttccactgttgggccgtcgaagttgcctgattatttctggc

agccgaattggatgtccagccacccgatccaatgcagttggcgcgccattcgatatcgga

ccctttccctttagtgagggttaatgct

>ACA33_SynSno_5nt

acctttgccaagccaatggcggctaactatctgaagaaccagccgatgtacgtgttccgt

aagacggagctcaagcactccaagaccgagctcaacttcaaggagtggcaaaaggccttt

accgatgtgatgggcatggacgagctgtacaagtaaactagtagcagacagatgcgccgt

ggataaattgttatacggagcagggtgtcgtccacgtgaagttgcctgattatttctggc

agccgaatttatatgttagccacccgatccaatgcagttggcgcgccattcgatatcgga

ccctttccctttagtgagggttaatgct

>ACA34_s1_SynSno_5nt

acctttgccaagccaatggcggctaactatctgaagaaccagccgatgtacgtgttccgt

aagacggagctcaagcactccaagaccgagctcaacttcaaggagtggcaaaaggccttt

accgatgtgatgggcatggacgagctgtacaagtaaactagtagcagacagatgcgccgt

ggataaattgttataagagtcttgatgttcatgacttgaagttgcctgattatttctggc

agccgaattggctgttgagccacccgatccaatgcagttggcgcgccattcgatatcgga

ccctttccctttagtgagggttaatgct

>ACA34_s2_SynSno_5nt

acctttgccaagccaatggcggctaactatctgaagaaccagccgatgtacgtgttccgt

aagacggagctcaagcactccaagaccgagctcaacttcaaggagtggcaaaaggccttt

accgatgtgatgggcatggacgagctgtacaagtaaactagtagcagacagatgcgccgt

ggataaattgttatagaaccatgaatgcagaaacactgaagttgcctgattatttctggc

agccgaattatgaaacgagccacccgatccaatgcagttggcgcgccattcgatatcgga

ccctttccctttagtgagggttaatgct

>ACA36_s1_SynSno_5nt

acctttgccaagccaatggcggctaactatctgaagaaccagccgatgtacgtgttccgt

aagacggagctcaagcactccaagaccgagctcaacttcaaggagtggcaaaaggccttt

accgatgtgatgggcatggacgagctgtacaagtaaactagtagcagacagatgcgccgt

ggataaattgttatacctggtaatttgactcccacaggaagttgcctgattatttctggc

agccgaatttgtgtctaagccacccgatccaatgcagttggcgcgccattcgatatcgga

ccctttccctttagtgagggttaatgct

>ACA36_s2_SynSno_5nt

acctttgccaagccaatggcggctaactatctgaagaaccagccgatgtacgtgttccgt

aagacggagctcaagcactccaagaccgagctcaacttcaaggagtggcaaaaggccttt

accgatgtgatgggcatggacgagctgtacaagtaaactagtagcagacagatgcgccgt

ggataaattgttatatttctgcaaatgaatccgagaagaagttgcctgattatttctggc

agccgaattgaggtaggagccacccgatccaatgcagttggcgcgccattcgatatcgga

ccctttccctttagtgagggttaatgct

>ACA4_SynSno_5nt

acctttgccaagccaatggcggctaactatctgaagaaccagccgatgtacgtgttccgt

aagacggagctcaagcactccaagaccgagctcaacttcaaggagtggcaaaaggccttt

accgatgtgatgggcatggacgagctgtacaagtaaactagtagcagacagatgcgccgt

ggataaattgttatacacccatagttggtggctgtgtgaagttgcctgattatttctggc

agccgaatttgagaggcagccacccgatccaatgcagttggcgcgccattcgatatcgga

ccctttccctttagtgagggttaatgct

>ACA40_SynSno_5nt

acctttgccaagccaatggcggctaactatctgaagaaccagccgatgtacgtgttccgt

aagacggagctcaagcactccaagaccgagctcaacttcaaggagtggcaaaaggccttt

accgatgtgatgggcatggacgagctgtacaagtaaactagtagcagacagatgcgccgt

ggataaattgttataaggcgatgtatggttgacccctgaagttgcctgattatttctggc

agccgaattctaaatttagccacccgatccaatgcagttggcgcgccattcgatatcgga

ccctttccctttagtgagggttaatgct

>ACA41_SynSno_5nt

acctttgccaagccaatggcggctaactatctgaagaaccagccgatgtacgtgttccgt

aagacggagctcaagcactccaagaccgagctcaacttcaaggagtggcaaaaggccttt

accgatgtgatgggcatggacgagctgtacaagtaaactagtagcagacagatgcgccgt

ggataaattgttatacactcggaattgccaggctagtgaagttgcctgattatttctggc

agccgaattaccctaaaagccacccgatccaatgcagttggcgcgccattcgatatcgga

ccctttccctttagtgagggttaatgct

>ACA42_s1_SynSno_5nt

acctttgccaagccaatggcggctaactatctgaagaaccagccgatgtacgtgttccgt

aagacggagctcaagcactccaagaccgagctcaacttcaaggagtggcaaaaggccttt

accgatgtgatgggcatggacgagctgtacaagtaaactagtagcagacagatgcgccgt

ggataaattgttatagtttgacacttgaaataacggcgaagttgcctgattatttctggc

agccgaatttctggctgagccacccgatccaatgcagttggcgcgccattcgatatcgga

ccctttccctttagtgagggttaatgct

>ACA42_s2_SynSno_5nt

acctttgccaagccaatggcggctaactatctgaagaaccagccgatgtacgtgttccgt

aagacggagctcaagcactccaagaccgagctcaacttcaaggagtggcaaaaggccttt

accgatgtgatgggcatggacgagctgtacaagtaaactagtagcagacagatgcgccgt

ggataaattgttataaaaaattcagtgatggggatctgaagttgcctgattatttctggc

agccgaattttacttcgagccacccgatccaatgcagttggcgcgccattcgatatcgga

ccctttccctttagtgagggttaatgct

>ACA43_SynSno_5nt

acctttgccaagccaatggcggctaactatctgaagaaccagccgatgtacgtgttccgt

aagacggagctcaagcactccaagaccgagctcaacttcaaggagtggcaaaaggccttt

accgatgtgatgggcatggacgagctgtacaagtaaactagtagcagacagatgcgccgt

ggataaattgttataacgccaccattggtagcccgatgaagttgcctgattatttctggc

agccgaatttcatcacgagccacccgatccaatgcagttggcgcgccattcgatatcgga

ccctttccctttagtgagggttaatgct

>ACA44_s1_SynSno_5nt

acctttgccaagccaatggcggctaactatctgaagaaccagccgatgtacgtgttccgt

aagacggagctcaagcactccaagaccgagctcaacttcaaggagtggcaaaaggccttt

accgatgtgatgggcatggacgagctgtacaagtaaactagtagcagacagatgcgccgt

ggataaattgttatagtactttggatgttggcccacggaagttgcctgattatttctggc

agccgaattacacaatgagccacccgatccaatgcagttggcgcgccattcgatatcgga

ccctttccctttagtgagggttaatgct

>ACA44_s2_SynSno_5nt

acctttgccaagccaatggcggctaactatctgaagaaccagccgatgtacgtgttccgt

aagacggagctcaagcactccaagaccgagctcaacttcaaggagtggcaaaaggccttt

accgatgtgatgggcatggacgagctgtacaagtaaactagtagcagacagatgcgccgt

ggataaattgttatacctaggagtgtgcaaatgaggggaagttgcctgattatttctggc

agccgaattatttcaacagccacccgatccaatgcagttggcgcgccattcgatatcgga

ccctttccctttagtgagggttaatgct

>ACA46_SynSno_5nt

acctttgccaagccaatggcggctaactatctgaagaaccagccgatgtacgtgttccgt

aagacggagctcaagcactccaagaccgagctcaacttcaaggagtggcaaaaggccttt

accgatgtgatgggcatggacgagctgtacaagtaaactagtagcagacagatgcgccgt

ggataaattgttataagcacgcgtatgttaacgcgctgaagttgcctgattatttctggc

agccgaattcaccatttagccacccgatccaatgcagttggcgcgccattcgatatcgga

ccctttccctttagtgagggttaatgct

>ACA48_SynSno_5nt

acctttgccaagccaatggcggctaactatctgaagaaccagccgatgtacgtgttccgt

aagacggagctcaagcactccaagaccgagctcaacttcaaggagtggcaaaaggccttt

accgatgtgatgggcatggacgagctgtacaagtaaactagtagcagacagatgcgccgt

ggataaattgttataccttaatgaatggatccccctagaagttgcctgattatttctggc

agccgaatttgaaatttagccacccgatccaatgcagttggcgcgccattcgatatcgga

ccctttccctttagtgagggttaatgct

>U19_up_SynSno_5nt

acctttgccaagccaatggcggctaactatctgaagaaccagccgatgtacgtgttccgt

aagacggagctcaagcactccaagaccgagctcaacttcaaggagtggcaaaaggccttt

accgatgtgatgggcatggacgagctgtacaagtaaactagtagcagacagatgcgccgt

ggataaattgttatacactggtaactgtgacccaatagaagttgcctgattatttctggc

agccgaattcgccatcgagccacccgatccaatgcagttggcgcgccattcgatatcgga

ccctttccctttagtgagggttaatgct

>U19_dn_SynSno_5nt

acctttgccaagccaatggcggctaactatctgaagaaccagccgatgtacgtgttccgt

aagacggagctcaagcactccaagaccgagctcaacttcaaggagtggcaaaaggccttt

accgatgtgatgggcatggacgagctgtacaagtaaactagtagcagacagatgcgccgt

ggataaattgttatacaaacgactatgctctactcttgaagttgcctgattatttctggc

agccgaattcggttttgagccacccgatccaatgcagttggcgcgccattcgatatcgga

ccctttccctttagtgagggttaatgct

>U23_up_SynSno_5nt

acctttgccaagccaatggcggctaactatctgaagaaccagccgatgtacgtgttccgt

aagacggagctcaagcactccaagaccgagctcaacttcaaggagtggcaaaaggccttt

accgatgtgatgggcatggacgagctgtacaagtaaactagtagcagacagatgcgccgt

ggataaattgttataagatggcgaatggctcccgtctgaagttgcctgattatttctggc

agccgaattactccagcagccacccgatccaatgcagttggcgcgccattcgatatcgga

ccctttccctttagtgagggttaatgct

>U64_up_SynSno_5nt

acctttgccaagccaatggcggctaactatctgaagaaccagccgatgtacgtgttccgt

aagacggagctcaagcactccaagaccgagctcaacttcaaggagtggcaaaaggccttt

accgatgtgatgggcatggacgagctgtacaagtaaactagtagcagacagatgcgccgt

ggataaattgttataaaaatttacgtggcagctaatcgaagttgcctgattatttctggc

agccgaattcatagcagagccacccgatccaatgcagttggcgcgccattcgatatcgga

ccctttccctttagtgagggttaatgct

>U65_up_SynSno_5nt

acctttgccaagccaatggcggctaactatctgaagaaccagccgatgtacgtgttccgt

aagacggagctcaagcactccaagaccgagctcaacttcaaggagtggcaaaaggccttt

accgatgtgatgggcatggacgagctgtacaagtaaactagtagcagacagatgcgccgt

ggataaattgttataacaacctggatggtggatttgtgaagttgcctgattatttctggc

agccgaatttggatcacagccacccgatccaatgcagttggcgcgccattcgatatcgga

ccctttccctttagtgagggttaatgct

>U65_dn_SynSno_5nt

acctttgccaagccaatggcggctaactatctgaagaaccagccgatgtacgtgttccgt

aagacggagctcaagcactccaagaccgagctcaacttcaaggagtggcaaaaggccttt

accgatgtgatgggcatggacgagctgtacaagtaaactagtagcagacagatgcgccgt

ggataaattgttatagtaggtcggctgttccgcctaggaagttgcctgattatttctggc

agccgaattccgtagatagccacccgatccaatgcagttggcgcgccattcgatatcgga

ccctttccctttagtgagggttaatgct

>U66_up_SynSno_5nt

acctttgccaagccaatggcggctaactatctgaagaaccagccgatgtacgtgttccgt

aagacggagctcaagcactccaagaccgagctcaacttcaaggagtggcaaaaggccttt

accgatgtgatgggcatggacgagctgtacaagtaaactagtagcagacagatgcgccgt

ggataaattgttatacaagtgttcctgtgatatctgggaagttgcctgattatttctggc

agccgaattagacccaaagccacccgatccaatgcagttggcgcgccattcgatatcgga

ccctttccctttagtgagggttaatgct

>U68_up_SynSno_5nt

acctttgccaagccaatggcggctaactatctgaagaaccagccgatgtacgtgttccgt

aagacggagctcaagcactccaagaccgagctcaacttcaaggagtggcaaaaggccttt

accgatgtgatgggcatggacgagctgtacaagtaaactagtagcagacagatgcgccgt

ggataaattgttatagaaccattcatggcgaacctcggaagttgcctgattatttctggc

agccgaattactattaaagccacccgatccaatgcagttggcgcgccattcgatatcgga

ccctttccctttagtgagggttaatgct

>U69_up_SynSno_5nt

acctttgccaagccaatggcggctaactatctgaagaaccagccgatgtacgtgttccgt

aagacggagctcaagcactccaagaccgagctcaacttcaaggagtggcaaaaggccttt

accgatgtgatgggcatggacgagctgtacaagtaaactagtagcagacagatgcgccgt

ggataaattgttataacttctaatgtgaatttaccaagaagttgcctgattatttctggc

agccgaattgcgagatgagccacccgatccaatgcagttggcgcgccattcgatatcgga

ccctttccctttagtgagggttaatgct

>U69_dn_SynSno_5nt

acctttgccaagccaatggcggctaactatctgaagaaccagccgatgtacgtgttccgt

aagacggagctcaagcactccaagaccgagctcaacttcaaggagtggcaaaaggccttt

accgatgtgatgggcatggacgagctgtacaagtaaactagtagcagacagatgcgccgt

ggataaattgttatagaccgctgtctgaaaacgtcgggaagttgcctgattatttctggc

agccgaattccggccgcagccacccgatccaatgcagttggcgcgccattcgatatcgga

ccctttccctttagtgagggttaatgct

>U70_up_SynSno_5nt

acctttgccaagccaatggcggctaactatctgaagaaccagccgatgtacgtgttccgt

aagacggagctcaagcactccaagaccgagctcaacttcaaggagtggcaaaaggccttt

accgatgtgatgggcatggacgagctgtacaagtaaactagtagcagacagatgcgccgt

ggataaattgttataccttgccctttgtacaagcaacgaagttgcctgattatttctggc

agccgaattctgaatacagccacccgatccaatgcagttggcgcgccattcgatatcgga

ccctttccctttagtgagggttaatgct

>U71a_up_SynSno_5nt

acctttgccaagccaatggcggctaactatctgaagaaccagccgatgtacgtgttccgt

aagacggagctcaagcactccaagaccgagctcaacttcaaggagtggcaaaaggccttt

accgatgtgatgggcatggacgagctgtacaagtaaactagtagcagacagatgcgccgt

ggataaattgttatacctagtcaggtgacggttcaccgaagttgcctgattatttctggc

agccgaattccgtgacgagccacccgatccaatgcagttggcgcgccattcgatatcgga

ccctttccctttagtgagggttaatgct

>U72_up_SynSno_5nt

acctttgccaagccaatggcggctaactatctgaagaaccagccgatgtacgtgttccgt

aagacggagctcaagcactccaagaccgagctcaacttcaaggagtggcaaaaggccttt

accgatgtgatgggcatggacgagctgtacaagtaaactagtagcagacagatgcgccgt

ggataaattgttataccgtgttagctgcgagccgcgggaagttgcctgattatttctggc

agccgaattggatcacgagccacccgatccaatgcagttggcgcgccattcgatatcgga

ccctttccctttagtgagggttaatgct

>U99_up_SynSno_5nt

acctttgccaagccaatggcggctaactatctgaagaaccagccgatgtacgtgttccgt

aagacggagctcaagcactccaagaccgagctcaacttcaaggagtggcaaaaggccttt

accgatgtgatgggcatggacgagctgtacaagtaaactagtagcagacagatgcgccgt

ggataaattgttatagaatgacatttgccaatcccctgaagttgcctgattatttctggc

agccgaattcccaagcgagccacccgatccaatgcagttggcgcgccattcgatatcgga

ccctttccctttagtgagggttaatgct

>ACA5_s1_SynSno_5nt

acctttgccaagccaatggcggctaactatctgaagaaccagccgatgtacgtgttccgt

aagacggagctcaagcactccaagaccgagctcaacttcaaggagtggcaaaaggccttt

accgatgtgatgggcatggacgagctgtacaagtaaactagtagcagacagatgcgccgt

ggataaattgttataaaggatgggctgaattgtcacagaagttgcctgattatttctggc

agccgaattcttcaattagccacccgatccaatgcagttggcgcgccattcgatatcgga

ccctttccctttagtgagggttaatgct

>ACA5_s2_SynSno_5nt

acctttgccaagccaatggcggctaactatctgaagaaccagccgatgtacgtgttccgt

aagacggagctcaagcactccaagaccgagctcaacttcaaggagtggcaaaaggccttt

accgatgtgatgggcatggacgagctgtacaagtaaactagtagcagacagatgcgccgt

ggataaattgttataaaccctttattgcccagtctgagaagttgcctgattatttctggc

agccgaattagtagctcagccacccgatccaatgcagttggcgcgccattcgatatcgga

ccctttccctttagtgagggttaatgct

>ACA50_s1_SynSno_5nt

acctttgccaagccaatggcggctaactatctgaagaaccagccgatgtacgtgttccgt

aagacggagctcaagcactccaagaccgagctcaacttcaaggagtggcaaaaggccttt

accgatgtgatgggcatggacgagctgtacaagtaaactagtagcagacagatgcgccgt

ggataaattgttataagtgggcttgtgtcaacttactgaagttgcctgattatttctggc

agccgaatttttcacggagccacccgatccaatgcagttggcgcgccattcgatatcgga

ccctttccctttagtgagggttaatgct

>ACA50_s2_SynSno_5nt

acctttgccaagccaatggcggctaactatctgaagaaccagccgatgtacgtgttccgt

aagacggagctcaagcactccaagaccgagctcaacttcaaggagtggcaaaaggccttt

accgatgtgatgggcatggacgagctgtacaagtaaactagtagcagacagatgcgccgt

ggataaattgttatagtgggttaaatgagttcgccacgaagttgcctgattatttctggc

agccgaattcgacccccagccacccgatccaatgcagttggcgcgccattcgatatcgga

ccctttccctttagtgagggttaatgct

>ACA52_SynSno_5nt

acctttgccaagccaatggcggctaactatctgaagaaccagccgatgtacgtgttccgt

aagacggagctcaagcactccaagaccgagctcaacttcaaggagtggcaaaaggccttt

accgatgtgatgggcatggacgagctgtacaagtaaactagtagcagacagatgcgccgt

ggataaattgttataacaccgcgaatgattattcgttgaagttgcctgattatttctggc

agccgaattttcggcgaagccacccgatccaatgcagttggcgcgccattcgatatcgga

ccctttccctttagtgagggttaatgct

>ACA54_s1_SynSno_5nt

acctttgccaagccaatggcggctaactatctgaagaaccagccgatgtacgtgttccgt

aagacggagctcaagcactccaagaccgagctcaacttcaaggagtggcaaaaggccttt

accgatgtgatgggcatggacgagctgtacaagtaaactagtagcagacagatgcgccgt

ggataaattgttataaattcatggatgtacgccactggaagttgcctgattatttctggc

agccgaattagagttttagccacccgatccaatgcagttggcgcgccattcgatatcgga

ccctttccctttagtgagggttaatgct

>ACA54_s2_SynSno_5nt

acctttgccaagccaatggcggctaactatctgaagaaccagccgatgtacgtgttccgt

aagacggagctcaagcactccaagaccgagctcaacttcaaggagtggcaaaaggccttt

accgatgtgatgggcatggacgagctgtacaagtaaactagtagcagacagatgcgccgt

ggataaattgttataagtccactgatgatgttgataggaagttgcctgattatttctggc

agccgaattactgctgcagccacccgatccaatgcagttggcgcgccattcgatatcgga

ccctttccctttagtgagggttaatgct

>ACA55_SynSno_5nt

acctttgccaagccaatggcggctaactatctgaagaaccagccgatgtacgtgttccgt

aagacggagctcaagcactccaagaccgagctcaacttcaaggagtggcaaaaggccttt

accgatgtgatgggcatggacgagctgtacaagtaaactagtagcagacagatgcgccgt

ggataaattgttatacaacgctgtctgaaagcggactgaagttgcctgattatttctggc

agccgaattagagttgaagccacccgatccaatgcagttggcgcgccattcgatatcgga

ccctttccctttagtgagggttaatgct

>ACA56_SynSno_5nt

acctttgccaagccaatggcggctaactatctgaagaaccagccgatgtacgtgttccgt

aagacggagctcaagcactccaagaccgagctcaacttcaaggagtggcaaaaggccttt

accgatgtgatgggcatggacgagctgtacaagtaaactagtagcagacagatgcgccgt

ggataaattgttatacagtcctccctggggagccagtgaagttgcctgattatttctggc

agccgaattaaggttcaagccacccgatccaatgcagttggcgcgccattcgatatcgga

ccctttccctttagtgagggttaatgct

>ACA58_SynSno_5nt

acctttgccaagccaatggcggctaactatctgaagaaccagccgatgtacgtgttccgt

aagacggagctcaagcactccaagaccgagctcaacttcaaggagtggcaaaaggccttt

accgatgtgatgggcatggacgagctgtacaagtaaactagtagcagacagatgcgccgt

ggataaattgttatacacaggtccctgtctaatctgcgaagttgcctgattatttctggc

agccgaatttccatctaagccacccgatccaatgcagttggcgcgccattcgatatcgga

ccctttccctttagtgagggttaatgct

>ACA6_SynSno_5nt

acctttgccaagccaatggcggctaactatctgaagaaccagccgatgtacgtgttccgt

aagacggagctcaagcactccaagaccgagctcaacttcaaggagtggcaaaaggccttt

accgatgtgatgggcatggacgagctgtacaagtaaactagtagcagacagatgcgccgt

ggataaattgttataacaggccgactgtttacgctgtgaagttgcctgattatttctggc

agccgaatttttcccatagccacccgatccaatgcagttggcgcgccattcgatatcgga

ccctttccctttagtgagggttaatgct

>ACA60_SynSno_5nt

acctttgccaagccaatggcggctaactatctgaagaaccagccgatgtacgtgttccgt

aagacggagctcaagcactccaagaccgagctcaacttcaaggagtggcaaaaggccttt

accgatgtgatgggcatggacgagctgtacaagtaaactagtagcagacagatgcgccgt

ggataaattgttataataacgcatttgccaacacattgaagttgcctgattatttctggc

agccgaattccgaggttagccacccgatccaatgcagttggcgcgccattcgatatcgga

ccctttccctttagtgagggttaatgct

>ACA61_SynSno_5nt

acctttgccaagccaatggcggctaactatctgaagaaccagccgatgtacgtgttccgt

aagacggagctcaagcactccaagaccgagctcaacttcaaggagtggcaaaaggccttt

accgatgtgatgggcatggacgagctgtacaagtaaactagtagcagacagatgcgccgt

ggataaattgttataaagggtacgatggaaatttcgagaagttgcctgattatttctggc

agccgaattcgccaattagccacccgatccaatgcagttggcgcgccattcgatatcgga

ccctttccctttagtgagggttaatgct

>ACA62_s1_SynSno_5nt

acctttgccaagccaatggcggctaactatctgaagaaccagccgatgtacgtgttccgt

aagacggagctcaagcactccaagaccgagctcaacttcaaggagtggcaaaaggccttt

accgatgtgatgggcatggacgagctgtacaagtaaactagtagcagacagatgcgccgt

ggataaattgttataagtacgattgtgtcaactcactgaagttgcctgattatttctggc

agccgaattacggttgtagccacccgatccaatgcagttggcgcgccattcgatatcgga

ccctttccctttagtgagggttaatgct

>ACA62_s2_SynSno_5nt

acctttgccaagccaatggcggctaactatctgaagaaccagccgatgtacgtgttccgt

aagacggagctcaagcactccaagaccgagctcaacttcaaggagtggcaaaaggccttt

accgatgtgatgggcatggacgagctgtacaagtaaactagtagcagacagatgcgccgt

ggataaattgttataatattttaaatgagttcgtatagaagttgcctgattatttctggc

agccgaattgggtgtggagccacccgatccaatgcagttggcgcgccattcgatatcgga

ccctttccctttagtgagggttaatgct

>ACA63_SynSno_5nt

acctttgccaagccaatggcggctaactatctgaagaaccagccgatgtacgtgttccgt

aagacggagctcaagcactccaagaccgagctcaacttcaaggagtggcaaaaggccttt

accgatgtgatgggcatggacgagctgtacaagtaaactagtagcagacagatgcgccgt

ggataaattgttatatcactaaaaatgataggtctgagaagttgcctgattatttctggc

agccgaattattagtacagccacccgatccaatgcagttggcgcgccattcgatatcgga

ccctttccctttagtgagggttaatgct

>ACA64_SynSno_5nt

acctttgccaagccaatggcggctaactatctgaagaaccagccgatgtacgtgttccgt

aagacggagctcaagcactccaagaccgagctcaacttcaaggagtggcaaaaggccttt

accgatgtgatgggcatggacgagctgtacaagtaaactagtagcagacagatgcgccgt

ggataaattgttatataagtggttttgagcttcttaagaagttgcctgattatttctggc

agccgaattcacactgtagccacccgatccaatgcagttggcgcgccattcgatatcgga

ccctttccctttagtgagggttaatgct

>ACA65_s1_SynSno_5nt

acctttgccaagccaatggcggctaactatctgaagaaccagccgatgtacgtgttccgt

aagacggagctcaagcactccaagaccgagctcaacttcaaggagtggcaaaaggccttt

accgatgtgatgggcatggacgagctgtacaagtaaactagtagcagacagatgcgccgt

ggataaattgttatagtcaataaaatgagaaactgacgaagttgcctgattatttctggc

agccgaattacatcaggagccacccgatccaatgcagttggcgcgccattcgatatcgga

ccctttccctttagtgagggttaatgct

>ACA65_s2_SynSno_5nt

acctttgccaagccaatggcggctaactatctgaagaaccagccgatgtacgtgttccgt

aagacggagctcaagcactccaagaccgagctcaacttcaaggagtggcaaaaggccttt

accgatgtgatgggcatggacgagctgtacaagtaaactagtagcagacagatgcgccgt

ggataaattgttataggaatcaaattggtgactccccgaagttgcctgattatttctggc

agccgaattgaactcggagccacccgatccaatgcagttggcgcgccattcgatatcgga

ccctttccctttagtgagggttaatgct

>ACA67_s1_SynSno_5nt

acctttgccaagccaatggcggctaactatctgaagaaccagccgatgtacgtgttccgt

aagacggagctcaagcactccaagaccgagctcaacttcaaggagtggcaaaaggccttt

accgatgtgatgggcatggacgagctgtacaagtaaactagtagcagacagatgcgccgt

ggataaattgttatacgtttgtacttgaaataacccagaagttgcctgattatttctggc

agccgaattccgaacggagccacccgatccaatgcagttggcgcgccattcgatatcgga

ccctttccctttagtgagggttaatgct

>ACA67_s2_SynSno_5nt

acctttgccaagccaatggcggctaactatctgaagaaccagccgatgtacgtgttccgt

aagacggagctcaagcactccaagaccgagctcaacttcaaggagtggcaaaaggccttt

accgatgtgatgggcatggacgagctgtacaagtaaactagtagcagacagatgcgccgt

ggataaattgttataaaacattcagtgatggggacttgaagttgcctgattatttctggc

agccgaattgagaaatgagccacccgatccaatgcagttggcgcgccattcgatatcgga

ccctttccctttagtgagggttaatgct

>ACA7_s1_SynSno_5nt

acctttgccaagccaatggcggctaactatctgaagaaccagccgatgtacgtgttccgt

aagacggagctcaagcactccaagaccgagctcaacttcaaggagtggcaaaaggccttt

accgatgtgatgggcatggacgagctgtacaagtaaactagtagcagacagatgcgccgt

ggataaattgttatagaaagttccgtggcgagaaactgaagttgcctgattatttctggc

agccgaatttacgttctagccacccgatccaatgcagttggcgcgccattcgatatcgga

ccctttccctttagtgagggttaatgct

>ACA7_s2_SynSno_5nt

acctttgccaagccaatggcggctaactatctgaagaaccagccgatgtacgtgttccgt

aagacggagctcaagcactccaagaccgagctcaacttcaaggagtggcaaaaggccttt

accgatgtgatgggcatggacgagctgtacaagtaaactagtagcagacagatgcgccgt

ggataaattgttatactctggtctttgaatgttgtaagaagttgcctgattatttctggc

agccgaattcggatgacagccacccgatccaatgcagttggcgcgccattcgatatcgga

ccctttccctttagtgagggttaatgct

>ACA8_s1_SynSno_5nt

acctttgccaagccaatggcggctaactatctgaagaaccagccgatgtacgtgttccgt

aagacggagctcaagcactccaagaccgagctcaacttcaaggagtggcaaaaggccttt

accgatgtgatgggcatggacgagctgtacaagtaaactagtagcagacagatgcgccgt

ggataaattgttatagtcaatttgatgagatcaacgtgaagttgcctgattatttctggc

agccgaatttagtgggtagccacccgatccaatgcagttggcgcgccattcgatatcgga

ccctttccctttagtgagggttaatgct

>ACA8_s2_SynSno_5nt

acctttgccaagccaatggcggctaactatctgaagaaccagccgatgtacgtgttccgt

aagacggagctcaagcactccaagaccgagctcaacttcaaggagtggcaaaaggccttt

accgatgtgatgggcatggacgagctgtacaagtaaactagtagcagacagatgcgccgt

ggataaattgttatactccctaccatgcaactcgtaagaagttgcctgattatttctggc

agccgaattacacgcaaagccacccgatccaatgcagttggcgcgccattcgatatcgga

ccctttccctttagtgagggttaatgct

>ACA9_s1_SynSno_5nt

acctttgccaagccaatggcggctaactatctgaagaaccagccgatgtacgtgttccgt

aagacggagctcaagcactccaagaccgagctcaacttcaaggagtggcaaaaggccttt

accgatgtgatgggcatggacgagctgtacaagtaaactagtagcagacagatgcgccgt

ggataaattgttataacagtaaggatggctgtcttaggaagttgcctgattatttctggc

agccgaattacgggtctagccacccgatccaatgcagttggcgcgccattcgatatcgga

ccctttccctttagtgagggttaatgct

>ACA9_s2_SynSno_5nt

acctttgccaagccaatggcggctaactatctgaagaaccagccgatgtacgtgttccgt

aagacggagctcaagcactccaagaccgagctcaacttcaaggagtggcaaaaggccttt

accgatgtgatgggcatggacgagctgtacaagtaaactagtagcagacagatgcgccgt

ggataaattgttatacccggtctattgtcaacaatgggaagttgcctgattatttctggc

agccgaattttacgtgcagccacccgatccaatgcagttggcgcgccattcgatatcgga

ccctttccctttagtgagggttaatgct

>E2_s1_SynSno_5nt

acctttgccaagccaatggcggctaactatctgaagaaccagccgatgtacgtgttccgt

aagacggagctcaagcactccaagaccgagctcaacttcaaggagtggcaaaaggccttt

accgatgtgatgggcatggacgagctgtacaagtaaactagtagcagacagatgcgccgt

ggataaattgttataacagccctactgtctacaccgtgaagttgcctgattatttctggc

agccgaattaaggtgtcagccacccgatccaatgcagttggcgcgccattcgatatcgga

ccctttccctttagtgagggttaatgct

>E2_s2_SynSno_5nt

acctttgccaagccaatggcggctaactatctgaagaaccagccgatgtacgtgttccgt

aagacggagctcaagcactccaagaccgagctcaacttcaaggagtggcaaaaggccttt

accgatgtgatgggcatggacgagctgtacaagtaaactagtagcagacagatgcgccgt

ggataaattgttatactccgtactatgtagctccccagaagttgcctgattatttctggc

agccgaattcacgcgaaagccacccgatccaatgcagttggcgcgccattcgatatcgga

ccctttccctttagtgagggttaatgct

>E3_SynSno_5nt

acctttgccaagccaatggcggctaactatctgaagaaccagccgatgtacgtgttccgt

aagacggagctcaagcactccaagaccgagctcaacttcaaggagtggcaaaaggccttt

accgatgtgatgggcatggacgagctgtacaagtaaactagtagcagacagatgcgccgt

ggataaattgttatatcaacagcgttgatagggcctcgaagttgcctgattatttctggc

agccgaattgtttgtgaagccacccgatccaatgcagttggcgcgccattcgatatcgga

ccctttccctttagtgagggttaatgct

>HBI-115_SynSno_5nt

acctttgccaagccaatggcggctaactatctgaagaaccagccgatgtacgtgttccgt

aagacggagctcaagcactccaagaccgagctcaacttcaaggagtggcaaaaggccttt

accgatgtgatgggcatggacgagctgtacaagtaaactagtagcagacagatgcgccgt

ggataaattgttatattgagtaacctgttctactgaagaagttgcctgattatttctggc

agccgaattggaagcgtagccacccgatccaatgcagttggcgcgccattcgatatcgga

ccctttccctttagtgagggttaatgct

>HBI-6_SynSno_5nt

acctttgccaagccaatggcggctaactatctgaagaaccagccgatgtacgtgttccgt

aagacggagctcaagcactccaagaccgagctcaacttcaaggagtggcaaaaggccttt

accgatgtgatgggcatggacgagctgtacaagtaaactagtagcagacagatgcgccgt

ggataaattgttataactcaaggattggtttgcgttcgaagttgcctgattatttctggc

agccgaattagccgtgcagccacccgatccaatgcagttggcgcgccattcgatatcgga

ccctttccctttagtgagggttaatgct

>HBI-61_SynSno_5nt

acctttgccaagccaatggcggctaactatctgaagaaccagccgatgtacgtgttccgt

aagacggagctcaagcactccaagaccgagctcaacttcaaggagtggcaaaaggccttt

accgatgtgatgggcatggacgagctgtacaagtaaactagtagcagacagatgcgccgt

ggataaattgttataaggtggtgtatgtgctgcgcacgaagttgcctgattatttctggc

agccgaattcaggcgcaagccacccgatccaatgcagttggcgcgccattcgatatcgga

ccctttccctttagtgagggttaatgct

>ACA1_SynSno_3nt

acctttgccaagccaatggcggctaactatctgaagaaccagccgatgtacgtgttccgt

aagacggagctcaagcactccaagaccgagctcaacttcaaggagtggcaaaaggccttt

accgatgtgatgggcatggacgagctgtacaagtaaactagtagcagacagatgcgccgt

ggataaattgttatattgagacttgtgaatacgccaagaagttgcctgattatttctggc

agccgaattaacagccaagccacccgatccaatgcagttggcgcgccattcgatatcgga

ccctttccctttagtgagggttaatgct

>ACA10_s1_SynSno_3nt

acctttgccaagccaatggcggctaactatctgaagaaccagccgatgtacgtgttccgt

aagacggagctcaagcactccaagaccgagctcaacttcaaggagtggcaaaaggccttt

accgatgtgatgggcatggacgagctgtacaagtaaactagtagcagacagatgcgccgt

ggataaattgttataaagtcccacatgagagtctctcgaagttgcctgattatttctggc

agccgaattataaatagagccacccgatccaatgcagttggcgcgccattcgatatcgga

ccctttccctttagtgagggttaatgct

>ACA10_s2_SynSno_3nt

acctttgccaagccaatggcggctaactatctgaagaaccagccgatgtacgtgttccgt

aagacggagctcaagcactccaagaccgagctcaacttcaaggagtggcaaaaggccttt

accgatgtgatgggcatggacgagctgtacaagtaaactagtagcagacagatgcgccgt

ggataaattgttatacagaagcggatgcggtacggctgaagttgcctgattatttctggc

agccgaattggtttacaagccacccgatccaatgcagttggcgcgccattcgatatcgga

ccctttccctttagtgagggttaatgct

>ACA13_SynSno_3nt

acctttgccaagccaatggcggctaactatctgaagaaccagccgatgtacgtgttccgt

aagacggagctcaagcactccaagaccgagctcaacttcaaggagtggcaaaaggccttt

accgatgtgatgggcatggacgagctgtacaagtaaactagtagcagacagatgcgccgt

ggataaattgttataagggggggactgaaacacccttgaagttgcctgattatttctggc

agccgaattgaaaccgcagccacccgatccaatgcagttggcgcgccattcgatatcgga

ccctttccctttagtgagggttaatgct

>ACA14a_SynSno_3nt

acctttgccaagccaatggcggctaactatctgaagaaccagccgatgtacgtgttccgt

aagacggagctcaagcactccaagaccgagctcaacttcaaggagtggcaaaaggccttt

accgatgtgatgggcatggacgagctgtacaagtaaactagtagcagacagatgcgccgt

ggataaattgttatattgcgtcaattgttttcagggagaagttgcctgattatttctggc

agccgaatttggcccctagccacccgatccaatgcagttggcgcgccattcgatatcgga

ccctttccctttagtgagggttaatgct

>ACA15_SynSno_3nt

acctttgccaagccaatggcggctaactatctgaagaaccagccgatgtacgtgttccgt

aagacggagctcaagcactccaagaccgagctcaacttcaaggagtggcaaaaggccttt

accgatgtgatgggcatggacgagctgtacaagtaaactagtagcagacagatgcgccgt

ggataaattgttatagttgtgatggtgtaggattaacgaagttgcctgattatttctggc

agccgaattacagtgcaagccacccgatccaatgcagttggcgcgccattcgatatcgga

ccctttccctttagtgagggttaatgct

>ACA16_SynSno_3nt

acctttgccaagccaatggcggctaactatctgaagaaccagccgatgtacgtgttccgt

aagacggagctcaagcactccaagaccgagctcaacttcaaggagtggcaaaaggccttt

accgatgtgatgggcatggacgagctgtacaagtaaactagtagcagacagatgcgccgt

ggataaattgttatagaaggggtgatgctgatcgcctgaagttgcctgattatttctggc

agccgaattaatcaccaagccacccgatccaatgcagttggcgcgccattcgatatcgga

ccctttccctttagtgagggttaatgct

>ACA17_s1_SynSno_3nt

acctttgccaagccaatggcggctaactatctgaagaaccagccgatgtacgtgttccgt

aagacggagctcaagcactccaagaccgagctcaacttcaaggagtggcaaaaggccttt

accgatgtgatgggcatggacgagctgtacaagtaaactagtagcagacagatgcgccgt

ggataaattgttatacaagccggactgaaataagagcgaagttgcctgattatttctggc

agccgaattcgttgaccagccacccgatccaatgcagttggcgcgccattcgatatcgga

ccctttccctttagtgagggttaatgct

>ACA17_s2_SynSno_3nt

acctttgccaagccaatggcggctaactatctgaagaaccagccgatgtacgtgttccgt

aagacggagctcaagcactccaagaccgagctcaacttcaaggagtggcaaaaggccttt

accgatgtgatgggcatggacgagctgtacaagtaaactagtagcagacagatgcgccgt

ggataaattgttatagcgaccccattgagatgctcacgaagttgcctgattatttctggc

agccgaattagatgcgtagccacccgatccaatgcagttggcgcgccattcgatatcgga

ccctttccctttagtgagggttaatgct

>ACA19_s1_SynSno_3nt

acctttgccaagccaatggcggctaactatctgaagaaccagccgatgtacgtgttccgt

aagacggagctcaagcactccaagaccgagctcaacttcaaggagtggcaaaaggccttt

accgatgtgatgggcatggacgagctgtacaagtaaactagtagcagacagatgcgccgt

ggataaattgttataacgcctcaattgaagtcccgtggaagttgcctgattatttctggc

agccgaattcttacagaagccacccgatccaatgcagttggcgcgccattcgatatcgga

ccctttccctttagtgagggttaatgct

>ACA19_s2_SynSno_3nt

acctttgccaagccaatggcggctaactatctgaagaaccagccgatgtacgtgttccgt

aagacggagctcaagcactccaagaccgagctcaacttcaaggagtggcaaaaggccttt

accgatgtgatgggcatggacgagctgtacaagtaaactagtagcagacagatgcgccgt

ggataaattgttataccttctcctgtgtacggctaaggaagttgcctgattatttctggc

agccgaattgatataagagccacccgatccaatgcagttggcgcgccattcgatatcgga

ccctttccctttagtgagggttaatgct

>ACA19_s3_SynSno_3nt

acctttgccaagccaatggcggctaactatctgaagaaccagccgatgtacgtgttccgt

aagacggagctcaagcactccaagaccgagctcaacttcaaggagtggcaaaaggccttt

accgatgtgatgggcatggacgagctgtacaagtaaactagtagcagacagatgcgccgt

ggataaattgttataacacgccgaatgattcccgtgtgaagttgcctgattatttctggc

agccgaatttaggtgggagccacccgatccaatgcagttggcgcgccattcgatatcgga

ccctttccctttagtgagggttaatgct

>ACA20_SynSno_3nt

acctttgccaagccaatggcggctaactatctgaagaaccagccgatgtacgtgttccgt

aagacggagctcaagcactccaagaccgagctcaacttcaaggagtggcaaaaggccttt

accgatgtgatgggcatggacgagctgtacaagtaaactagtagcagacagatgcgccgt

ggataaattgttataacgtatgatatgaacgcccgttgaagttgcctgattatttctggc

agccgaattctccctatagccacccgatccaatgcagttggcgcgccattcgatatcgga

ccctttccctttagtgagggttaatgct

>ACA21_s1_SynSno_3nt

acctttgccaagccaatggcggctaactatctgaagaaccagccgatgtacgtgttccgt

aagacggagctcaagcactccaagaccgagctcaacttcaaggagtggcaaaaggccttt

accgatgtgatgggcatggacgagctgtacaagtaaactagtagcagacagatgcgccgt

ggataaattgttatagggaggtacgtggcggggccccgaagttgcctgattatttctggc

agccgaattaaaactgtagccacccgatccaatgcagttggcgcgccattcgatatcgga

ccctttccctttagtgagggttaatgct

>ACA21_s2_SynSno_3nt

acctttgccaagccaatggcggctaactatctgaagaaccagccgatgtacgtgttccgt

aagacggagctcaagcactccaagaccgagctcaacttcaaggagtggcaaaaggccttt

accgatgtgatgggcatggacgagctgtacaagtaaactagtagcagacagatgcgccgt

ggataaattgttataaacaatcttgtgcaaaacccctgaagttgcctgattatttctggc

agccgaattggtcatcaagccacccgatccaatgcagttggcgcgccattcgatatcgga

ccctttccctttagtgagggttaatgct

>ACA22_s1_SynSno_3nt

acctttgccaagccaatggcggctaactatctgaagaaccagccgatgtacgtgttccgt

aagacggagctcaagcactccaagaccgagctcaacttcaaggagtggcaaaaggccttt

accgatgtgatgggcatggacgagctgtacaagtaaactagtagcagacagatgcgccgt

ggataaattgttatactttgacaagtgtccagtgtacgaagttgcctgattatttctggc

agccgaattcggtggggagccacccgatccaatgcagttggcgcgccattcgatatcgga

ccctttccctttagtgagggttaatgct

>ACA22_s2_SynSno_3nt

acctttgccaagccaatggcggctaactatctgaagaaccagccgatgtacgtgttccgt

aagacggagctcaagcactccaagaccgagctcaacttcaaggagtggcaaaaggccttt

accgatgtgatgggcatggacgagctgtacaagtaaactagtagcagacagatgcgccgt

ggataaattgttatagagtccgacatggcctcttctcgaagttgcctgattatttctggc

agccgaatttaaacggaagccacccgatccaatgcagttggcgcgccattcgatatcgga

ccctttccctttagtgagggttaatgct

>ACA23_s1_SynSno_3nt

acctttgccaagccaatggcggctaactatctgaagaaccagccgatgtacgtgttccgt

aagacggagctcaagcactccaagaccgagctcaacttcaaggagtggcaaaaggccttt

accgatgtgatgggcatggacgagctgtacaagtaaactagtagcagacagatgcgccgt

ggataaattgttataacgtcgtttttgagactaacgtgaagttgcctgattatttctggc

agccgaattcacaccggagccacccgatccaatgcagttggcgcgccattcgatatcgga

ccctttccctttagtgagggttaatgct

>ACA23_s2_SynSno_3nt

acctttgccaagccaatggcggctaactatctgaagaaccagccgatgtacgtgttccgt

aagacggagctcaagcactccaagaccgagctcaacttcaaggagtggcaaaaggccttt

accgatgtgatgggcatggacgagctgtacaagtaaactagtagcagacagatgcgccgt

ggataaattgttatatccgggtgagtgacgcgtcgaagaagttgcctgattatttctggc

agccgaattaagtggctagccacccgatccaatgcagttggcgcgccattcgatatcgga

ccctttccctttagtgagggttaatgct

>ACA24_s1_SynSno_3nt

acctttgccaagccaatggcggctaactatctgaagaaccagccgatgtacgtgttccgt

aagacggagctcaagcactccaagaccgagctcaacttcaaggagtggcaaaaggccttt

accgatgtgatgggcatggacgagctgtacaagtaaactagtagcagacagatgcgccgt

ggataaattgttatagttagctgaatgaactcgcacggaagttgcctgattatttctggc

agccgaatttaaccgctagccacccgatccaatgcagttggcgcgccattcgatatcgga

ccctttccctttagtgagggttaatgct

>ACA24_s2_SynSno_3nt

acctttgccaagccaatggcggctaactatctgaagaaccagccgatgtacgtgttccgt

aagacggagctcaagcactccaagaccgagctcaacttcaaggagtggcaaaaggccttt

accgatgtgatgggcatggacgagctgtacaagtaaactagtagcagacagatgcgccgt

ggataaattgttatagtgtaccgtctgtttaaccgccgaagttgcctgattatttctggc

agccgaattcccgactgagccacccgatccaatgcagttggcgcgccattcgatatcgga

ccctttccctttagtgagggttaatgct

>ACA25_s2_SynSno_3nt

acctttgccaagccaatggcggctaactatctgaagaaccagccgatgtacgtgttccgt

aagacggagctcaagcactccaagaccgagctcaacttcaaggagtggcaaaaggccttt

accgatgtgatgggcatggacgagctgtacaagtaaactagtagcagacagatgcgccgt

ggataaattgttataagacttcaaatgggctgtagcggaagttgcctgattatttctggc

agccgaatttacgagtcagccacccgatccaatgcagttggcgcgccattcgatatcgga

ccctttccctttagtgagggttaatgct

>ACA27_s1_SynSno_3nt

acctttgccaagccaatggcggctaactatctgaagaaccagccgatgtacgtgttccgt

aagacggagctcaagcactccaagaccgagctcaacttcaaggagtggcaaaaggccttt

accgatgtgatgggcatggacgagctgtacaagtaaactagtagcagacagatgcgccgt

ggataaattgttataacaggtcatgtgaactgtccccgaagttgcctgattatttctggc

agccgaattttttcttaagccacccgatccaatgcagttggcgcgccattcgatatcgga

ccctttccctttagtgagggttaatgct

>ACA27_s2_SynSno_3nt

acctttgccaagccaatggcggctaactatctgaagaaccagccgatgtacgtgttccgt

aagacggagctcaagcactccaagaccgagctcaacttcaaggagtggcaaaaggccttt

accgatgtgatgggcatggacgagctgtacaagtaaactagtagcagacagatgcgccgt

ggataaattgttatagagtcacggatggtgggcccctgaagttgcctgattatttctggc

agccgaattgtgaagtcagccacccgatccaatgcagttggcgcgccattcgatatcgga

ccctttccctttagtgagggttaatgct

>ACA28_s1_SynSno_3nt

acctttgccaagccaatggcggctaactatctgaagaaccagccgatgtacgtgttccgt

aagacggagctcaagcactccaagaccgagctcaacttcaaggagtggcaaaaggccttt

accgatgtgatgggcatggacgagctgtacaagtaaactagtagcagacagatgcgccgt

ggataaattgttatacctagacaattggatgtggtaggaagttgcctgattatttctggc

agccgaattacttggggagccacccgatccaatgcagttggcgcgccattcgatatcgga

ccctttccctttagtgagggttaatgct

>ACA28_s2_SynSno_3nt

acctttgccaagccaatggcggctaactatctgaagaaccagccgatgtacgtgttccgt

aagacggagctcaagcactccaagaccgagctcaacttcaaggagtggcaaaaggccttt

accgatgtgatgggcatggacgagctgtacaagtaaactagtagcagacagatgcgccgt

ggataaattgttatatgagcgctaatggacgcttggcgaagttgcctgattatttctggc

agccgaatttcgctttaagccacccgatccaatgcagttggcgcgccattcgatatcgga

ccctttccctttagtgagggttaatgct

>ACA2a_s1_SynSno_3nt

acctttgccaagccaatggcggctaactatctgaagaaccagccgatgtacgtgttccgt

aagacggagctcaagcactccaagaccgagctcaacttcaaggagtggcaaaaggccttt

accgatgtgatgggcatggacgagctgtacaagtaaactagtagcagacagatgcgccgt

ggataaattgttatagaagtggtgatgtttcggacttgaagttgcctgattatttctggc

agccgaattgccccaagagccacccgatccaatgcagttggcgcgccattcgatatcgga

ccctttccctttagtgagggttaatgct

>ACA2a_s2_SynSno_3nt

acctttgccaagccaatggcggctaactatctgaagaaccagccgatgtacgtgttccgt

aagacggagctcaagcactccaagaccgagctcaacttcaaggagtggcaaaaggccttt

accgatgtgatgggcatggacgagctgtacaagtaaactagtagcagacagatgcgccgt

ggataaattgttatagaaccaggaatgcatcaacaccgaagttgcctgattatttctggc

agccgaatttggttgtgagccacccgatccaatgcagttggcgcgccattcgatatcgga

ccctttccctttagtgagggttaatgct

>ACA3_s1_SynSno_3nt

acctttgccaagccaatggcggctaactatctgaagaaccagccgatgtacgtgttccgt

aagacggagctcaagcactccaagaccgagctcaacttcaaggagtggcaaaaggccttt

accgatgtgatgggcatggacgagctgtacaagtaaactagtagcagacagatgcgccgt

ggataaattgttatattcagagagctggaagagctaagaagttgcctgattatttctggc

agccgaattatccaataagccacccgatccaatgcagttggcgcgccattcgatatcgga

ccctttccctttagtgagggttaatgct

>ACA3_s2_SynSno_3nt

acctttgccaagccaatggcggctaactatctgaagaaccagccgatgtacgtgttccgt

aagacggagctcaagcactccaagaccgagctcaacttcaaggagtggcaaaaggccttt

accgatgtgatgggcatggacgagctgtacaagtaaactagtagcagacagatgcgccgt

ggataaattgttatagccaagtgtgtggacgccttgagaagttgcctgattatttctggc

agccgaatttccgtaccagccacccgatccaatgcagttggcgcgccattcgatatcgga

ccctttccctttagtgagggttaatgct

>ACA3-2_SynSno_3nt

acctttgccaagccaatggcggctaactatctgaagaaccagccgatgtacgtgttccgt

aagacggagctcaagcactccaagaccgagctcaacttcaaggagtggcaaaaggccttt

accgatgtgatgggcatggacgagctgtacaagtaaactagtagcagacagatgcgccgt

ggataaattgttatatgcagagagctggaagagctgagaagttgcctgattatttctggc

agccgaattcaacccatagccacccgatccaatgcagttggcgcgccattcgatatcgga

ccctttccctttagtgagggttaatgct

>ACA30_SynSno_3nt

acctttgccaagccaatggcggctaactatctgaagaaccagccgatgtacgtgttccgt

aagacggagctcaagcactccaagaccgagctcaacttcaaggagtggcaaaaggccttt

accgatgtgatgggcatggacgagctgtacaagtaaactagtagcagacagatgcgccgt

ggataaattgttataaactagcccatgtggtccctgtgaagttgcctgattatttctggc

agccgaattctccggccagccacccgatccaatgcagttggcgcgccattcgatatcgga

ccctttccctttagtgagggttaatgct

>ACA31_s1_SynSno_3nt

acctttgccaagccaatggcggctaactatctgaagaaccagccgatgtacgtgttccgt

aagacggagctcaagcactccaagaccgagctcaacttcaaggagtggcaaaaggccttt

accgatgtgatgggcatggacgagctgtacaagtaaactagtagcagacagatgcgccgt

ggataaattgttatagaagctggcatgtagactgttcgaagttgcctgattatttctggc

agccgaatttcggcaacagccacccgatccaatgcagttggcgcgccattcgatatcgga

ccctttccctttagtgagggttaatgct

>ACA31_s2_SynSno_3nt

acctttgccaagccaatggcggctaactatctgaagaaccagccgatgtacgtgttccgt

aagacggagctcaagcactccaagaccgagctcaacttcaaggagtggcaaaaggccttt

accgatgtgatgggcatggacgagctgtacaagtaaactagtagcagacagatgcgccgt

ggataaattgttataaaaccggcaatgaatatactttgaagttgcctgattatttctggc

agccgaattcggaaattagccacccgatccaatgcagttggcgcgccattcgatatcgga

ccctttccctttagtgagggttaatgct

>ACA32_SynSno_3nt

acctttgccaagccaatggcggctaactatctgaagaaccagccgatgtacgtgttccgt

aagacggagctcaagcactccaagaccgagctcaacttcaaggagtggcaaaaggccttt

accgatgtgatgggcatggacgagctgtacaagtaaactagtagcagacagatgcgccgt

ggataaattgttatagacgtgacactgttttgccgtcgaagttgcctgattatttctggc

agccgaattgcggacatagccacccgatccaatgcagttggcgcgccattcgatatcgga

ccctttccctttagtgagggttaatgct

>ACA33_SynSno_3nt

acctttgccaagccaatggcggctaactatctgaagaaccagccgatgtacgtgttccgt

aagacggagctcaagcactccaagaccgagctcaacttcaaggagtggcaaaaggccttt

accgatgtgatgggcatggacgagctgtacaagtaaactagtagcagacagatgcgccgt

ggataaattgttatacggagacgggtgtctgccacgtgaagttgcctgattatttctggc

agccgaatttcaccatcagccacccgatccaatgcagttggcgcgccattcgatatcgga

ccctttccctttagtgagggttaatgct

>ACA34_s1_SynSno_3nt

acctttgccaagccaatggcggctaactatctgaagaaccagccgatgtacgtgttccgt

aagacggagctcaagcactccaagaccgagctcaacttcaaggagtggcaaaaggccttt

accgatgtgatgggcatggacgagctgtacaagtaaactagtagcagacagatgcgccgt

ggataaattgttataagagtagtgatgttactgacttgaagttgcctgattatttctggc

agccgaattctaggctaagccacccgatccaatgcagttggcgcgccattcgatatcgga

ccctttccctttagtgagggttaatgct

>ACA34_s2_SynSno_3nt

acctttgccaagccaatggcggctaactatctgaagaaccagccgatgtacgtgttccgt

aagacggagctcaagcactccaagaccgagctcaacttcaaggagtggcaaaaggccttt

accgatgtgatgggcatggacgagctgtacaagtaaactagtagcagacagatgcgccgt

ggataaattgttatagaacccggaatgcatcaacactgaagttgcctgattatttctggc

agccgaatttcagagctagccacccgatccaatgcagttggcgcgccattcgatatcgga

ccctttccctttagtgagggttaatgct

>ACA36_s1_SynSno_3nt

acctttgccaagccaatggcggctaactatctgaagaaccagccgatgtacgtgttccgt

aagacggagctcaagcactccaagaccgagctcaacttcaaggagtggcaaaaggccttt

accgatgtgatgggcatggacgagctgtacaagtaaactagtagcagacagatgcgccgt

ggataaattgttatacctgggcatttgacgaccacaggaagttgcctgattatttctggc

agccgaattctccctttagccacccgatccaatgcagttggcgcgccattcgatatcgga

ccctttccctttagtgagggttaatgct

>ACA36_s2_SynSno_3nt

acctttgccaagccaatggcggctaactatctgaagaaccagccgatgtacgtgttccgt

aagacggagctcaagcactccaagaccgagctcaacttcaaggagtggcaaaaggccttt

accgatgtgatgggcatggacgagctgtacaagtaaactagtagcagacagatgcgccgt

ggataaattgttatatttcttaaaatgaagacgagaagaagttgcctgattatttctggc

agccgaattaaagcaatagccacccgatccaatgcagttggcgcgccattcgatatcgga

ccctttccctttagtgagggttaatgct

>ACA4_SynSno_3nt

acctttgccaagccaatggcggctaactatctgaagaaccagccgatgtacgtgttccgt

aagacggagctcaagcactccaagaccgagctcaacttcaaggagtggcaaaaggccttt

accgatgtgatgggcatggacgagctgtacaagtaaactagtagcagacagatgcgccgt

ggataaattgttatacaccccgagttggtttctgtgtgaagttgcctgattatttctggc

agccgaattcgacctagagccacccgatccaatgcagttggcgcgccattcgatatcgga

ccctttccctttagtgagggttaatgct

>ACA40_SynSno_3nt

acctttgccaagccaatggcggctaactatctgaagaaccagccgatgtacgtgttccgt

aagacggagctcaagcactccaagaccgagctcaacttcaaggagtggcaaaaggccttt

accgatgtgatgggcatggacgagctgtacaagtaaactagtagcagacagatgcgccgt

ggataaattgttataaggcgcggtatggtgtacccctgaagttgcctgattatttctggc

agccgaatttttcgaaaagccacccgatccaatgcagttggcgcgccattcgatatcgga

ccctttccctttagtgagggttaatgct

>ACA41_SynSno_3nt

acctttgccaagccaatggcggctaactatctgaagaaccagccgatgtacgtgttccgt

aagacggagctcaagcactccaagaccgagctcaacttcaaggagtggcaaaaggccttt

accgatgtgatgggcatggacgagctgtacaagtaaactagtagcagacagatgcgccgt

ggataaattgttatacactcttaattgccctgctagtgaagttgcctgattatttctggc

agccgaattccatgactagccacccgatccaatgcagttggcgcgccattcgatatcgga

ccctttccctttagtgagggttaatgct

>ACA42_s1_SynSno_3nt

acctttgccaagccaatggcggctaactatctgaagaaccagccgatgtacgtgttccgt

aagacggagctcaagcactccaagaccgagctcaacttcaaggagtggcaaaaggccttt

accgatgtgatgggcatggacgagctgtacaagtaaactagtagcagacagatgcgccgt

ggataaattgttatagtttgcaacttgaacgaacggcgaagttgcctgattatttctggc

agccgaatttgctatgaagccacccgatccaatgcagttggcgcgccattcgatatcgga

ccctttccctttagtgagggttaatgct

>ACA42_s2_SynSno_3nt

acctttgccaagccaatggcggctaactatctgaagaaccagccgatgtacgtgttccgt

aagacggagctcaagcactccaagaccgagctcaacttcaaggagtggcaaaaggccttt

accgatgtgatgggcatggacgagctgtacaagtaaactagtagcagacagatgcgccgt

ggataaattgttataaaaaaggcagtgatttggatctgaagttgcctgattatttctggc

agccgaattgggatgatagccacccgatccaatgcagttggcgcgccattcgatatcgga

ccctttccctttagtgagggttaatgct

>ACA43_SynSno_3nt

acctttgccaagccaatggcggctaactatctgaagaaccagccgatgtacgtgttccgt

aagacggagctcaagcactccaagaccgagctcaacttcaaggagtggcaaaaggccttt

accgatgtgatgggcatggacgagctgtacaagtaaactagtagcagacagatgcgccgt

ggataaattgttataacgcccacattggtctcccgatgaagttgcctgattatttctggc

agccgaattcgtcctctagccacccgatccaatgcagttggcgcgccattcgatatcgga

ccctttccctttagtgagggttaatgct

>ACA44_s1_SynSno_3nt

acctttgccaagccaatggcggctaactatctgaagaaccagccgatgtacgtgttccgt

aagacggagctcaagcactccaagaccgagctcaacttcaaggagtggcaaaaggccttt

accgatgtgatgggcatggacgagctgtacaagtaaactagtagcagacagatgcgccgt

ggataaattgttatagtactggggatgttttcccacggaagttgcctgattatttctggc

agccgaattttgcgtcaagccacccgatccaatgcagttggcgcgccattcgatatcgga

ccctttccctttagtgagggttaatgct

>ACA44_s2_SynSno_3nt

acctttgccaagccaatggcggctaactatctgaagaaccagccgatgtacgtgttccgt

aagacggagctcaagcactccaagaccgagctcaacttcaaggagtggcaaaaggccttt

accgatgtgatgggcatggacgagctgtacaagtaaactagtagcagacagatgcgccgt

ggataaattgttatacctagtcgtgtgcacctgaggggaagttgcctgattatttctggc

agccgaattatataaccagccacccgatccaatgcagttggcgcgccattcgatatcgga

ccctttccctttagtgagggttaatgct

>ACA48_SynSno_3nt

acctttgccaagccaatggcggctaactatctgaagaaccagccgatgtacgtgttccgt

aagacggagctcaagcactccaagaccgagctcaacttcaaggagtggcaaaaggccttt

accgatgtgatgggcatggacgagctgtacaagtaaactagtagcagacagatgcgccgt

ggataaattgttataccttacggaatggagacccctagaagttgcctgattatttctggc

agccgaattttaggtgaagccacccgatccaatgcagttggcgcgccattcgatatcgga

ccctttccctttagtgagggttaatgct

>U19_up_SynSno_3nt

acctttgccaagccaatggcggctaactatctgaagaaccagccgatgtacgtgttccgt

aagacggagctcaagcactccaagaccgagctcaacttcaaggagtggcaaaaggccttt

accgatgtgatgggcatggacgagctgtacaagtaaactagtagcagacagatgcgccgt

ggataaattgttatacactgtgaactgtgcaccaatagaagttgcctgattatttctggc

agccgaattttaccgttagccacccgatccaatgcagttggcgcgccattcgatatcgga

ccctttccctttagtgagggttaatgct

>U19_dn_SynSno_3nt

acctttgccaagccaatggcggctaactatctgaagaaccagccgatgtacgtgttccgt

aagacggagctcaagcactccaagaccgagctcaacttcaaggagtggcaaaaggccttt

accgatgtgatgggcatggacgagctgtacaagtaaactagtagcagacagatgcgccgt

ggataaattgttatacaaactcctatgctagactcttgaagttgcctgattatttctggc

agccgaatttgggtgcgagccacccgatccaatgcagttggcgcgccattcgatatcgga

ccctttccctttagtgagggttaatgct

>U23_up_SynSno_3nt

acctttgccaagccaatggcggctaactatctgaagaaccagccgatgtacgtgttccgt

aagacggagctcaagcactccaagaccgagctcaacttcaaggagtggcaaaaggccttt

accgatgtgatgggcatggacgagctgtacaagtaaactagtagcagacagatgcgccgt

ggataaattgttataagatgtagaatggcgaccgtctgaagttgcctgattatttctggc

agccgaattccaaaaaaagccacccgatccaatgcagttggcgcgccattcgatatcgga

ccctttccctttagtgagggttaatgct

>U64_up_SynSno_3nt

acctttgccaagccaatggcggctaactatctgaagaaccagccgatgtacgtgttccgt

aagacggagctcaagcactccaagaccgagctcaacttcaaggagtggcaaaaggccttt

accgatgtgatgggcatggacgagctgtacaagtaaactagtagcagacagatgcgccgt

ggataaattgttataaaaatggacgtggcctctaatcgaagttgcctgattatttctggc

agccgaattggttagtcagccacccgatccaatgcagttggcgcgccattcgatatcgga

ccctttccctttagtgagggttaatgct

>U65_up_SynSno_3nt

acctttgccaagccaatggcggctaactatctgaagaaccagccgatgtacgtgttccgt

aagacggagctcaagcactccaagaccgagctcaacttcaaggagtggcaaaaggccttt

accgatgtgatgggcatggacgagctgtacaagtaaactagtagcagacagatgcgccgt

ggataaattgttataacaacagggatggtttatttgtgaagttgcctgattatttctggc

agccgaattggtctccgagccacccgatccaatgcagttggcgcgccattcgatatcgga

ccctttccctttagtgagggttaatgct

>U65_dn_SynSno_3nt

acctttgccaagccaatggcggctaactatctgaagaaccagccgatgtacgtgttccgt

aagacggagctcaagcactccaagaccgagctcaacttcaaggagtggcaaaaggccttt

accgatgtgatgggcatggacgagctgtacaagtaaactagtagcagacagatgcgccgt

ggataaattgttatagtagggaggctgttaagcctaggaagttgcctgattatttctggc

agccgaatttcattccaagccacccgatccaatgcagttggcgcgccattcgatatcgga

ccctttccctttagtgagggttaatgct

>U66_up_SynSno_3nt

acctttgccaagccaatggcggctaactatctgaagaaccagccgatgtacgtgttccgt

aagacggagctcaagcactccaagaccgagctcaacttcaaggagtggcaaaaggccttt

accgatgtgatgggcatggacgagctgtacaagtaaactagtagcagacagatgcgccgt

ggataaattgttatacaagttgtcctgtgcgatctgggaagttgcctgattatttctggc

agccgaattgaagcattagccacccgatccaatgcagttggcgcgccattcgatatcgga

ccctttccctttagtgagggttaatgct

>U68_up_SynSno_3nt

acctttgccaagccaatggcggctaactatctgaagaaccagccgatgtacgtgttccgt

aagacggagctcaagcactccaagaccgagctcaacttcaaggagtggcaaaaggccttt

accgatgtgatgggcatggacgagctgtacaagtaaactagtagcagacagatgcgccgt

ggataaattgttatagaacccgtcatggctcacctcggaagttgcctgattatttctggc

agccgaattaaacgccgagccacccgatccaatgcagttggcgcgccattcgatatcgga

ccctttccctttagtgagggttaatgct

>U69_up_SynSno_3nt

acctttgccaagccaatggcggctaactatctgaagaaccagccgatgtacgtgttccgt

aagacggagctcaagcactccaagaccgagctcaacttcaaggagtggcaaaaggccttt

accgatgtgatgggcatggacgagctgtacaagtaaactagtagcagacagatgcgccgt

ggataaattgttataacttcgcatgtgaaggtaccaagaagttgcctgattatttctggc

agccgaattttattgacagccacccgatccaatgcagttggcgcgccattcgatatcgga

ccctttccctttagtgagggttaatgct

>U69_dn_SynSno_3nt

acctttgccaagccaatggcggctaactatctgaagaaccagccgatgtacgtgttccgt

aagacggagctcaagcactccaagaccgagctcaacttcaaggagtggcaaaaggccttt

accgatgtgatgggcatggacgagctgtacaagtaaactagtagcagacagatgcgccgt

ggataaattgttatagaccgaggtctgaacccgtcgggaagttgcctgattatttctggc

agccgaattaacagcgcagccacccgatccaatgcagttggcgcgccattcgatatcgga

ccctttccctttagtgagggttaatgct

>U70_up_SynSno_3nt

acctttgccaagccaatggcggctaactatctgaagaaccagccgatgtacgtgttccgt

aagacggagctcaagcactccaagaccgagctcaacttcaaggagtggcaaaaggccttt

accgatgtgatgggcatggacgagctgtacaagtaaactagtagcagacagatgcgccgt

ggataaattgttataccttgaactttgtaacagcaacgaagttgcctgattatttctggc

agccgaattgtctggtaagccacccgatccaatgcagttggcgcgccattcgatatcgga

ccctttccctttagtgagggttaatgct

>U71a_up_SynSno_3nt

acctttgccaagccaatggcggctaactatctgaagaaccagccgatgtacgtgttccgt

aagacggagctcaagcactccaagaccgagctcaacttcaaggagtggcaaaaggccttt

accgatgtgatgggcatggacgagctgtacaagtaaactagtagcagacagatgcgccgt

ggataaattgttatacctaggaaggtgacttttcaccgaagttgcctgattatttctggc

agccgaatttatggattagccacccgatccaatgcagttggcgcgccattcgatatcgga

ccctttccctttagtgagggttaatgct

>U72_up_SynSno_3nt

acctttgccaagccaatggcggctaactatctgaagaaccagccgatgtacgtgttccgt

aagacggagctcaagcactccaagaccgagctcaacttcaaggagtggcaaaaggccttt

accgatgtgatgggcatggacgagctgtacaagtaaactagtagcagacagatgcgccgt

ggataaattgttataccgtgggagctgcgctccgcgggaagttgcctgattatttctggc

agccgaattccaccacaagccacccgatccaatgcagttggcgcgccattcgatatcgga

ccctttccctttagtgagggttaatgct

>U99_up_SynSno_3nt

acctttgccaagccaatggcggctaactatctgaagaaccagccgatgtacgtgttccgt

aagacggagctcaagcactccaagaccgagctcaacttcaaggagtggcaaaaggccttt

accgatgtgatgggcatggacgagctgtacaagtaaactagtagcagacagatgcgccgt

ggataaattgttatagaatgcaatttgcccctcccctgaagttgcctgattatttctggc

agccgaattcgtagagaagccacccgatccaatgcagttggcgcgccattcgatatcgga

ccctttccctttagtgagggttaatgct

>ACA5_s1_SynSno_3nt

acctttgccaagccaatggcggctaactatctgaagaaccagccgatgtacgtgttccgt

aagacggagctcaagcactccaagaccgagctcaacttcaaggagtggcaaaaggccttt

accgatgtgatgggcatggacgagctgtacaagtaaactagtagcagacagatgcgccgt

ggataaattgttataaaggagtggctgaagggtcacagaagttgcctgattatttctggc

agccgaatttagggggaagccacccgatccaatgcagttggcgcgccattcgatatcgga

ccctttccctttagtgagggttaatgct

>ACA5_s2_SynSno_3nt

acctttgccaagccaatggcggctaactatctgaagaaccagccgatgtacgtgttccgt

aagacggagctcaagcactccaagaccgagctcaacttcaaggagtggcaaaaggccttt

accgatgtgatgggcatggacgagctgtacaagtaaactagtagcagacagatgcgccgt

ggataaattgttataaacccggtattgccacgtctgagaagttgcctgattatttctggc

agccgaattaactcggtagccacccgatccaatgcagttggcgcgccattcgatatcgga

ccctttccctttagtgagggttaatgct

>ACA50_s1_SynSno_3nt

acctttgccaagccaatggcggctaactatctgaagaaccagccgatgtacgtgttccgt

aagacggagctcaagcactccaagaccgagctcaacttcaaggagtggcaaaaggccttt

accgatgtgatgggcatggacgagctgtacaagtaaactagtagcagacagatgcgccgt

ggataaattgttataagtggtattgtgtccccttactgaagttgcctgattatttctggc

agccgaatttgatggggagccacccgatccaatgcagttggcgcgccattcgatatcgga

ccctttccctttagtgagggttaatgct

>ACA50_s2_SynSno_3nt

acctttgccaagccaatggcggctaactatctgaagaaccagccgatgtacgtgttccgt

aagacggagctcaagcactccaagaccgagctcaacttcaaggagtggcaaaaggccttt

accgatgtgatgggcatggacgagctgtacaagtaaactagtagcagacagatgcgccgt

ggataaattgttatagtgggggaaatgagggcgccacgaagttgcctgattatttctggc

agccgaatttataaggcagccacccgatccaatgcagttggcgcgccattcgatatcgga

ccctttccctttagtgagggttaatgct

>ACA52_SynSno_3nt

acctttgccaagccaatggcggctaactatctgaagaaccagccgatgtacgtgttccgt

aagacggagctcaagcactccaagaccgagctcaacttcaaggagtggcaaaaggccttt

accgatgtgatgggcatggacgagctgtacaagtaaactagtagcagacagatgcgccgt

ggataaattgttataacacctagaatgatgcttcgttgaagttgcctgattatttctggc

agccgaattgcatgaaaagccacccgatccaatgcagttggcgcgccattcgatatcgga

ccctttccctttagtgagggttaatgct

>ACA54_s1_SynSno_3nt

acctttgccaagccaatggcggctaactatctgaagaaccagccgatgtacgtgttccgt

aagacggagctcaagcactccaagaccgagctcaacttcaaggagtggcaaaaggccttt

accgatgtgatgggcatggacgagctgtacaagtaaactagtagcagacagatgcgccgt

ggataaattgttataaattccgggatgtaatccactggaagttgcctgattatttctggc

agccgaattgtccgcccagccacccgatccaatgcagttggcgcgccattcgatatcgga

ccctttccctttagtgagggttaatgct

>ACA54_s2_SynSno_3nt

acctttgccaagccaatggcggctaactatctgaagaaccagccgatgtacgtgttccgt

aagacggagctcaagcactccaagaccgagctcaacttcaaggagtggcaaaaggccttt

accgatgtgatgggcatggacgagctgtacaagtaaactagtagcagacagatgcgccgt

ggataaattgttataagtcccatgatgattgtgataggaagttgcctgattatttctggc

agccgaatttctatacgagccacccgatccaatgcagttggcgcgccattcgatatcgga

ccctttccctttagtgagggttaatgct

>ACA55_SynSno_3nt

acctttgccaagccaatggcggctaactatctgaagaaccagccgatgtacgtgttccgt

aagacggagctcaagcactccaagaccgagctcaacttcaaggagtggcaaaaggccttt

accgatgtgatgggcatggacgagctgtacaagtaaactagtagcagacagatgcgccgt

ggataaattgttatacaacgaggtctgaactcggactgaagttgcctgattatttctggc

agccgaatttttgtggcagccacccgatccaatgcagttggcgcgccattcgatatcgga

ccctttccctttagtgagggttaatgct

>ACA56_SynSno_3nt

acctttgccaagccaatggcggctaactatctgaagaaccagccgatgtacgtgttccgt

aagacggagctcaagcactccaagaccgagctcaacttcaaggagtggcaaaaggccttt

accgatgtgatgggcatggacgagctgtacaagtaaactagtagcagacagatgcgccgt

ggataaattgttatacagtcagccctgggtcgccagtgaagttgcctgattatttctggc

agccgaatttggtaggtagccacccgatccaatgcagttggcgcgccattcgatatcgga

ccctttccctttagtgagggttaatgct

>ACA58_SynSno_3nt

acctttgccaagccaatggcggctaactatctgaagaaccagccgatgtacgtgttccgt

aagacggagctcaagcactccaagaccgagctcaacttcaaggagtggcaaaaggccttt

accgatgtgatgggcatggacgagctgtacaagtaaactagtagcagacagatgcgccgt

ggataaattgttatacacagtgccctgtcgcatctgcgaagttgcctgattatttctggc

agccgaattaattgtaaagccacccgatccaatgcagttggcgcgccattcgatatcgga

ccctttccctttagtgagggttaatgct

>ACA6_SynSno_3nt

acctttgccaagccaatggcggctaactatctgaagaaccagccgatgtacgtgttccgt

aagacggagctcaagcactccaagaccgagctcaacttcaaggagtggcaaaaggccttt

accgatgtgatgggcatggacgagctgtacaagtaaactagtagcagacagatgcgccgt

ggataaattgttataacaggaagactgttgccgctgtgaagttgcctgattatttctggc

agccgaattcggatcgaagccacccgatccaatgcagttggcgcgccattcgatatcgga

ccctttccctttagtgagggttaatgct

>ACA60_SynSno_3nt

acctttgccaagccaatggcggctaactatctgaagaaccagccgatgtacgtgttccgt

aagacggagctcaagcactccaagaccgagctcaacttcaaggagtggcaaaaggccttt

accgatgtgatgggcatggacgagctgtacaagtaaactagtagcagacagatgcgccgt

ggataaattgttataataactaatttgcccccacattgaagttgcctgattatttctggc

agccgaattcgcaggttagccacccgatccaatgcagttggcgcgccattcgatatcgga

ccctttccctttagtgagggttaatgct

>ACA61_SynSno_3nt

acctttgccaagccaatggcggctaactatctgaagaaccagccgatgtacgtgttccgt

aagacggagctcaagcactccaagaccgagctcaacttcaaggagtggcaaaaggccttt

accgatgtgatgggcatggacgagctgtacaagtaaactagtagcagacagatgcgccgt

ggataaattgttataaaggggccgatggacctttcgagaagttgcctgattatttctggc

agccgaattagtcgggcagccacccgatccaatgcagttggcgcgccattcgatatcgga

ccctttccctttagtgagggttaatgct

>ACA62_s1_SynSno_3nt

acctttgccaagccaatggcggctaactatctgaagaaccagccgatgtacgtgttccgt

aagacggagctcaagcactccaagaccgagctcaacttcaaggagtggcaaaaggccttt

accgatgtgatgggcatggacgagctgtacaagtaaactagtagcagacagatgcgccgt

ggataaattgttataagtactcttgtgtcccctcactgaagttgcctgattatttctggc

agccgaatttgggctggagccacccgatccaatgcagttggcgcgccattcgatatcgga

ccctttccctttagtgagggttaatgct

>ACA62_s2_SynSno_3nt

acctttgccaagccaatggcggctaactatctgaagaaccagccgatgtacgtgttccgt

aagacggagctcaagcactccaagaccgagctcaacttcaaggagtggcaaaaggccttt

accgatgtgatgggcatggacgagctgtacaagtaaactagtagcagacagatgcgccgt

ggataaattgttataatattggaaatgagggcgtatagaagttgcctgattatttctggc

agccgaattcggcgtaaagccacccgatccaatgcagttggcgcgccattcgatatcgga

ccctttccctttagtgagggttaatgct

>ACA63_SynSno_3nt

acctttgccaagccaatggcggctaactatctgaagaaccagccgatgtacgtgttccgt

aagacggagctcaagcactccaagaccgagctcaacttcaaggagtggcaaaaggccttt

accgatgtgatgggcatggacgagctgtacaagtaaactagtagcagacagatgcgccgt

ggataaattgttatatcactccaaatgatctgtctgagaagttgcctgattatttctggc

agccgaattaactacacagccacccgatccaatgcagttggcgcgccattcgatatcgga

ccctttccctttagtgagggttaatgct

>ACA64_SynSno_3nt

acctttgccaagccaatggcggctaactatctgaagaaccagccgatgtacgtgttccgt

aagacggagctcaagcactccaagaccgagctcaacttcaaggagtggcaaaaggccttt

accgatgtgatgggcatggacgagctgtacaagtaaactagtagcagacagatgcgccgt

ggataaattgttatataagtttttttgagagtcttaagaagttgcctgattatttctggc

agccgaattgttctgtcagccacccgatccaatgcagttggcgcgccattcgatatcgga

ccctttccctttagtgagggttaatgct

>ACA65_s1_SynSno_3nt

acctttgccaagccaatggcggctaactatctgaagaaccagccgatgtacgtgttccgt

aagacggagctcaagcactccaagaccgagctcaacttcaaggagtggcaaaaggccttt

accgatgtgatgggcatggacgagctgtacaagtaaactagtagcagacagatgcgccgt

ggataaattgttatagtcaagcaaatgagccactgacgaagttgcctgattatttctggc

agccgaattggcggagcagccacccgatccaatgcagttggcgcgccattcgatatcgga

ccctttccctttagtgagggttaatgct

>ACA65_s2_SynSno_3nt

acctttgccaagccaatggcggctaactatctgaagaaccagccgatgtacgtgttccgt

aagacggagctcaagcactccaagaccgagctcaacttcaaggagtggcaaaaggccttt

accgatgtgatgggcatggacgagctgtacaagtaaactagtagcagacagatgcgccgt

ggataaattgttataggaatacaattggttcctccccgaagttgcctgattatttctggc

agccgaattatgagctcagccacccgatccaatgcagttggcgcgccattcgatatcgga

ccctttccctttagtgagggttaatgct

>ACA67_s1_SynSno_3nt

acctttgccaagccaatggcggctaactatctgaagaaccagccgatgtacgtgttccgt

aagacggagctcaagcactccaagaccgagctcaacttcaaggagtggcaaaaggccttt

accgatgtgatgggcatggacgagctgtacaagtaaactagtagcagacagatgcgccgt

ggataaattgttatacgttttgacttgaacgaacccagaagttgcctgattatttctggc

agccgaattcacgatccagccacccgatccaatgcagttggcgcgccattcgatatcgga

ccctttccctttagtgagggttaatgct

>ACA67_s2_SynSno_3nt

acctttgccaagccaatggcggctaactatctgaagaaccagccgatgtacgtgttccgt

aagacggagctcaagcactccaagaccgagctcaacttcaaggagtggcaaaaggccttt

accgatgtgatgggcatggacgagctgtacaagtaaactagtagcagacagatgcgccgt

ggataaattgttataaaacaggcagtgatttggacttgaagttgcctgattatttctggc

agccgaattttaattgtagccacccgatccaatgcagttggcgcgccattcgatatcgga

ccctttccctttagtgagggttaatgct

>ACA7_s1_SynSno_3nt

acctttgccaagccaatggcggctaactatctgaagaaccagccgatgtacgtgttccgt

aagacggagctcaagcactccaagaccgagctcaacttcaaggagtggcaaaaggccttt

accgatgtgatgggcatggacgagctgtacaagtaaactagtagcagacagatgcgccgt

ggataaattgttatagaaagggccgtggctcgaaactgaagttgcctgattatttctggc

agccgaattgtggacccagccacccgatccaatgcagttggcgcgccattcgatatcgga

ccctttccctttagtgagggttaatgct

>ACA7_s2_SynSno_3nt

acctttgccaagccaatggcggctaactatctgaagaaccagccgatgtacgtgttccgt

aagacggagctcaagcactccaagaccgagctcaacttcaaggagtggcaaaaggccttt

accgatgtgatgggcatggacgagctgtacaagtaaactagtagcagacagatgcgccgt

ggataaattgttatactctgtgctttgaagtttgtaagaagttgcctgattatttctggc

agccgaattaaacgaggagccacccgatccaatgcagttggcgcgccattcgatatcgga

ccctttccctttagtgagggttaatgct

>ACA8_s1_SynSno_3nt

acctttgccaagccaatggcggctaactatctgaagaaccagccgatgtacgtgttccgt

aagacggagctcaagcactccaagaccgagctcaacttcaaggagtggcaaaaggccttt

accgatgtgatgggcatggacgagctgtacaagtaaactagtagcagacagatgcgccgt

ggataaattgttatagtcaaggtgatgagcgcaacgtgaagttgcctgattatttctggc

agccgaattccatgggtagccacccgatccaatgcagttggcgcgccattcgatatcgga

ccctttccctttagtgagggttaatgct

>ACA8_s2_SynSno_3nt

acctttgccaagccaatggcggctaactatctgaagaaccagccgatgtacgtgttccgt

aagacggagctcaagcactccaagaccgagctcaacttcaaggagtggcaaaaggccttt

accgatgtgatgggcatggacgagctgtacaagtaaactagtagcagacagatgcgccgt

ggataaattgttatactcccgcccatgcacatcgtaagaagttgcctgattatttctggc

agccgaattcacaacgcagccacccgatccaatgcagttggcgcgccattcgatatcgga

ccctttccctttagtgagggttaatgct

>ACA9_s1_SynSno_3nt

acctttgccaagccaatggcggctaactatctgaagaaccagccgatgtacgtgttccgt

aagacggagctcaagcactccaagaccgagctcaacttcaaggagtggcaaaaggccttt

accgatgtgatgggcatggacgagctgtacaagtaaactagtagcagacagatgcgccgt

ggataaattgttataacagtccggatggcgttcttaggaagttgcctgattatttctggc

agccgaattaggtgacaagccacccgatccaatgcagttggcgcgccattcgatatcgga

ccctttccctttagtgagggttaatgct

>ACA9_s2_SynSno_3nt

acctttgccaagccaatggcggctaactatctgaagaaccagccgatgtacgtgttccgt

aagacggagctcaagcactccaagaccgagctcaacttcaaggagtggcaaaaggccttt

accgatgtgatgggcatggacgagctgtacaagtaaactagtagcagacagatgcgccgt

ggataaattgttatacccgggatattgtccccaatgggaagttgcctgattatttctggc

agccgaattccatttagagccacccgatccaatgcagttggcgcgccattcgatatcgga

ccctttccctttagtgagggttaatgct

>E2_s1_SynSno_3nt

acctttgccaagccaatggcggctaactatctgaagaaccagccgatgtacgtgttccgt

aagacggagctcaagcactccaagaccgagctcaacttcaaggagtggcaaaaggccttt

accgatgtgatgggcatggacgagctgtacaagtaaactagtagcagacagatgcgccgt

ggataaattgttataacagcaatactgtcgccaccgtgaagttgcctgattatttctggc

agccgaattacttctgcagccacccgatccaatgcagttggcgcgccattcgatatcgga

ccctttccctttagtgagggttaatgct

>E2_s2_SynSno_3nt

acctttgccaagccaatggcggctaactatctgaagaaccagccgatgtacgtgttccgt

aagacggagctcaagcactccaagaccgagctcaacttcaaggagtggcaaaaggccttt

accgatgtgatgggcatggacgagctgtacaagtaaactagtagcagacagatgcgccgt

ggataaattgttatactccggcctatgtatatccccagaagttgcctgattatttctggc

agccgaatttcgggtccagccacccgatccaatgcagttggcgcgccattcgatatcgga

ccctttccctttagtgagggttaatgct

>E3_SynSno_3nt

acctttgccaagccaatggcggctaactatctgaagaaccagccgatgtacgtgttccgt

aagacggagctcaagcactccaagaccgagctcaacttcaaggagtggcaaaaggccttt

accgatgtgatgggcatggacgagctgtacaagtaaactagtagcagacagatgcgccgt

ggataaattgttatatcaacctcgttgatctggcctcgaagttgcctgattatttctggc

agccgaattccgcctctagccacccgatccaatgcagttggcgcgccattcgatatcgga

ccctttccctttagtgagggttaatgct

>HBI-115_SynSno_3nt

acctttgccaagccaatggcggctaactatctgaagaaccagccgatgtacgtgttccgt

aagacggagctcaagcactccaagaccgagctcaacttcaaggagtggcaaaaggccttt

accgatgtgatgggcatggacgagctgtacaagtaaactagtagcagacagatgcgccgt

ggataaattgttatattgaggcacctgttagactgaagaagttgcctgattatttctggc

agccgaattgatgaccaagccacccgatccaatgcagttggcgcgccattcgatatcgga

ccctttccctttagtgagggttaatgct

>HBI-6_SynSno_3nt

acctttgccaagccaatggcggctaactatctgaagaaccagccgatgtacgtgttccgt

aagacggagctcaagcactccaagaccgagctcaacttcaaggagtggcaaaaggccttt

accgatgtgatgggcatggacgagctgtacaagtaaactagtagcagacagatgcgccgt

ggataaattgttataactcactgattggtgggcgttcgaagttgcctgattatttctggc

agccgaatttgtccccaagccacccgatccaatgcagttggcgcgccattcgatatcgga

ccctttccctttagtgagggttaatgct

>HBI-61_SynSno_3nt

acctttgccaagccaatggcggctaactatctgaagaaccagccgatgtacgtgttccgt

aagacggagctcaagcactccaagaccgagctcaacttcaaggagtggcaaaaggccttt

accgatgtgatgggcatggacgagctgtacaagtaaactagtagcagacagatgcgccgt

ggataaattgttataaggtgtggtatgtgaggcgcacgaagttgcctgattatttctggc

agccgaattgtccaagtagccacccgatccaatgcagttggcgcgccattcgatatcgga

ccctttccctttagtgagggttaatgct

>ACA1_SynSno_hyb10nt_mut1C

acctttgccaagccaatggcggctaactatctgaagaaccagccgatgtacgtgttccgt

aagacggagctcaagcactccaagaccgagctcaacttcaaggagtggcaaaaggccttt

accgatgtgatgggcatggacgagctgtacaagtaaactagtagcagacagatgcgccgt

ggataaattgttataggtctcattgtcaagcataaccgaagttgcctgattatttctggc

agccgaatttgcttatcagccacccgatccaatgcagttggcgcgccattcgatatcgga

ccctttccctttagtgagggttaatgct

>ACA10_s1_SynSno_hyb10nt_mut1C

acctttgccaagccaatggcggctaactatctgaagaaccagccgatgtacgtgttccgt

aagacggagctcaagcactccaagaccgagctcaacttcaaggagtggcaaaaggccttt

accgatgtgatgggcatggacgagctgtacaagtaaactagtagcagacagatgcgccgt

ggataaattgttatacctgaaaacatcagctgagagagaagttgcctgattatttctggc

agccgaattggacggttagccacccgatccaatgcagttggcgcgccattcgatatcgga

ccctttccctttagtgagggttaatgct

>ACA10_s2_SynSno_hyb10nt_mut1C

acctttgccaagccaatggcggctaactatctgaagaaccagccgatgtacgtgttccgt

aagacggagctcaagcactccaagaccgagctcaacttcaaggagtggcaaaaggccttt

accgatgtgatgggcatggacgagctgtacaagtaaactagtagcagacagatgcgccgt

ggataaattgttataactcctaggatccgtgcattaggaagttgcctgattatttctggc

agccgaattgaagtgatagccacccgatccaatgcagttggcgcgccattcgatatcgga

ccctttccctttagtgagggttaatgct

>ACA13_SynSno_hyb10nt_mut1C

acctttgccaagccaatggcggctaactatctgaagaaccagccgatgtacgtgttccgt

aagacggagctcaagcactccaagaccgagctcaacttcaaggagtggcaaaaggccttt

accgatgtgatgggcatggacgagctgtacaagtaaactagtagcagacagatgcgccgt

ggataaattgttatacttttttgactcaacacaaagggaagttgcctgattatttctggc

agccgaattgtaatccgagccacccgatccaatgcagttggcgcgccattcgatatcgga

ccctttccctttagtgagggttaatgct

>ACA14a_SynSno_hyb10nt_mut1C

acctttgccaagccaatggcggctaactatctgaagaaccagccgatgtacgtgttccgt

aagacggagctcaagcactccaagaccgagctcaacttcaaggagtggcaaaaggccttt

accgatgtgatgggcatggacgagctgtacaagtaaactagtagcagacagatgcgccgt

ggataaattgttataggtatgaaattcttggactttcgaagttgcctgattatttctggc

agccgaattttaatgcaagccacccgatccaatgcagttggcgcgccattcgatatcgga

ccctttccctttagtgagggttaatgct

>ACA15_SynSno_hyb10nt_mut1C

acctttgccaagccaatggcggctaactatctgaagaaccagccgatgtacgtgttccgt

aagacggagctcaagcactccaagaccgagctcaacttcaaggagtggcaaaaggccttt

accgatgtgatgggcatggacgagctgtacaagtaaactagtagcagacagatgcgccgt

ggataaattgttatatggtgtctggtctattcggccagaagttgcctgattatttctggc

agccgaattcagtctgtagccacccgatccaatgcagttggcgcgccattcgatatcgga

ccctttccctttagtgagggttaatgct

>ACA16_SynSno_hyb10nt_mut1C

acctttgccaagccaatggcggctaactatctgaagaaccagccgatgtacgtgttccgt

aagacggagctcaagcactccaagaccgagctcaacttcaaggagtggcaaaaggccttt

accgatgtgatgggcatggacgagctgtacaagtaaactagtagcagacagatgcgccgt

ggataaattgttatatcctttttgatccttcgataaggaagttgcctgattatttctggc

agccgaattaagtactaagccacccgatccaatgcagttggcgcgccattcgatatcgga

ccctttccctttagtgagggttaatgct

>ACA17_s1_SynSno_hyb10nt_mut1C

acctttgccaagccaatggcggctaactatctgaagaaccagccgatgtacgtgttccgt

aagacggagctcaagcactccaagaccgagctcaacttcaaggagtggcaaaaggccttt

accgatgtgatgggcatggacgagctgtacaagtaaactagtagcagacagatgcgccgt

ggataaattgttataacctaatgactcaacgcctctagaagttgcctgattatttctggc

agccgaattctctgccgagccacccgatccaatgcagttggcgcgccattcgatatcgga

ccctttccctttagtgagggttaatgct

>ACA17_s2_SynSno_hyb10nt_mut1C

acctttgccaagccaatggcggctaactatctgaagaaccagccgatgtacgtgttccgt

aagacggagctcaagcactccaagaccgagctcaacttcaaggagtggcaaaaggccttt

accgatgtgatgggcatggacgagctgtacaagtaaactagtagcagacagatgcgccgt

ggataaattgttatatatcaaacattcagcgtagacagaagttgcctgattatttctggc

agccgaattagcgtacaagccacccgatccaatgcagttggcgcgccattcgatatcgga

ccctttccctttagtgagggttaatgct

>ACA19_s1_SynSno_hyb10nt_mut1C

acctttgccaagccaatggcggctaactatctgaagaaccagccgatgtacgtgttccgt

aagacggagctcaagcactccaagaccgagctcaacttcaaggagtggcaaaaggccttt

accgatgtgatgggcatggacgagctgtacaagtaaactagtagcagacagatgcgccgt

ggataaattgttatacataagaaattcaatgaaatgtgaagttgcctgattatttctggc

agccgaattgtacgatcagccacccgatccaatgcagttggcgcgccattcgatatcgga

ccctttccctttagtgagggttaatgct

>ACA19_s2_SynSno_hyb10nt_mut1C

acctttgccaagccaatggcggctaactatctgaagaaccagccgatgtacgtgttccgt

aagacggagctcaagcactccaagaccgagctcaacttcaaggagtggcaaaaggccttt

accgatgtgatgggcatggacgagctgtacaagtaaactagtagcagacagatgcgccgt

ggataaattgttataaaggagactgtctaattagcctgaagttgcctgattatttctggc

agccgaatttgcaaagcagccacccgatccaatgcagttggcgcgccattcgatatcgga

ccctttccctttagtgagggttaatgct

>ACA19_s3_SynSno_hyb10nt_mut1C

acctttgccaagccaatggcggctaactatctgaagaaccagccgatgtacgtgttccgt

aagacggagctcaagcactccaagaccgagctcaacttcaaggagtggcaaaaggccttt

accgatgtgatgggcatggacgagctgtacaagtaaactagtagcagacagatgcgccgt

ggataaattgttatacacataagaatcatgaaatgtggaagttgcctgattatttctggc

agccgaattggacttctagccacccgatccaatgcagttggcgcgccattcgatatcgga

ccctttccctttagtgagggttaatgct

>ACA20_SynSno_hyb10nt_mut1C

acctttgccaagccaatggcggctaactatctgaagaaccagccgatgtacgtgttccgt

aagacggagctcaagcactccaagaccgagctcaacttcaaggagtggcaaaaggccttt

accgatgtgatgggcatggacgagctgtacaagtaaactagtagcagacagatgcgccgt

ggataaattgttatacatgcgtatatcaaataaatgggaagttgcctgattatttctggc

agccgaattttatatgaagccacccgatccaatgcagttggcgcgccattcgatatcgga

ccctttccctttagtgagggttaatgct

>ACA21_s1_SynSno_hyb10nt_mut1C

acctttgccaagccaatggcggctaactatctgaagaaccagccgatgtacgtgttccgt

aagacggagctcaagcactccaagaccgagctcaacttcaaggagtggcaaaaggccttt

accgatgtgatgggcatggacgagctgtacaagtaaactagtagcagacagatgcgccgt

ggataaattgttatatttcttgacgtcgcttttaaaagaagttgcctgattatttctggc

agccgaattcccccagcagccacccgatccaatgcagttggcgcgccattcgatatcgga

ccctttccctttagtgagggttaatgct

>ACA21_s2_SynSno_hyb10nt_mut1C

acctttgccaagccaatggcggctaactatctgaagaaccagccgatgtacgtgttccgt

aagacggagctcaagcactccaagaccgagctcaacttcaaggagtggcaaaaggccttt

accgatgtgatgggcatggacgagctgtacaagtaaactagtagcagacagatgcgccgt

ggataaattgttataccaccgattgtccacccaaaaggaagttgcctgattatttctggc

agccgaattctacgtaaagccacccgatccaatgcagttggcgcgccattcgatatcgga

ccctttccctttagtgagggttaatgct

>ACA22_s1_SynSno_hyb10nt_mut1C

acctttgccaagccaatggcggctaactatctgaagaaccagccgatgtacgtgttccgt

aagacggagctcaagcactccaagaccgagctcaacttcaaggagtggcaaaaggccttt

accgatgtgatgggcatggacgagctgtacaagtaaactagtagcagacagatgcgccgt

ggataaattgttataagggtcaaagtctcactgtgcagaagttgcctgattatttctggc

agccgaattataactctagccacccgatccaatgcagttggcgcgccattcgatatcgga

ccctttccctttagtgagggttaatgct

>ACA22_s2_SynSno_hyb10nt_mut1C

acctttgccaagccaatggcggctaactatctgaagaaccagccgatgtacgtgttccgt

aagacggagctcaagcactccaagaccgagctcaacttcaaggagtggcaaaaggccttt

accgatgtgatgggcatggacgagctgtacaagtaaactagtagcagacagatgcgccgt

ggataaattgttatatctgaatacatcgcagaggagagaagttgcctgattatttctggc

agccgaattgtctggaaagccacccgatccaatgcagttggcgcgccattcgatatcgga

ccctttccctttagtgagggttaatgct

>ACA23_s1_SynSno_hyb10nt_mut1C

acctttgccaagccaatggcggctaactatctgaagaaccagccgatgtacgtgttccgt

aagacggagctcaagcactccaagaccgagctcaacttcaaggagtggcaaaaggccttt

accgatgtgatgggcatggacgagctgtacaagtaaactagtagcagacagatgcgccgt

ggataaattgttatacatgatgttttcagcagccatggaagttgcctgattatttctggc

agccgaattatagtcagagccacccgatccaatgcagttggcgcgccattcgatatcgga

ccctttccctttagtgagggttaatgct

>ACA23_s2_SynSno_hyb10nt_mut1C

acctttgccaagccaatggcggctaactatctgaagaaccagccgatgtacgtgttccgt

aagacggagctcaagcactccaagaccgagctcaacttcaaggagtggcaaaaggccttt

accgatgtgatgggcatggacgagctgtacaagtaaactagtagcagacagatgcgccgt

ggataaattgttatagaatttggagtcactatgatccgaagttgcctgattatttctggc

agccgaattcattttggagccacccgatccaatgcagttggcgcgccattcgatatcgga

ccctttccctttagtgagggttaatgct

>ACA24_s1_SynSno_hyb10nt_mut1C

acctttgccaagccaatggcggctaactatctgaagaaccagccgatgtacgtgttccgt

aagacggagctcaagcactccaagaccgagctcaacttcaaggagtggcaaaaggccttt

accgatgtgatgggcatggacgagctgtacaagtaaactagtagcagacagatgcgccgt

ggataaattgttatatggctaggaatcaaagatacatgaagttgcctgattatttctggc

agccgaattatgcgagaagccacccgatccaatgcagttggcgcgccattcgatatcgga

ccctttccctttagtgagggttaatgct

>ACA24_s2_SynSno_hyb10nt_mut1C

acctttgccaagccaatggcggctaactatctgaagaaccagccgatgtacgtgttccgt

aagacggagctcaagcactccaagaccgagctcaacttcaaggagtggcaaaaggccttt

accgatgtgatgggcatggacgagctgtacaagtaaactagtagcagacagatgcgccgt

ggataaattgttatatgtgcaagtctcttgccaataagaagttgcctgattatttctggc

agccgaatttgctaagcagccacccgatccaatgcagttggcgcgccattcgatatcgga

ccctttccctttagtgagggttaatgct

>ACA25_s1_SynSno_hyb10nt_mut1C

acctttgccaagccaatggcggctaactatctgaagaaccagccgatgtacgtgttccgt

aagacggagctcaagcactccaagaccgagctcaacttcaaggagtggcaaaaggccttt

accgatgtgatgggcatggacgagctgtacaagtaaactagtagcagacagatgcgccgt

ggataaattgttatatcacagcgtttcctttgaaatggaagttgcctgattatttctggc

agccgaattagtgctacagccacccgatccaatgcagttggcgcgccattcgatatcgga

ccctttccctttagtgagggttaatgct

>ACA25_s2_SynSno_hyb10nt_mut1C

acctttgccaagccaatggcggctaactatctgaagaaccagccgatgtacgtgttccgt

aagacggagctcaagcactccaagaccgagctcaacttcaaggagtggcaaaaggccttt

accgatgtgatgggcatggacgagctgtacaagtaaactagtagcagacagatgcgccgt

ggataaattgttatactcaggaaaatcggagtgctatgaagttgcctgattatttctggc

agccgaattcatagtggagccacccgatccaatgcagttggcgcgccattcgatatcgga

ccctttccctttagtgagggttaatgct

>ACA27_s1_SynSno_hyb10nt_mut1C

acctttgccaagccaatggcggctaactatctgaagaaccagccgatgtacgtgttccgt

aagacggagctcaagcactccaagaccgagctcaacttcaaggagtggcaaaaggccttt

accgatgtgatgggcatggacgagctgtacaagtaaactagtagcagacagatgcgccgt

ggataaattgttatacacttgaatgtcaaagtgaaaagaagttgcctgattatttctggc

agccgaattcaaacgcgagccacccgatccaatgcagttggcgcgccattcgatatcgga

ccctttccctttagtgagggttaatgct

>ACA27_s2_SynSno_hyb10nt_mut1C

acctttgccaagccaatggcggctaactatctgaagaaccagccgatgtacgtgttccgt

aagacggagctcaagcactccaagaccgagctcaacttcaaggagtggcaaaaggccttt

accgatgtgatgggcatggacgagctgtacaagtaaactagtagcagacagatgcgccgt

ggataaattgttatatctgacaggatcgttttaaaaggaagttgcctgattatttctggc

agccgaatttcctctctagccacccgatccaatgcagttggcgcgccattcgatatcgga

ccctttccctttagtgagggttaatgct

>ACA28_s1_SynSno_hyb10nt_mut1C

acctttgccaagccaatggcggctaactatctgaagaaccagccgatgtacgtgttccgt

aagacggagctcaagcactccaagaccgagctcaacttcaaggagtggcaaaaggccttt

accgatgtgatgggcatggacgagctgtacaagtaaactagtagcagacagatgcgccgt

ggataaattgttataaagctcaaattcgagtgttgctgaagttgcctgattatttctggc

agccgaattttctctgaagccacccgatccaatgcagttggcgcgccattcgatatcgga

ccctttccctttagtgagggttaatgct

>ACA28_s2_SynSno_hyb10nt_mut1C

acctttgccaagccaatggcggctaactatctgaagaaccagccgatgtacgtgttccgt

aagacggagctcaagcactccaagaccgagctcaacttcaaggagtggcaaaaggccttt

accgatgtgatgggcatggacgagctgtacaagtaaactagtagcagacagatgcgccgt

ggataaattgttatagtctatataatcgaataggttagaagttgcctgattatttctggc

agccgaatttgtaatagagccacccgatccaatgcagttggcgcgccattcgatatcgga

ccctttccctttagtgagggttaatgct

>ACA2a_s1_SynSno_hyb10nt_mut1C

acctttgccaagccaatggcggctaactatctgaagaaccagccgatgtacgtgttccgt

aagacggagctcaagcactccaagaccgagctcaacttcaaggagtggcaaaaggccttt

accgatgtgatgggcatggacgagctgtacaagtaaactagtagcagacagatgcgccgt

ggataaattgttatatcctgtttgatcttgattcagggaagttgcctgattatttctggc

agccgaatttttctaggagccacccgatccaatgcagttggcgcgccattcgatatcgga

ccctttccctttagtgagggttaatgct

>ACA2a_s2_SynSno_hyb10nt_mut1C

acctttgccaagccaatggcggctaactatctgaagaaccagccgatgtacgtgttccgt

aagacggagctcaagcactccaagaccgagctcaacttcaaggagtggcaaaaggccttt

accgatgtgatgggcatggacgagctgtacaagtaaactagtagcagacagatgcgccgt

ggataaattgttatatccaactgaatccagaccacaagaagttgcctgattatttctggc

agccgaattgatttgacagccacccgatccaatgcagttggcgcgccattcgatatcgga

ccctttccctttagtgagggttaatgct

>ACA3_s1_SynSno_hyb10nt_mut1C

acctttgccaagccaatggcggctaactatctgaagaaccagccgatgtacgtgttccgt

aagacggagctcaagcactccaagaccgagctcaacttcaaggagtggcaaaaggccttt

accgatgtgatgggcatggacgagctgtacaagtaaactagtagcagacagatgcgccgt

ggataaattgttataggactctagctcgactctagccgaagttgcctgattatttctggc

agccgaattataaaataagccacccgatccaatgcagttggcgcgccattcgatatcgga

ccctttccctttagtgagggttaatgct

>ACA1_SynSno_hyb10nt_mut1A

acctttgccaagccaatggcggctaactatctgaagaaccagccgatgtacgtgttccgt

aagacggagctcaagcactccaagaccgagctcaacttcaaggagtggcaaaaggccttt

accgatgtgatgggcatggacgagctgtacaagtaaactagtagcagacagatgcgccgt

ggataaattgttataggtctcattgtaaagcataaccgaagttgcctgattatttctggc

agccgaattctcaatacagccacccgatccaatgcagttggcgcgccattcgatatcgga

ccctttccctttagtgagggttaatgct

>ACA10_s1_SynSno_hyb10nt_mut1A

acctttgccaagccaatggcggctaactatctgaagaaccagccgatgtacgtgttccgt

aagacggagctcaagcactccaagaccgagctcaacttcaaggagtggcaaaaggccttt

accgatgtgatgggcatggacgagctgtacaagtaaactagtagcagacagatgcgccgt

ggataaattgttatacctgaaaacataagctgagagagaagttgcctgattatttctggc

agccgaattccgccttcagccacccgatccaatgcagttggcgcgccattcgatatcgga

ccctttccctttagtgagggttaatgct

>ACA10_s2_SynSno_hyb10nt_mut1A

acctttgccaagccaatggcggctaactatctgaagaaccagccgatgtacgtgttccgt

aagacggagctcaagcactccaagaccgagctcaacttcaaggagtggcaaaaggccttt

accgatgtgatgggcatggacgagctgtacaagtaaactagtagcagacagatgcgccgt

ggataaattgttataactcctaggatacgtgcattaggaagttgcctgattatttctggc

agccgaattctgtagctagccacccgatccaatgcagttggcgcgccattcgatatcgga

ccctttccctttagtgagggttaatgct

>ACA13_SynSno_hyb10nt_mut1A

acctttgccaagccaatggcggctaactatctgaagaaccagccgatgtacgtgttccgt

aagacggagctcaagcactccaagaccgagctcaacttcaaggagtggcaaaaggccttt

accgatgtgatgggcatggacgagctgtacaagtaaactagtagcagacagatgcgccgt

ggataaattgttatacttttttgactaaacacaaagggaagttgcctgattatttctggc

agccgaattatctacggagccacccgatccaatgcagttggcgcgccattcgatatcgga

ccctttccctttagtgagggttaatgct

>ACA14a_SynSno_hyb10nt_mut1A

acctttgccaagccaatggcggctaactatctgaagaaccagccgatgtacgtgttccgt

aagacggagctcaagcactccaagaccgagctcaacttcaaggagtggcaaaaggccttt

accgatgtgatgggcatggacgagctgtacaagtaaactagtagcagacagatgcgccgt

ggataaattgttataggtatgaaattattggactttcgaagttgcctgattatttctggc

agccgaatttgatgcttagccacccgatccaatgcagttggcgcgccattcgatatcgga

ccctttccctttagtgagggttaatgct

>ACA15_SynSno_hyb10nt_mut1A

acctttgccaagccaatggcggctaactatctgaagaaccagccgatgtacgtgttccgt

aagacggagctcaagcactccaagaccgagctcaacttcaaggagtggcaaaaggccttt

accgatgtgatgggcatggacgagctgtacaagtaaactagtagcagacagatgcgccgt

ggataaattgttatatggtgtctggtatattcggccagaagttgcctgattatttctggc

agccgaattatcctgcgagccacccgatccaatgcagttggcgcgccattcgatatcgga

ccctttccctttagtgagggttaatgct

>ACA16_SynSno_hyb10nt_mut1A

acctttgccaagccaatggcggctaactatctgaagaaccagccgatgtacgtgttccgt

aagacggagctcaagcactccaagaccgagctcaacttcaaggagtggcaaaaggccttt

accgatgtgatgggcatggacgagctgtacaagtaaactagtagcagacagatgcgccgt

ggataaattgttatatcctttttgatacttcgataaggaagttgcctgattatttctggc

agccgaattgatagggtagccacccgatccaatgcagttggcgcgccattcgatatcgga

ccctttccctttagtgagggttaatgct

>ACA17_s1_SynSno_hyb10nt_mut1A

acctttgccaagccaatggcggctaactatctgaagaaccagccgatgtacgtgttccgt

aagacggagctcaagcactccaagaccgagctcaacttcaaggagtggcaaaaggccttt

accgatgtgatgggcatggacgagctgtacaagtaaactagtagcagacagatgcgccgt

ggataaattgttataacctaatgactaaacgcctctagaagttgcctgattatttctggc

agccgaattaaacgattagccacccgatccaatgcagttggcgcgccattcgatatcgga

ccctttccctttagtgagggttaatgct

>ACA17_s2_SynSno_hyb10nt_mut1A

acctttgccaagccaatggcggctaactatctgaagaaccagccgatgtacgtgttccgt

aagacggagctcaagcactccaagaccgagctcaacttcaaggagtggcaaaaggccttt

accgatgtgatgggcatggacgagctgtacaagtaaactagtagcagacagatgcgccgt

ggataaattgttatatatcaaacattaagcgtagacagaagttgcctgattatttctggc

agccgaatttgacggtgagccacccgatccaatgcagttggcgcgccattcgatatcgga

ccctttccctttagtgagggttaatgct

>ACA19_s1_SynSno_hyb10nt_mut1A

acctttgccaagccaatggcggctaactatctgaagaaccagccgatgtacgtgttccgt

aagacggagctcaagcactccaagaccgagctcaacttcaaggagtggcaaaaggccttt

accgatgtgatgggcatggacgagctgtacaagtaaactagtagcagacagatgcgccgt

ggataaattgttatacataagaaattaaatgaaatgtgaagttgcctgattatttctggc

agccgaattgtccgtagagccacccgatccaatgcagttggcgcgccattcgatatcgga

ccctttccctttagtgagggttaatgct

>ACA19_s2_SynSno_hyb10nt_mut1A

acctttgccaagccaatggcggctaactatctgaagaaccagccgatgtacgtgttccgt

aagacggagctcaagcactccaagaccgagctcaacttcaaggagtggcaaaaggccttt

accgatgtgatgggcatggacgagctgtacaagtaaactagtagcagacagatgcgccgt

ggataaattgttataaaggagactgtataattagcctgaagttgcctgattatttctggc

agccgaattatttccatagccacccgatccaatgcagttggcgcgccattcgatatcgga

ccctttccctttagtgagggttaatgct

>ACA19_s3_SynSno_hyb10nt_mut1A

acctttgccaagccaatggcggctaactatctgaagaaccagccgatgtacgtgttccgt

aagacggagctcaagcactccaagaccgagctcaacttcaaggagtggcaaaaggccttt

accgatgtgatgggcatggacgagctgtacaagtaaactagtagcagacagatgcgccgt

ggataaattgttatacacataagaataatgaaatgtggaagttgcctgattatttctggc

agccgaattggcgtttaagccacccgatccaatgcagttggcgcgccattcgatatcgga

ccctttccctttagtgagggttaatgct

>ACA20_SynSno_hyb10nt_mut1A

acctttgccaagccaatggcggctaactatctgaagaaccagccgatgtacgtgttccgt

aagacggagctcaagcactccaagaccgagctcaacttcaaggagtggcaaaaggccttt

accgatgtgatgggcatggacgagctgtacaagtaaactagtagcagacagatgcgccgt

ggataaattgttatacatgcgtatataaaataaatgggaagttgcctgattatttctggc

agccgaattgcatatagagccacccgatccaatgcagttggcgcgccattcgatatcgga

ccctttccctttagtgagggttaatgct

>ACA21_s1_SynSno_hyb10nt_mut1A

acctttgccaagccaatggcggctaactatctgaagaaccagccgatgtacgtgttccgt

aagacggagctcaagcactccaagaccgagctcaacttcaaggagtggcaaaaggccttt

accgatgtgatgggcatggacgagctgtacaagtaaactagtagcagacagatgcgccgt

ggataaattgttatatttcttgacgtagcttttaaaagaagttgcctgattatttctggc

agccgaattggcaagttagccacccgatccaatgcagttggcgcgccattcgatatcgga

ccctttccctttagtgagggttaatgct

>ACA21_s2_SynSno_hyb10nt_mut1A

acctttgccaagccaatggcggctaactatctgaagaaccagccgatgtacgtgttccgt

aagacggagctcaagcactccaagaccgagctcaacttcaaggagtggcaaaaggccttt

accgatgtgatgggcatggacgagctgtacaagtaaactagtagcagacagatgcgccgt

ggataaattgttataccaccgattgtacacccaaaaggaagttgcctgattatttctggc

agccgaattgctttcctagccacccgatccaatgcagttggcgcgccattcgatatcgga

ccctttccctttagtgagggttaatgct

>ACA22_s1_SynSno_hyb10nt_mut1A

acctttgccaagccaatggcggctaactatctgaagaaccagccgatgtacgtgttccgt

aagacggagctcaagcactccaagaccgagctcaacttcaaggagtggcaaaaggccttt

accgatgtgatgggcatggacgagctgtacaagtaaactagtagcagacagatgcgccgt

ggataaattgttataagggtcaaagtatcactgtgcagaagttgcctgattatttctggc

agccgaattgacatattagccacccgatccaatgcagttggcgcgccattcgatatcgga

ccctttccctttagtgagggttaatgct

>ACA22_s2_SynSno_hyb10nt_mut1A

acctttgccaagccaatggcggctaactatctgaagaaccagccgatgtacgtgttccgt

aagacggagctcaagcactccaagaccgagctcaacttcaaggagtggcaaaaggccttt

accgatgtgatgggcatggacgagctgtacaagtaaactagtagcagacagatgcgccgt

ggataaattgttatatctgaatacatagcagaggagagaagttgcctgattatttctggc

agccgaattatgataagagccacccgatccaatgcagttggcgcgccattcgatatcgga

ccctttccctttagtgagggttaatgct

>ACA23_s1_SynSno_hyb10nt_mut1A

acctttgccaagccaatggcggctaactatctgaagaaccagccgatgtacgtgttccgt

aagacggagctcaagcactccaagaccgagctcaacttcaaggagtggcaaaaggccttt

accgatgtgatgggcatggacgagctgtacaagtaaactagtagcagacagatgcgccgt

ggataaattgttatacatgatgttttaagcagccatggaagttgcctgattatttctggc

agccgaattgattgctaagccacccgatccaatgcagttggcgcgccattcgatatcgga

ccctttccctttagtgagggttaatgct

>ACA23_s2_SynSno_hyb10nt_mut1A

acctttgccaagccaatggcggctaactatctgaagaaccagccgatgtacgtgttccgt

aagacggagctcaagcactccaagaccgagctcaacttcaaggagtggcaaaaggccttt

accgatgtgatgggcatggacgagctgtacaagtaaactagtagcagacagatgcgccgt

ggataaattgttatagaatttggagtaactatgatccgaagttgcctgattatttctggc

agccgaattagcggcatagccacccgatccaatgcagttggcgcgccattcgatatcgga

ccctttccctttagtgagggttaatgct

>ACA24_s1_SynSno_hyb10nt_mut1A

acctttgccaagccaatggcggctaactatctgaagaaccagccgatgtacgtgttccgt

aagacggagctcaagcactccaagaccgagctcaacttcaaggagtggcaaaaggccttt

accgatgtgatgggcatggacgagctgtacaagtaaactagtagcagacagatgcgccgt

ggataaattgttatatggctaggaataaaagatacatgaagttgcctgattatttctggc

agccgaattttccagtaagccacccgatccaatgcagttggcgcgccattcgatatcgga

ccctttccctttagtgagggttaatgct

>ACA24_s2_SynSno_hyb10nt_mut1A

acctttgccaagccaatggcggctaactatctgaagaaccagccgatgtacgtgttccgt

aagacggagctcaagcactccaagaccgagctcaacttcaaggagtggcaaaaggccttt

accgatgtgatgggcatggacgagctgtacaagtaaactagtagcagacagatgcgccgt

ggataaattgttatatgtgcaagtctattgccaataagaagttgcctgattatttctggc

agccgaattattgttagagccacccgatccaatgcagttggcgcgccattcgatatcgga

ccctttccctttagtgagggttaatgct

>ACA25_s1_SynSno_hyb10nt_mut1A

acctttgccaagccaatggcggctaactatctgaagaaccagccgatgtacgtgttccgt

aagacggagctcaagcactccaagaccgagctcaacttcaaggagtggcaaaaggccttt

accgatgtgatgggcatggacgagctgtacaagtaaactagtagcagacagatgcgccgt

ggataaattgttatatcacagcgtttactttgaaatggaagttgcctgattatttctggc

agccgaattcacaaaaaagccacccgatccaatgcagttggcgcgccattcgatatcgga

ccctttccctttagtgagggttaatgct

>ACA25_s2_SynSno_hyb10nt_mut1A

acctttgccaagccaatggcggctaactatctgaagaaccagccgatgtacgtgttccgt

aagacggagctcaagcactccaagaccgagctcaacttcaaggagtggcaaaaggccttt

accgatgtgatgggcatggacgagctgtacaagtaaactagtagcagacagatgcgccgt

ggataaattgttatactcaggaaaataggagtgctatgaagttgcctgattatttctggc

agccgaattgagagcatagccacccgatccaatgcagttggcgcgccattcgatatcgga

ccctttccctttagtgagggttaatgct

>ACA27_s1_SynSno_hyb10nt_mut1A

acctttgccaagccaatggcggctaactatctgaagaaccagccgatgtacgtgttccgt

aagacggagctcaagcactccaagaccgagctcaacttcaaggagtggcaaaaggccttt

accgatgtgatgggcatggacgagctgtacaagtaaactagtagcagacagatgcgccgt

ggataaattgttatacacttgaatgtaaaagtgaaaagaagttgcctgattatttctggc

agccgaatttcgtaggcagccacccgatccaatgcagttggcgcgccattcgatatcgga

ccctttccctttagtgagggttaatgct

>ACA27_s2_SynSno_hyb10nt_mut1A

acctttgccaagccaatggcggctaactatctgaagaaccagccgatgtacgtgttccgt

aagacggagctcaagcactccaagaccgagctcaacttcaaggagtggcaaaaggccttt

accgatgtgatgggcatggacgagctgtacaagtaaactagtagcagacagatgcgccgt

ggataaattgttatatctgacaggatagttttaaaaggaagttgcctgattatttctggc

agccgaattctgttgccagccacccgatccaatgcagttggcgcgccattcgatatcgga

ccctttccctttagtgagggttaatgct

>ACA28_s1_SynSno_hyb10nt_mut1A

acctttgccaagccaatggcggctaactatctgaagaaccagccgatgtacgtgttccgt

aagacggagctcaagcactccaagaccgagctcaacttcaaggagtggcaaaaggccttt

accgatgtgatgggcatggacgagctgtacaagtaaactagtagcagacagatgcgccgt

ggataaattgttataaagctcaaattagagtgttgctgaagttgcctgattatttctggc

agccgaattcttgtcgaagccacccgatccaatgcagttggcgcgccattcgatatcgga

ccctttccctttagtgagggttaatgct

>ACA28_s2_SynSno_hyb10nt_mut1A

acctttgccaagccaatggcggctaactatctgaagaaccagccgatgtacgtgttccgt

aagacggagctcaagcactccaagaccgagctcaacttcaaggagtggcaaaaggccttt

accgatgtgatgggcatggacgagctgtacaagtaaactagtagcagacagatgcgccgt

ggataaattgttatagtctatataatagaataggttagaagttgcctgattatttctggc

agccgaattcggtgtagagccacccgatccaatgcagttggcgcgccattcgatatcgga

ccctttccctttagtgagggttaatgct

>ACA2a_s1_SynSno_hyb10nt_mut1A

acctttgccaagccaatggcggctaactatctgaagaaccagccgatgtacgtgttccgt

aagacggagctcaagcactccaagaccgagctcaacttcaaggagtggcaaaaggccttt

accgatgtgatgggcatggacgagctgtacaagtaaactagtagcagacagatgcgccgt

ggataaattgttatatcctgtttgatattgattcagggaagttgcctgattatttctggc

agccgaatttacgagtaagccacccgatccaatgcagttggcgcgccattcgatatcgga

ccctttccctttagtgagggttaatgct

>ACA2a_s2_SynSno_hyb10nt_mut1A

acctttgccaagccaatggcggctaactatctgaagaaccagccgatgtacgtgttccgt

aagacggagctcaagcactccaagaccgagctcaacttcaaggagtggcaaaaggccttt

accgatgtgatgggcatggacgagctgtacaagtaaactagtagcagacagatgcgccgt

ggataaattgttatatccaactgaatacagaccacaagaagttgcctgattatttctggc

agccgaattccgtctccagccacccgatccaatgcagttggcgcgccattcgatatcgga

ccctttccctttagtgagggttaatgct

>ACA3_s1_SynSno_hyb10nt_mut1A

acctttgccaagccaatggcggctaactatctgaagaaccagccgatgtacgtgttccgt

aagacggagctcaagcactccaagaccgagctcaacttcaaggagtggcaaaaggccttt

accgatgtgatgggcatggacgagctgtacaagtaaactagtagcagacagatgcgccgt

ggataaattgttataggactctagctagactctagccgaagttgcctgattatttctggc

agccgaattacataaaaagccacccgatccaatgcagttggcgcgccattcgatatcgga

ccctttccctttagtgagggttaatgct

>ACA1_SynSno_hyb10nt_mut1T

acctttgccaagccaatggcggctaactatctgaagaaccagccgatgtacgtgttccgt

aagacggagctcaagcactccaagaccgagctcaacttcaaggagtggcaaaaggccttt

accgatgtgatgggcatggacgagctgtacaagtaaactagtagcagacagatgcgccgt

ggataaattgttataggtctcattgttaagcataaccgaagttgcctgattatttctggc

agccgaattccctgtccagccacccgatccaatgcagttggcgcgccattcgatatcgga

ccctttccctttagtgagggttaatgct

>ACA10_s1_SynSno_hyb10nt_mut1T

acctttgccaagccaatggcggctaactatctgaagaaccagccgatgtacgtgttccgt

aagacggagctcaagcactccaagaccgagctcaacttcaaggagtggcaaaaggccttt

accgatgtgatgggcatggacgagctgtacaagtaaactagtagcagacagatgcgccgt

ggataaattgttatacctgaaaacattagctgagagagaagttgcctgattatttctggc

agccgaattccccctctagccacccgatccaatgcagttggcgcgccattcgatatcgga

ccctttccctttagtgagggttaatgct

>ACA10_s2_SynSno_hyb10nt_mut1T

acctttgccaagccaatggcggctaactatctgaagaaccagccgatgtacgtgttccgt

aagacggagctcaagcactccaagaccgagctcaacttcaaggagtggcaaaaggccttt

accgatgtgatgggcatggacgagctgtacaagtaaactagtagcagacagatgcgccgt

ggataaattgttataactcctaggattcgtgcattaggaagttgcctgattatttctggc

agccgaattagaccgtgagccacccgatccaatgcagttggcgcgccattcgatatcgga

ccctttccctttagtgagggttaatgct

>ACA13_SynSno_hyb10nt_mut1T

acctttgccaagccaatggcggctaactatctgaagaaccagccgatgtacgtgttccgt

aagacggagctcaagcactccaagaccgagctcaacttcaaggagtggcaaaaggccttt

accgatgtgatgggcatggacgagctgtacaagtaaactagtagcagacagatgcgccgt

ggataaattgttatacttttttgacttaacacaaagggaagttgcctgattatttctggc

agccgaattcatgtataagccacccgatccaatgcagttggcgcgccattcgatatcgga

ccctttccctttagtgagggttaatgct

>ACA14a_SynSno_hyb10nt_mut1T

acctttgccaagccaatggcggctaactatctgaagaaccagccgatgtacgtgttccgt

aagacggagctcaagcactccaagaccgagctcaacttcaaggagtggcaaaaggccttt

accgatgtgatgggcatggacgagctgtacaagtaaactagtagcagacagatgcgccgt

ggataaattgttataggtatgaaatttttggactttcgaagttgcctgattatttctggc

agccgaattgggaacacagccacccgatccaatgcagttggcgcgccattcgatatcgga

ccctttccctttagtgagggttaatgct

>ACA15_SynSno_hyb10nt_mut1T

acctttgccaagccaatggcggctaactatctgaagaaccagccgatgtacgtgttccgt

aagacggagctcaagcactccaagaccgagctcaacttcaaggagtggcaaaaggccttt

accgatgtgatgggcatggacgagctgtacaagtaaactagtagcagacagatgcgccgt

ggataaattgttatatggtgtctggtttattcggccagaagttgcctgattatttctggc

agccgaatttcgtgctaagccacccgatccaatgcagttggcgcgccattcgatatcgga

ccctttccctttagtgagggttaatgct

>ACA16_SynSno_hyb10nt_mut1T

acctttgccaagccaatggcggctaactatctgaagaaccagccgatgtacgtgttccgt

aagacggagctcaagcactccaagaccgagctcaacttcaaggagtggcaaaaggccttt

accgatgtgatgggcatggacgagctgtacaagtaaactagtagcagacagatgcgccgt

ggataaattgttatatcctttttgattcttcgataaggaagttgcctgattatttctggc

agccgaattcaaaatgtagccacccgatccaatgcagttggcgcgccattcgatatcgga

ccctttccctttagtgagggttaatgct

>ACA17_s1_SynSno_hyb10nt_mut1T

acctttgccaagccaatggcggctaactatctgaagaaccagccgatgtacgtgttccgt

aagacggagctcaagcactccaagaccgagctcaacttcaaggagtggcaaaaggccttt

accgatgtgatgggcatggacgagctgtacaagtaaactagtagcagacagatgcgccgt

ggataaattgttataacctaatgacttaacgcctctagaagttgcctgattatttctggc

agccgaattggcggtttagccacccgatccaatgcagttggcgcgccattcgatatcgga

ccctttccctttagtgagggttaatgct

>ACA17_s2_SynSno_hyb10nt_mut1T

acctttgccaagccaatggcggctaactatctgaagaaccagccgatgtacgtgttccgt

aagacggagctcaagcactccaagaccgagctcaacttcaaggagtggcaaaaggccttt

accgatgtgatgggcatggacgagctgtacaagtaaactagtagcagacagatgcgccgt

ggataaattgttatatatcaaacatttagcgtagacagaagttgcctgattatttctggc

agccgaattagttagcaagccacccgatccaatgcagttggcgcgccattcgatatcgga

ccctttccctttagtgagggttaatgct

>ACA19_s1_SynSno_hyb10nt_mut1T

acctttgccaagccaatggcggctaactatctgaagaaccagccgatgtacgtgttccgt

aagacggagctcaagcactccaagaccgagctcaacttcaaggagtggcaaaaggccttt

accgatgtgatgggcatggacgagctgtacaagtaaactagtagcagacagatgcgccgt

ggataaattgttatacataagaaatttaatgaaatgtgaagttgcctgattatttctggc

agccgaatttggattcaagccacccgatccaatgcagttggcgcgccattcgatatcgga

ccctttccctttagtgagggttaatgct

>ACA19_s2_SynSno_hyb10nt_mut1T

acctttgccaagccaatggcggctaactatctgaagaaccagccgatgtacgtgttccgt

aagacggagctcaagcactccaagaccgagctcaacttcaaggagtggcaaaaggccttt

accgatgtgatgggcatggacgagctgtacaagtaaactagtagcagacagatgcgccgt

ggataaattgttataaaggagactgtttaattagcctgaagttgcctgattatttctggc

agccgaattctgtccgcagccacccgatccaatgcagttggcgcgccattcgatatcgga

ccctttccctttagtgagggttaatgct

>ACA19_s3_SynSno_hyb10nt_mut1T

acctttgccaagccaatggcggctaactatctgaagaaccagccgatgtacgtgttccgt

aagacggagctcaagcactccaagaccgagctcaacttcaaggagtggcaaaaggccttt

accgatgtgatgggcatggacgagctgtacaagtaaactagtagcagacagatgcgccgt

ggataaattgttatacacataagaattatgaaatgtggaagttgcctgattatttctggc

agccgaattacgcctatagccacccgatccaatgcagttggcgcgccattcgatatcgga

ccctttccctttagtgagggttaatgct

>ACA20_SynSno_hyb10nt_mut1T

acctttgccaagccaatggcggctaactatctgaagaaccagccgatgtacgtgttccgt

aagacggagctcaagcactccaagaccgagctcaacttcaaggagtggcaaaaggccttt

accgatgtgatgggcatggacgagctgtacaagtaaactagtagcagacagatgcgccgt

ggataaattgttatacatgcgtatattaaataaatgggaagttgcctgattatttctggc

agccgaattctatgagcagccacccgatccaatgcagttggcgcgccattcgatatcgga

ccctttccctttagtgagggttaatgct

>ACA21_s1_SynSno_hyb10nt_mut1T

acctttgccaagccaatggcggctaactatctgaagaaccagccgatgtacgtgttccgt

aagacggagctcaagcactccaagaccgagctcaacttcaaggagtggcaaaaggccttt

accgatgtgatgggcatggacgagctgtacaagtaaactagtagcagacagatgcgccgt

ggataaattgttatatttcttgacgttgcttttaaaagaagttgcctgattatttctggc

agccgaattttcatgccagccacccgatccaatgcagttggcgcgccattcgatatcgga

ccctttccctttagtgagggttaatgct

>ACA21_s2_SynSno_hyb10nt_mut1T

acctttgccaagccaatggcggctaactatctgaagaaccagccgatgtacgtgttccgt

aagacggagctcaagcactccaagaccgagctcaacttcaaggagtggcaaaaggccttt

accgatgtgatgggcatggacgagctgtacaagtaaactagtagcagacagatgcgccgt

ggataaattgttataccaccgattgttcacccaaaaggaagttgcctgattatttctggc

agccgaattcctaccatagccacccgatccaatgcagttggcgcgccattcgatatcgga

ccctttccctttagtgagggttaatgct

>ACA22_s1_SynSno_hyb10nt_mut1T

acctttgccaagccaatggcggctaactatctgaagaaccagccgatgtacgtgttccgt

aagacggagctcaagcactccaagaccgagctcaacttcaaggagtggcaaaaggccttt

accgatgtgatgggcatggacgagctgtacaagtaaactagtagcagacagatgcgccgt

ggataaattgttataagggtcaaagtttcactgtgcagaagttgcctgattatttctggc

agccgaattctgagaccagccacccgatccaatgcagttggcgcgccattcgatatcgga

ccctttccctttagtgagggttaatgct

>ACA22_s2_SynSno_hyb10nt_mut1T

acctttgccaagccaatggcggctaactatctgaagaaccagccgatgtacgtgttccgt

aagacggagctcaagcactccaagaccgagctcaacttcaaggagtggcaaaaggccttt

accgatgtgatgggcatggacgagctgtacaagtaaactagtagcagacagatgcgccgt

ggataaattgttatatctgaatacattgcagaggagagaagttgcctgattatttctggc

agccgaattaagagtggagccacccgatccaatgcagttggcgcgccattcgatatcgga

ccctttccctttagtgagggttaatgct

>ACA23_s1_SynSno_hyb10nt_mut1T

acctttgccaagccaatggcggctaactatctgaagaaccagccgatgtacgtgttccgt

aagacggagctcaagcactccaagaccgagctcaacttcaaggagtggcaaaaggccttt

accgatgtgatgggcatggacgagctgtacaagtaaactagtagcagacagatgcgccgt

ggataaattgttatacatgatgtttttagcagccatggaagttgcctgattatttctggc

agccgaattagaatggcagccacccgatccaatgcagttggcgcgccattcgatatcgga

ccctttccctttagtgagggttaatgct

>ACA23_s2_SynSno_hyb10nt_mut1T

acctttgccaagccaatggcggctaactatctgaagaaccagccgatgtacgtgttccgt

aagacggagctcaagcactccaagaccgagctcaacttcaaggagtggcaaaaggccttt

accgatgtgatgggcatggacgagctgtacaagtaaactagtagcagacagatgcgccgt

ggataaattgttatagaatttggagttactatgatccgaagttgcctgattatttctggc

agccgaatttcagctccagccacccgatccaatgcagttggcgcgccattcgatatcgga

ccctttccctttagtgagggttaatgct

>ACA24_s1_SynSno_hyb10nt_mut1T

acctttgccaagccaatggcggctaactatctgaagaaccagccgatgtacgtgttccgt

aagacggagctcaagcactccaagaccgagctcaacttcaaggagtggcaaaaggccttt

accgatgtgatgggcatggacgagctgtacaagtaaactagtagcagacagatgcgccgt

ggataaattgttatatggctaggaattaaagatacatgaagttgcctgattatttctggc

agccgaatttttgccatagccacccgatccaatgcagttggcgcgccattcgatatcgga

ccctttccctttagtgagggttaatgct

>ACA24_s2_SynSno_hyb10nt_mut1T

acctttgccaagccaatggcggctaactatctgaagaaccagccgatgtacgtgttccgt

aagacggagctcaagcactccaagaccgagctcaacttcaaggagtggcaaaaggccttt

accgatgtgatgggcatggacgagctgtacaagtaaactagtagcagacagatgcgccgt

ggataaattgttatatgtgcaagtcttttgccaataagaagttgcctgattatttctggc

agccgaatttcggactgagccacccgatccaatgcagttggcgcgccattcgatatcgga

ccctttccctttagtgagggttaatgct

>ACA25_s1_SynSno_hyb10nt_mut1T

acctttgccaagccaatggcggctaactatctgaagaaccagccgatgtacgtgttccgt

aagacggagctcaagcactccaagaccgagctcaacttcaaggagtggcaaaaggccttt

accgatgtgatgggcatggacgagctgtacaagtaaactagtagcagacagatgcgccgt

ggataaattgttatatcacagcgttttctttgaaatggaagttgcctgattatttctggc

agccgaatttacacggcagccacccgatccaatgcagttggcgcgccattcgatatcgga

ccctttccctttagtgagggttaatgct

>ACA25_s2_SynSno_hyb10nt_mut1T

acctttgccaagccaatggcggctaactatctgaagaaccagccgatgtacgtgttccgt

aagacggagctcaagcactccaagaccgagctcaacttcaaggagtggcaaaaggccttt

accgatgtgatgggcatggacgagctgtacaagtaaactagtagcagacagatgcgccgt

ggataaattgttatactcaggaaaattggagtgctatgaagttgcctgattatttctggc

agccgaattagaaacgcagccacccgatccaatgcagttggcgcgccattcgatatcgga

ccctttccctttagtgagggttaatgct

>ACA27_s1_SynSno_hyb10nt_mut1T

acctttgccaagccaatggcggctaactatctgaagaaccagccgatgtacgtgttccgt

aagacggagctcaagcactccaagaccgagctcaacttcaaggagtggcaaaaggccttt

accgatgtgatgggcatggacgagctgtacaagtaaactagtagcagacagatgcgccgt

ggataaattgttatacacttgaatgttaaagtgaaaagaagttgcctgattatttctggc

agccgaattaaacactaagccacccgatccaatgcagttggcgcgccattcgatatcgga

ccctttccctttagtgagggttaatgct

>ACA27_s2_SynSno_hyb10nt_mut1T

acctttgccaagccaatggcggctaactatctgaagaaccagccgatgtacgtgttccgt

aagacggagctcaagcactccaagaccgagctcaacttcaaggagtggcaaaaggccttt

accgatgtgatgggcatggacgagctgtacaagtaaactagtagcagacagatgcgccgt

ggataaattgttatatctgacaggattgttttaaaaggaagttgcctgattatttctggc

agccgaatttcgacgcgagccacccgatccaatgcagttggcgcgccattcgatatcgga

ccctttccctttagtgagggttaatgct

>ACA28_s1_SynSno_hyb10nt_mut1T

acctttgccaagccaatggcggctaactatctgaagaaccagccgatgtacgtgttccgt

aagacggagctcaagcactccaagaccgagctcaacttcaaggagtggcaaaaggccttt

accgatgtgatgggcatggacgagctgtacaagtaaactagtagcagacagatgcgccgt

ggataaattgttataaagctcaaatttgagtgttgctgaagttgcctgattatttctggc

agccgaatttgactttcagccacccgatccaatgcagttggcgcgccattcgatatcgga

ccctttccctttagtgagggttaatgct

>ACA28_s2_SynSno_hyb10nt_mut1T

acctttgccaagccaatggcggctaactatctgaagaaccagccgatgtacgtgttccgt

aagacggagctcaagcactccaagaccgagctcaacttcaaggagtggcaaaaggccttt

accgatgtgatgggcatggacgagctgtacaagtaaactagtagcagacagatgcgccgt

ggataaattgttatagtctatataattgaataggttagaagttgcctgattatttctggc

agccgaatttcgttaagagccacccgatccaatgcagttggcgcgccattcgatatcgga

ccctttccctttagtgagggttaatgct

>ACA2a_s1_SynSno_hyb10nt_mut1T

acctttgccaagccaatggcggctaactatctgaagaaccagccgatgtacgtgttccgt

aagacggagctcaagcactccaagaccgagctcaacttcaaggagtggcaaaaggccttt

accgatgtgatgggcatggacgagctgtacaagtaaactagtagcagacagatgcgccgt

ggataaattgttatatcctgtttgattttgattcagggaagttgcctgattatttctggc

agccgaattctctcgttagccacccgatccaatgcagttggcgcgccattcgatatcgga

ccctttccctttagtgagggttaatgct

>ACA2a_s2_SynSno_hyb10nt_mut1T

acctttgccaagccaatggcggctaactatctgaagaaccagccgatgtacgtgttccgt

aagacggagctcaagcactccaagaccgagctcaacttcaaggagtggcaaaaggccttt

accgatgtgatgggcatggacgagctgtacaagtaaactagtagcagacagatgcgccgt

ggataaattgttatatccaactgaattcagaccacaagaagttgcctgattatttctggc

agccgaattgagtcaacagccacccgatccaatgcagttggcgcgccattcgatatcgga

ccctttccctttagtgagggttaatgct

>ACA3_s1_SynSno_hyb10nt_mut1T

acctttgccaagccaatggcggctaactatctgaagaaccagccgatgtacgtgttccgt

aagacggagctcaagcactccaagaccgagctcaacttcaaggagtggcaaaaggccttt

accgatgtgatgggcatggacgagctgtacaagtaaactagtagcagacagatgcgccgt

ggataaattgttataggactctagcttgactctagccgaagttgcctgattatttctggc

agccgaattcgcgtcgtagccacccgatccaatgcagttggcgcgccattcgatatcgga

ccctttccctttagtgagggttaatgct

>ACA1_SynSno_hyb10nt_remove1

acctttgccaagccaatggcggctaactatctgaagaaccagccgatgtacgtgttccgt

aagacggagctcaagcactccaagaccgagctcaacttcaaggagtggcaaaaggccttt

accgatgtgatgggcatggacgagctgtacaagtaaactagtagcagacagatgcgccgt

ggataaattgttataggtctcattgtaagcataaccgaagttgcctgattatttctggca

gccgaattagggcgcagagccacccgatccaatgcagttggcgcgccattcgatatcgga

ccctttccctttagtgagggttaatgct

>ACA10_s1_SynSno_hyb10nt_remove1

acctttgccaagccaatggcggctaactatctgaagaaccagccgatgtacgtgttccgt

aagacggagctcaagcactccaagaccgagctcaacttcaaggagtggcaaaaggccttt

accgatgtgatgggcatggacgagctgtacaagtaaactagtagcagacagatgcgccgt

ggataaattgttatacctgaaaacatagctgagagagaagttgcctgattatttctggca

gccgaattaaactttccagccacccgatccaatgcagttggcgcgccattcgatatcgga

ccctttccctttagtgagggttaatgct

>ACA10_s2_SynSno_hyb10nt_remove1

acctttgccaagccaatggcggctaactatctgaagaaccagccgatgtacgtgttccgt

aagacggagctcaagcactccaagaccgagctcaacttcaaggagtggcaaaaggccttt

accgatgtgatgggcatggacgagctgtacaagtaaactagtagcagacagatgcgccgt

ggataaattgttataactcctaggatcgtgcattaggaagttgcctgattatttctggca

gccgaattagctggaatagccacccgatccaatgcagttggcgcgccattcgatatcgga

ccctttccctttagtgagggttaatgct

>ACA13_SynSno_hyb10nt_remove1

acctttgccaagccaatggcggctaactatctgaagaaccagccgatgtacgtgttccgt

aagacggagctcaagcactccaagaccgagctcaacttcaaggagtggcaaaaggccttt

accgatgtgatgggcatggacgagctgtacaagtaaactagtagcagacagatgcgccgt

ggataaattgttatacttttttgactaacacaaagggaagttgcctgattatttctggca

gccgaattacagcgctaagccacccgatccaatgcagttggcgcgccattcgatatcgga

ccctttccctttagtgagggttaatgct

>ACA14a_SynSno_hyb10nt_remove1

acctttgccaagccaatggcggctaactatctgaagaaccagccgatgtacgtgttccgt

aagacggagctcaagcactccaagaccgagctcaacttcaaggagtggcaaaaggccttt

accgatgtgatgggcatggacgagctgtacaagtaaactagtagcagacagatgcgccgt

ggataaattgttataggtatgaaattttggactttcgaagttgcctgattatttctggca

gccgaattatttgcctgagccacccgatccaatgcagttggcgcgccattcgatatcgga

ccctttccctttagtgagggttaatgct

>ACA15_SynSno_hyb10nt_remove1

acctttgccaagccaatggcggctaactatctgaagaaccagccgatgtacgtgttccgt

aagacggagctcaagcactccaagaccgagctcaacttcaaggagtggcaaaaggccttt

accgatgtgatgggcatggacgagctgtacaagtaaactagtagcagacagatgcgccgt

ggataaattgttatatggtgtctggttattcggccagaagttgcctgattatttctggca

gccgaattagaagactgagccacccgatccaatgcagttggcgcgccattcgatatcgga

ccctttccctttagtgagggttaatgct

>ACA16_SynSno_hyb10nt_remove1

acctttgccaagccaatggcggctaactatctgaagaaccagccgatgtacgtgttccgt

aagacggagctcaagcactccaagaccgagctcaacttcaaggagtggcaaaaggccttt

accgatgtgatgggcatggacgagctgtacaagtaaactagtagcagacagatgcgccgt

ggataaattgttatatcctttttgatcttcgataaggaagttgcctgattatttctggca

gccgaattacacctgacagccacccgatccaatgcagttggcgcgccattcgatatcgga

ccctttccctttagtgagggttaatgct

>ACA17_s2_SynSno_hyb10nt_remove1

acctttgccaagccaatggcggctaactatctgaagaaccagccgatgtacgtgttccgt

aagacggagctcaagcactccaagaccgagctcaacttcaaggagtggcaaaaggccttt

accgatgtgatgggcatggacgagctgtacaagtaaactagtagcagacagatgcgccgt

ggataaattgttatatatcaaacattagcgtagacagaagttgcctgattatttctggca

gccgaattaaactccgtagccacccgatccaatgcagttggcgcgccattcgatatcgga

ccctttccctttagtgagggttaatgct

>ACA19_s1_SynSno_hyb10nt_remove1

acctttgccaagccaatggcggctaactatctgaagaaccagccgatgtacgtgttccgt

aagacggagctcaagcactccaagaccgagctcaacttcaaggagtggcaaaaggccttt

accgatgtgatgggcatggacgagctgtacaagtaaactagtagcagacagatgcgccgt

ggataaattgttatacataagaaattaatgaaatgtgaagttgcctgattatttctggca

gccgaattaactgacgcagccacccgatccaatgcagttggcgcgccattcgatatcgga

ccctttccctttagtgagggttaatgct

>ACA19_s2_SynSno_hyb10nt_remove1

acctttgccaagccaatggcggctaactatctgaagaaccagccgatgtacgtgttccgt

aagacggagctcaagcactccaagaccgagctcaacttcaaggagtggcaaaaggccttt

accgatgtgatgggcatggacgagctgtacaagtaaactagtagcagacagatgcgccgt

ggataaattgttataaaggagactgttaattagcctgaagttgcctgattatttctggca

gccgaattaagaggacgagccacccgatccaatgcagttggcgcgccattcgatatcgga

ccctttccctttagtgagggttaatgct

>ACA19_s3_SynSno_hyb10nt_remove1

acctttgccaagccaatggcggctaactatctgaagaaccagccgatgtacgtgttccgt

aagacggagctcaagcactccaagaccgagctcaacttcaaggagtggcaaaaggccttt

accgatgtgatgggcatggacgagctgtacaagtaaactagtagcagacagatgcgccgt

ggataaattgttatacacataagaatatgaaatgtggaagttgcctgattatttctggca

gccgaattaggaagaagagccacccgatccaatgcagttggcgcgccattcgatatcgga

ccctttccctttagtgagggttaatgct

>ACA20_SynSno_hyb10nt_remove1

acctttgccaagccaatggcggctaactatctgaagaaccagccgatgtacgtgttccgt

aagacggagctcaagcactccaagaccgagctcaacttcaaggagtggcaaaaggccttt

accgatgtgatgggcatggacgagctgtacaagtaaactagtagcagacagatgcgccgt

ggataaattgttatacatgcgtatataaataaatgggaagttgcctgattatttctggca

gccgaattaactgtagcagccacccgatccaatgcagttggcgcgccattcgatatcgga

ccctttccctttagtgagggttaatgct

>ACA21_s1_SynSno_hyb10nt_remove1

acctttgccaagccaatggcggctaactatctgaagaaccagccgatgtacgtgttccgt

aagacggagctcaagcactccaagaccgagctcaacttcaaggagtggcaaaaggccttt

accgatgtgatgggcatggacgagctgtacaagtaaactagtagcagacagatgcgccgt

ggataaattgttatatttcttgacgtgcttttaaaagaagttgcctgattatttctggca

gccgaattagtaagatcagccacccgatccaatgcagttggcgcgccattcgatatcgga

ccctttccctttagtgagggttaatgct

>ACA21_s2_SynSno_hyb10nt_remove1

acctttgccaagccaatggcggctaactatctgaagaaccagccgatgtacgtgttccgt

aagacggagctcaagcactccaagaccgagctcaacttcaaggagtggcaaaaggccttt

accgatgtgatgggcatggacgagctgtacaagtaaactagtagcagacagatgcgccgt

ggataaattgttataccaccgattgtcacccaaaaggaagttgcctgattatttctggca

gccgaattacggaagacagccacccgatccaatgcagttggcgcgccattcgatatcgga

ccctttccctttagtgagggttaatgct

>ACA22_s1_SynSno_hyb10nt_remove1

acctttgccaagccaatggcggctaactatctgaagaaccagccgatgtacgtgttccgt

aagacggagctcaagcactccaagaccgagctcaacttcaaggagtggcaaaaggccttt

accgatgtgatgggcatggacgagctgtacaagtaaactagtagcagacagatgcgccgt

ggataaattgttataagggtcaaagttcactgtgcagaagttgcctgattatttctggca

gccgaattagtgtacatagccacccgatccaatgcagttggcgcgccattcgatatcgga

ccctttccctttagtgagggttaatgct

>ACA22_s2_SynSno_hyb10nt_remove1

acctttgccaagccaatggcggctaactatctgaagaaccagccgatgtacgtgttccgt

aagacggagctcaagcactccaagaccgagctcaacttcaaggagtggcaaaaggccttt

accgatgtgatgggcatggacgagctgtacaagtaaactagtagcagacagatgcgccgt

ggataaattgttatatctgaatacatgcagaggagagaagttgcctgattatttctggca

gccgaattagatatgaaagccacccgatccaatgcagttggcgcgccattcgatatcgga

ccctttccctttagtgagggttaatgct

>ACA23_s1_SynSno_hyb10nt_remove1

acctttgccaagccaatggcggctaactatctgaagaaccagccgatgtacgtgttccgt

aagacggagctcaagcactccaagaccgagctcaacttcaaggagtggcaaaaggccttt

accgatgtgatgggcatggacgagctgtacaagtaaactagtagcagacagatgcgccgt

ggataaattgttatacatgatgttttagcagccatggaagttgcctgattatttctggca

gccgaattactctctggagccacccgatccaatgcagttggcgcgccattcgatatcgga

ccctttccctttagtgagggttaatgct

>ACA23_s2_SynSno_hyb10nt_remove1

acctttgccaagccaatggcggctaactatctgaagaaccagccgatgtacgtgttccgt

aagacggagctcaagcactccaagaccgagctcaacttcaaggagtggcaaaaggccttt

accgatgtgatgggcatggacgagctgtacaagtaaactagtagcagacagatgcgccgt

ggataaattgttatagaatttggagtactatgatccgaagttgcctgattatttctggca

gccgaattatattggcaagccacccgatccaatgcagttggcgcgccattcgatatcgga

ccctttccctttagtgagggttaatgct

>ACA24_s1_SynSno_hyb10nt_remove1

acctttgccaagccaatggcggctaactatctgaagaaccagccgatgtacgtgttccgt

aagacggagctcaagcactccaagaccgagctcaacttcaaggagtggcaaaaggccttt

accgatgtgatgggcatggacgagctgtacaagtaaactagtagcagacagatgcgccgt

ggataaattgttatatggctaggaataaagatacatgaagttgcctgattatttctggca

gccgaattaacttatagagccacccgatccaatgcagttggcgcgccattcgatatcgga

ccctttccctttagtgagggttaatgct

>ACA24_s2_SynSno_hyb10nt_remove1

acctttgccaagccaatggcggctaactatctgaagaaccagccgatgtacgtgttccgt

aagacggagctcaagcactccaagaccgagctcaacttcaaggagtggcaaaaggccttt

accgatgtgatgggcatggacgagctgtacaagtaaactagtagcagacagatgcgccgt

ggataaattgttatatgtgcaagtctttgccaataagaagttgcctgattatttctggca

gccgaattacactgttgagccacccgatccaatgcagttggcgcgccattcgatatcgga

ccctttccctttagtgagggttaatgct

>ACA25_s1_SynSno_hyb10nt_remove1

acctttgccaagccaatggcggctaactatctgaagaaccagccgatgtacgtgttccgt

aagacggagctcaagcactccaagaccgagctcaacttcaaggagtggcaaaaggccttt

accgatgtgatgggcatggacgagctgtacaagtaaactagtagcagacagatgcgccgt

ggataaattgttatatcacagcgtttctttgaaatggaagttgcctgattatttctggca

gccgaattaggcacgtaagccacccgatccaatgcagttggcgcgccattcgatatcgga

ccctttccctttagtgagggttaatgct

>ACA25_s2_SynSno_hyb10nt_remove1

acctttgccaagccaatggcggctaactatctgaagaaccagccgatgtacgtgttccgt

aagacggagctcaagcactccaagaccgagctcaacttcaaggagtggcaaaaggccttt

accgatgtgatgggcatggacgagctgtacaagtaaactagtagcagacagatgcgccgt

ggataaattgttatactcaggaaaatggagtgctatgaagttgcctgattatttctggca

gccgaattaacatttacagccacccgatccaatgcagttggcgcgccattcgatatcgga

ccctttccctttagtgagggttaatgct

>ACA27_s1_SynSno_hyb10nt_remove1

acctttgccaagccaatggcggctaactatctgaagaaccagccgatgtacgtgttccgt

aagacggagctcaagcactccaagaccgagctcaacttcaaggagtggcaaaaggccttt

accgatgtgatgggcatggacgagctgtacaagtaaactagtagcagacagatgcgccgt

ggataaattgttatacacttgaatgtaaagtgaaaagaagttgcctgattatttctggca

gccgaattacgccgtccagccacccgatccaatgcagttggcgcgccattcgatatcgga

ccctttccctttagtgagggttaatgct

>ACA27_s2_SynSno_hyb10nt_remove1

acctttgccaagccaatggcggctaactatctgaagaaccagccgatgtacgtgttccgt

aagacggagctcaagcactccaagaccgagctcaacttcaaggagtggcaaaaggccttt

accgatgtgatgggcatggacgagctgtacaagtaaactagtagcagacagatgcgccgt

ggataaattgttatatctgacaggatgttttaaaaggaagttgcctgattatttctggca

gccgaattaggactatgagccacccgatccaatgcagttggcgcgccattcgatatcgga

ccctttccctttagtgagggttaatgct

>ACA28_s1_SynSno_hyb10nt_remove1

acctttgccaagccaatggcggctaactatctgaagaaccagccgatgtacgtgttccgt

aagacggagctcaagcactccaagaccgagctcaacttcaaggagtggcaaaaggccttt

accgatgtgatgggcatggacgagctgtacaagtaaactagtagcagacagatgcgccgt

ggataaattgttataaagctcaaattgagtgttgctgaagttgcctgattatttctggca

gccgaattatgcttaaaagccacccgatccaatgcagttggcgcgccattcgatatcgga

ccctttccctttagtgagggttaatgct

>ACA28_s2_SynSno_hyb10nt_remove1

acctttgccaagccaatggcggctaactatctgaagaaccagccgatgtacgtgttccgt

aagacggagctcaagcactccaagaccgagctcaacttcaaggagtggcaaaaggccttt

accgatgtgatgggcatggacgagctgtacaagtaaactagtagcagacagatgcgccgt

ggataaattgttatagtctatataatgaataggttagaagttgcctgattatttctggca

gccgaattactttgacaagccacccgatccaatgcagttggcgcgccattcgatatcgga

ccctttccctttagtgagggttaatgct

>ACA2a_s1_SynSno_hyb10nt_remove1

acctttgccaagccaatggcggctaactatctgaagaaccagccgatgtacgtgttccgt

aagacggagctcaagcactccaagaccgagctcaacttcaaggagtggcaaaaggccttt

accgatgtgatgggcatggacgagctgtacaagtaaactagtagcagacagatgcgccgt

ggataaattgttatatcctgtttgatttgattcagggaagttgcctgattatttctggca

gccgaattaacatcaagagccacccgatccaatgcagttggcgcgccattcgatatcgga

ccctttccctttagtgagggttaatgct

>ACA2a_s2_SynSno_hyb10nt_remove1

acctttgccaagccaatggcggctaactatctgaagaaccagccgatgtacgtgttccgt

aagacggagctcaagcactccaagaccgagctcaacttcaaggagtggcaaaaggccttt

accgatgtgatgggcatggacgagctgtacaagtaaactagtagcagacagatgcgccgt

ggataaattgttatatccaactgaatcagaccacaagaagttgcctgattatttctggca

gccgaattacctactctagccacccgatccaatgcagttggcgcgccattcgatatcgga

ccctttccctttagtgagggttaatgct

>ACA3_s1_SynSno_hyb10nt_remove1

acctttgccaagccaatggcggctaactatctgaagaaccagccgatgtacgtgttccgt

aagacggagctcaagcactccaagaccgagctcaacttcaaggagtggcaaaaggccttt

accgatgtgatgggcatggacgagctgtacaagtaaactagtagcagacagatgcgccgt

ggataaattgttataggactctagctgactctagccgaagttgcctgattatttctggca

gccgaattatagatacgagccacccgatccaatgcagttggcgcgccattcgatatcgga

ccctttccctttagtgagggttaatgct

>AllrRNASites___rRNALSUL28S_1515_psi

gccgatgtacgtgttccgtaagacggagctcaagcactccaagaccgagctcaacttcaa

ggagtggcaaaaggcctttaccgatgtgatgggcatggacgagctgtacaagtaaactag

tagcagacagatgcgccgtggatggagcacgagcgcacgtgttaggacccgaaagatggt

gaactatgcctgggcagggcgaagccagaggaaactctgctagttaagccacccgatcca

atgcttaaggtcaggtcactgcttgacggagccatagttggcgcgccattcgatatcgga

ccctttccctttagtgagggttaatgct

>AllrRNASites___rRNALSUL28S_1561_psi

gccgatgtacgtgttccgtaagacggagctcaagcactccaagaccgagctcaacttcaa

ggagtggcaaaaggcctttaccgatgtgatgggcatggacgagctgtacaagtaaactag

tagcagacagatgcgccgtggatctgggcagggcgaagccagaggaaactctggtggagg

tccgtagcggtcctgacgtgcaaatcggtcgtccgaccctaagcgaagccacccgatcca

atgcttaaggtcaggtcactgcttgacggagccatagttggcgcgccattcgatatcgga

ccctttccctttagtgagggttaatgct

>AllrRNASites___rRNALSUL28S_1656_psi

gccgatgtacgtgttccgtaagacggagctcaagcactccaagaccgagctcaacttcaa

ggagtggcaaaaggcctttaccgatgtgatgggcatggacgagctgtacaagtaaactag

tagcagacagatgcgccgtggattaatcgaaccatctagtagctggttccctccgaagtt

tccctcaggatagctggcgctctcgcagacccgacgcatccaattcagccacccgatcca

atgcttaaggtcaggtcactgcttgacggagccatagttggcgcgccattcgatatcgga

ccctttccctttagtgagggttaatgct

>AllrRNASites___rRNALSUL28S_1662_psi

gccgatgtacgtgttccgtaagacggagctcaagcactccaagaccgagctcaacttcaa

ggagtggcaaaaggcctttaccgatgtgatgggcatggacgagctgtacaagtaaactag

tagcagacagatgcgccgtggataaccatctagtagctggttccctccgaagtttccctc

aggatagctggcgctctcgcagacccgacgcacccccgctcaatcgagccacccgatcca

atgcttaaggtcaggtcactgcttgacggagccatagttggcgcgccattcgatatcgga

ccctttccctttagtgagggttaatgct

>AllrRNASites___rRNALSUL28S_1723_psi

gccgatgtacgtgttccgtaagacggagctcaagcactccaagaccgagctcaacttcaa

ggagtggcaaaaggcctttaccgatgtgatgggcatggacgagctgtacaagtaaactag

tagcagacagatgcgccgtggatccgacgcacccccgccacgcagttttatccggtaaag

cgaatgattagaggtcttggggccgaaacgatctcaactaaaagcgagccacccgatcca

atgcttaaggtcaggtcactgcttgacggagccatagttggcgcgccattcgatatcgga

ccctttccctttagtgagggttaatgct

>AllrRNASites___rRNALSUL28S_1758_psi

gccgatgtacgtgttccgtaagacggagctcaagcactccaagaccgagctcaacttcaa

ggagtggcaaaaggcctttaccgatgtgatgggcatggacgagctgtacaagtaaactag

tagcagacagatgcgccgtggatagcgaatgattagaggtcttggggccgaaacgatctc

aacctattctcaaactttaaatgggtaagaagcccggccacgcttcagccacccgatcca

atgcttaaggtcaggtcactgcttgacggagccatagttggcgcgccattcgatatcgga

ccctttccctttagtgagggttaatgct

>AllrRNASites___rRNALSUL28S_1761_psi

gccgatgtacgtgttccgtaagacggagctcaagcactccaagaccgagctcaacttcaa

ggagtggcaaaaggcctttaccgatgtgatgggcatggacgagctgtacaagtaaactag

tagcagacagatgcgccgtggatgaatgattagaggtcttggggccgaaacgatctcaac

ctattctcaaactttaaatgggtaagaagcccggctcgagtggctcagccacccgatcca

atgcttaaggtcaggtcactgcttgacggagccatagttggcgcgccattcgatatcgga

ccctttccctttagtgagggttaatgct

>AllrRNASites___rRNALSUL28S_1771_psi

gccgatgtacgtgttccgtaagacggagctcaagcactccaagaccgagctcaacttcaa

ggagtggcaaaaggcctttaccgatgtgatgggcatggacgagctgtacaagtaaactag

tagcagacagatgcgccgtggataggtcttggggccgaaacgatctcaacctattctcaa

actttaaatgggtaagaagcccggctcgctggcgtggatcaataggagccacccgatcca

atgcttaaggtcaggtcactgcttgacggagccatagttggcgcgccattcgatatcgga

ccctttccctttagtgagggttaatgct

>AllrRNASites___rRNALSUL28S_1838_psi

gccgatgtacgtgttccgtaagacggagctcaagcactccaagaccgagctcaacttcaa

ggagtggcaaaaggcctttaccgatgtgatgggcatggacgagctgtacaagtaaactag

tagcagacagatgcgccgtggatggcgtggagccggggtggaatgcgagtgcctagtggg

ccacttttggtaagcagaactggcgctgcgggatgaaccgtgagcgagccacccgatcca

atgcttaaggtcaggtcactgcttgacggagccatagttggcgcgccattcgatatcgga

ccctttccctttagtgagggttaatgct

>AllrRNASites___rRNALSUL28S_1840_psi

gccgatgtacgtgttccgtaagacggagctcaagcactccaagaccgagctcaacttcaa

ggagtggcaaaaggcctttaccgatgtgatgggcatggacgagctgtacaagtaaactag

tagcagacagatgcgccgtggatcgtggagccggggtggaatgcgagtgcctagtgggcc

acttttggtaagcagaactggcgctgcgggatgaaccgtttgtataagccacccgatcca

atgcttaaggtcaggtcactgcttgacggagccatagttggcgcgccattcgatatcgga

ccctttccctttagtgagggttaatgct

>AllrRNASites___rRNALSUL28S_2485_psi

gccgatgtacgtgttccgtaagacggagctcaagcactccaagaccgagctcaacttcaa

ggagtggcaaaaggcctttaccgatgtgatgggcatggacgagctgtacaagtaaactag

tagcagacagatgcgccgtggatttccgaagggacgggcgatggcctccgttgccctcgg

ccgatcgaaagggagtcgggttcagatccccgaatccgcactcgccagccacccgatcca

atgcttaaggtcaggtcactgcttgacggagccatagttggcgcgccattcgatatcgga

ccctttccctttagtgagggttaatgct

>AllrRNASites___rRNALSUL28S_3606_psi

gccgatgtacgtgttccgtaagacggagctcaagcactccaagaccgagctcaacttcaa

ggagtggcaaaaggcctttaccgatgtgatgggcatggacgagctgtacaagtaaactag

tagcagacagatgcgccgtggatagcagccgacttagaactggtgcggaccaggggaatc

cgactgtttaattaaaacaaagcatcgcgaaggcccgctacgccgtagccacccgatcca

atgcttaaggtcaggtcactgcttgacggagccatagttggcgcgccattcgatatcgga

ccctttccctttagtgagggttaatgct

>AllrRNASites___rRNALSUL28S_3608_psi

gccgatgtacgtgttccgtaagacggagctcaagcactccaagaccgagctcaacttcaa

ggagtggcaaaaggcctttaccgatgtgatgggcatggacgagctgtacaagtaaactag

tagcagacagatgcgccgtggatcagccgacttagaactggtgcggaccaggggaatccg

actgtttaattaaaacaaagcatcgcgaaggcccgcggcaccctgtagccacccgatcca

atgcttaaggtcaggtcactgcttgacggagccatagttggcgcgccattcgatatcgga

ccctttccctttagtgagggttaatgct

>AllrRNASites___rRNALSUL28S_3664_psi

gccgatgtacgtgttccgtaagacggagctcaagcactccaagaccgagctcaacttcaa

ggagtggcaaaaggcctttaccgatgtgatgggcatggacgagctgtacaagtaaactag

tagcagacagatgcgccgtggatgcatcgcgaaggcccgcggcgggtgttgacgcgatgt

gatttctgcccagtgctctgaatgtcaaagtgaagaaatcaccgaaagccacccgatcca

atgcttaaggtcaggtcactgcttgacggagccatagttggcgcgccattcgatatcgga

ccctttccctttagtgagggttaatgct

>AllrRNASites___rRNALSUL28S_3684_psi

gccgatgtacgtgttccgtaagacggagctcaagcactccaagaccgagctcaacttcaa

ggagtggcaaaaggcctttaccgatgtgatgggcatggacgagctgtacaagtaaactag

tagcagacagatgcgccgtggatgggtgttgacgcgatgtgatttctgcccagtgctctg

aatgtcaaagtgaagaaattcaatgaagcgcgggtaaaccacaaacagccacccgatcca

atgcttaaggtcaggtcactgcttgacggagccatagttggcgcgccattcgatatcgga

ccctttccctttagtgagggttaatgct

>AllrRNASites___rRNALSUL28S_3699_psi

gccgatgtacgtgttccgtaagacggagctcaagcactccaagaccgagctcaacttcaa

ggagtggcaaaaggcctttaccgatgtgatgggcatggacgagctgtacaagtaaactag

tagcagacagatgcgccgtggatgtgatttctgcccagtgctctgaatgtcaaagtgaag

aaattcaatgaagcgcgggtaaacggcgggagtaactagcgatcgtagccacccgatcca

atgcttaaggtcaggtcactgcttgacggagccatagttggcgcgccattcgatatcgga

ccctttccctttagtgagggttaatgct

>AllrRNASites___rRNALSUL28S_3703_psi

gccgatgtacgtgttccgtaagacggagctcaagcactccaagaccgagctcaacttcaa

ggagtggcaaaaggcctttaccgatgtgatgggcatggacgagctgtacaagtaaactag

tagcagacagatgcgccgtggattttctgcccagtgctctgaatgtcaaagtgaagaaat

tcaatgaagcgcgggtaaacggcgggagtaactatgactctaccaaagccacccgatcca

atgcttaaggtcaggtcactgcttgacggagccatagttggcgcgccattcgatatcgga

ccctttccctttagtgagggttaatgct

>AllrRNASites___rRNALSUL28S_3727_psi

gccgatgtacgtgttccgtaagacggagctcaagcactccaagaccgagctcaacttcaa

ggagtggcaaaaggcctttaccgatgtgatgggcatggacgagctgtacaagtaaactag

tagcagacagatgcgccgtggataaagtgaagaaattcaatgaagcgcgggtaaacggcg

ggagtaactatgactctcttaaggtagccaaatgcctcaatgcaggagccacccgatcca

atgcttaaggtcaggtcactgcttgacggagccatagttggcgcgccattcgatatcgga

ccctttccctttagtgagggttaatgct

>AllrRNASites___rRNALSUL28S_3731_psi

gccgatgtacgtgttccgtaagacggagctcaagcactccaagaccgagctcaacttcaa

ggagtggcaaaaggcctttaccgatgtgatgggcatggacgagctgtacaagtaaactag

tagcagacagatgcgccgtggattgaagaaattcaatgaagcgcgggtaaacggcgggag

taactatgactctcttaaggtagccaaatgcctcgtcacaaagctcagccacccgatcca

atgcttaaggtcaggtcactgcttgacggagccatagttggcgcgccattcgatatcgga

ccctttccctttagtgagggttaatgct

>AllrRNASites___rRNALSUL28S_3733_psi

gccgatgtacgtgttccgtaagacggagctcaagcactccaagaccgagctcaacttcaa

ggagtggcaaaaggcctttaccgatgtgatgggcatggacgagctgtacaagtaaactag

tagcagacagatgcgccgtggataagaaattcaatgaagcgcgggtaaacggcgggagta

actatgactctcttaaggtagccaaatgcctcgtcatcgctttgtcagccacccgatcca

atgcttaaggtcaggtcactgcttgacggagccatagttggcgcgccattcgatatcgga

ccctttccctttagtgagggttaatgct

>AllrRNASites___rRNALSUL28S_3737_psi

gccgatgtacgtgttccgtaagacggagctcaagcactccaagaccgagctcaacttcaa

ggagtggcaaaaggcctttaccgatgtgatgggcatggacgagctgtacaagtaaactag

tagcagacagatgcgccgtggataattcaatgaagcgcgggtaaacggcgggagtaacta

tgactctcttaaggtagccaaatgcctcgtcatctaatgtgagtatagccacccgatcca

atgcttaaggtcaggtcactgcttgacggagccatagttggcgcgccattcgatatcgga

ccctttccctttagtgagggttaatgct

>AllrRNASites___rRNALSUL28S_3739_psi

gccgatgtacgtgttccgtaagacggagctcaagcactccaagaccgagctcaacttcaa

ggagtggcaaaaggcctttaccgatgtgatgggcatggacgagctgtacaagtaaactag

tagcagacagatgcgccgtggatttcaatgaagcgcgggtaaacggcgggagtaactatg

actctcttaaggtagccaaatgcctcgtcatctaattaacggctacagccacccgatcca

atgcttaaggtcaggtcactgcttgacggagccatagttggcgcgccattcgatatcgga

ccctttccctttagtgagggttaatgct

>AllrRNASites___rRNALSUL28S_3791_psi

gccgatgtacgtgttccgtaagacggagctcaagcactccaagaccgagctcaacttcaa

ggagtggcaaaaggcctttaccgatgtgatgggcatggacgagctgtacaagtaaactag

tagcagacagatgcgccgtggatccaaatgcctcgtcatctaattagtgacgcgcatgaa

tggatgaacgagattcccactgtccctacctactatccttcattcaagccacccgatcca

atgcttaaggtcaggtcactgcttgacggagccatagttggcgcgccattcgatatcgga

ccctttccctttagtgagggttaatgct

>AllrRNASites___rRNALSUL28S_3813_psi

gccgatgtacgtgttccgtaagacggagctcaagcactccaagaccgagctcaacttcaa

ggagtggcaaaaggcctttaccgatgtgatgggcatggacgagctgtacaagtaaactag

tagcagacagatgcgccgtggatagtgacgcgcatgaatggatgaacgagattcccactg

tccctacctactatccagcgaaaccacagccaagggaatggcgaacagccacccgatcca

atgcttaaggtcaggtcactgcttgacggagccatagttggcgcgccattcgatatcgga

ccctttccctttagtgagggttaatgct

>AllrRNASites___rRNALSUL28S_3820_psi

gccgatgtacgtgttccgtaagacggagctcaagcactccaagaccgagctcaacttcaa

ggagtggcaaaaggcctttaccgatgtgatgggcatggacgagctgtacaagtaaactag

tagcagacagatgcgccgtggatcgcatgaatggatgaacgagattcccactgtccctac

ctactatccagcgaaaccacagccaagggaacgggcttgtcggcctagccacccgatcca

atgcttaaggtcaggtcactgcttgacggagccatagttggcgcgccattcgatatcgga

ccctttccctttagtgagggttaatgct

>AllrRNASites___rRNALSUL28S_3822_psi

gccgatgtacgtgttccgtaagacggagctcaagcactccaagaccgagctcaacttcaa

ggagtggcaaaaggcctttaccgatgtgatgggcatggacgagctgtacaagtaaactag

tagcagacagatgcgccgtggatcatgaatggatgaacgagattcccactgtccctacct

actatccagcgaaaccacagccaagggaacgggcttggcgaattgaagccacccgatcca

atgcttaaggtcaggtcactgcttgacggagccatagttggcgcgccattcgatatcgga

ccctttccctttagtgagggttaatgct

>AllrRNASites___rRNALSUL28S_3853_psi

gccgatgtacgtgttccgtaagacggagctcaagcactccaagaccgagctcaacttcaa

ggagtggcaaaaggcctttaccgatgtgatgggcatggacgagctgtacaagtaaactag

tagcagacagatgcgccgtggatctacctactatccagcgaaaccacagccaagggaacg

ggcttggcggaatcagcggggaaagaagaccctgttgattggactgagccacccgatcca

atgcttaaggtcaggtcactgcttgacggagccatagttggcgcgccattcgatatcgga

ccctttccctttagtgagggttaatgct

>AllrRNASites___rRNALSUL28S_3889_psi

gccgatgtacgtgttccgtaagacggagctcaagcactccaagaccgagctcaacttcaa

ggagtggcaaaaggcctttaccgatgtgatgggcatggacgagctgtacaagtaaactag

tagcagacagatgcgccgtggatgggcttggcggaatcagcggggaaagaagaccctgtt

gagcttgactctagtctggcacggtgaagagacatgagccacgtgaagccacccgatcca

atgcttaaggtcaggtcactgcttgacggagccatagttggcgcgccattcgatatcgga

ccctttccctttagtgagggttaatgct

>AllrRNASites___rRNALSUL28S_3928_psi

gccgatgtacgtgttccgtaagacggagctcaagcactccaagaccgagctcaacttcaa

ggagtggcaaaaggcctttaccgatgtgatgggcatggacgagctgtacaagtaaactag

tagcagacagatgcgccgtggatgcttgactctagtctggcacggtgaagagacatgaga

ggtgtagaataagtgggaggcccccggcgcccccccggctatccgaagccacccgatcca

atgcttaaggtcaggtcactgcttgacggagccatagttggcgcgccattcgatatcgga

ccctttccctttagtgagggttaatgct

>AllrRNASites___rRNALSUL28S_4253_psi

gccgatgtacgtgttccgtaagacggagctcaagcactccaagaccgagctcaacttcaa

ggagtggcaaaaggcctttaccgatgtgatgggcatggacgagctgtacaagtaaactag

tagcagacagatgcgccgtggatcagaaacctcccgtggagcagaagggcaaaagctcgc

ttgatcttgattttcagtacgaatacagaccgtgaaaggaaactccagccacccgatcca

atgcttaaggtcaggtcactgcttgacggagccatagttggcgcgccattcgatatcgga

ccctttccctttagtgagggttaatgct

>AllrRNASites___rRNALSUL28S_4256_psi

gccgatgtacgtgttccgtaagacggagctcaagcactccaagaccgagctcaacttcaa

ggagtggcaaaaggcctttaccgatgtgatgggcatggacgagctgtacaagtaaactag

tagcagacagatgcgccgtggataaacctcccgtggagcagaagggcaaaagctcgcttg

atcttgattttcagtacgaatacagaccgtgaaagcggaccccttcagccacccgatcca

atgcttaaggtcaggtcactgcttgacggagccatagttggcgcgccattcgatatcgga

ccctttccctttagtgagggttaatgct

>AllrRNASites___rRNALSUL28S_4259_psi

gccgatgtacgtgttccgtaagacggagctcaagcactccaagaccgagctcaacttcaa

ggagtggcaaaaggcctttaccgatgtgatgggcatggacgagctgtacaagtaaactag

tagcagacagatgcgccgtggatcctcccgtggagcagaagggcaaaagctcgcttgatc

ttgattttcagtacgaatacagaccgtgaaagcggggcccgattacagccacccgatcca

atgcttaaggtcaggtcactgcttgacggagccatagttggcgcgccattcgatatcgga

ccctttccctttagtgagggttaatgct

>AllrRNASites___rRNALSUL28S_4272_psi

gccgatgtacgtgttccgtaagacggagctcaagcactccaagaccgagctcaacttcaa

ggagtggcaaaaggcctttaccgatgtgatgggcatggacgagctgtacaagtaaactag

tagcagacagatgcgccgtggatagaagggcaaaagctcgcttgatcttgattttcagta

cgaatacagaccgtgaaagcggggcctcacgatccttcggcagtaaagccacccgatcca

atgcttaaggtcaggtcactgcttgacggagccatagttggcgcgccattcgatatcgga

ccctttccctttagtgagggttaatgct

>AllrRNASites___rRNALSUL28S_4313_psi

gccgatgtacgtgttccgtaagacggagctcaagcactccaagaccgagctcaacttcaa

ggagtggcaaaaggcctttaccgatgtgatgggcatggacgagctgtacaagtaaactag

tagcagacagatgcgccgtggattacagaccgtgaaagcggggcctcacgatccttctga

ccttttgggttttaagcaggaggtgtcagaaaagttaccacaggaaagccacccgatcca

atgcttaaggtcaggtcactgcttgacggagccatagttggcgcgccattcgatatcgga

ccctttccctttagtgagggttaatgct

>AllrRNASites___rRNALSUL28S_4321_psi

gccgatgtacgtgttccgtaagacggagctcaagcactccaagaccgagctcaacttcaa

ggagtggcaaaaggcctttaccgatgtgatgggcatggacgagctgtacaagtaaactag

tagcagacagatgcgccgtggatgtgaaagcggggcctcacgatccttctgaccttttgg

gttttaagcaggaggtgtcagaaaagttaccacagggacgtacggtagccacccgatcca

atgcttaaggtcaggtcactgcttgacggagccatagttggcgcgccattcgatatcgga

ccctttccctttagtgagggttaatgct

>AllrRNASites___rRNALSUL28S_4363_psi

gccgatgtacgtgttccgtaagacggagctcaagcactccaagaccgagctcaacttcaa

ggagtggcaaaaggcctttaccgatgtgatgggcatggacgagctgtacaagtaaactag

tagcagacagatgcgccgtggataagcaggaggtgtcagaaaagttaccacagggataac

tggcttgtggcggccaagcgttcatagcgacgtcgcttaaaatagtagccacccgatcca

atgcttaaggtcaggtcactgcttgacggagccatagttggcgcgccattcgatatcgga

ccctttccctttagtgagggttaatgct

>AllrRNASites___rRNALSUL28S_4380_psi

gccgatgtacgtgttccgtaagacggagctcaagcactccaagaccgagctcaacttcaa

ggagtggcaaaaggcctttaccgatgtgatgggcatggacgagctgtacaagtaaactag

tagcagacagatgcgccgtggataaagttaccacagggataactggcttgtggcggccaa

gcgttcatagcgacgtcgctttttgatccttcgatgtcgactttttagccacccgatcca

atgcttaaggtcaggtcactgcttgacggagccatagttggcgcgccattcgatatcgga

ccctttccctttagtgagggttaatgct

>AllrRNASites___rRNALSUL28S_4391_psi

gccgatgtacgtgttccgtaagacggagctcaagcactccaagaccgagctcaacttcaa

ggagtggcaaaaggcctttaccgatgtgatgggcatggacgagctgtacaagtaaactag

tagcagacagatgcgccgtggatagggataactggcttgtggcggccaagcgttcatagc

gacgtcgctttttgatccttcgatgtcggctcttcctacgacggggagccacccgatcca

atgcttaaggtcaggtcactgcttgacggagccatagttggcgcgccattcgatatcgga

ccctttccctttagtgagggttaatgct

>AllrRNASites___rRNALSUL28S_4402_psi

gccgatgtacgtgttccgtaagacggagctcaagcactccaagaccgagctcaacttcaa

ggagtggcaaaaggcctttaccgatgtgatgggcatggacgagctgtacaagtaaactag

tagcagacagatgcgccgtggatgcttgtggcggccaagcgttcatagcgacgtcgcttt

ttgatccttcgatgtcggctcttcctatcattgtgaaggttatttgagccacccgatcca

atgcttaaggtcaggtcactgcttgacggagccatagttggcgcgccattcgatatcgga

ccctttccctttagtgagggttaatgct

>AllrRNASites___rRNALSUL28S_4417_psi

gccgatgtacgtgttccgtaagacggagctcaagcactccaagaccgagctcaacttcaa

ggagtggcaaaaggcctttaccgatgtgatgggcatggacgagctgtacaagtaaactag

tagcagacagatgcgccgtggatgcgttcatagcgacgtcgctttttgatccttcgatgt

cggctcttcctatcattgtgaagcagaattcgccaagcccaacaatagccacccgatcca

atgcttaaggtcaggtcactgcttgacggagccatagttggcgcgccattcgatatcgga

ccctttccctttagtgagggttaatgct

>AllrRNASites___rRNALSUL28S_4431_psi

gccgatgtacgtgttccgtaagacggagctcaagcactccaagaccgagctcaacttcaa

ggagtggcaaaaggcctttaccgatgtgatgggcatggacgagctgtacaagtaaactag

tagcagacagatgcgccgtggatgtcgctttttgatccttcgatgtcggctcttcctatc

attgtgaagcagaattcgccaagcgttggattgttcacctgatagaagccacccgatcca

atgcttaaggtcaggtcactgcttgacggagccatagttggcgcgccattcgatatcgga

ccctttccctttagtgagggttaatgct

>AllrRNASites___rRNALSUL28S_4460_psi

gccgatgtacgtgttccgtaagacggagctcaagcactccaagaccgagctcaacttcaa

ggagtggcaaaaggcctttaccgatgtgatgggcatggacgagctgtacaagtaaactag

tagcagacagatgcgccgtggatttcctatcattgtgaagcagaattcgccaagcgttgg

attgttcacccactaatagggaacgtgagctgggtttatccagactagccacccgatcca

atgcttaaggtcaggtcactgcttgacggagccatagttggcgcgccattcgatatcgga

ccctttccctttagtgagggttaatgct

>AllrRNASites___rRNALSUL28S_4481_psi

gccgatgtacgtgttccgtaagacggagctcaagcactccaagaccgagctcaacttcaa

ggagtggcaaaaggcctttaccgatgtgatgggcatggacgagctgtacaagtaaactag

tagcagacagatgcgccgtggatattcgccaagcgttggattgttcacccactaataggg

aacgtgagctgggtttagaccgtcgtgagacaggttagtttaggcaagccacccgatcca

atgcttaaggtcaggtcactgcttgacggagccatagttggcgcgccattcgatatcgga

ccctttccctttagtgagggttaatgct

>AllrRNASites___rRNALSUL28S_4491_psi

gccgatgtacgtgttccgtaagacggagctcaagcactccaagaccgagctcaacttcaa

ggagtggcaaaaggcctttaccgatgtgatgggcatggacgagctgtacaagtaaactag

tagcagacagatgcgccgtggatcgttggattgttcacccactaatagggaacgtgagct

gggtttagaccgtcgtgagacaggttagttttaccctaagtatgcgagccacccgatcca

atgcttaaggtcaggtcactgcttgacggagccatagttggcgcgccattcgatatcgga

ccctttccctttagtgagggttaatgct

>AllrRNASites___rRNALSUL28S_4512_psi

gccgatgtacgtgttccgtaagacggagctcaagcactccaagaccgagctcaacttcaa

ggagtggcaaaaggcctttaccgatgtgatgggcatggacgagctgtacaagtaaactag

tagcagacagatgcgccgtggatatagggaacgtgagctgggtttagaccgtcgtgagac

aggttagttttaccctactgatgatgtgttgttgccattgtaccggagccacccgatcca

atgcttaaggtcaggtcactgcttgacggagccatagttggcgcgccattcgatatcgga

ccctttccctttagtgagggttaatgct

>AllrRNASites___rRNALSUL28S_4529_psi

gccgatgtacgtgttccgtaagacggagctcaagcactccaagaccgagctcaacttcaa

ggagtggcaaaaggcctttaccgatgtgatgggcatggacgagctgtacaagtaaactag

tagcagacagatgcgccgtggatggtttagaccgtcgtgagacaggttagttttacccta

ctgatgatgtgttgttgccatggtaatcctgctcagtaatcttaatagccacccgatcca

atgcttaaggtcaggtcactgcttgacggagccatagttggcgcgccattcgatatcgga

ccctttccctttagtgagggttaatgct

>AllrRNASites___rRNALSUL28S_4536_psi

gccgatgtacgtgttccgtaagacggagctcaagcactccaagaccgagctcaacttcaa

ggagtggcaaaaggcctttaccgatgtgatgggcatggacgagctgtacaagtaaactag

tagcagacagatgcgccgtggataccgtcgtgagacaggttagttttaccctactgatga

tgtgttgttgccatggtaatcctgctcagtacgagaggagaaatgcagccacccgatcca

atgcttaaggtcaggtcactgcttgacggagccatagttggcgcgccattcgatatcgga

ccctttccctttagtgagggttaatgct

>AllrRNASites___rRNALSUL28S_4539_psi

gccgatgtacgtgttccgtaagacggagctcaagcactccaagaccgagctcaacttcaa

ggagtggcaaaaggcctttaccgatgtgatgggcatggacgagctgtacaagtaaactag

tagcagacagatgcgccgtggatgtcgtgagacaggttagttttaccctactgatgatgt

gttgttgccatggtaatcctgctcagtacgagaggaacagccatgaagccacccgatcca

atgcttaaggtcaggtcactgcttgacggagccatagttggcgcgccattcgatatcgga

ccctttccctttagtgagggttaatgct

>AllrRNASites___rRNALSUL28S_4588_psi

gccgatgtacgtgttccgtaagacggagctcaagcactccaagaccgagctcaacttcaa

ggagtggcaaaaggcctttaccgatgtgatgggcatggacgagctgtacaagtaaactag

tagcagacagatgcgccgtggatgtaatcctgctcagtacgagaggaaccgcaggttcag

acatttggtgtatgtgcttggctgaggagccaatggggaagcatgcagccacccgatcca

atgcttaaggtcaggtcactgcttgacggagccatagttggcgcgccattcgatatcgga

ccctttccctttagtgagggttaatgct

>AllrRNASites___rRNALSUL28S_4596_psi

gccgatgtacgtgttccgtaagacggagctcaagcactccaagaccgagctcaacttcaa

ggagtggcaaaaggcctttaccgatgtgatgggcatggacgagctgtacaagtaaactag

tagcagacagatgcgccgtggatgctcagtacgagaggaaccgcaggttcagacatttgg

tgtatgtgcttggctgaggagccaatggggcgaagctagcccctttagccacccgatcca

atgcttaaggtcaggtcactgcttgacggagccatagttggcgcgccattcgatatcgga

ccctttccctttagtgagggttaatgct

>AllrRNASites___rRNALSUL28S_4633_psi

gccgatgtacgtgttccgtaagacggagctcaagcactccaagaccgagctcaacttcaa

ggagtggcaaaaggcctttaccgatgtgatgggcatggacgagctgtacaagtaaactag

tagcagacagatgcgccgtggattgtatgtgcttggctgaggagccaatggggcgaagct

accatctgtgggattatgactgaacgcctctaagtcaggttgcgcgagccacccgatcca

atgcttaaggtcaggtcactgcttgacggagccatagttggcgcgccattcgatatcgga

ccctttccctttagtgagggttaatgct

>AllrRNASites___rRNALSUL28S_4649_psi

gccgatgtacgtgttccgtaagacggagctcaagcactccaagaccgagctcaacttcaa

ggagtggcaaaaggcctttaccgatgtgatgggcatggacgagctgtacaagtaaactag

tagcagacagatgcgccgtggataggagccaatggggcgaagctaccatctgtgggatta

tgactgaacgcctctaagtcagaatcccgcccaggcgaaacataaaagccacccgatcca

atgcttaaggtcaggtcactgcttgacggagccatagttggcgcgccattcgatatcgga

ccctttccctttagtgagggttaatgct

>AllrRNASites___rRNALSUL28S_4927_psi

gccgatgtacgtgttccgtaagacggagctcaagcactccaagaccgagctcaacttcaa

ggagtggcaaaaggcctttaccgatgtgatgggcatggacgagctgtacaagtaaactag

tagcagacagatgcgccgtggatgtcacgcaccgcacgttcgtggggaacctggcgctaa

accattcgtagacgacctgcttctgggtcggggtttcggtccaatcagccacccgatcca

atgcttaaggtcaggtcactgcttgacggagccatagttggcgcgccattcgatatcgga

ccctttccctttagtgagggttaatgct

>AllrRNASites___rRNALSUL28S_4928_psi

gccgatgtacgtgttccgtaagacggagctcaagcactccaagaccgagctcaacttcaa

ggagtggcaaaaggcctttaccgatgtgatgggcatggacgagctgtacaagtaaactag

tagcagacagatgcgccgtggattcacgcaccgcacgttcgtggggaacctggcgctaaa

ccattcgtagacgacctgcttctgggtcggggtttcgtcttaaaggagccacccgatcca

atgcttaaggtcaggtcactgcttgacggagccatagttggcgcgccattcgatatcgga

ccctttccctttagtgagggttaatgct

>AllrRNASites___rRNALSUL28S_4956_psi

gccgatgtacgtgttccgtaagacggagctcaagcactccaagaccgagctcaacttcaa

ggagtggcaaaaggcctttaccgatgtgatgggcatggacgagctgtacaagtaaactag

tagcagacagatgcgccgtggatggcgctaaaccattcgtagacgacctgcttctgggtc

ggggtttcgtacgtagcagagcagctccctcgctgcgaaggatgagagccacccgatcca

atgcttaaggtcaggtcactgcttgacggagccatagttggcgcgccattcgatatcgga

ccctttccctttagtgagggttaatgct

>AllrRNASites___rRNALSUL28S_4965_psi

gccgatgtacgtgttccgtaagacggagctcaagcactccaagaccgagctcaacttcaa

ggagtggcaaaaggcctttaccgatgtgatgggcatggacgagctgtacaagtaaactag

tagcagacagatgcgccgtggatccattcgtagacgacctgcttctgggtcggggtttcg

tacgtagcagagcagctccctcgctgcgatctattgaaggtcatagagccacccgatcca

atgcttaaggtcaggtcactgcttgacggagccatagttggcgcgccattcgatatcgga

ccctttccctttagtgagggttaatgct

>AllrRNASites___rRNALSUS58S_57_psi

gccgatgtacgtgttccgtaagacggagctcaagcactccaagaccgagctcaacttcaa

ggagtggcaaaaggcctttaccgatgtgatgggcatggacgagctgtacaagtaaactag

tagcagacagatgcgccgtggatgatcactcggctcgtgcgtcgatgaagaacgcagcgc

tagctgcgagaattaatgtgaattgcaggacacattgatagtgagtagccacccgatcca

atgcttaaggtcaggtcactgcttgacggagccatagttggcgcgccattcgatatcgga

ccctttccctttagtgagggttaatgct

>AllrRNASites___rRNALSUS58S_71_psi

gccgatgtacgtgttccgtaagacggagctcaagcactccaagaccgagctcaacttcaa

ggagtggcaaaaggcctttaccgatgtgatgggcatggacgagctgtacaagtaaactag

tagcagacagatgcgccgtggattgcgtcgatgaagaacgcagcgctagctgcgagaatt

aatgtgaattgcaggacacattgatcatcgacacttcgaaagtgttagccacccgatcca

atgcttaaggtcaggtcactgcttgacggagccatagttggcgcgccattcgatatcgga

ccctttccctttagtgagggttaatgct

>AllrRNASites___rRNASSU18S_34_psi

gccgatgtacgtgttccgtaagacggagctcaagcactccaagaccgagctcaacttcaa

ggagtggcaaaaggcctttaccgatgtgatgggcatggacgagctgtacaagtaaactag

tagcagacagatgcgccgtggatagttgccttacctggttgatcctgccagtagcatatg

cttgtctcaaagattaagccatgcatgtctaagtacgcaacttaatagccacccgatcca

atgcttaaggtcaggtcactgcttgacggagccatagttggcgcgccattcgatatcgga

ccctttccctttagtgagggttaatgct

>AllrRNASites___rRNASSU18S_36_psi

gccgatgtacgtgttccgtaagacggagctcaagcactccaagaccgagctcaacttcaa

ggagtggcaaaaggcctttaccgatgtgatgggcatggacgagctgtacaagtaaactag

tagcagacagatgcgccgtggatttgccttacctggttgatcctgccagtagcatatgct

tgtctcaaagattaagccatgcatgtctaagtacgcactgcggctgagccacccgatcca

atgcttaaggtcaggtcactgcttgacggagccatagttggcgcgccattcgatatcgga

ccctttccctttagtgagggttaatgct

>AllrRNASites___rRNASSU18S_93_psi

gccgatgtacgtgttccgtaagacggagctcaagcactccaagaccgagctcaacttcaa

ggagtggcaaaaggcctttaccgatgtgatgggcatggacgagctgtacaagtaaactag

tagcagacagatgcgccgtggatgcatgtctaagtacgcacggccggtacagtgaaactg

cgaatggctcattaaatcagttatggttcctttggtcgctaggtgaagccacccgatcca

atgcttaaggtcaggtcactgcttgacggagccatagttggcgcgccattcgatatcgga

ccctttccctttagtgagggttaatgct

>AllrRNASites___rRNASSU18S_105_psi

gccgatgtacgtgttccgtaagacggagctcaagcactccaagaccgagctcaacttcaa

ggagtggcaaaaggcctttaccgatgtgatgggcatggacgagctgtacaagtaaactag

tagcagacagatgcgccgtggatacgcacggccggtacagtgaaactgcgaatggctcat

taaatcagttatggttcctttggtcgctcgctcctctcagctatcgagccacccgatcca

atgcttaaggtcaggtcactgcttgacggagccatagttggcgcgccattcgatatcgga

ccctttccctttagtgagggttaatgct

>AllrRNASites___rRNASSU18S_109_psi

gccgatgtacgtgttccgtaagacggagctcaagcactccaagaccgagctcaacttcaa

ggagtggcaaaaggcctttaccgatgtgatgggcatggacgagctgtacaagtaaactag

tagcagacagatgcgccgtggatacggccggtacagtgaaactgcgaatggctcattaaa

tcagttatggttcctttggtcgctcgctcctctcctactgttgcaaagccacccgatcca

atgcttaaggtcaggtcactgcttgacggagccatagttggcgcgccattcgatatcgga

ccctttccctttagtgagggttaatgct

>AllrRNASites___rRNASSU18S_119_psi

gccgatgtacgtgttccgtaagacggagctcaagcactccaagaccgagctcaacttcaa

ggagtggcaaaaggcctttaccgatgtgatgggcatggacgagctgtacaagtaaactag

tagcagacagatgcgccgtggatcagtgaaactgcgaatggctcattaaatcagttatgg

ttcctttggtcgctcgctcctctcctacttggataacttcatggaaagccacccgatcca

atgcttaaggtcaggtcactgcttgacggagccatagttggcgcgccattcgatatcgga

ccctttccctttagtgagggttaatgct

>AllrRNASites___rRNASSU18S_210_psi

gccgatgtacgtgttccgtaagacggagctcaagcactccaagaccgagctcaacttcaa

ggagtggcaaaaggcctttaccgatgtgatgggcatggacgagctgtacaagtaaactag

tagcagacagatgcgccgtggattaatacatgccgacgggcgctgacccccttcgcgggg

gggatgcgtgcatttatcagatcaaaaccaacccggtctttctgcaagccacccgatcca

atgcttaaggtcaggtcactgcttgacggagccatagttggcgcgccattcgatatcgga

ccctttccctttagtgagggttaatgct

>AllrRNASites___rRNASSU18S_218_psi

gccgatgtacgtgttccgtaagacggagctcaagcactccaagaccgagctcaacttcaa

ggagtggcaaaaggcctttaccgatgtgatgggcatggacgagctgtacaagtaaactag

tagcagacagatgcgccgtggatgccgacgggcgctgacccccttcgcgggggggatgcg

tgcatttatcagatcaaaaccaacccggtcagcccctccgttgactagccacccgatcca

atgcttaaggtcaggtcactgcttgacggagccatagttggcgcgccattcgatatcgga

ccctttccctttagtgagggttaatgct

>AllrRNASites___rRNASSU18S_406_psi

gccgatgtacgtgttccgtaagacggagctcaagcactccaagaccgagctcaacttcaa

ggagtggcaaaaggcctttaccgatgtgatgggcatggacgagctgtacaagtaaactag

tagcagacagatgcgccgtggatctttcgatggtagtcgccgtgcctaccatggtgacca

cgggtgacggggaatcagggttcgattccggagagggaattcacaaagccacccgatcca

atgcttaaggtcaggtcactgcttgacggagccatagttggcgcgccattcgatatcgga

ccctttccctttagtgagggttaatgct

>AllrRNASites___rRNASSU18S_572_psi

gccgatgtacgtgttccgtaagacggagctcaagcactccaagaccgagctcaacttcaa

ggagtggcaaaaggcctttaccgatgtgatgggcatggacgagctgtacaagtaaactag

tagcagacagatgcgccgtggatacaggactctttcgaggccctgtaattggaatgagtc

cactttaaatcctttaacgaggatccattggagggcaagtaatgttagccacccgatcca

atgcttaaggtcaggtcactgcttgacggagccatagttggcgcgccattcgatatcgga

ccctttccctttagtgagggttaatgct

>AllrRNASites___rRNASSU18S_609_psi

gccgatgtacgtgttccgtaagacggagctcaagcactccaagaccgagctcaacttcaa

ggagtggcaaaaggcctttaccgatgtgatgggcatggacgagctgtacaagtaaactag

tagcagacagatgcgccgtggatcactttaaatcctttaacgaggatccattggagggca

agtctggtgccagcagccgcggtaattccagctccaatgtcttgatagccacccgatcca

atgcttaaggtcaggtcactgcttgacggagccatagttggcgcgccattcgatatcgga

ccctttccctttagtgagggttaatgct

>AllrRNASites___rRNASSU18S_649_psi

gccgatgtacgtgttccgtaagacggagctcaagcactccaagaccgagctcaacttcaa

ggagtggcaaaaggcctttaccgatgtgatgggcatggacgagctgtacaagtaaactag

tagcagacagatgcgccgtggatctggtgccagcagccgcggtaattccagctccaatag

cgtatattaaagttgctgcagttaaaaagctcgtagttctcgcgaaagccacccgatcca

atgcttaaggtcaggtcactgcttgacggagccatagttggcgcgccattcgatatcgga

ccctttccctttagtgagggttaatgct

>AllrRNASites___rRNASSU18S_651_psi

gccgatgtacgtgttccgtaagacggagctcaagcactccaagaccgagctcaacttcaa

ggagtggcaaaaggcctttaccgatgtgatgggcatggacgagctgtacaagtaaactag

tagcagacagatgcgccgtggatggtgccagcagccgcggtaattccagctccaatagcg

tatattaaagttgctgcagttaaaaagctcgtagttggtagtcgaaagccacccgatcca

atgcttaaggtcaggtcactgcttgacggagccatagttggcgcgccattcgatatcgga

ccctttccctttagtgagggttaatgct

>AllrRNASites___rRNASSU18S_681_psi

gccgatgtacgtgttccgtaagacggagctcaagcactccaagaccgagctcaacttcaa

ggagtggcaaaaggcctttaccgatgtgatgggcatggacgagctgtacaagtaaactag

tagcagacagatgcgccgtggataatagcgtatattaaagttgctgcagttaaaaagctc

gtagttggatcttgggagcgggcgggcggtccgccgcgacaccgccagccacccgatcca

atgcttaaggtcaggtcactgcttgacggagccatagttggcgcgccattcgatatcgga

ccctttccctttagtgagggttaatgct

>AllrRNASites___rRNASSU18S_686_psi

gccgatgtacgtgttccgtaagacggagctcaagcactccaagaccgagctcaacttcaa

ggagtggcaaaaggcctttaccgatgtgatgggcatggacgagctgtacaagtaaactag

tagcagacagatgcgccgtggatcgtatattaaagttgctgcagttaaaaagctcgtagt

tggatcttgggagcgggcgggcggtccgccgcgaggcgcagtcccgagccacccgatcca

atgcttaaggtcaggtcactgcttgacggagccatagttggcgcgccattcgatatcgga

ccctttccctttagtgagggttaatgct

>AllrRNASites___rRNASSU18S_801_psi

gccgatgtacgtgttccgtaagacggagctcaagcactccaagaccgagctcaacttcaa

ggagtggcaaaaggcctttaccgatgtgatgggcatggacgagctgtacaagtaaactag

tagcagacagatgcgccgtggattcgatgctcttagctgagtgtcccgcggggcccgaag

cgtttactttgaaaaaattagagtgttcaaagcaggccaatctcaaagccacccgatcca

atgcttaaggtcaggtcactgcttgacggagccatagttggcgcgccattcgatatcgga

ccctttccctttagtgagggttaatgct

>AllrRNASites___rRNASSU18S_814_psi

gccgatgtacgtgttccgtaagacggagctcaagcactccaagaccgagctcaacttcaa

ggagtggcaaaaggcctttaccgatgtgatgggcatggacgagctgtacaagtaaactag

tagcagacagatgcgccgtggatctgagtgtcccgcggggcccgaagcgtttactttgaa

aaaattagagtgttcaaagcaggcccgagccgcctggattatagagagccacccgatcca

atgcttaaggtcaggtcactgcttgacggagccatagttggcgcgccattcgatatcgga

ccctttccctttagtgagggttaatgct

>AllrRNASites___rRNASSU18S_815_psi

gccgatgtacgtgttccgtaagacggagctcaagcactccaagaccgagctcaacttcaa

ggagtggcaaaaggcctttaccgatgtgatgggcatggacgagctgtacaagtaaactag

tagcagacagatgcgccgtggattgagtgtcccgcggggcccgaagcgtttactttgaaa

aaattagagtgttcaaagcaggcccgagccgcctggatagttttgcagccacccgatcca

atgcttaaggtcaggtcactgcttgacggagccatagttggcgcgccattcgatatcgga

ccctttccctttagtgagggttaatgct

>AllrRNASites___rRNASSU18S_822_psi

gccgatgtacgtgttccgtaagacggagctcaagcactccaagaccgagctcaacttcaa

ggagtggcaaaaggcctttaccgatgtgatgggcatggacgagctgtacaagtaaactag

tagcagacagatgcgccgtggatcccgcggggcccgaagcgtttactttgaaaaaattag

agtgttcaaagcaggcccgagccgcctggataccgcagcgtcggaaagccacccgatcca

atgcttaaggtcaggtcactgcttgacggagccatagttggcgcgccattcgatatcgga

ccctttccctttagtgagggttaatgct

>AllrRNASites___rRNASSU18S_863_psi

gccgatgtacgtgttccgtaagacggagctcaagcactccaagaccgagctcaacttcaa

ggagtggcaaaaggcctttaccgatgtgatgggcatggacgagctgtacaagtaaactag

tagcagacagatgcgccgtggatttcaaagcaggcccgagccgcctggataccgcagcta

ggaataatggaataggaccgcggttctattttgttggtcacgttcaagccacccgatcca

atgcttaaggtcaggtcactgcttgacggagccatagttggcgcgccattcgatatcgga

ccctttccctttagtgagggttaatgct

>AllrRNASites___rRNASSU18S_866_psi

gccgatgtacgtgttccgtaagacggagctcaagcactccaagaccgagctcaacttcaa

ggagtggcaaaaggcctttaccgatgtgatgggcatggacgagctgtacaagtaaactag

tagcagacagatgcgccgtggataaagcaggcccgagccgcctggataccgcagctagga

ataatggaataggaccgcggttctattttgttggtttttatcagtaagccacccgatcca

atgcttaaggtcaggtcactgcttgacggagccatagttggcgcgccattcgatatcgga

ccctttccctttagtgagggttaatgct

>AllrRNASites___rRNASSU18S_918_psi

gccgatgtacgtgttccgtaagacggagctcaagcactccaagaccgagctcaacttcaa

ggagtggcaaaaggcctttaccgatgtgatgggcatggacgagctgtacaagtaaactag

tagcagacagatgcgccgtggatcgcggttctattttgttggttttcggaactgaggcca

tgattaagagggacggccgggggcattcgtattgcgcccatcgcttagccacccgatcca

atgcttaaggtcaggtcactgcttgacggagccatagttggcgcgccattcgatatcgga

ccctttccctttagtgagggttaatgct

>AllrRNASites___rRNASSU18S_966_psi

gccgatgtacgtgttccgtaagacggagctcaagcactccaagaccgagctcaacttcaa

ggagtggcaaaaggcctttaccgatgtgatgggcatggacgagctgtacaagtaaactag

tagcagacagatgcgccgtggatgacggccgggggcattcgtattgcgccgctagaggtg

aaattccttggaccggcgcaagacggaccagagcgaaaactgttgcagccacccgatcca

atgcttaaggtcaggtcactgcttgacggagccatagttggcgcgccattcgatatcgga

ccctttccctttagtgagggttaatgct

>AllrRNASites___rRNASSU18S_1005_psi

gccgatgtacgtgttccgtaagacggagctcaagcactccaagaccgagctcaacttcaa

ggagtggcaaaaggcctttaccgatgtgatgggcatggacgagctgtacaagtaaactag

tagcagacagatgcgccgtggatattccttggaccggcgcaagacggaccagagcgaaag

catttgccaagaatgttttcattaatcaagaacgaaagctgtgttcagccacccgatcca

atgcttaaggtcaggtcactgcttgacggagccatagttggcgcgccattcgatatcgga

ccctttccctttagtgagggttaatgct

>AllrRNASites___rRNASSU18S_1057_psi

gccgatgtacgtgttccgtaagacggagctcaagcactccaagaccgagctcaacttcaa

ggagtggcaaaaggcctttaccgatgtgatgggcatggacgagctgtacaagtaaactag

tagcagacagatgcgccgtggatttttcattaatcaagaacgaaagtcggaggttcgaag

acgatcagataccgtcgtagttccgaccataaacgatgcttaaataagccacccgatcca

atgcttaaggtcaggtcactgcttgacggagccatagttggcgcgccattcgatatcgga

ccctttccctttagtgagggttaatgct

>AllrRNASites___rRNASSU18S_1082_psi

gccgatgtacgtgttccgtaagacggagctcaagcactccaagaccgagctcaacttcaa

ggagtggcaaaaggcctttaccgatgtgatgggcatggacgagctgtacaagtaaactag

tagcagacagatgcgccgtggatggaggttcgaagacgatcagataccgtcgtagttccg

accataaacgatgccgaccggcgatgcggcggcgttataaatacaaagccacccgatcca

atgcttaaggtcaggtcactgcttgacggagccatagttggcgcgccattcgatatcgga

ccctttccctttagtgagggttaatgct

>AllrRNASites___rRNASSU18S_1175_psi

gccgatgtacgtgttccgtaagacggagctcaagcactccaagaccgagctcaacttcaa

ggagtggcaaaaggcctttaccgatgtgatgggcatggacgagctgtacaagtaaactag

tagcagacagatgcgccgtggatagcttccgggaaaccaaagtctttgggttccgggggg

agtatggttgcaaagctgaaacttaaaggaattgacggtgactatgagccacccgatcca

atgcttaaggtcaggtcactgcttgacggagccatagttggcgcgccattcgatatcgga

ccctttccctttagtgagggttaatgct

>AllrRNASites___rRNASSU18S_1239_psi

gccgatgtacgtgttccgtaagacggagctcaagcactccaagaccgagctcaacttcaa

ggagtggcaaaaggcctttaccgatgtgatgggcatggacgagctgtacaagtaaactag

tagcagacagatgcgccgtggatggaattgacggaagggcaccaccaggagtggagcctg

cggcttaatttgactcaacacgggaaacctcacccggcaacaccgtagccacccgatcca

atgcttaaggtcaggtcactgcttgacggagccatagttggcgcgccattcgatatcgga

ccctttccctttagtgagggttaatgct

>AllrRNASites___rRNASSU18S_1245_psi

gccgatgtacgtgttccgtaagacggagctcaagcactccaagaccgagctcaacttcaa

ggagtggcaaaaggcctttaccgatgtgatgggcatggacgagctgtacaagtaaactag

tagcagacagatgcgccgtggatgacggaagggcaccaccaggagtggagcctgcggctt

aatttgactcaacacgggaaacctcacccggcccggactaagcgaaagccacccgatcca

atgcttaaggtcaggtcactgcttgacggagccatagttggcgcgccattcgatatcgga

ccctttccctttagtgagggttaatgct

>AllrRNASites___rRNASSU18S_1348_psi

gccgatgtacgtgttccgtaagacggagctcaagcactccaagaccgagctcaacttcaa

ggagtggcaaaaggcctttaccgatgtgatgggcatggacgagctgtacaagtaaactag

tagcagacagatgcgccgtggattttctcgattccgtgggtggtggtgcatggccgttct

tagttggtggagcgatttgtctggttaattccgataacgattgataagccacccgatcca

atgcttaaggtcaggtcactgcttgacggagccatagttggcgcgccattcgatatcgga

ccctttccctttagtgagggttaatgct

>AllrRNASites___rRNASSU18S_1368_psi

gccgatgtacgtgttccgtaagacggagctcaagcactccaagaccgagctcaacttcaa

ggagtggcaaaaggcctttaccgatgtgatgggcatggacgagctgtacaagtaaactag

tagcagacagatgcgccgtggattggtgcatggccgttcttagttggtggagcgatttgt

ctggttaattccgataacgaacgagactctggcatgctatgtacaaagccacccgatcca

atgcttaaggtcaggtcactgcttgacggagccatagttggcgcgccattcgatatcgga

ccctttccctttagtgagggttaatgct

>AllrRNASites___rRNASSU18S_1446_psi

gccgatgtacgtgttccgtaagacggagctcaagcactccaagaccgagctcaacttcaa

ggagtggcaaaaggcctttaccgatgtgatgggcatggacgagctgtacaagtaaactag

tagcagacagatgcgccgtggattagttacgcgacccccgagcggtcggcgtcccccaac

ttcttagagggacaagtggcgttcagccacccgagattaccgaaaaagccacccgatcca

atgcttaaggtcaggtcactgcttgacggagccatagttggcgcgccattcgatatcgga

ccctttccctttagtgagggttaatgct

>AllrRNASites___rRNASSU18S_1626_psi

gccgatgtacgtgttccgtaagacggagctcaagcactccaagaccgagctcaacttcaa

ggagtggcaaaaggcctttaccgatgtgatgggcatggacgagctgtacaagtaaactag

tagcagacagatgcgccgtggatgttgaaccccattcgtgatggggatcggggattgcaa

ttattccccatgaacgaggaattcccagtaagtgcgggtccggcacagccacccgatcca

atgcttaaggtcaggtcactgcttgacggagccatagttggcgcgccattcgatatcgga

ccctttccctttagtgagggttaatgct

>AllrRNASites___rRNASSU18S_1644_psi

gccgatgtacgtgttccgtaagacggagctcaagcactccaagaccgagctcaacttcaa

ggagtggcaaaaggcctttaccgatgtgatgggcatggacgagctgtacaagtaaactag

tagcagacagatgcgccgtggattggggatcggggattgcaattattccccatgaacgag

gaattcccagtaagtgcgggtcataagcttgcgttgatgtactgggagccacccgatcca

atgcttaaggtcaggtcactgcttgacggagccatagttggcgcgccattcgatatcgga

ccctttccctttagtgagggttaatgct

>AllrRNASites___rRNASSU18S_1693_psi

gccgatgtacgtgttccgtaagacggagctcaagcactccaagaccgagctcaacttcaa

ggagtggcaaaaggcctttaccgatgtgatgggcatggacgagctgtacaagtaaactag

tagcagacagatgcgccgtggatagtgcgggtcataagcttgcgttgattaagtccctgc

cctttgtacacaccgcccgtcgctactaccgattggatactctagaagccacccgatcca

atgcttaaggtcaggtcactgcttgacggagccatagttggcgcgccattcgatatcgga

ccctttccctttagtgagggttaatgct

>AllrRNASites___snRNAU1_5_psi

gccgatgtacgtgttccgtaagacggagctcaagcactccaagaccgagctcaacttcaa

ggagtggcaaaaggcctttaccgatgtgatgggcatggacgagctgtacaagtaaactag

tagcagacagatgcgccgtggatacggacatctaccacgaaattgttatagaagttgcct

atacttacctggcaggggagataccatgatcacgaaggataagtgaagccacccgatcca

atgcttaaggtcaggtcactgcttgacggagccatagttggcgcgccattcgatatcgga

ccctttccctttagtgagggttaatgct

>AllrRNASites___snRNAU1_6_psi

gccgatgtacgtgttccgtaagacggagctcaagcactccaagaccgagctcaacttcaa

ggagtggcaaaaggcctttaccgatgtgatgggcatggacgagctgtacaagtaaactag

tagcagacagatgcgccgtggatcggacatctaccacgaaattgttatagaagttgccta

tacttacctggcaggggagataccatgatcacgaaggttgggaagcagccacccgatcca

atgcttaaggtcaggtcactgcttgacggagccatagttggcgcgccattcgatatcgga

ccctttccctttagtgagggttaatgct

>AllrRNASites___snRNAU12_18_psi

gccgatgtacgtgttccgtaagacggagctcaagcactccaagaccgagctcaacttcaa

ggagtggcaaaaggcctttaccgatgtgatgggcatggacgagctgtacaagtaaactag

tagcagacagatgcgccgtggatacgaaattgttatagaagttgccttgccttaaactta

tgagtaaggaaaataacgattcggggtgacgcccgaatttaaacttagccacccgatcca

atgcttaaggtcaggtcactgcttgacggagccatagttggcgcgccattcgatatcgga

ccctttccctttagtgagggttaatgct

>AllrRNASites___snRNAU12_27_psi

gccgatgtacgtgttccgtaagacggagctcaagcactccaagaccgagctcaacttcaa

ggagtggcaaaaggcctttaccgatgtgatgggcatggacgagctgtacaagtaaactag

tagcagacagatgcgccgtggatttatagaagttgccttgccttaaacttatgagtaagg

aaaataacgattcggggtgacgcccgaatcctcactgcctaggaacagccacccgatcca

atgcttaaggtcaggtcactgcttgacggagccatagttggcgcgccattcgatatcgga

ccctttccctttagtgagggttaatgct

>AllrRNASites___snRNAU4_4_psi

gccgatgtacgtgttccgtaagacggagctcaagcactccaagaccgagctcaacttcaa

ggagtggcaaaaggcctttaccgatgtgatgggcatggacgagctgtacaagtaaactag

tagcagacagatgcgccgtggattacggacatctaccacgaaattgttatagaagttgcc

tagctttgcgcagtggcagtatcgtagccaatgaggtcacgacccaagccacccgatcca

atgcttaaggtcaggtcactgcttgacggagccatagttggcgcgccattcgatatcgga

ccctttccctttagtgagggttaatgct

>AllrRNASites___snRNAU4_72_psi

gccgatgtacgtgttccgtaagacggagctcaagcactccaagaccgagctcaacttcaa

ggagtggcaaaaggcctttaccgatgtgatgggcatggacgagctgtacaagtaaactag

tagcagacagatgcgccgtggattgaggtctatccgaggcgcgattattgctaattgaaa

acttttcccaataccccgccgtgacgacttgcaatatatgggcattagccacccgatcca

atgcttaaggtcaggtcactgcttgacggagccatagttggcgcgccattcgatatcgga

ccctttccctttagtgagggttaatgct

>AllrRNASites___snRNAU4_79_psi

gccgatgtacgtgttccgtaagacggagctcaagcactccaagaccgagctcaacttcaa

ggagtggcaaaaggcctttaccgatgtgatgggcatggacgagctgtacaagtaaactag

tagcagacagatgcgccgtggattatccgaggcgcgattattgctaattgaaaacttttc

ccaataccccgccgtgacgacttgcaatatagtcggcacaggctacagccacccgatcca

atgcttaaggtcaggtcactgcttgacggagccatagttggcgcgccattcgatatcgga

ccctttccctttagtgagggttaatgct

>AllrRNASites___snRNAU4atac_12_psi

gccgatgtacgtgttccgtaagacggagctcaagcactccaagaccgagctcaacttcaa

ggagtggcaaaaggcctttaccgatgtgatgggcatggacgagctgtacaagtaaactag

tagcagacagatgcgccgtggattctaccacgaaattgttatagaagttgcctaaccatc

cttttcttggggttgcgctactgtccaatgagcgcataggacgatgagccacccgatcca

atgcttaaggtcaggtcactgcttgacggagccatagttggcgcgccattcgatatcgga

ccctttccctttagtgagggttaatgct

>AllrRNASites___snRNAU5_43_psi

gccgatgtacgtgttccgtaagacggagctcaagcactccaagaccgagctcaacttcaa

ggagtggcaaaaggcctttaccgatgtgatgggcatggacgagctgtacaagtaaactag

tagcagacagatgcgccgtggattactctggtttctcttcagatcgcataaatctttcgc

cttttactaaagatttccgtggagaggaacaactctgaagtaaaccagccacccgatcca

atgcttaaggtcaggtcactgcttgacggagccatagttggcgcgccattcgatatcgga

ccctttccctttagtgagggttaatgct

>AllrRNASites___snRNAU5_46_psi

gccgatgtacgtgttccgtaagacggagctcaagcactccaagaccgagctcaacttcaa

ggagtggcaaaaggcctttaccgatgtgatgggcatggacgagctgtacaagtaaactag

tagcagacagatgcgccgtggattctggtttctcttcagatcgcataaatctttcgcctt

ttactaaagatttccgtggagaggaacaactctgagtccatcgaatagccacccgatcca

atgcttaaggtcaggtcactgcttgacggagccatagttggcgcgccattcgatatcgga

ccctttccctttagtgagggttaatgct

>AllrRNASites___snRNAU5_53_psi

gccgatgtacgtgttccgtaagacggagctcaagcactccaagaccgagctcaacttcaa

ggagtggcaaaaggcctttaccgatgtgatgggcatggacgagctgtacaagtaaactag

tagcagacagatgcgccgtggattctcttcagatcgcataaatctttcgccttttactaa

agatttccgtggagaggaacaactctgagtcttaaccctcgctattagccacccgatcca

atgcttaaggtcaggtcactgcttgacggagccatagttggcgcgccattcgatatcgga

ccctttccctttagtgagggttaatgct

>AllrRNASites___snRNAU6_31_psi

gccgatgtacgtgttccgtaagacggagctcaagcactccaagaccgagctcaacttcaa

ggagtggcaaaaggcctttaccgatgtgatgggcatggacgagctgtacaagtaaactag

tagcagacagatgcgccgtggatagaagttgcctgtgctcgcttcggcagcacatatact

aaaattggaacgatacagagaagatttagcatggccccacgaagggagccacccgatcca

atgcttaaggtcaggtcactgcttgacggagccatagttggcgcgccattcgatatcgga

ccctttccctttagtgagggttaatgct

>AllrRNASites___snRNAU6_40_psi

gccgatgtacgtgttccgtaagacggagctcaagcactccaagaccgagctcaacttcaa

ggagtggcaaaaggcctttaccgatgtgatgggcatggacgagctgtacaagtaaactag

tagcagacagatgcgccgtggatctgtgctcgcttcggcagcacatatactaaaattgga

acgatacagagaagatttagcatggcccctgcgcaagggaatcttaagccacccgatcca

atgcttaaggtcaggtcactgcttgacggagccatagttggcgcgccattcgatatcgga

ccctttccctttagtgagggttaatgct

>AllrRNASites___snRNAU6_87_psi

gccgatgtacgtgttccgtaagacggagctcaagcactccaagaccgagctcaacttcaa

ggagtggcaaaaggcctttaccgatgtgatgggcatggacgagctgtacaagtaaactag

tagcagacagatgcgccgtggatgaagatttagcatggcccctgcgcaaggatgacacgc

aaattcgtgaagcgttccatatttttgattatttctggctatacgcagccacccgatcca

atgcttaaggtcaggtcactgcttgacggagccatagttggcgcgccattcgatatcgga

ccctttccctttagtgagggttaatgct

>AllrRNASites___snRNAU6atac_83_psi

gccgatgtacgtgttccgtaagacggagctcaagcactccaagaccgagctcaacttcaa

ggagtggcaaaaggcctttaccgatgtgatgggcatggacgagctgtacaagtaaactag

tagcagacagatgcgccgtggataggatggaagaggccctcgggcctgacaacacgcata

cggttaaggcattgccacctacttcgtggcatctaaccccctaaccagccacccgatcca

atgcttaaggtcaggtcactgcttgacggagccatagttggcgcgccattcgatatcgga

ccctttccctttagtgagggttaatgct

>AllrRNASites_OnePointMut___rRNALSUL28S_1515_psi

gccgatgtacgtgttccgtaagacggagctcaagcactccaagaccgagctcaacttcaa

ggagtggcaaaaggcctttaccgatgtgatgggcatggacgagctgtacaagtaaactag

tagcagacagatgcgccgtggatggagcacgagcgcacgtgttaggacccgaaagatggt

gaacgatgcctgggcagggcgaagccagaggaaactctccacgtaaagccacccgatcca

atgcttaaggtcaggtcactgcttgacggagccatagttggcgcgccattcgatatcgga

ccctttccctttagtgagggttaatgct

>AllrRNASites_OnePointMut___rRNALSUL28S_1561_psi

gccgatgtacgtgttccgtaagacggagctcaagcactccaagaccgagctcaacttcaa

ggagtggcaaaaggcctttaccgatgtgatgggcatggacgagctgtacaagtaaactag

tagcagacagatgcgccgtggatctgggcagggcgaagccagaggaaactctggtggagg

tccggagcggtcctgacgtgcaaatcggtcgtccgaccgggtccctagccacccgatcca

atgcttaaggtcaggtcactgcttgacggagccatagttggcgcgccattcgatatcgga

ccctttccctttagtgagggttaatgct

>AllrRNASites_OnePointMut___rRNALSUL28S_1656_psi

gccgatgtacgtgttccgtaagacggagctcaagcactccaagaccgagctcaacttcaa

ggagtggcaaaaggcctttaccgatgtgatgggcatggacgagctgtacaagtaaactag

tagcagacagatgcgccgtggattaatcgaaccatctagtagctggttccctccgaagtt

tcccgcaggatagctggcgctctcgcagacccgacgcacttctccaagccacccgatcca

atgcttaaggtcaggtcactgcttgacggagccatagttggcgcgccattcgatatcgga

ccctttccctttagtgagggttaatgct

>AllrRNASites_OnePointMut___rRNALSUL28S_1662_psi

gccgatgtacgtgttccgtaagacggagctcaagcactccaagaccgagctcaacttcaa

ggagtggcaaaaggcctttaccgatgtgatgggcatggacgagctgtacaagtaaactag

tagcagacagatgcgccgtggataaccatctagtagctggttccctccgaagtttccctc

aggagagctggcgctctcgcagacccgacgcacccccggcgggggtagccacccgatcca

atgcttaaggtcaggtcactgcttgacggagccatagttggcgcgccattcgatatcgga

ccctttccctttagtgagggttaatgct

>AllrRNASites_OnePointMut___rRNALSUL28S_1723_psi

gccgatgtacgtgttccgtaagacggagctcaagcactccaagaccgagctcaacttcaa

ggagtggcaaaaggcctttaccgatgtgatgggcatggacgagctgtacaagtaaactag

tagcagacagatgcgccgtggatccgacgcacccccgccacgcagttttatccggtaaag

cgaaggattagaggtcttggggccgaaacgatctcaacataatctgagccacccgatcca

atgcttaaggtcaggtcactgcttgacggagccatagttggcgcgccattcgatatcgga

ccctttccctttagtgagggttaatgct

>AllrRNASites_OnePointMut___rRNALSUL28S_1758_psi

gccgatgtacgtgttccgtaagacggagctcaagcactccaagaccgagctcaacttcaa

ggagtggcaaaaggcctttaccgatgtgatgggcatggacgagctgtacaagtaaactag

tagcagacagatgcgccgtggatagcgaatgattagaggtcttggggccgaaacgatctc

aaccgattctcaaactttaaatgggtaagaagcccggcatgtcaccagccacccgatcca

atgcttaaggtcaggtcactgcttgacggagccatagttggcgcgccattcgatatcgga

ccctttccctttagtgagggttaatgct

>AllrRNASites_OnePointMut___rRNALSUL28S_1761_psi

gccgatgtacgtgttccgtaagacggagctcaagcactccaagaccgagctcaacttcaa

ggagtggcaaaaggcctttaccgatgtgatgggcatggacgagctgtacaagtaaactag

tagcagacagatgcgccgtggatgaatgattagaggtcttggggccgaaacgatctcaac

ctatgctcaaactttaaatgggtaagaagcccggctcgattgacttagccacccgatcca

atgcttaaggtcaggtcactgcttgacggagccatagttggcgcgccattcgatatcgga

ccctttccctttagtgagggttaatgct

>AllrRNASites_OnePointMut___rRNALSUL28S_1771_psi

gccgatgtacgtgttccgtaagacggagctcaagcactccaagaccgagctcaacttcaa

ggagtggcaaaaggcctttaccgatgtgatgggcatggacgagctgtacaagtaaactag

tagcagacagatgcgccgtggataggtcttggggccgaaacgatctcaacctattctcaa

acttgaaatgggtaagaagcccggctcgctggcgtggacggtacagagccacccgatcca

atgcttaaggtcaggtcactgcttgacggagccatagttggcgcgccattcgatatcgga

ccctttccctttagtgagggttaatgct

>AllrRNASites_OnePointMut___rRNALSUL28S_1838_psi

gccgatgtacgtgttccgtaagacggagctcaagcactccaagaccgagctcaacttcaa

ggagtggcaaaaggcctttaccgatgtgatgggcatggacgagctgtacaagtaaactag

tagcagacagatgcgccgtggatggcgtggagccggggtggaatgcgagtgcctagtggg

ccacgtttggtaagcagaactggcgctgcgggatgaaccgtagtggagccacccgatcca

atgcttaaggtcaggtcactgcttgacggagccatagttggcgcgccattcgatatcgga

ccctttccctttagtgagggttaatgct

>AllrRNASites_OnePointMut___rRNALSUL28S_1840_psi

gccgatgtacgtgttccgtaagacggagctcaagcactccaagaccgagctcaacttcaa

ggagtggcaaaaggcctttaccgatgtgatgggcatggacgagctgtacaagtaaactag

tagcagacagatgcgccgtggatcgtggagccggggtggaatgcgagtgcctagtgggcc

acttgtggtaagcagaactggcgctgcgggatgaaccggtgacattagccacccgatcca

atgcttaaggtcaggtcactgcttgacggagccatagttggcgcgccattcgatatcgga

ccctttccctttagtgagggttaatgct

>AllrRNASites_OnePointMut___rRNALSUL28S_2485_psi

gccgatgtacgtgttccgtaagacggagctcaagcactccaagaccgagctcaacttcaa

ggagtggcaaaaggcctttaccgatgtgatgggcatggacgagctgtacaagtaaactag

tagcagacagatgcgccgtggatttccgaagggacgggcgatggcctccgttgccctcgg

ccgagcgaaagggagtcgggttcagatccccgaatccgctgatttaagccacccgatcca

atgcttaaggtcaggtcactgcttgacggagccatagttggcgcgccattcgatatcgga

ccctttccctttagtgagggttaatgct

>AllrRNASites_OnePointMut___rRNALSUL28S_3606_psi

gccgatgtacgtgttccgtaagacggagctcaagcactccaagaccgagctcaacttcaa

ggagtggcaaaaggcctttaccgatgtgatgggcatggacgagctgtacaagtaaactag

tagcagacagatgcgccgtggatagcagccgacttagaactggtgcggaccaggggaatc

cgacggtttaattaaaacaaagcatcgcgaaggcccgcagctttgtagccacccgatcca

atgcttaaggtcaggtcactgcttgacggagccatagttggcgcgccattcgatatcgga

ccctttccctttagtgagggttaatgct

>AllrRNASites_OnePointMut___rRNALSUL28S_3608_psi

gccgatgtacgtgttccgtaagacggagctcaagcactccaagaccgagctcaacttcaa

ggagtggcaaaaggcctttaccgatgtgatgggcatggacgagctgtacaagtaaactag

tagcagacagatgcgccgtggatcagccgacttagaactggtgcggaccaggggaatccg

actggttaattaaaacaaagcatcgcgaaggcccgcggttcggccgagccacccgatcca

atgcttaaggtcaggtcactgcttgacggagccatagttggcgcgccattcgatatcgga

ccctttccctttagtgagggttaatgct

>AllrRNASites_OnePointMut___rRNALSUL28S_3664_psi

gccgatgtacgtgttccgtaagacggagctcaagcactccaagaccgagctcaacttcaa

ggagtggcaaaaggcctttaccgatgtgatgggcatggacgagctgtacaagtaaactag

tagcagacagatgcgccgtggatgcatcgcgaaggcccgcggcgggtgttgacgcgatgt

gattgctgcccagtgctctgaatgtcaaagtgaagaaagacattccagccacccgatcca

atgcttaaggtcaggtcactgcttgacggagccatagttggcgcgccattcgatatcgga

ccctttccctttagtgagggttaatgct

>AllrRNASites_OnePointMut___rRNALSUL28S_3684_psi

gccgatgtacgtgttccgtaagacggagctcaagcactccaagaccgagctcaacttcaa

ggagtggcaaaaggcctttaccgatgtgatgggcatggacgagctgtacaagtaaactag

tagcagacagatgcgccgtggatgggtgttgacgcgatgtgatttctgcccagtgctctg

aatggcaaagtgaagaaattcaatgaagcgcgggtaaaggctgatgagccacccgatcca

atgcttaaggtcaggtcactgcttgacggagccatagttggcgcgccattcgatatcgga

ccctttccctttagtgagggttaatgct

>AllrRNASites_OnePointMut___rRNALSUL28S_3699_psi

gccgatgtacgtgttccgtaagacggagctcaagcactccaagaccgagctcaacttcaa

ggagtggcaaaaggcctttaccgatgtgatgggcatggacgagctgtacaagtaaactag

tagcagacagatgcgccgtggatgtgatttctgcccagtgctctgaatgtcaaagtgaag

aaatgcaatgaagcgcgggtaaacggcgggagtaactagagatctgagccacccgatcca

atgcttaaggtcaggtcactgcttgacggagccatagttggcgcgccattcgatatcgga

ccctttccctttagtgagggttaatgct

>AllrRNASites_OnePointMut___rRNALSUL28S_3703_psi

gccgatgtacgtgttccgtaagacggagctcaagcactccaagaccgagctcaacttcaa

ggagtggcaaaaggcctttaccgatgtgatgggcatggacgagctgtacaagtaaactag

tagcagacagatgcgccgtggattttctgcccagtgctctgaatgtcaaagtgaagaaat

tcaaggaagcgcgggtaaacggcgggagtaactatgacggatagagagccacccgatcca

atgcttaaggtcaggtcactgcttgacggagccatagttggcgcgccattcgatatcgga

ccctttccctttagtgagggttaatgct

>AllrRNASites_OnePointMut___rRNALSUL28S_3727_psi

gccgatgtacgtgttccgtaagacggagctcaagcactccaagaccgagctcaacttcaa

ggagtggcaaaaggcctttaccgatgtgatgggcatggacgagctgtacaagtaaactag

tagcagacagatgcgccgtggataaagtgaagaaattcaatgaagcgcgggtaaacggcg

ggaggaactatgactctcttaaggtagccaaatgcctctggatagaagccacccgatcca

atgcttaaggtcaggtcactgcttgacggagccatagttggcgcgccattcgatatcgga

ccctttccctttagtgagggttaatgct

>AllrRNASites_OnePointMut___rRNALSUL28S_3731_psi

gccgatgtacgtgttccgtaagacggagctcaagcactccaagaccgagctcaacttcaa

ggagtggcaaaaggcctttaccgatgtgatgggcatggacgagctgtacaagtaaactag

tagcagacagatgcgccgtggattgaagaaattcaatgaagcgcgggtaaacggcgggag

taacgatgactctcttaaggtagccaaatgcctcgtcaagttcaatagccacccgatcca

atgcttaaggtcaggtcactgcttgacggagccatagttggcgcgccattcgatatcgga

ccctttccctttagtgagggttaatgct

>AllrRNASites_OnePointMut___rRNALSUL28S_3733_psi

gccgatgtacgtgttccgtaagacggagctcaagcactccaagaccgagctcaacttcaa

ggagtggcaaaaggcctttaccgatgtgatgggcatggacgagctgtacaagtaaactag

tagcagacagatgcgccgtggataagaaattcaatgaagcgcgggtaaacggcgggagta

actaggactctcttaaggtagccaaatgcctcgtcatcgggagacaagccacccgatcca

atgcttaaggtcaggtcactgcttgacggagccatagttggcgcgccattcgatatcgga

ccctttccctttagtgagggttaatgct

>AllrRNASites_OnePointMut___rRNALSUL28S_3737_psi

gccgatgtacgtgttccgtaagacggagctcaagcactccaagaccgagctcaacttcaa

ggagtggcaaaaggcctttaccgatgtgatgggcatggacgagctgtacaagtaaactag

tagcagacagatgcgccgtggataattcaatgaagcgcgggtaaacggcgggagtaacta

tgacgctcttaaggtagccaaatgcctcgtcatctaatgtgcgattagccacccgatcca

atgcttaaggtcaggtcactgcttgacggagccatagttggcgcgccattcgatatcgga

ccctttccctttagtgagggttaatgct

>AllrRNASites_OnePointMut___rRNALSUL28S_3739_psi

gccgatgtacgtgttccgtaagacggagctcaagcactccaagaccgagctcaacttcaa

ggagtggcaaaaggcctttaccgatgtgatgggcatggacgagctgtacaagtaaactag

tagcagacagatgcgccgtggatttcaatgaagcgcgggtaaacggcgggagtaactatg

actcgcttaaggtagccaaatgcctcgtcatctaattatgcgatttagccacccgatcca

atgcttaaggtcaggtcactgcttgacggagccatagttggcgcgccattcgatatcgga

ccctttccctttagtgagggttaatgct

>AllrRNASites_OnePointMut___rRNALSUL28S_3791_psi

gccgatgtacgtgttccgtaagacggagctcaagcactccaagaccgagctcaacttcaa

ggagtggcaaaaggcctttaccgatgtgatgggcatggacgagctgtacaagtaaactag

tagcagacagatgcgccgtggatccaaatgcctcgtcatctaattagtgacgcgcatgaa

tggaggaacgagattcccactgtccctacctactatcctcgaatgaagccacccgatcca

atgcttaaggtcaggtcactgcttgacggagccatagttggcgcgccattcgatatcgga

ccctttccctttagtgagggttaatgct

>AllrRNASites_OnePointMut___rRNALSUL28S_3813_psi

gccgatgtacgtgttccgtaagacggagctcaagcactccaagaccgagctcaacttcaa

ggagtggcaaaaggcctttaccgatgtgatgggcatggacgagctgtacaagtaaactag

tagcagacagatgcgccgtggatagtgacgcgcatgaatggatgaacgagattcccactg

tcccgacctactatccagcgaaaccacagccaagggaaaattttccagccacccgatcca

atgcttaaggtcaggtcactgcttgacggagccatagttggcgcgccattcgatatcgga

ccctttccctttagtgagggttaatgct

>AllrRNASites_OnePointMut___rRNALSUL28S_3820_psi

gccgatgtacgtgttccgtaagacggagctcaagcactccaagaccgagctcaacttcaa

ggagtggcaaaaggcctttaccgatgtgatgggcatggacgagctgtacaagtaaactag

tagcagacagatgcgccgtggatcgcatgaatggatgaacgagattcccactgtccctac

ctacgatccagcgaaaccacagccaagggaacgggcttggttaagcagccacccgatcca

atgcttaaggtcaggtcactgcttgacggagccatagttggcgcgccattcgatatcgga

ccctttccctttagtgagggttaatgct

>AllrRNASites_OnePointMut___rRNALSUL28S_3822_psi

gccgatgtacgtgttccgtaagacggagctcaagcactccaagaccgagctcaacttcaa

ggagtggcaaaaggcctttaccgatgtgatgggcatggacgagctgtacaagtaaactag

tagcagacagatgcgccgtggatcatgaatggatgaacgagattcccactgtccctacct

actagccagcgaaaccacagccaagggaacgggcttggcgctgaatagccacccgatcca

atgcttaaggtcaggtcactgcttgacggagccatagttggcgcgccattcgatatcgga

ccctttccctttagtgagggttaatgct

>AllrRNASites_OnePointMut___rRNALSUL28S_3853_psi

gccgatgtacgtgttccgtaagacggagctcaagcactccaagaccgagctcaacttcaa

ggagtggcaaaaggcctttaccgatgtgatgggcatggacgagctgtacaagtaaactag

tagcagacagatgcgccgtggatctacctactatccagcgaaaccacagccaagggaacg

ggctgggcggaatcagcggggaaagaagaccctgttgataaagagcagccacccgatcca

atgcttaaggtcaggtcactgcttgacggagccatagttggcgcgccattcgatatcgga

ccctttccctttagtgagggttaatgct

>AllrRNASites_OnePointMut___rRNALSUL28S_3889_psi

gccgatgtacgtgttccgtaagacggagctcaagcactccaagaccgagctcaacttcaa

ggagtggcaaaaggcctttaccgatgtgatgggcatggacgagctgtacaagtaaactag

tagcagacagatgcgccgtggatgggcttggcggaatcagcggggaaagaagaccctgtt

gagcgtgactctagtctggcacggtgaagagacatgaggacgccatagccacccgatcca

atgcttaaggtcaggtcactgcttgacggagccatagttggcgcgccattcgatatcgga

ccctttccctttagtgagggttaatgct

>AllrRNASites_OnePointMut___rRNALSUL28S_3928_psi

gccgatgtacgtgttccgtaagacggagctcaagcactccaagaccgagctcaacttcaa

ggagtggcaaaaggcctttaccgatgtgatgggcatggacgagctgtacaagtaaactag

tagcagacagatgcgccgtggatgcttgactctagtctggcacggtgaagagacatgaga

ggtggagaataagtgggaggcccccggcgcccccccggtttaactaagccacccgatcca

atgcttaaggtcaggtcactgcttgacggagccatagttggcgcgccattcgatatcgga

ccctttccctttagtgagggttaatgct

>AllrRNASites_OnePointMut___rRNALSUL28S_4253_psi

gccgatgtacgtgttccgtaagacggagctcaagcactccaagaccgagctcaacttcaa

ggagtggcaaaaggcctttaccgatgtgatgggcatggacgagctgtacaagtaaactag

tagcagacagatgcgccgtggatcagaaacctcccgtggagcagaagggcaaaagctcgc

ttgagcttgattttcagtacgaatacagaccgtgaaagcgcactctagccacccgatcca

atgcttaaggtcaggtcactgcttgacggagccatagttggcgcgccattcgatatcgga

ccctttccctttagtgagggttaatgct

>AllrRNASites_OnePointMut___rRNALSUL28S_4256_psi

gccgatgtacgtgttccgtaagacggagctcaagcactccaagaccgagctcaacttcaa

ggagtggcaaaaggcctttaccgatgtgatgggcatggacgagctgtacaagtaaactag

tagcagacagatgcgccgtggataaacctcccgtggagcagaagggcaaaagctcgcttg

atctggattttcagtacgaatacagaccgtgaaagcggattacctaagccacccgatcca

atgcttaaggtcaggtcactgcttgacggagccatagttggcgcgccattcgatatcgga

ccctttccctttagtgagggttaatgct

>AllrRNASites_OnePointMut___rRNALSUL28S_4259_psi

gccgatgtacgtgttccgtaagacggagctcaagcactccaagaccgagctcaacttcaa

ggagtggcaaaaggcctttaccgatgtgatgggcatggacgagctgtacaagtaaactag

tagcagacagatgcgccgtggatcctcccgtggagcagaagggcaaaagctcgcttgatc

ttgagtttcagtacgaatacagaccgtgaaagcggggccctaggcaagccacccgatcca

atgcttaaggtcaggtcactgcttgacggagccatagttggcgcgccattcgatatcgga

ccctttccctttagtgagggttaatgct

>AllrRNASites_OnePointMut___rRNALSUL28S_4272_psi

gccgatgtacgtgttccgtaagacggagctcaagcactccaagaccgagctcaacttcaa

ggagtggcaaaaggcctttaccgatgtgatgggcatggacgagctgtacaagtaaactag

tagcagacagatgcgccgtggatagaagggcaaaagctcgcttgatcttgattttcagta

cgaagacagaccgtgaaagcggggcctcacgatccttcaaaggctaagccacccgatcca

atgcttaaggtcaggtcactgcttgacggagccatagttggcgcgccattcgatatcgga

ccctttccctttagtgagggttaatgct

>AllrRNASites_OnePointMut___rRNALSUL28S_4313_psi

gccgatgtacgtgttccgtaagacggagctcaagcactccaagaccgagctcaacttcaa

ggagtggcaaaaggcctttaccgatgtgatgggcatggacgagctgtacaagtaaactag

tagcagacagatgcgccgtggattacagaccgtgaaagcggggcctcacgatccttctga

ccttgtgggttttaagcaggaggtgtcagaaaagttacccatatcgagccacccgatcca

atgcttaaggtcaggtcactgcttgacggagccatagttggcgcgccattcgatatcgga

ccctttccctttagtgagggttaatgct

>AllrRNASites_OnePointMut___rRNALSUL28S_4321_psi

gccgatgtacgtgttccgtaagacggagctcaagcactccaagaccgagctcaacttcaa

ggagtggcaaaaggcctttaccgatgtgatgggcatggacgagctgtacaagtaaactag

tagcagacagatgcgccgtggatgtgaaagcggggcctcacgatccttctgaccttttgg

gtttgaagcaggaggtgtcagaaaagttaccacagggagttcgccaagccacccgatcca

atgcttaaggtcaggtcactgcttgacggagccatagttggcgcgccattcgatatcgga

ccctttccctttagtgagggttaatgct

>AllrRNASites_OnePointMut___rRNALSUL28S_4363_psi

gccgatgtacgtgttccgtaagacggagctcaagcactccaagaccgagctcaacttcaa

ggagtggcaaaaggcctttaccgatgtgatgggcatggacgagctgtacaagtaaactag

tagcagacagatgcgccgtggataagcaggaggtgtcagaaaagttaccacagggataac

tggcgtgtggcggccaagcgttcatagcgacgtcgcttgaaacctaagccacccgatcca

atgcttaaggtcaggtcactgcttgacggagccatagttggcgcgccattcgatatcgga

ccctttccctttagtgagggttaatgct

>AllrRNASites_OnePointMut___rRNALSUL28S_4380_psi

gccgatgtacgtgttccgtaagacggagctcaagcactccaagaccgagctcaacttcaa

ggagtggcaaaaggcctttaccgatgtgatgggcatggacgagctgtacaagtaaactag

tagcagacagatgcgccgtggataaagttaccacagggataactggcttgtggcggccaa

gcgtgcatagcgacgtcgctttttgatccttcgatgtcgtcaattaagccacccgatcca

atgcttaaggtcaggtcactgcttgacggagccatagttggcgcgccattcgatatcgga

ccctttccctttagtgagggttaatgct

>AllrRNASites_OnePointMut___rRNALSUL28S_4391_psi

gccgatgtacgtgttccgtaagacggagctcaagcactccaagaccgagctcaacttcaa

ggagtggcaaaaggcctttaccgatgtgatgggcatggacgagctgtacaagtaaactag

tagcagacagatgcgccgtggatagggataactggcttgtggcggccaagcgttcatagc

gacggcgctttttgatccttcgatgtcggctcttcctactacgggaagccacccgatcca

atgcttaaggtcaggtcactgcttgacggagccatagttggcgcgccattcgatatcgga

ccctttccctttagtgagggttaatgct

>AllrRNASites_OnePointMut___rRNALSUL28S_4402_psi

gccgatgtacgtgttccgtaagacggagctcaagcactccaagaccgagctcaacttcaa

ggagtggcaaaaggcctttaccgatgtgatgggcatggacgagctgtacaagtaaactag

tagcagacagatgcgccgtggatgcttgtggcggccaagcgttcatagcgacgtcgcttt

ttgagccttcgatgtcggctcttcctatcattgtgaagcgggtattagccacccgatcca

atgcttaaggtcaggtcactgcttgacggagccatagttggcgcgccattcgatatcgga

ccctttccctttagtgagggttaatgct

>AllrRNASites_OnePointMut___rRNALSUL28S_4417_psi

gccgatgtacgtgttccgtaagacggagctcaagcactccaagaccgagctcaacttcaa

ggagtggcaaaaggcctttaccgatgtgatgggcatggacgagctgtacaagtaaactag

tagcagacagatgcgccgtggatgcgttcatagcgacgtcgctttttgatccttcgatgt

cggcgcttcctatcattgtgaagcagaattcgccaagcgtaacccgagccacccgatcca

atgcttaaggtcaggtcactgcttgacggagccatagttggcgcgccattcgatatcgga

ccctttccctttagtgagggttaatgct

>AllrRNASites_OnePointMut___rRNALSUL28S_4431_psi

gccgatgtacgtgttccgtaagacggagctcaagcactccaagaccgagctcaacttcaa

ggagtggcaaaaggcctttaccgatgtgatgggcatggacgagctgtacaagtaaactag

tagcagacagatgcgccgtggatgtcgctttttgatccttcgatgtcggctcttcctatc

attgggaagcagaattcgccaagcgttggattgttcacgaggccgcagccacccgatcca

atgcttaaggtcaggtcactgcttgacggagccatagttggcgcgccattcgatatcgga

ccctttccctttagtgagggttaatgct

>AllrRNASites_OnePointMut___rRNALSUL28S_4460_psi

gccgatgtacgtgttccgtaagacggagctcaagcactccaagaccgagctcaacttcaa

ggagtggcaaaaggcctttaccgatgtgatgggcatggacgagctgtacaagtaaactag

tagcagacagatgcgccgtggatttcctatcattgtgaagcagaattcgccaagcgttgg

attggtcacccactaatagggaacgtgagctgggtttaaggttggaagccacccgatcca

atgcttaaggtcaggtcactgcttgacggagccatagttggcgcgccattcgatatcgga

ccctttccctttagtgagggttaatgct

>AllrRNASites_OnePointMut___rRNALSUL28S_4481_psi

gccgatgtacgtgttccgtaagacggagctcaagcactccaagaccgagctcaacttcaa

ggagtggcaaaaggcctttaccgatgtgatgggcatggacgagctgtacaagtaaactag

tagcagacagatgcgccgtggatattcgccaagcgttggattgttcacccactaataggg

aacgggagctgggtttagaccgtcgtgagacaggttagtattcgcaagccacccgatcca

atgcttaaggtcaggtcactgcttgacggagccatagttggcgcgccattcgatatcgga

ccctttccctttagtgagggttaatgct

>AllrRNASites_OnePointMut___rRNALSUL28S_4491_psi

gccgatgtacgtgttccgtaagacggagctcaagcactccaagaccgagctcaacttcaa

ggagtggcaaaaggcctttaccgatgtgatgggcatggacgagctgtacaagtaaactag

tagcagacagatgcgccgtggatcgttggattgttcacccactaatagggaacgtgagct

gggtgtagaccgtcgtgagacaggttagttttaccctagaatcccaagccacccgatcca

atgcttaaggtcaggtcactgcttgacggagccatagttggcgcgccattcgatatcgga

ccctttccctttagtgagggttaatgct

>AllrRNASites_OnePointMut___rRNALSUL28S_4512_psi

gccgatgtacgtgttccgtaagacggagctcaagcactccaagaccgagctcaacttcaa

ggagtggcaaaaggcctttaccgatgtgatgggcatggacgagctgtacaagtaaactag

tagcagacagatgcgccgtggatatagggaacgtgagctgggtttagaccgtcgtgagac

aggtgagttttaccctactgatgatgtgttgttgccatctcttaatagccacccgatcca

atgcttaaggtcaggtcactgcttgacggagccatagttggcgcgccattcgatatcgga

ccctttccctttagtgagggttaatgct

>AllrRNASites_OnePointMut___rRNALSUL28S_4529_psi

gccgatgtacgtgttccgtaagacggagctcaagcactccaagaccgagctcaacttcaa

ggagtggcaaaaggcctttaccgatgtgatgggcatggacgagctgtacaagtaaactag

tagcagacagatgcgccgtggatggtttagaccgtcgtgagacaggttagttttacccta

ctgaggatgtgttgttgccatggtaatcctgctcagtacggtatacagccacccgatcca

atgcttaaggtcaggtcactgcttgacggagccatagttggcgcgccattcgatatcgga

ccctttccctttagtgagggttaatgct

>AllrRNASites_OnePointMut___rRNALSUL28S_4536_psi

gccgatgtacgtgttccgtaagacggagctcaagcactccaagaccgagctcaacttcaa

ggagtggcaaaaggcctttaccgatgtgatgggcatggacgagctgtacaagtaaactag

tagcagacagatgcgccgtggataccgtcgtgagacaggttagttttaccctactgatga

tgtggtgttgccatggtaatcctgctcagtacgagaggtcagggcaagccacccgatcca

atgcttaaggtcaggtcactgcttgacggagccatagttggcgcgccattcgatatcgga

ccctttccctttagtgagggttaatgct

>AllrRNASites_OnePointMut___rRNALSUL28S_4539_psi

gccgatgtacgtgttccgtaagacggagctcaagcactccaagaccgagctcaacttcaa

ggagtggcaaaaggcctttaccgatgtgatgggcatggacgagctgtacaagtaaactag

tagcagacagatgcgccgtggatgtcgtgagacaggttagttttaccctactgatgatgt

gttggtgccatggtaatcctgctcagtacgagaggaacgtattacgagccacccgatcca

atgcttaaggtcaggtcactgcttgacggagccatagttggcgcgccattcgatatcgga

ccctttccctttagtgagggttaatgct

>AllrRNASites_OnePointMut___rRNALSUL28S_4588_psi

gccgatgtacgtgttccgtaagacggagctcaagcactccaagaccgagctcaacttcaa

ggagtggcaaaaggcctttaccgatgtgatgggcatggacgagctgtacaagtaaactag

tagcagacagatgcgccgtggatgtaatcctgctcagtacgagaggaaccgcaggttcag

acatgtggtgtatgtgcttggctgaggagccaatggggtcagagacagccacccgatcca

atgcttaaggtcaggtcactgcttgacggagccatagttggcgcgccattcgatatcgga

ccctttccctttagtgagggttaatgct

>AllrRNASites_OnePointMut___rRNALSUL28S_4596_psi

gccgatgtacgtgttccgtaagacggagctcaagcactccaagaccgagctcaacttcaa

ggagtggcaaaaggcctttaccgatgtgatgggcatggacgagctgtacaagtaaactag

tagcagacagatgcgccgtggatgctcagtacgagaggaaccgcaggttcagacatttgg

tgtaggtgcttggctgaggagccaatggggcgaagctaggtgctacagccacccgatcca

atgcttaaggtcaggtcactgcttgacggagccatagttggcgcgccattcgatatcgga

ccctttccctttagtgagggttaatgct

>AllrRNASites_OnePointMut___rRNALSUL28S_4633_psi

gccgatgtacgtgttccgtaagacggagctcaagcactccaagaccgagctcaacttcaa

ggagtggcaaaaggcctttaccgatgtgatgggcatggacgagctgtacaagtaaactag

tagcagacagatgcgccgtggattgtatgtgcttggctgaggagccaatggggcgaagct

accagctgtgggattatgactgaacgcctctaagtcagacagtttaagccacccgatcca

atgcttaaggtcaggtcactgcttgacggagccatagttggcgcgccattcgatatcgga

ccctttccctttagtgagggttaatgct

>AllrRNASites_OnePointMut___rRNALSUL28S_4649_psi

gccgatgtacgtgttccgtaagacggagctcaagcactccaagaccgagctcaacttcaa

ggagtggcaaaaggcctttaccgatgtgatgggcatggacgagctgtacaagtaaactag

tagcagacagatgcgccgtggataggagccaatggggcgaagctaccatctgtgggatta

tgacggaacgcctctaagtcagaatcccgcccaggcgactcgctcgagccacccgatcca

atgcttaaggtcaggtcactgcttgacggagccatagttggcgcgccattcgatatcgga

ccctttccctttagtgagggttaatgct

>AllrRNASites_OnePointMut___rRNALSUL28S_4927_psi

gccgatgtacgtgttccgtaagacggagctcaagcactccaagaccgagctcaacttcaa

ggagtggcaaaaggcctttaccgatgtgatgggcatggacgagctgtacaagtaaactag

tagcagacagatgcgccgtggatgtcacgcaccgcacgttcgtggggaacctggcgctaa

accagtcgtagacgacctgcttctgggtcggggtttcggcccaagtagccacccgatcca

atgcttaaggtcaggtcactgcttgacggagccatagttggcgcgccattcgatatcgga

ccctttccctttagtgagggttaatgct

>AllrRNASites_OnePointMut___rRNALSUL28S_4928_psi

gccgatgtacgtgttccgtaagacggagctcaagcactccaagaccgagctcaacttcaa

ggagtggcaaaaggcctttaccgatgtgatgggcatggacgagctgtacaagtaaactag

tagcagacagatgcgccgtggattcacgcaccgcacgttcgtggggaacctggcgctaaa

ccatgcgtagacgacctgcttctgggtcggggtttcgtcatacagaagccacccgatcca

atgcttaaggtcaggtcactgcttgacggagccatagttggcgcgccattcgatatcgga

ccctttccctttagtgagggttaatgct

>AllrRNASites_OnePointMut___rRNALSUL28S_4956_psi

gccgatgtacgtgttccgtaagacggagctcaagcactccaagaccgagctcaacttcaa

ggagtggcaaaaggcctttaccgatgtgatgggcatggacgagctgtacaagtaaactag

tagcagacagatgcgccgtggatggcgctaaaccattcgtagacgacctgcttctgggtc

gggggttcgtacgtagcagagcagctccctcgctgcgatatattgaagccacccgatcca

atgcttaaggtcaggtcactgcttgacggagccatagttggcgcgccattcgatatcgga

ccctttccctttagtgagggttaatgct

>AllrRNASites_OnePointMut___rRNALSUL28S_4965_psi

gccgatgtacgtgttccgtaagacggagctcaagcactccaagaccgagctcaacttcaa

ggagtggcaaaaggcctttaccgatgtgatgggcatggacgagctgtacaagtaaactag

tagcagacagatgcgccgtggatccattcgtagacgacctgcttctgggtcggggtttcg

tacggagcagagcagctccctcgctgcgatctattgaacccaaataagccacccgatcca

atgcttaaggtcaggtcactgcttgacggagccatagttggcgcgccattcgatatcgga

ccctttccctttagtgagggttaatgct

>AllrRNASites_OnePointMut___rRNALSUS58S_57_psi

gccgatgtacgtgttccgtaagacggagctcaagcactccaagaccgagctcaacttcaa

ggagtggcaaaaggcctttaccgatgtgatgggcatggacgagctgtacaagtaaactag

tagcagacagatgcgccgtggatgatcactcggctcgtgcgtcgatgaagaacgcagcgc

tagcggcgagaattaatgtgaattgcaggacacattgaaatttatgagccacccgatcca

atgcttaaggtcaggtcactgcttgacggagccatagttggcgcgccattcgatatcgga

ccctttccctttagtgagggttaatgct

>AllrRNASites_OnePointMut___rRNALSUS58S_71_psi

gccgatgtacgtgttccgtaagacggagctcaagcactccaagaccgagctcaacttcaa

ggagtggcaaaaggcctttaccgatgtgatgggcatggacgagctgtacaagtaaactag

tagcagacagatgcgccgtggattgcgtcgatgaagaacgcagcgctagctgcgagaatt

aatgggaattgcaggacacattgatcatcgacacttcgtacatggcagccacccgatcca

atgcttaaggtcaggtcactgcttgacggagccatagttggcgcgccattcgatatcgga

ccctttccctttagtgagggttaatgct

>AllrRNASites_OnePointMut___rRNASSU18S_34_psi

gccgatgtacgtgttccgtaagacggagctcaagcactccaagaccgagctcaacttcaa

ggagtggcaaaaggcctttaccgatgtgatgggcatggacgagctgtacaagtaaactag

tagcagacagatgcgccgtggatagttgccttacctggttgatcctgccagtagcatatg

cttggctcaaagattaagccatgcatgtctaagtacgcacccgtaaagccacccgatcca

atgcttaaggtcaggtcactgcttgacggagccatagttggcgcgccattcgatatcgga

ccctttccctttagtgagggttaatgct

>AllrRNASites_OnePointMut___rRNASSU18S_36_psi

gccgatgtacgtgttccgtaagacggagctcaagcactccaagaccgagctcaacttcaa

ggagtggcaaaaggcctttaccgatgtgatgggcatggacgagctgtacaagtaaactag

tagcagacagatgcgccgtggatttgccttacctggttgatcctgccagtagcatatgct

tgtcgcaaagattaagccatgcatgtctaagtacgcacctcatgggagccacccgatcca

atgcttaaggtcaggtcactgcttgacggagccatagttggcgcgccattcgatatcgga

ccctttccctttagtgagggttaatgct

>AllrRNASites_OnePointMut___rRNASSU18S_93_psi

gccgatgtacgtgttccgtaagacggagctcaagcactccaagaccgagctcaacttcaa

ggagtggcaaaaggcctttaccgatgtgatgggcatggacgagctgtacaagtaaactag

tagcagacagatgcgccgtggatgcatgtctaagtacgcacggccggtacagtgaaactg

cgaagggctcattaaatcagttatggttcctttggtcgacactctaagccacccgatcca

atgcttaaggtcaggtcactgcttgacggagccatagttggcgcgccattcgatatcgga

ccctttccctttagtgagggttaatgct

>AllrRNASites_OnePointMut___rRNASSU18S_105_psi

gccgatgtacgtgttccgtaagacggagctcaagcactccaagaccgagctcaacttcaa

ggagtggcaaaaggcctttaccgatgtgatgggcatggacgagctgtacaagtaaactag

tagcagacagatgcgccgtggatacgcacggccggtacagtgaaactgcgaatggctcat

taaagcagttatggttcctttggtcgctcgctcctctcgtgcgaaaagccacccgatcca

atgcttaaggtcaggtcactgcttgacggagccatagttggcgcgccattcgatatcgga

ccctttccctttagtgagggttaatgct

>AllrRNASites_OnePointMut___rRNASSU18S_109_psi

gccgatgtacgtgttccgtaagacggagctcaagcactccaagaccgagctcaacttcaa

ggagtggcaaaaggcctttaccgatgtgatgggcatggacgagctgtacaagtaaactag

tagcagacagatgcgccgtggatacggccggtacagtgaaactgcgaatggctcattaaa

tcaggtatggttcctttggtcgctcgctcctctcctacgtggcactagccacccgatcca

atgcttaaggtcaggtcactgcttgacggagccatagttggcgcgccattcgatatcgga

ccctttccctttagtgagggttaatgct

>AllrRNASites_OnePointMut___rRNASSU18S_119_psi

gccgatgtacgtgttccgtaagacggagctcaagcactccaagaccgagctcaacttcaa

ggagtggcaaaaggcctttaccgatgtgatgggcatggacgagctgtacaagtaaactag

tagcagacagatgcgccgtggatcagtgaaactgcgaatggctcattaaatcagttatgg

ttccgttggtcgctcgctcctctcctacttggataactcgctatgtagccacccgatcca

atgcttaaggtcaggtcactgcttgacggagccatagttggcgcgccattcgatatcgga

ccctttccctttagtgagggttaatgct

>AllrRNASites_OnePointMut___rRNASSU18S_210_psi

gccgatgtacgtgttccgtaagacggagctcaagcactccaagaccgagctcaacttcaa

ggagtggcaaaaggcctttaccgatgtgatgggcatggacgagctgtacaagtaaactag

tagcagacagatgcgccgtggattaatacatgccgacgggcgctgacccccttcgcgggg

gggaggcgtgcatttatcagatcaaaaccaacccggtccctgagtcagccacccgatcca

atgcttaaggtcaggtcactgcttgacggagccatagttggcgcgccattcgatatcgga

ccctttccctttagtgagggttaatgct

>AllrRNASites_OnePointMut___rRNASSU18S_218_psi

gccgatgtacgtgttccgtaagacggagctcaagcactccaagaccgagctcaacttcaa

ggagtggcaaaaggcctttaccgatgtgatgggcatggacgagctgtacaagtaaactag

tagcagacagatgcgccgtggatgccgacgggcgctgacccccttcgcgggggggatgcg

tgcagttatcagatcaaaaccaacccggtcagcccctcttggcctgagccacccgatcca

atgcttaaggtcaggtcactgcttgacggagccatagttggcgcgccattcgatatcgga

ccctttccctttagtgagggttaatgct

>AllrRNASites_OnePointMut___rRNASSU18S_406_psi

gccgatgtacgtgttccgtaagacggagctcaagcactccaagaccgagctcaacttcaa

ggagtggcaaaaggcctttaccgatgtgatgggcatggacgagctgtacaagtaaactag

tagcagacagatgcgccgtggatctttcgatggtagtcgccgtgcctaccatggtgacca

cgggggacggggaatcagggttcgattccggagagggatatctggcagccacccgatcca

atgcttaaggtcaggtcactgcttgacggagccatagttggcgcgccattcgatatcgga

ccctttccctttagtgagggttaatgct

>AllrRNASites_OnePointMut___rRNASSU18S_572_psi

gccgatgtacgtgttccgtaagacggagctcaagcactccaagaccgagctcaacttcaa

ggagtggcaaaaggcctttaccgatgtgatgggcatggacgagctgtacaagtaaactag

tagcagacagatgcgccgtggatacaggactctttcgaggccctgtaattggaatgagtc

cactgtaaatcctttaacgaggatccattggagggcaacgaacaagagccacccgatcca

atgcttaaggtcaggtcactgcttgacggagccatagttggcgcgccattcgatatcgga

ccctttccctttagtgagggttaatgct

>AllrRNASites_OnePointMut___rRNASSU18S_609_psi

gccgatgtacgtgttccgtaagacggagctcaagcactccaagaccgagctcaacttcaa

ggagtggcaaaaggcctttaccgatgtgatgggcatggacgagctgtacaagtaaactag

tagcagacagatgcgccgtggatcactttaaatcctttaacgaggatccattggagggca

agtcgggtgccagcagccgcggtaattccagctccaatatactcccagccacccgatcca

atgcttaaggtcaggtcactgcttgacggagccatagttggcgcgccattcgatatcgga

ccctttccctttagtgagggttaatgct

>AllrRNASites_OnePointMut___rRNASSU18S_649_psi

gccgatgtacgtgttccgtaagacggagctcaagcactccaagaccgagctcaacttcaa

ggagtggcaaaaggcctttaccgatgtgatgggcatggacgagctgtacaagtaaactag

tagcagacagatgcgccgtggatctggtgccagcagccgcggtaattccagctccaatag

cgtagattaaagttgctgcagttaaaaagctcgtagttttctcgccagccacccgatcca

atgcttaaggtcaggtcactgcttgacggagccatagttggcgcgccattcgatatcgga

ccctttccctttagtgagggttaatgct

>AllrRNASites_OnePointMut___rRNASSU18S_651_psi

gccgatgtacgtgttccgtaagacggagctcaagcactccaagaccgagctcaacttcaa

ggagtggcaaaaggcctttaccgatgtgatgggcatggacgagctgtacaagtaaactag

tagcagacagatgcgccgtggatggtgccagcagccgcggtaattccagctccaatagcg

tatagtaaagttgctgcagttaaaaagctcgtagttggttgtggctagccacccgatcca

atgcttaaggtcaggtcactgcttgacggagccatagttggcgcgccattcgatatcgga

ccctttccctttagtgagggttaatgct

>AllrRNASites_OnePointMut___rRNASSU18S_681_psi

gccgatgtacgtgttccgtaagacggagctcaagcactccaagaccgagctcaacttcaa

ggagtggcaaaaggcctttaccgatgtgatgggcatggacgagctgtacaagtaaactag

tagcagacagatgcgccgtggataatagcgtatattaaagttgctgcagttaaaaagctc

gtaggtggatcttgggagcgggcgggcggtccgccgcgggcggtaaagccacccgatcca

atgcttaaggtcaggtcactgcttgacggagccatagttggcgcgccattcgatatcgga

ccctttccctttagtgagggttaatgct

>AllrRNASites_OnePointMut___rRNASSU18S_686_psi

gccgatgtacgtgttccgtaagacggagctcaagcactccaagaccgagctcaacttcaa

ggagtggcaaaaggcctttaccgatgtgatgggcatggacgagctgtacaagtaaactag

tagcagacagatgcgccgtggatcgtatattaaagttgctgcagttaaaaagctcgtagt

tggagcttgggagcgggcgggcggtccgccgcgaggcgtctcgggcagccacccgatcca

atgcttaaggtcaggtcactgcttgacggagccatagttggcgcgccattcgatatcgga

ccctttccctttagtgagggttaatgct

>AllrRNASites_OnePointMut___rRNASSU18S_801_psi

gccgatgtacgtgttccgtaagacggagctcaagcactccaagaccgagctcaacttcaa

ggagtggcaaaaggcctttaccgatgtgatgggcatggacgagctgtacaagtaaactag

tagcagacagatgcgccgtggattcgatgctcttagctgagtgtcccgcggggcccgaag

cgttgactttgaaaaaattagagtgttcaaagcaggcccctcgcttagccacccgatcca

atgcttaaggtcaggtcactgcttgacggagccatagttggcgcgccattcgatatcgga

ccctttccctttagtgagggttaatgct

>AllrRNASites_OnePointMut___rRNASSU18S_814_psi

gccgatgtacgtgttccgtaagacggagctcaagcactccaagaccgagctcaacttcaa

ggagtggcaaaaggcctttaccgatgtgatgggcatggacgagctgtacaagtaaactag

tagcagacagatgcgccgtggatctgagtgtcccgcggggcccgaagcgtttactttgaa

aaaagtagagtgttcaaagcaggcccgagccgcctggagtctaaccagccacccgatcca

atgcttaaggtcaggtcactgcttgacggagccatagttggcgcgccattcgatatcgga

ccctttccctttagtgagggttaatgct

>AllrRNASites_OnePointMut___rRNASSU18S_815_psi

gccgatgtacgtgttccgtaagacggagctcaagcactccaagaccgagctcaacttcaa

ggagtggcaaaaggcctttaccgatgtgatgggcatggacgagctgtacaagtaaactag

tagcagacagatgcgccgtggattgagtgtcccgcggggcccgaagcgtttactttgaaa

aaatgagagtgttcaaagcaggcccgagccgcctggataaaatccgagccacccgatcca

atgcttaaggtcaggtcactgcttgacggagccatagttggcgcgccattcgatatcgga

ccctttccctttagtgagggttaatgct

>AllrRNASites_OnePointMut___rRNASSU18S_822_psi

gccgatgtacgtgttccgtaagacggagctcaagcactccaagaccgagctcaacttcaa

ggagtggcaaaaggcctttaccgatgtgatgggcatggacgagctgtacaagtaaactag

tagcagacagatgcgccgtggatcccgcggggcccgaagcgtttactttgaaaaaattag

agtggtcaaagcaggcccgagccgcctggataccgcagtcagtgtgagccacccgatcca

atgcttaaggtcaggtcactgcttgacggagccatagttggcgcgccattcgatatcgga

ccctttccctttagtgagggttaatgct

>AllrRNASites_OnePointMut___rRNASSU18S_863_psi

gccgatgtacgtgttccgtaagacggagctcaagcactccaagaccgagctcaacttcaa

ggagtggcaaaaggcctttaccgatgtgatgggcatggacgagctgtacaagtaaactag

tagcagacagatgcgccgtggatttcaaagcaggcccgagccgcctggataccgcagcta

ggaagaatggaataggaccgcggttctattttgttggttgagctctagccacccgatcca

atgcttaaggtcaggtcactgcttgacggagccatagttggcgcgccattcgatatcgga

ccctttccctttagtgagggttaatgct

>AllrRNASites_OnePointMut___rRNASSU18S_866_psi

gccgatgtacgtgttccgtaagacggagctcaagcactccaagaccgagctcaacttcaa

ggagtggcaaaaggcctttaccgatgtgatgggcatggacgagctgtacaagtaaactag

tagcagacagatgcgccgtggataaagcaggcccgagccgcctggataccgcagctagga

ataagggaataggaccgcggttctattttgttggtttttagctaccagccacccgatcca

atgcttaaggtcaggtcactgcttgacggagccatagttggcgcgccattcgatatcgga

ccctttccctttagtgagggttaatgct

>AllrRNASites_OnePointMut___rRNASSU18S_918_psi

gccgatgtacgtgttccgtaagacggagctcaagcactccaagaccgagctcaacttcaa

ggagtggcaaaaggcctttaccgatgtgatgggcatggacgagctgtacaagtaaactag

tagcagacagatgcgccgtggatcgcggttctattttgttggttttcggaactgaggcca

tgatgaagagggacggccgggggcattcgtattgcgcccttgccacagccacccgatcca

atgcttaaggtcaggtcactgcttgacggagccatagttggcgcgccattcgatatcgga

ccctttccctttagtgagggttaatgct

>AllrRNASites_OnePointMut___rRNASSU18S_966_psi

gccgatgtacgtgttccgtaagacggagctcaagcactccaagaccgagctcaacttcaa

ggagtggcaaaaggcctttaccgatgtgatgggcatggacgagctgtacaagtaaactag

tagcagacagatgcgccgtggatgacggccgggggcattcgtattgcgccgctagaggtg

aaatgccttggaccggcgcaagacggaccagagcgaaattcccctaagccacccgatcca

atgcttaaggtcaggtcactgcttgacggagccatagttggcgcgccattcgatatcgga

ccctttccctttagtgagggttaatgct

>AllrRNASites_OnePointMut___rRNASSU18S_1005_psi

gccgatgtacgtgttccgtaagacggagctcaagcactccaagaccgagctcaacttcaa

ggagtggcaaaaggcctttaccgatgtgatgggcatggacgagctgtacaagtaaactag

tagcagacagatgcgccgtggatattccttggaccggcgcaagacggaccagagcgaaag

cattggccaagaatgttttcattaatcaagaacgaaagtgctgtgaagccacccgatcca

atgcttaaggtcaggtcactgcttgacggagccatagttggcgcgccattcgatatcgga

ccctttccctttagtgagggttaatgct

>AllrRNASites_OnePointMut___rRNASSU18S_1057_psi

gccgatgtacgtgttccgtaagacggagctcaagcactccaagaccgagctcaacttcaa

ggagtggcaaaaggcctttaccgatgtgatgggcatggacgagctgtacaagtaaactag

tagcagacagatgcgccgtggatttttcattaatcaagaacgaaagtcggaggttcgaag

acgagcagataccgtcgtagttccgaccataaacgatgctagggtgagccacccgatcca

atgcttaaggtcaggtcactgcttgacggagccatagttggcgcgccattcgatatcgga

ccctttccctttagtgagggttaatgct

>AllrRNASites_OnePointMut___rRNASSU18S_1082_psi

gccgatgtacgtgttccgtaagacggagctcaagcactccaagaccgagctcaacttcaa

ggagtggcaaaaggcctttaccgatgtgatgggcatggacgagctgtacaagtaaactag

tagcagacagatgcgccgtggatggaggttcgaagacgatcagataccgtcgtagttccg

accagaaacgatgccgaccggcgatgcggcggcgttataccatgacagccacccgatcca

atgcttaaggtcaggtcactgcttgacggagccatagttggcgcgccattcgatatcgga

ccctttccctttagtgagggttaatgct

>AllrRNASites_OnePointMut___rRNASSU18S_1175_psi

gccgatgtacgtgttccgtaagacggagctcaagcactccaagaccgagctcaacttcaa

ggagtggcaaaaggcctttaccgatgtgatgggcatggacgagctgtacaagtaaactag

tagcagacagatgcgccgtggatagcttccgggaaaccaaagtctttgggttccgggggg

agtagggttgcaaagctgaaacttaaaggaattgacgggtgccatcagccacccgatcca

atgcttaaggtcaggtcactgcttgacggagccatagttggcgcgccattcgatatcgga

ccctttccctttagtgagggttaatgct

>AllrRNASites_OnePointMut___rRNASSU18S_1239_psi

gccgatgtacgtgttccgtaagacggagctcaagcactccaagaccgagctcaacttcaa

ggagtggcaaaaggcctttaccgatgtgatgggcatggacgagctgtacaagtaaactag

tagcagacagatgcgccgtggatggaattgacggaagggcaccaccaggagtggagcctg

cggcgtaatttgactcaacacgggaaacctcacccggccagaactgagccacccgatcca

atgcttaaggtcaggtcactgcttgacggagccatagttggcgcgccattcgatatcgga

ccctttccctttagtgagggttaatgct

>AllrRNASites_OnePointMut___rRNASSU18S_1245_psi

gccgatgtacgtgttccgtaagacggagctcaagcactccaagaccgagctcaacttcaa

ggagtggcaaaaggcctttaccgatgtgatgggcatggacgagctgtacaagtaaactag

tagcagacagatgcgccgtggatgacggaagggcaccaccaggagtggagcctgcggctt

aattggactcaacacgggaaacctcacccggcccggacaaggtacgagccacccgatcca

atgcttaaggtcaggtcactgcttgacggagccatagttggcgcgccattcgatatcgga

ccctttccctttagtgagggttaatgct

>AllrRNASites_OnePointMut___rRNASSU18S_1348_psi

gccgatgtacgtgttccgtaagacggagctcaagcactccaagaccgagctcaacttcaa

ggagtggcaaaaggcctttaccgatgtgatgggcatggacgagctgtacaagtaaactag

tagcagacagatgcgccgtggattttctcgattccgtgggtggtggtgcatggccgttct

tagtgggtggagcgatttgtctggttaattccgataaccttcactcagccacccgatcca

atgcttaaggtcaggtcactgcttgacggagccatagttggcgcgccattcgatatcgga

ccctttccctttagtgagggttaatgct

>AllrRNASites_OnePointMut___rRNASSU18S_1368_psi

gccgatgtacgtgttccgtaagacggagctcaagcactccaagaccgagctcaacttcaa

ggagtggcaaaaggcctttaccgatgtgatgggcatggacgagctgtacaagtaaactag

tagcagacagatgcgccgtggattggtgcatggccgttcttagttggtggagcgatttgt

ctgggtaattccgataacgaacgagactctggcatgcttcgtatgtagccacccgatcca

atgcttaaggtcaggtcactgcttgacggagccatagttggcgcgccattcgatatcgga

ccctttccctttagtgagggttaatgct

>AllrRNASites_OnePointMut___rRNASSU18S_1446_psi

gccgatgtacgtgttccgtaagacggagctcaagcactccaagaccgagctcaacttcaa

ggagtggcaaaaggcctttaccgatgtgatgggcatggacgagctgtacaagtaaactag

tagcagacagatgcgccgtggattagttacgcgacccccgagcggtcggcgtcccccaac

ttctgagagggacaagtggcgttcagccacccgagattctagcattagccacccgatcca

atgcttaaggtcaggtcactgcttgacggagccatagttggcgcgccattcgatatcgga

ccctttccctttagtgagggttaatgct

>AllrRNASites_OnePointMut___rRNASSU18S_1626_psi

gccgatgtacgtgttccgtaagacggagctcaagcactccaagaccgagctcaacttcaa

ggagtggcaaaaggcctttaccgatgtgatgggcatggacgagctgtacaagtaaactag

tagcagacagatgcgccgtggatgttgaaccccattcgtgatggggatcggggattgcaa

ttatgccccatgaacgaggaattcccagtaagtgcgggcccttggcagccacccgatcca

atgcttaaggtcaggtcactgcttgacggagccatagttggcgcgccattcgatatcgga

ccctttccctttagtgagggttaatgct

>AllrRNASites_OnePointMut___rRNASSU18S_1644_psi

gccgatgtacgtgttccgtaagacggagctcaagcactccaagaccgagctcaacttcaa

ggagtggcaaaaggcctttaccgatgtgatgggcatggacgagctgtacaagtaaactag

tagcagacagatgcgccgtggattggggatcggggattgcaattattccccatgaacgag

gaatgcccagtaagtgcgggtcataagcttgcgttgatgagtataaagccacccgatcca

atgcttaaggtcaggtcactgcttgacggagccatagttggcgcgccattcgatatcgga

ccctttccctttagtgagggttaatgct

>AllrRNASites_OnePointMut___rRNASSU18S_1693_psi

gccgatgtacgtgttccgtaagacggagctcaagcactccaagaccgagctcaacttcaa

ggagtggcaaaaggcctttaccgatgtgatgggcatggacgagctgtacaagtaaactag

tagcagacagatgcgccgtggatagtgcgggtcataagcttgcgttgattaagtccctgc

ccttggtacacaccgcccgtcgctactaccgattggatcgatatgcagccacccgatcca

atgcttaaggtcaggtcactgcttgacggagccatagttggcgcgccattcgatatcgga

ccctttccctttagtgagggttaatgct

>AllrRNASites_OnePointMut___snRNAU1_5_psi

gccgatgtacgtgttccgtaagacggagctcaagcactccaagaccgagctcaacttcaa

ggagtggcaaaaggcctttaccgatgtgatgggcatggacgagctgtacaagtaaactag

tagcagacagatgcgccgtggatacggacatctaccacgaaattgttatagaagttgcct

atacgtacctggcaggggagataccatgatcacgaaggatcttcatagccacccgatcca

atgcttaaggtcaggtcactgcttgacggagccatagttggcgcgccattcgatatcgga

ccctttccctttagtgagggttaatgct

>AllrRNASites_OnePointMut___snRNAU1_6_psi

gccgatgtacgtgttccgtaagacggagctcaagcactccaagaccgagctcaacttcaa

ggagtggcaaaaggcctttaccgatgtgatgggcatggacgagctgtacaagtaaactag

tagcagacagatgcgccgtggatcggacatctaccacgaaattgttatagaagttgccta

tactgacctggcaggggagataccatgatcacgaaggtcatacctgagccacccgatcca

atgcttaaggtcaggtcactgcttgacggagccatagttggcgcgccattcgatatcgga

ccctttccctttagtgagggttaatgct

>AllrRNASites_OnePointMut___snRNAU12_18_psi

gccgatgtacgtgttccgtaagacggagctcaagcactccaagaccgagctcaacttcaa

ggagtggcaaaaggcctttaccgatgtgatgggcatggacgagctgtacaagtaaactag

tagcagacagatgcgccgtggatacgaaattgttatagaagttgccttgccttaaactta

tgaggaaggaaaataacgattcggggtgacgcccgaataaaatcgaagccacccgatcca

atgcttaaggtcaggtcactgcttgacggagccatagttggcgcgccattcgatatcgga

ccctttccctttagtgagggttaatgct

>AllrRNASites_OnePointMut___snRNAU12_27_psi

gccgatgtacgtgttccgtaagacggagctcaagcactccaagaccgagctcaacttcaa

ggagtggcaaaaggcctttaccgatgtgatgggcatggacgagctgtacaagtaaactag

tagcagacagatgcgccgtggatttatagaagttgccttgccttaaacttatgagtaagg

aaaagaacgattcggggtgacgcccgaatcctcactgcacttcagtagccacccgatcca

atgcttaaggtcaggtcactgcttgacggagccatagttggcgcgccattcgatatcgga

ccctttccctttagtgagggttaatgct

>AllrRNASites_OnePointMut___snRNAU4_4_psi

gccgatgtacgtgttccgtaagacggagctcaagcactccaagaccgagctcaacttcaa

ggagtggcaaaaggcctttaccgatgtgatgggcatggacgagctgtacaagtaaactag

tagcagacagatgcgccgtggattacggacatctaccacgaaattgttatagaagttgcc

tagcgttgcgcagtggcagtatcgtagccaatgaggtcttagtgcaagccacccgatcca

atgcttaaggtcaggtcactgcttgacggagccatagttggcgcgccattcgatatcgga

ccctttccctttagtgagggttaatgct

>AllrRNASites_OnePointMut___snRNAU4_72_psi

gccgatgtacgtgttccgtaagacggagctcaagcactccaagaccgagctcaacttcaa

ggagtggcaaaaggcctttaccgatgtgatgggcatggacgagctgtacaagtaaactag

tagcagacagatgcgccgtggattgaggtctatccgaggcgcgattattgctaattgaaa

acttgtcccaataccccgccgtgacgacttgcaatatatacggatgagccacccgatcca

atgcttaaggtcaggtcactgcttgacggagccatagttggcgcgccattcgatatcgga

ccctttccctttagtgagggttaatgct

>AllrRNASites_OnePointMut___snRNAU4_79_psi

gccgatgtacgtgttccgtaagacggagctcaagcactccaagaccgagctcaacttcaa

ggagtggcaaaaggcctttaccgatgtgatgggcatggacgagctgtacaagtaaactag

tagcagacagatgcgccgtggattatccgaggcgcgattattgctaattgaaaacttttc

ccaagaccccgccgtgacgacttgcaatatagtcggcacaagcacaagccacccgatcca

atgcttaaggtcaggtcactgcttgacggagccatagttggcgcgccattcgatatcgga

ccctttccctttagtgagggttaatgct

>AllrRNASites_OnePointMut___snRNAU4atac_12_psi

gccgatgtacgtgttccgtaagacggagctcaagcactccaagaccgagctcaacttcaa

ggagtggcaaaaggcctttaccgatgtgatgggcatggacgagctgtacaagtaaactag

tagcagacagatgcgccgtggattctaccacgaaattgttatagaagttgcctaaccatc

ctttgcttggggttgcgctactgtccaatgagcgcatagacacgccagccacccgatcca

atgcttaaggtcaggtcactgcttgacggagccatagttggcgcgccattcgatatcgga

ccctttccctttagtgagggttaatgct

>AllrRNASites_OnePointMut___snRNAU5_43_psi

gccgatgtacgtgttccgtaagacggagctcaagcactccaagaccgagctcaacttcaa

ggagtggcaaaaggcctttaccgatgtgatgggcatggacgagctgtacaagtaaactag

tagcagacagatgcgccgtggattactctggtttctcttcagatcgcataaatctttcgc

ctttgactaaagatttccgtggagaggaacaactctgagcggaactagccacccgatcca

atgcttaaggtcaggtcactgcttgacggagccatagttggcgcgccattcgatatcgga

ccctttccctttagtgagggttaatgct

>AllrRNASites_OnePointMut___snRNAU5_46_psi

gccgatgtacgtgttccgtaagacggagctcaagcactccaagaccgagctcaacttcaa

ggagtggcaaaaggcctttaccgatgtgatgggcatggacgagctgtacaagtaaactag

tagcagacagatgcgccgtggattctggtttctcttcagatcgcataaatctttcgcctt

ttacgaaagatttccgtggagaggaacaactctgagtctatcttacagccacccgatcca

atgcttaaggtcaggtcactgcttgacggagccatagttggcgcgccattcgatatcgga

ccctttccctttagtgagggttaatgct

>AllrRNASites_OnePointMut___snRNAU5_53_psi

gccgatgtacgtgttccgtaagacggagctcaagcactccaagaccgagctcaacttcaa

ggagtggcaaaaggcctttaccgatgtgatgggcatggacgagctgtacaagtaaactag

tagcagacagatgcgccgtggattctcttcagatcgcataaatctttcgccttttactaa

agatgtccgtggagaggaacaactctgagtcttaacccattagattagccacccgatcca

atgcttaaggtcaggtcactgcttgacggagccatagttggcgcgccattcgatatcgga

ccctttccctttagtgagggttaatgct

>AllrRNASites_OnePointMut___snRNAU6_31_psi

gccgatgtacgtgttccgtaagacggagctcaagcactccaagaccgagctcaacttcaa

ggagtggcaaaaggcctttaccgatgtgatgggcatggacgagctgtacaagtaaactag

tagcagacagatgcgccgtggatagaagttgcctgtgctcgcttcggcagcacatatact

aaaagtggaacgatacagagaagatttagcatggcccccctagggtagccacccgatcca

atgcttaaggtcaggtcactgcttgacggagccatagttggcgcgccattcgatatcgga

ccctttccctttagtgagggttaatgct

>AllrRNASites_OnePointMut___snRNAU6_40_psi

gccgatgtacgtgttccgtaagacggagctcaagcactccaagaccgagctcaacttcaa

ggagtggcaaaaggcctttaccgatgtgatgggcatggacgagctgtacaagtaaactag

tagcagacagatgcgccgtggatctgtgctcgcttcggcagcacatatactaaaattgga

acgagacagagaagatttagcatggcccctgcgcaaggatgtgatgagccacccgatcca

atgcttaaggtcaggtcactgcttgacggagccatagttggcgcgccattcgatatcgga

ccctttccctttagtgagggttaatgct

>AllrRNASites_OnePointMut___snRNAU6_87_psi

gccgatgtacgtgttccgtaagacggagctcaagcactccaagaccgagctcaacttcaa

ggagtggcaaaaggcctttaccgatgtgatgggcatggacgagctgtacaagtaaactag

tagcagacagatgcgccgtggatgaagatttagcatggcccctgcgcaaggatgacacgc

aaatgcgtgaagcgttccatatttttgattatttctgggaatggacagccacccgatcca

atgcttaaggtcaggtcactgcttgacggagccatagttggcgcgccattcgatatcgga

ccctttccctttagtgagggttaatgct

>AllrRNASites_OnePointMut___snRNAU6atac_83_psi

gccgatgtacgtgttccgtaagacggagctcaagcactccaagaccgagctcaacttcaa

ggagtggcaaaaggcctttaccgatgtgatgggcatggacgagctgtacaagtaaactag

tagcagacagatgcgccgtggataggatggaagaggccctcgggcctgacaacacgcata

cggtgaaggcattgccacctacttcgtggcatctaaccttgtagggagccacccgatcca

atgcttaaggtcaggtcactgcttgacggagccatagttggcgcgccattcgatatcgga

ccctttccctttagtgagggttaatgct
