# Supplementary material for: A systematic dissection of determinants and consequences of snoRNA-guided pseudouridylation of human mRNA
Source: Nucleic Acids Res. 2022 May 10;50(9):4900–16. doi: 10.1093/nar/gkac347 (PMC9122591; doi:10.1093/nar/gkac347)
Supplement: gkac347_Supplemental_Files [file gkac347_supplemental_files.zip › Nir_etal_TableS8.docx]

**Supplementary Table S8:** Oligos used in this study

| Lib cloning polII FW | 5’- TCAGTCGCCGCTGCCAGATCGCGGTACTAGTACGTGACAGATGCGCCGTGGAT - 3’ |
| --- | --- |
| Lib cloning polII RV | 5’ - TTGTTCCGCCGCTTCGCTGACTGTGGGCGCGCCTATAGCATTGGATCGGGTGGCT - 3’ |
| Lib cloning polI FW | 5’ - CTATAGAATACTCAAGCTTGgacagatgcgccgtggat - 3’ |
| Lib cloning polI RV | 5’ - GCTCGGTACCCGGGGATCCTCgcattggatcgggtggct - 3’ |
| PolII RT primer | 5’ - AGCATTAACCCTCACTAAAGGGAAAGG -3’ |
| PolI RT primer | 5’ - CCAGTGAATTGTAATACGACTCACTATAGG - 3’ |
| Subset specific inner PCR | 5’ - gcattggatcgggtggct - 3’ |
| subset specific (G10) | 5’ - CAAGCAGAAGACGGCATACGAGATCAACGGTCGTGACTGGAGTTCAGACGTGTGCTCTTCCGATCTgcattggatcgggtggct - 3’ |
| 2P_universal primer | 5’ - AATGATACGGCGACCACCGAGATCTACACTCTTTCCCTACACGACGCTCTTCCGATCT - 3’ |
| Psi-reporter-FW | 5’ - GCTTGACTAATACGACTCACTATAGGGAAGCACCCCCCCGCAGCAACGAGACGCCACAAGCCCACTVVVVVVGACCAACAAGAGCGAAC - 3’ |
| Psi-reporter-RV | 5’ - AGACGTGTGCTCTTCCGATCTTTGGTGCCGGTTGTTGCGCTTTTGCGCCTTGTTCGTCGTGGTTTTGGGTTCGCTCTTGTTGGTC -3 |
| ACA61 CFTR hyb only FW | 5’ – gttgatcagcttaatcctcctgaTGCCTAGTccatcggatctgaacactggtcttg – 3’ |
| ACA61 CFTR hyb only RV | 5’ – gcttcactattacttttcctccttttaGCGccaccaagaccagtgttcagatcc – 3’ |
| ACA61 gibson vector FW | 5’ – aggaaaagtaatagtgaagctggcc – 3’ |
| ACA61 gibson vector FW | 5’ – aggaggattaagctgatcaactttacc – 3’ |
| DMD hyb only FW | 5’ - ggtaaagttgatcagcttaatcctcctgaAATGCTGTccatcggatctgaacactggtcttggtggAGGtaaaaggaggaaaagtaatagtgaagc – 3’ |
| H2A RV | 5’ – ggcccacggggaactggagacc – 3’ |
| GFP reporter Flag FW | 5′-GCTCTAGA*TAATACGACTCACTATA*GGGGGCCACC**ATG**GACTACAAGGACGACGACGATAAGGTGAGCAAGGGCGAGG-3′ |
| GFP reporter Flag RV | 5′-mCmGTCCTCCTTGAAGTCGATGCCCTTCAGCTC-3′ |
| DMD readthrough control | 5’ -**GCAGG***AU*UUUCCU****UCA****CAGC*AUUUAGGCAAAUAA(A)n - 3’ |
| DMD Ψ-PTC | 5’ -**GCAGG***AU*UUUCCU****ΨGA****CAGCAUU*UAGGCAAAUAA(A)n - 3’ |
| DMD U-PTC | 5’ - **GCAGG***AU*UUUCCU****UGA****CAGCAUU*UAGGCAAAUAA(A)n - 3’ |
| CFTR readthrough control | 5’ - **GCAGG***A*UAUCGCG****UAU****CUAGGCAUAGGC*AAAUAA(A)n - 3’ |
| CFTR Ψ-PTC | 5’ -**GCAGG***A*UAUCGCG****ΨAA****CUAGGCAUAGGC*AAAUAA(A)n - 3’ |
| CFTR U-PTC | 5’ - **GCAGG***A*UAUCGCG****UAA****CUAGGCAUAGGC*AAAUAA(A)n - 3’ |
