# Supplementary material for: A systematic dissection of determinants and consequences of snoRNA-guided pseudouridylation of human mRNA
Source: Nucleic Acids Res. 2022 May 10;50(9):4900–16. doi: 10.1093/nar/gkac347 (PMC9122591; doi:10.1093/nar/gkac347)
Supplement: gkac347_Supplemental_Files [file gkac347_supplemental_files.zip › Nir_et_al_Supplementary_Figures.docx]

**Supplementary Figures**


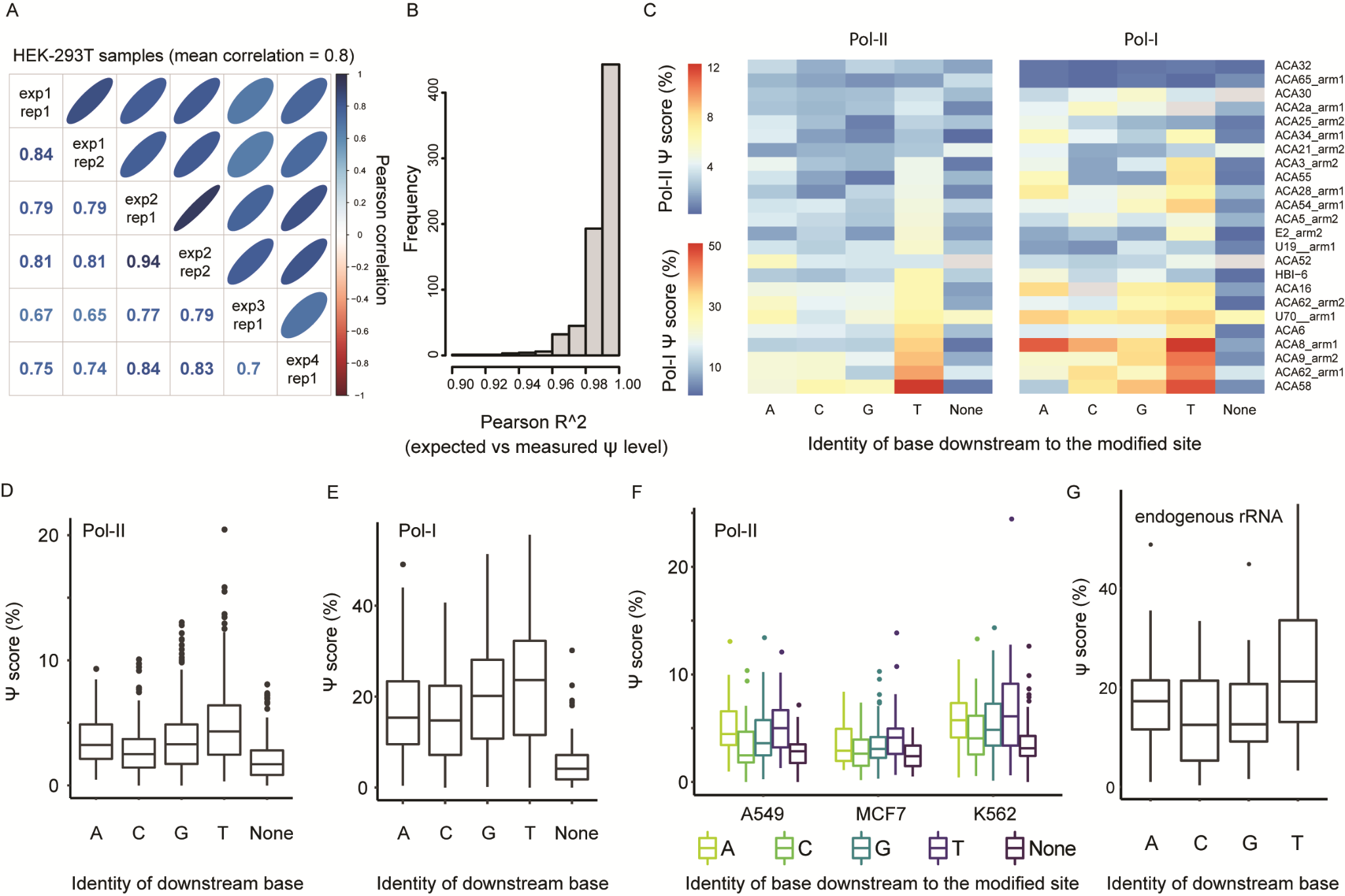


**Figure S1. Ψ quantification across experiments and analysis of the effect of the downstream nucleotide.** (**A**) Library-specific Ψ-Seq was conducted on HEK-293T samples (n=6) transfected with Pol-II-promoter libraries. Pearson’s correlation between all samples is presented as correlation value (number in blue) and distribution of data (ellipse). Overall, four experiments (‘exp’) were designed, each with up to two replicates (‘rep’). (**B**) Histogram representing the distribution of R^2 of pearson correlation calculated between expected and measured Ψ level of each of the 729 reporter sequences described in **Figure 1J,K**. (**C**) Heatmap of Ψ-score of synthetic snoRNA targets with distinct bases downstream to the modified uridine as measured in HEK-293T cells transfected with Pol-II- or Pol-I-promoter libraries (left and right, respectively). Each row represents constructs targeted by a specific snoRNA (listed on the right). Columns represent the identity of the base downstream to the modification site. Shown are constructs with a minimal difference of 2% in Ψ-score between at least two columns (in both Pol-II and Pol-I experiments). Rows are ordered according to maximal Ψ-score in the Pol-II library. N=2. (**D-F**) Ψ-score values of synthetic snoRNA targets with distinct bases downstream to the modified uridine as measured in HEK-293T cells transfected with Pol-II- (**D**) or Pol-I-promoter (**E**) libraries (n=6 and 2, respectively) or in A549, MCF7 and K562 cells (**F**) transfected with Pol-II-promoter libraries (n=1). (**G**) Ψ-score of known Ψ sites in 18S and 28S measured by Ψ-Seq of total RNA purified from HEK-293T cells. Sites are binned according to the base found downstream to the modified site. Boxplot parameters are as in **Figure 1**. N=3.

 
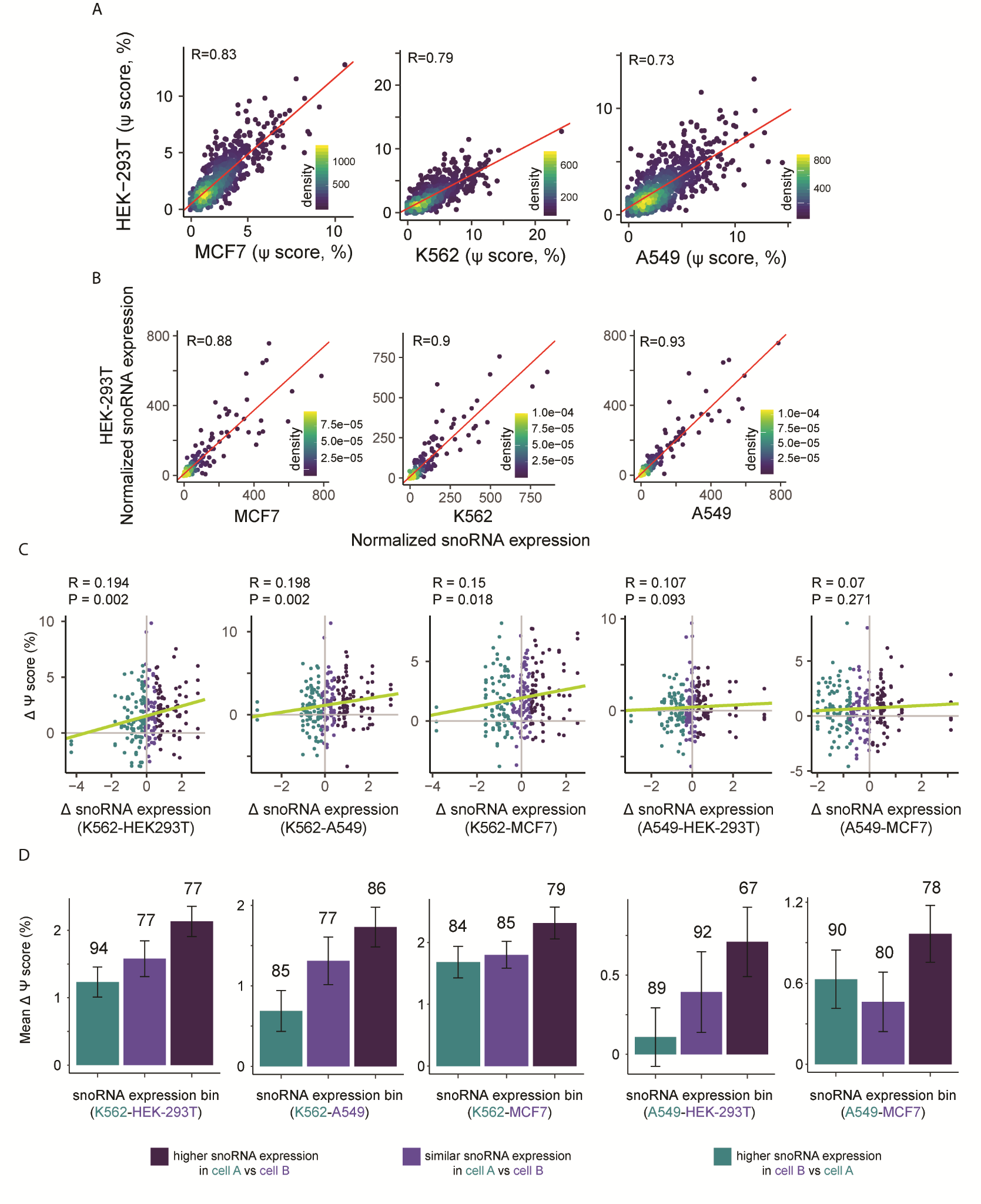


**Figure S2. A correlation between Ψ levels and snoRNA abundance across different cell lines.** (**A-B**) Targeted Ψ-Seq was conducted on the four indicated cell lines each transfected with Pol-II-promoter libraries (n=1). Colors indicate Kernel density estimate of data distribution (arbitrary units).  Regression line in red. (**A**)  Pearson’s correlation between Ψ-score in HEK-293T cells versus Ψ-score in MCF7, K562 or A549 cells is plotted. (**B**) Pearson’s correlation between snoRNA abundance in HEK-293T cells versus snoRNA abundance in MCF7, K562 or A549 cells is plotted. (**C**) Same analysis as in **Figure 4B**, shown for all other cell line pairs. R, P: Spearman’s correlation and p value, respectively. (**D**) Same analysis as in **Figure 4C**, shown for all other cell line pairs.


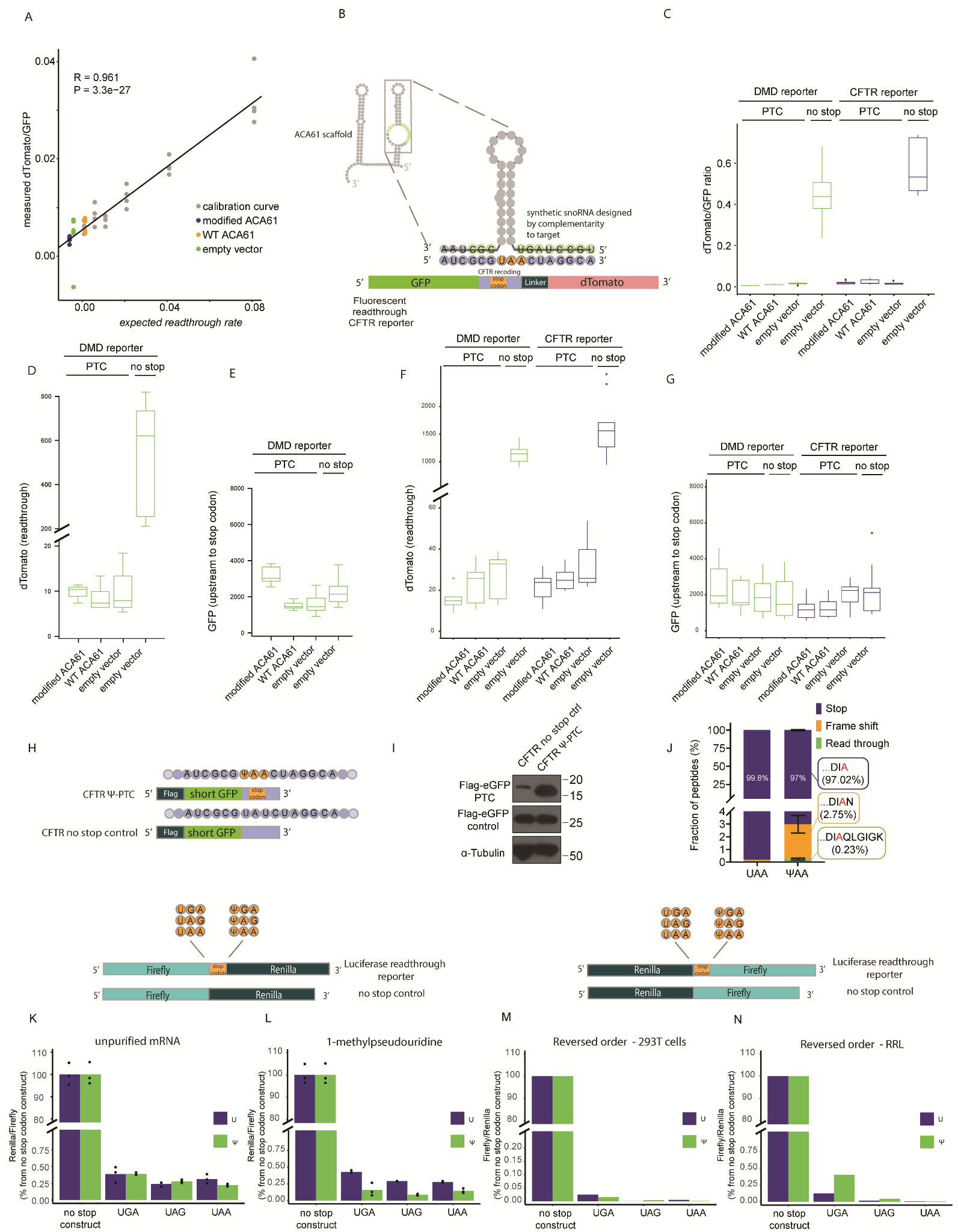


**Figure S3. Ψ in stop codons does not result in significant stop codon readthrough in HEK-293T cells.** (**A**) Calibration curve for the DMD reporter. 0%, 0.5%, 1%, 2%, 4% and 8% of the reporter that does not contain a PTC (DMD no stop reporter) were mixed with the DMD PTC reporter and transfected to HEK-293T cells. The fluorescence was measured in the plate reader alongside the samples, and the levels of readthrough of the samples was estimated from the calibration curve. (**B**) Design of a synthetic snoRNA to target a premature termination codon (PTC) causing cystic fibrosis (CFTR). The targeting strategy was the same as for the DMD reporter (**Figure 5A**). (**C**) Readthrough plate reader measurements in an additional experiment exhibited similar results for the DMD reporter and CFTR reporter with their corresponding targeting snoRNAs. (**D-G**) Separate, non-normalized, fluorescent plate reader measurements of the experiments in **Figure 5C** and **Supplementary Figure S3C**. (**D**) dTomato measurement of **Figure 5C**. (**E**) GFP measurement of **Figure 5C**. (**F**) dTomato measurement of **Supplementary Figure S3C**. (**G**) GFP measurement of **Supplementary Figure S3C**. (**H**) Constructs for quantification of readthrough of a 100% pseudouridylated CFTR stop codon. The cloning strategy was the same as for the DMD PTC (**Figure 5D**). (**I**) Proteins were purified from HEK-293T cells transfected with the synthetic CFTR RNAs and a longer control RNA, also tagged with FLAG, and subjected to Western blot analysis. Top panel: Flag antibody, product of the synthetic reporters. Middle panel: Flag antibody, transfection control. Bottom panel: Tubulin antibody, loading control. (**J**) LC-MS/MS analysis of the constructs containing the CFTR PTC, either non-pseudouridylated (left) or pseudouridylated (right) (sensitivity of the assay ranged between 0.03%-0.3%, data not shown). (**K-N**) Dual luciferase assay measuring the readthrough of the various stop codons. The scheme above each panel illustrates the constructs used in the dual luciferase assay. In **K** and **L**, RNA encoding firefly luciferase, followed by either of the stop codons or none and Renilla luciferase was in vitro transcribed to contain either uridine or Ψ in **K**, and uridine or 1-methyl-Ψ in **L**. In **M** and **N**, the order of the luciferases is reversed. (**K**) Measurements as in **Figure 5F**, using unpurified RNA. (**L**) HEK-293T cells were transfected with mRNA in vitro transcribed with either 100% 1-methylpseudouridine or uridine, which was HPLC-purified. (**M**) Non-purified in vitro transcribed mRNAs from the reversed order constructs were transfected to HEK-293T cells. (**N**) Non-purified in vitro transcribed mRNAs from the reversed order constructs were translated using rabbit reticulocyte lysate (RRL).


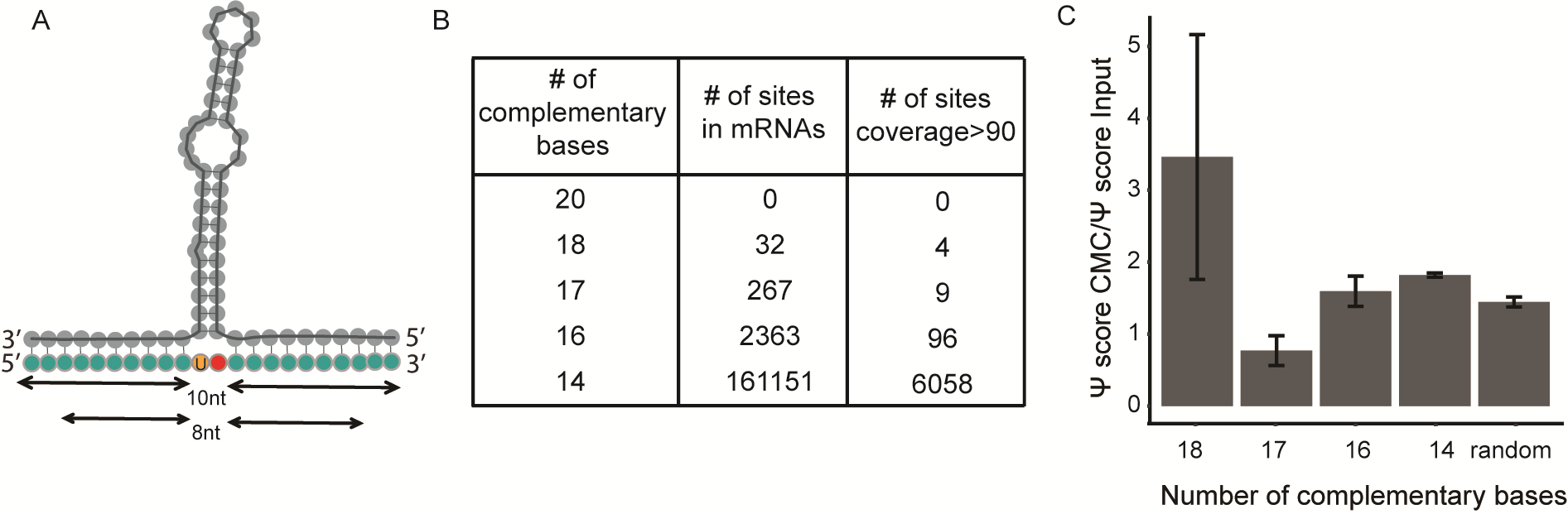


**Figure S4. Putative endogenous mRNA targets of human H/ACA box snoRNAs**. (**A**) For each snoRNA, we searched for human mRNAs with a complete match to the 10 (or 8) bases immediately surrounding the modified site from both sides (indicated by arrows). No such mRNAs were found. (**B**) For each snoRNA, we searched for mRNAs with 14-20 bases complementarity, by allowing mismatches to occur within the stretch of 20 nucleotides surrounding the modified site (green bases in **A**). The table displays the number of mRNA:snoRNA pairs found in the analysis. (**C**) Using data from Carlile et al., pseudouridine level of the sites found in **B** was quantified as a function of the number of bases complementary to human snoRNAs. The “random” group represents pseudouridine levels at 1,000 random uridine positions within human mRNAs. The barplot depicts mean ± SEM of sites with coverage >90 reads for both CMC- and mock-treated samples (i.e., “treatment” and “input”, respectively).

**Supplementary Tables**

**Supplementary Table S1**: Sequences of twist library and endogenous rRNAs, used as transcriptome for alignment

**Supplementary Table S2**: Table of Ψ-score, stops and coverage of each construct in each library. Used in the analysis shown in **Figs. 1-4** and **supplementary Figs. S1,S2**.

**Supplementary Table S3**: Table of Ψ-score of in vitro transcribed reporter sequences. Used in the analysis shown in **Figure 1K,L**.

**Supplementary Table S4**: Table of expression of snoRNAs and coding genes from HEK-293T cells treated with siControl or siDKC1. Used in the analysis shown in **Figure 2B,C**.

**Supplementary Table S5**: Table of expression of snoRNAs from HEK-293T cells overexpressing ACA21 and ACA61 or an empty vector control. Used in the analysis shown in **Figure 2F**.

**Supplementary Table S6**: Table of Ψ-score, stops and coverage of rRNA in total RNA samples. Used in the analysis shown in **supplementary Figure S1G**.

**Supplementary Table S7**: Table of expression of snoRNAs in various cell lines and the corresponding Ψ-scores of their target constructs in each cell line. Used in the analysis shown in **Figure 4** and **supplementary Figures S1F and S2**.

**Supplementary Table S8**: Oligos used in this study.
